# ~100% upcycling of chlorinated/fluorinated plastic mixtures to H_2_ and nanotubes over FeNi/Ni/C by microwave catalysis

**DOI:** 10.1038/s41467-026-73141-w

**Published:** 2026-05-15

**Authors:** Jun Zhao, Jianlong Yang, Duanda Wang, Huanrong Zhang, Qianqian Jia, Lei Zhang, Zhenguo An, Mianqi Xue, Haijiao Xie, Wangjing Ma, Lu Zhang, Sui Zhao, Junwang Tang

**Affiliations:** 1 State Key Laboratory of Cryogenic Science and Technology, Technical Institute of Physics and Chemistry, Chinese Academy of Sciences, Beijing, China; 2 Industrial Catalysis Center, Department of Chemical Engineering, Tsinghua University, Beijing, China; 3 Yulin Innovation Institute of Clean Energy, Yulin, China; 4 Laboratory of Bio-Inspired Smart Interface Science, Technical Institute of Physics and Chemistry, Chinese Academy of Sciences, Beijing, China; 5 University of Chinese Academy of Sciences, Beijing, China; 6 National Engineering Research Center for Engineering Plastics, Technical Institute of Physics and Chemistry, Chinese Academy of Sciences, Beijing, China; 7 Hangzhou Yanqu Information Technology Co., Ltd., Hangzhou, China

**Keywords:** Heterogeneous catalysis, Chemical engineering, Catalytic mechanisms

## Abstract

The catalytic upcycling of plastic mixtures into H_2_ and carbon nanotubes (CNTs) represents a transformative strategy for advancing plastic management and the circular economy. Herein, an in-situ constructed high-performance FeNi/Ni/C catalyst combined with prompt microwave catalysis enables efficient conversion of LDPE-PVC, LDPE-HDPE-PP-PS-PVC-PTFE and landfilled waste mixture into valuable H_2_ and CNTs, resulting in near-quantitative atom economy (i.e., H_2_ and carbon efficiencies are nearly 100%) together with H_2_ and CNTs yields reaching >922 mmol g^-1^_catalyst_ and >10600 mg g^-1^_catalyst_, respectively. Furthermore, this microwave catalysis strategy presents an excellent stability (>35 cycles) via Cl/F-based regeneration and energy efficiency (80% reduction vs. traditional thermal catalysis), alongside a high turnover number of 107.6 mol_carbon_ mol^-1^_NiFe_. Fundamentally, the Ni species contribute to C-H breaking, and Fe is beneficial for CNT formation during plastic decomposition. Here, we show a cost-effective platform for upcycling of chlorinated/fluorinated plastic mixtures, aligning with resource circularity objectives.

## Abstract (web summary)

Turning mixed plastic waste into useful products is a major challenge for a circular economy. This study uses a microwave-driven iron-nickel catalyst to convert even halogenated plastic mixtures into hydrogen and carbon nanotubes with high yield and stability.

## Introduction

Plastics, predominantly derived from fossil resources, serve as an indispensable material in modern society. Yet over 70% of post-consumer plastic waste is either landfilled or discarded, driving environmental pollution and critical resources underutilization^1–3^. From a sustainability perspective, plastic waste represents an untapped resource reservoir for synthesizing value-added chemicals^4^. Among various plastic waste recycling methods, chemical recycling has emerged as a promising strategy due to its flexibility, sustainability, and potential for circularity. In recent years, several innovative catalytic upcycling approaches have been proposed. These can selectively convert plastic waste into high-value products, such as lubricants, fuels, H_2_, and advanced nano-carbon materials^5–11^, offering a promising strategy for the upcycling of plastic waste.

Carbon nanotubes (CNTs), composed of hexagonally arranged carbon atoms forming cylindrical architectures, are distinctive carbon-based nanostructures. They possess high electrical conductivity, exceptional tensile strength, and low density, supporting broad applications in power storage, aerospace engineering, electronics, semiconductors, flexible/wearable devices, and environmental technologies^12,13^. The global CNT market currently stands at approximately $12.5 billion, projected to exceed $35.8 billion by 2034 with a 12.4% compound annual growth rate^14^. Against this market backdrop, sustainable CNT synthesis has emerged as a critical industrial priority.

Chemical vapor deposition (CVD) dominates current CNT production due to its operational simplicity and technical maturity. This process synthesizes CNTs by catalytically decomposing volatile precursors such as methane, ethylene, acetylene, benzene, xylene, and toluene, which are produced from fossil fuels^15^. However, in the context of sustainable carbon materials development, substituting fossil-derived feedstocks and addressing energy-intensive requirements in CVD are critical for future low-carbon CNT production.

Waste plastics, characterized by low carbon-to-hydrogen ratios (0.5–1 for polymers like low-density polyethylene (LDPE), high-density polyethylene (HDPE), polypropylene (PP), polystyrene (PS), and polyvinyl chloride (PVC)), represent a significantly underutilized carbon resource, with annual global waste exceeding 370 million tons^13,16^. This underscores their substantial potential as sustainable carbon feedstocks for CNT production. Furthermore, H_2_ can be produced as another valuable chemical during plastic conversion.

As an alternative to CVD, catalytic decomposition enables one-step conversion of plastic waste at moderate temperatures^17,18^. Therefore, the catalytic synthesis of H_2_ and CNT from plastic wastes offers a promising alternative. This dual-output approach supports sustainable CNT production by valorizing plastic waste and reducing reliance on petrochemical precursors. However, to convert chlorine or fluorine-containing plastic is very challenging, in particular the durability of the catalyst.

In this work, a prompt strategy with a tandem catalyst structure was developed for upcycling chlorinated/fluorinated plastic waste mixtures into H_2_ and CNTs using an in-situ constructed FeNi/Ni/C catalyst under microwave promotion. The FeC-FeNi/Ni/C were used as a precursor and synthesized via a solvent-free, rapid, and energy-efficient microwave method. During upcycling, Cl/F released from PVC/PTFE regenerates the catalyst by reconstructing the active FeNi/Ni/C catalyst, enabling both catalytic lifespan (>35 cycles) and activity. Concurrently, fluoride species from PTFE decomposition can also cleave C-C bonds and expose fresh catalytic sites, amplifying efficiency. Ultimately, this well-designed process achieves a H_2_ yield of >922 mmol g^−^^1^_catalyst_ (purity of 87–98 vol%) and CNTs yields of >10,600 mg g^−1^_catalyst_, ca. 100% H_2_ and carbon efficiency after upcycling of complex plastic mixtures (LDPE-PVC or LDPE-HDPE-PP-PS-PVC-PTFE), while reducing energy consumption by 80% versus conventional thermal catalysis. The dual role of chlorinated/fluorinated plastic mixtures, as both feedstocks and in situ regenerants, enables long-term operation without supplementing additional fresh catalysts. This innovative strategy offers a promising and practical pathway for the treatment and valorization of chlorinated/fluorinated plastic waste mixtures.

## Results

### Investigation of reaction parameters

A special tandem design of the catalyst bed was made in this study, in which one part of the catalyst particles and plastic waste were mixed and placed in the reactor as the bottom layer, and the other part of the catalyst particles was placed on top of the bottom layer (Supplementary Fig. 1). To demonstrate the superiority and functionality of tandem design, a comparison experiment with only catalyst and plastic waste mixture was carried out. As shown in Supplementary Fig. 2, the tandem design not only reduces the ratio of byproducts C_2+_ and oil but also increases the purity of H_2_ in the gaseous phase. Ultimately, an H_2_ efficiency of 90% is achieved. This is considerably higher than the 68.3% obtained with the process of only mixing the catalyst with the plastic waste.

Catalyst composition, microwave irradiation power, and plastic-to-catalyst mass ratio critically influence plastic decomposition efficiency^18^. So these parameters were systematically optimized through a three-step protocol. First, Ni-Fe/C catalyst precursors with varying Ni/Fe ratios (Ni/C, Ni_19_Fe/C, Ni_9.5_Fe/C, Ni_6.3_Fe/C, Fe_3_C) were synthesized via a solvent-free, rapid, and energy-efficient microwave synthesis method. Following the catalytic decomposition of LDPE, H_2_ efficiency reaches 85.8%, 88%, 90%, 87% and 76% for the respective catalysts (Supplementary Fig. 3 and Supplementary Table 1). Based on these results, Ni_9.5_Fe/C precursor, which was in-situ converted to FeNi/Ni/C, which will be proved later, is the optimal catalyst and selected for further investigation.

Next, the effect of microwave irradiation power on H_2_ efficiency was evaluated. The H_2_ efficiency increases from 85.7% to 90% as the microwave power rises from 500 W to 600 W. However, at 700 W, the H_2_ efficiency decreases to 84% (Supplementary Fig. 2b, Supplementary Fig. 4 and Supplementary Table 2). The above results establish 600 W as optimal. Subsequently, plastic-to-catalyst mass ratios were optimized. As shown in Supplementary Fig. 5 and Supplementary Table 3, a 3g: 3g (plastic: catalyst) ratio in the bottom layer with an additional 3 g of catalyst placed in the top layer maximizes catalytic efficiency.

Based on the above results, the optimal reaction parameters are identified as follows: tandem catalysis configuration, Ni_9.5_Fe/C as the catalyst precursor, 600 W microwave power, a 1:1 mass ratio of plastic to catalyst (3 g each) in the bottom layer, and 3 g of catalyst in the top layer.

### Decomposition of LDPE or PVC

Prior to microwave catalysis, the mechanically pulverized LDPE plastic and the FeNi/Ni/C were uniformly mixed in a 3 g: 3 g mass ratio. Then, this mixture was placed in the bottom layer, with the remaining 3 g of catalyst placed in the top layer of the vessel. After that, the mixture was then processed under microwave irradiation in N_2_ atmosphere. To evaluate the long-term stability of the catalyst, 35 successive cycles of decomposition of two types of plastic mixture were carried out. Supplementary Fig. 6 illustrates the product distribution of the cycles 1-25 and cycles 31-35 for LDPE and cycles 26-30 for PVC over the optimized catalyst FeNi/Ni/C.

The FeNi/Ni/C catalyst exhibits robust performance during the first 18 cycles, converting more than 90 wt% of plastic into gas (weight ratio: 10–29 wt%) and solid carbon (weight ratio: 68–85 wt%) in each cycle (Supplementary Fig. 6a, Supplementary Table 4 and Supplementary Movie 1). H_2_ efficiency ranges from 77% to 98.2% with a purity of 85 to 98.4 vol% during the first 18 cycles, accompanying 80–100% carbon efficiency. This indicates that LDPE is almost converted into H_2_ and carbon products (Supplementary Fig. 6b-c).

However, between cycles 19 to 25, the efficiency and purity of H_2_ exhibit a progressive decline, dropping from 60% to 39.2% and 74 vol% to 52 vol%, respectively. This decline is attributed to significant carbon accumulation on the catalyst surface (e.g., cycle 25: 55,000 mg), which likely blocks active sites (Supplementary Fig. 6c). To enhance the catalyst stability, PVC, as the second type of plastic, was introduced in cycles 26–30, pyrolysis gas chromatography-mass spectrometry (Py-GC-MS) characterization was performed to investigate the product distribution of PVC decomposition. Results show that the percentage of HCl in the pyrolysis products accounts for approximately 18% (Supplementary Table 5), which can be absorbed by Ni and Fe in the catalyst to form nickel chloride and iron chloride. Subsequently, nickel and iron chloride are reduced by the produced hydrogen, a process that likely facilitates catalyst regeneration^19–21^.

The catalyst shows signs of regeneration in cycles 26–30, LDPE was then put in the reactor for cycles 31–35, the H_2_ efficiency rebounds from 39.2% to 85.4%, and the purity increased from 52 vol% to 89 vol% in cycle 31. This confirms the Cl^-^-induced regeneration of deactivated catalysts. However, activity declines again in cycles 32 to 35 as carbon accumulation reaches 58,350 mg by cycle 31. It suggests that Cl^-^ regeneration of the used catalyst may be necessary for each catalytic cycle (Supplementary Fig. 6b). This hypothesis can be further verified through subsequent experiments.

### Decomposition of LDPE and PVC mixtures

**Fig. 1:**
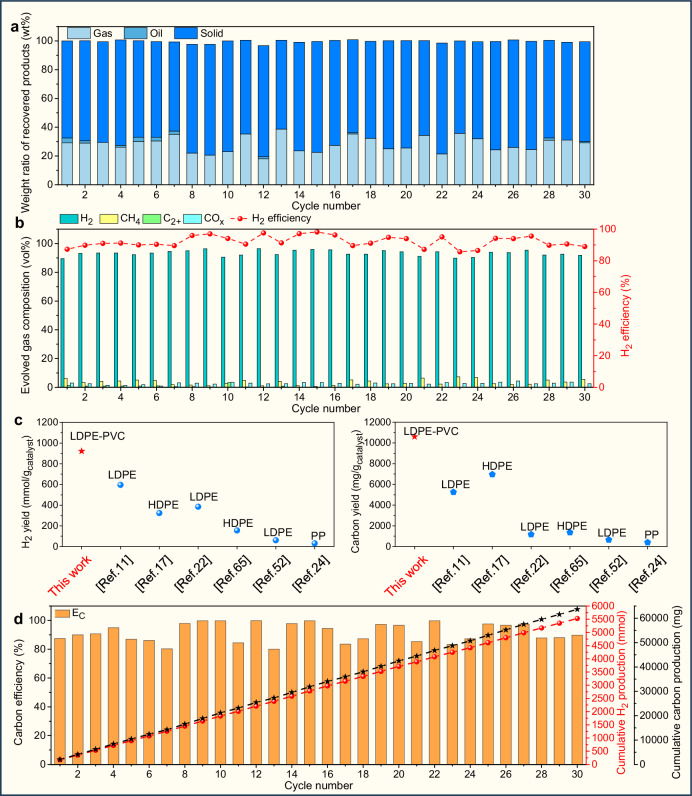
Successive cycles of microwave catalytic decomposition of plastic over FeNi/Ni/C catalyst. **a** The weight ratio of recovered gas, oil and solid. **b** Evolved gas composition (vol%) and H_2_ efficiency (%). **c** Summary of H_2_ yield (mmol g^−1^_catalyst_) and carbon yield (mg g^−1^_catalyst_) achieved in this work and other representative studies (Ti_3_AlC_2_ (Ref. ^11^), FeAlO_x_ (Ref. ^17^), Al_i_Fe_j_O_K_ (Ref. ^22^), Fe/Ni-CeO_2_@CNTs (Ref. ^65^), Fe_1_Co_1_Al_2_ (Ref. ^52^), stainless-steel mesh (Ref. ^24^). **d** Carbon efficiency (%) (E_C_), cumulative H_2_ production (mmol) and cumulative carbon production (mg). Reaction conditions: 3 g plastic and 3 g FeNi/Ni/C catalyst in the bottom layer; an additional 3 g FeNi/Ni/C catalyst in the top layer. Plastic: LDPE and PVC mixture (the mass ratio of LDPE to PVC is 9:1).

To further discover the potential of this catalyst and Cl^-^ regeneration process, microwave catalytic decomposition of an LDPE and PVC mixture (LDPE: PVC = 9:1) was conducted over 30 successive cycles (Supplementary Table 6). Notably, the catalyst exhibits robust performance. In each cycle, more than 95 wt% of the plastic mixture is efficiently converted into gas and solid carbon. H_2_ efficiency reaches 85.7%-98.2% with a purity of 89.4-97 vol%. Correspondingly, carbon efficiency reaches 83.3–99.9% (Fig. 1).

The remarkable performance of this catalyst is compared with that of representative benchmark studies, as shown in Fig. 1c. Specifically, FeNi/Ni/C catalyst achieves H_2_ and CNTs yields (922 mmol g^−1^_catalyst_ and 10,608 mg g^−1^_catalyst_) that are much higher than the reported benchmark catalysts although more stable plastic (PVC and LDPE) was used in this study than the previous ones (LDPE, HDPE, PP or PS) (Fig. 1c and Supplementary Table 7). Therefore, this FeNi/Ni/C catalyst enables highly efficient plastic upcycling to valuable H_2_ and CNTs^17,22–24^. Moreover, after 30 successive cycles, the cumulative amounts of H_2_ and carbon reach 5530 mmol and 63650 mg, respectively (Fig. 1d and Supplementary Fig. 7). These outcomes suggest that nearly all H atoms in the LDPE and PVC mixture are efficiently transformed into H_2_, while the carbon atoms are turned into CNTs, indicating that this approach has a nearly 100% atom economy. The superiority and functionality of this approach are further illustrated in Supplementary Figs. 8–10. Importantly, the approach can also maintain good catalytic performance even with PVC contents as low as 1% (LDPE: PVC = 99:1) over 30 successive cycles (Supplementary Figs. 11).

To further emphasize the advantages of microwave catalysis, a comparison between traditional thermal catalysis and microwave catalysis under suitable reaction conditions for the same conversion rate of plastic waste mixtures was made. Results show that traditional thermal catalysis operated at 600 °C obtains a lower H_2_ efficiency of 60%, which is 35% lower than that achieved by microwave catalysis at 450 °C. Consequently, the oil weight ratio increases to 42.9 wt%, indicating relatively poor catalytic efficiency (Supplementary Fig. 12a). This difference can be attributed to the “selective heating” effect of microwave catalysis, which creates a non-equilibrium field that preferentially activates catalytic active sites and minimizes side reactions^17,25–27^. In contrast, traditional heating methods rely on heat transfer across the heating medium and cannot achieve the same level of efficiency^17,25–27^. Additionally, energy consumption metrics further highlight microwave catalysis superiority: thermal catalysis at 600 °C requires 0.995 kWh versus 0.234 kWh for microwave catalysis at 450 °C under the condition of the similar conversion rate of the plastic waste mixture (2.7 g LDPE, 0.3 g PVC and 3 g FeNi/Ni/C in the bottom layer, with another 3 g FeNi/Ni/C in the top layer) (Supplementary Fig. 12b and Supplementary Figs. 13, 14). The fourfold energy reduction, coupled with lower operating temperatures (450 °C vs. 600 °C), positions microwave catalysis as a high-efficiency, low-carbon alternative for plastic upcycling.

Results presented above suggest that PVC can effectively enhance the cyclic lifespan and catalytic stability of FeNi/Ni/C catalysts, while microwave catalysis exhibits lower energy requirements and more favorable catalytic performance compared to traditional thermal catalysis.

### Carbonaceous products characterization

**Fig. 2:**
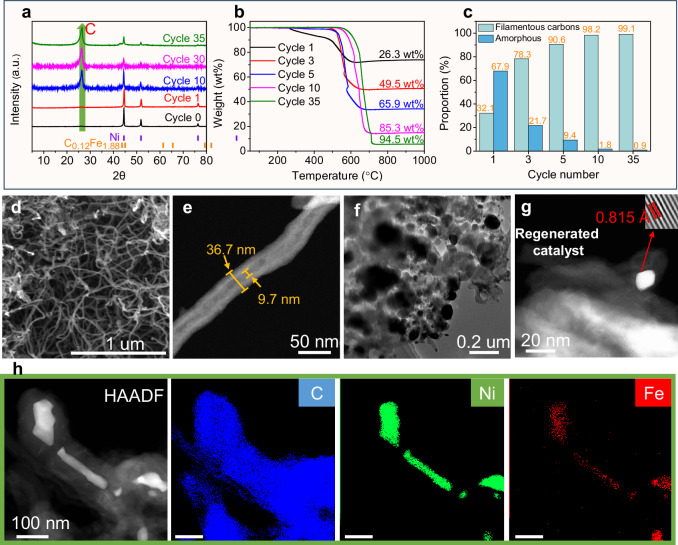
Characterization of the carbon product and catalysts. **a** XRD patterns. **b** TGA analysis. **c** Proportion of different carbon types in the carbon products. **d** SEM and **e** STEM images of the acquired carbon. **f** TEM image of FeC-FeNi/Ni/C. **g** STEM image of the regenerated FeNi/Ni/C catalyst and **h** corresponding element mapping (blue for C, green for Ni, and red for Fe).

Carbonaceous materials derived from microwave-catalyzed plastic decomposition were structurally and compositionally analyzed. X-ray diffraction (XRD) reveals a prominent graphitic carbon peak at approximately 26° and intensified signal with reaction cycles indicates progressive carbon accumulation (Fig. 2a). Thermogravimetric (TGA) quantified post-reaction carbon content, rising from 26.3 wt% (cycle 1) to 94.5 wt% (cycle 35), confirming cumulative carbon deposition (Fig. 2b). This accumulation is attributed to the selective cleavage of C-H bonds by the catalyst under microwave irradiation, resulting in the extraction of H_2_ from plastic and the growth of carbon materials with the assistance of the catalyst^17^.

Interestingly, the accumulated carbon materials undergo a dynamic structural evolution during cycling. The initial cycle (cycle 1) predominantly produces amorphous carbon, which has an oxidation temperature ~450 °C. It progressively transforms into more valuable CNT which have an oxidation temperature > 535 °C^28–30^ through a recrystallization process, improving the purity and value of CNTs^31,32^. By cycle 35, filamentous CNTs constitute 99.1% of carbon products, indicating their high commercial application potential (Fig. 2c). Raman spectroscopy corroborates increasing graphitization in the carbon products with successive cycles, which is consistent with the above results (Supplementary Fig. 15).

The structural and compositional details of the carbon products were further examined using scanning electron microscopy (SEM) and scanning transmission electron microscopy (STEM). SEM images reveal extensive filamentous carbon structures (Fig. 2d). STEM analysis confirms the filamentous carbon is multi-walled carbon nanotubes (MWCNTs) with inner and outer diameters of approximately 9.7 nm and 36.7 nm, respectively (Fig. 2e), indicating ca. 40 layers of CNT.

Additionally, the length of the produced CNTs was also measured by transmission electron microscope (TEM) and atomic force microscopy (AFM). Results indicate that CNTs from the first cycle exhibit a length distribution of 0.15-2.55 μm, with an average length of 0.48 μm (Supplementary Fig. 16a). For CNTs from 30 cycles, TEM measurements reveal a length distribution of 0.3-1.5 μm and an average length of 0.67 μm, higher than that of first-cycle CNTs (Supplementary Fig. 16b). Similarly, AFM characterization confirms a length distribution of 0.44–1.44 μm, which is largely consistent with TEM results (Supplementary Fig. 16c).

The lithium-ion storage and electromagnetic wave absorption performance of CNTs produced after 35 cycles was evaluated. The results reveal a specific capacity of approximately 200-300 mAh g^-1^ over 200 cycles, which demonstrates excellent Li-ion intercalation and deintercalation capabilities^33,34^ (Supplementary Fig. 17). Electromagnetic wave absorption analysis shows minimum reflection loss of −39.6 dB and an absorption bandwidth of 3.9 GHz (11.3–15.2 GHz) (Supplementary Fig. 18). These results indicate the potential of these CNTs produced by microwave-catalyzed plastic decomposition as high-performance lithium-ion storage and electromagnetic wave absorption materials^35–37^. Collectively, the carbonaceous product derived from FeNi/Ni/C-catalyzed plastic decomposition consists of multi-walled carbon nanotubes, which exhibit promising application prospects for lithium battery energy storage and electromagnetic wave absorption.

### Mechanism of catalyst regeneration by Cl^-^

The results above demonstrate that the designed process can efficiently convert plastics into blue H_2_ and high-value CNTs. However, the reason for the long-term stability of catalysts, especially when PVC is used as a co-feedstock, requires further investigation. Therefore, detailed analysis including X-ray absorption spectroscopy (XAS), XRD, TEM, and STEM was carried out to monitor the structure of the FeC-FeNi/Ni/C (precursor) and the in-situ formed FeNi/Ni/C catalysts.

The FeC-FeNi/Ni/C structure was initially investigated using X-ray photoelectron spectroscopy (XPS) (Supplementary Fig. 19). Ni 2p XPS spectra reveal peaks at around 852.6 eV (Ni^0^ 2p_3/2_), 854.9 eV (Ni^2+^ 2p_3/2_) and 869.6 eV (Ni^0^ 2p_1/2_)^38,39^, while Fe 2p spectrum show signatures at around 705.4 eV (Fe^0^ 2p_3/2_) and 711.5 eV (Fe^3+^ 2p_3/2_)^40^, confirming the successful synthesis of the FeC-FeNi/Ni/C. Subsequently, XRD analysis was employed for further structural characterization (Supplementary Fig. 20). Diffraction peaks corresponding to Ni metal (PDF no. 04-0850) and C_0.12_Fe_1.88_ (PDF no. 44-1293) are identified. The relatively low intensity of the Fe diffraction peaks is likely due to the high dispersion of Fe^41^. X-ray fluorescence spectroscopy (XRF) verifies a Ni/Fe molar ratio of 9.5, consistent with design parameters (Supplementary Fig. 21). TGA reveals that the FeC-FeNi/Ni/C contains 2.6 wt% carbon, indicating that Ni and Fe dominance in the catalyst bulk (Supplementary Fig. 22).

For microscopic topography characterization, TEM and high-angle annular dark-field scanning transmission electron microscopy (HAADF-STEM) equipped with energy-dispersive X-ray spectroscopy (EDX) were employed. The FeC-FeNi/Ni/C exhibit a spherical morphology, with an average size of 19.8 nm (Fig. 2f and Supplementary Fig. 23). EDX shows that Ni and Fe atoms are not fully alloyed in FeC-FeNi/Ni/C (Supplementary Fig. 24). Importantly, after the 15th cycle (Feedstock: LDPE and PVC mixtures), Ni and Fe form an alloy with lattice fringes corresponding to the FeNi_3_ (331) plane (d-spacing: 0.815 Å) (Fig. 2g, h and Supplementary Fig. 25).

**Fig. 3:**
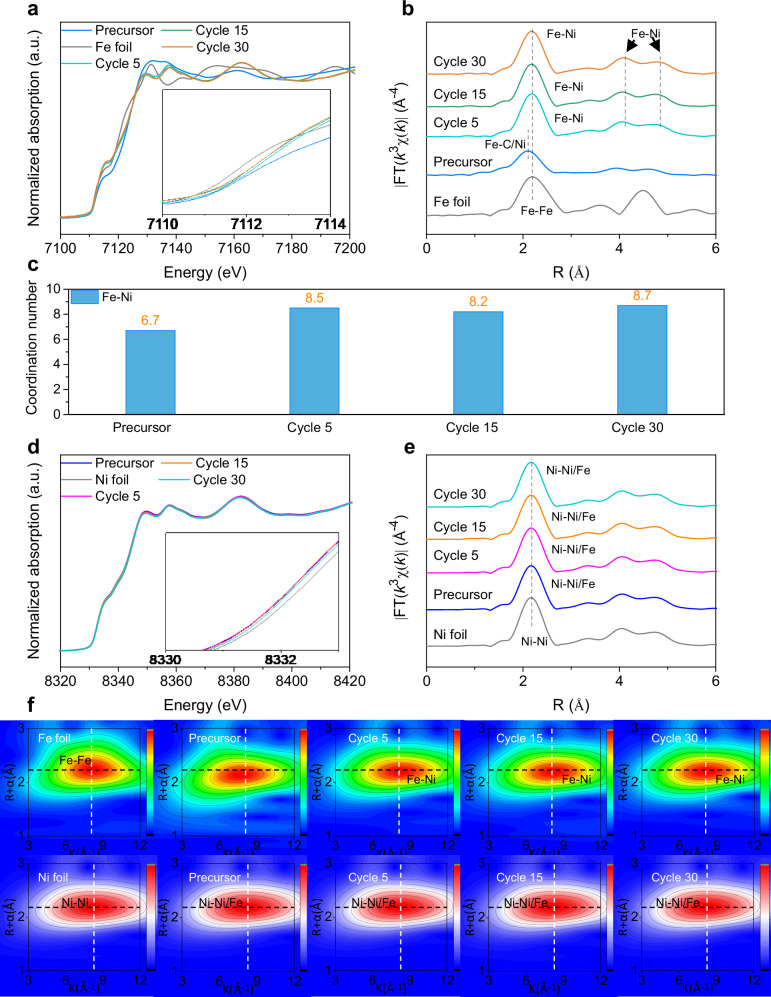
XAS analysis. **a** Fe K-edge XANES spectra of FeC-FeNi/Ni/C (precursor) and FeNi/Ni/C (regenerated) catalysts as well as references. **b** Fourier transforms (FT) of Fe EXAFS spectra of FeC-FeNi/Ni/C and FeNi/Ni/C catalysts and references. **c** Changes of coordination number in FeC-FeNi/Ni/C and FeNi/Ni/C catalysts. **d** Ni K-edge XANES spectra of FeC-FeNi/Ni/C and FeNi/Ni/C catalysts as well as references. **e** Fourier transforms (FT) of EXAFS spectra (Ni) of FeC-FeNi/Ni/C and FeNi/Ni/C catalysts and references. **f** WT-EXAFS of Fe and Ni.

To further reveal the structure change from the catalyst precursor (FeC-FeNi/Ni/C) to the in-situ formed catalyst FeNi/Ni/C, the local structure of the precursor and the catalyst was investigated by XAS. As shown in Fig. 3, Supplementary Figs. 26, 27 and Supplementary Table 8, the Fe K-edge XAS spectrum of the precursor FeC-FeNi/Ni/C exhibits a significant shift to higher energy compared to the Fe foil, indicating Fe in a higher oxidation state^42,43^. Importantly, the Fe K-edge of the regenerated catalyst (Cycle 5, Cycle 15 and Cycle 30 with a feedstock ratio of LDPE to PVC of 9:1) shifts to a lower energy, approaching that of Fe foil. This suggests a reduction of Fe in the precursor to a state close to Fe foil^44^ (Fig. 3a and Supplementary Table 8). This is because in-situ reconstruction occurs under the action of the Cl^-^ released by PVC during the microwave catalytic decomposition of the LDPE-PVC mixture process. This process leads to the formation of FeNi alloy compounds^45^, facilitating the change of the precursor to a highly efficient FeNi/Ni/C catalyst. Extended X-ray absorption fine structure (EXAFS) analysis reveals dominant Fe-Ni coordination at 2.2 Å in FeNi/Ni/C versus Fe-C/Ni bonding at around 2.1 Å in the precursor FeC-FeNi/Ni/C. Enhanced Ni-Fe scattering^46^ singles at ~4.0 Å and ~4.7 Å in FeNi/Ni/C further validate structural evolution (Fig. 3b).

The coordination numbers of Fe-Ni in FeC-FeNi/Ni/C and FeNi/Ni/C also support the above findings. In FeC-FeNi/Ni/C, the Fe-Ni coordination number is 6.7. In the regenerated FeNi/Ni/C, it increases to 8.5 (Cycle 5), 8.2 (Cycle 15), and 8.7 (Cycle 30), respectively (Fig. 3c). Ni K-edge shows metallic nature in both FeC-FeNi/Ni/C and FeNi/Ni/C, with energies slightly below Ni foil. Additionally, detailed analysis of the Fe K-edge energy (E_0_) (the insert of Fig. 3a) shows a slight positive shift from 7112.8 eV (Fe foil reference) to 7113.1 eV in the regenerated catalyst, suggesting minor electron loss and a slightly higher oxidation state relative to metallic iron. Similarly, analysis of the Ni K-edge energy (the insert of Fig. 3d) reveals a minor negative shift from 8332.6 eV (Ni foil reference) to 8332.4 eV in the regenerated catalyst, implying a small gain in electrons and a slightly lower oxidation state compared with metallic nickel. This indicates there is a small electron transfer between Ni and Fe^45,47^. The Ni-Fe coordination number also increases from 6.5 in FeC-FeNi/Ni/C to 6.8 (Cycle 5), 6.6 (Cycle 15), and 6.8 (Cycle 30) in FeNi/Ni/C. The Ni-Fe coordination number has minimal change due to the higher Ni content compared to Fe (Supplementary Fig. 21). Furthermore, a peak at approximately 2.2 Å corresponds to Ni-Ni/Fe coordination (Fig. 3e).

Wavelet transform (WT) was employed to further analyze Fe and Ni K-edge EXAFS oscillations. The WT contour plot of Fe foil shows a single intensity maximum at ~8.1 Å^-1^ (Fig. 3f), corresponding to Fe-Fe coordination. For the FeC-FeNi/Ni/C precursor, the WT maximum appears at 7.8 Å^-1^. In contrast, the WT maxima of regenerated FeNi/Ni/C (Cycles 5, 15, and 30) shift to ~8.2 Å^-1^, consistent with Ni-Fe coordination. The above results indicate that iron does not form good coordination with nickel in the precursor, but in the regenerated catalyst. For Ni K-edge EXAFS oscillations, the WT maxima of Ni foil, FeC-FeNi/Ni/C precursor, and regenerated FeNi/Ni/C (Cycles 5, 15, and 30) all appear at ~8.3 Å^-1^, attributed to Ni-Ni or Ni-Ni/Fe coordination. This is due to the higher Ni content relative to Fe and the metallic nature of Ni. Collectively, the WT results are consistent with the aforementioned results (Fig. 3a–e).

Additional supporting evidence for the catalyst regeneration was also investigated. Specifically, the reconstruction of FeNi alloy in the PVC-assisted FeC-FeNi/Ni/C catalyst follows the procedure: hydrogen generated during plastic decomposition reduces the catalyst to produce water molecules, which combine with hydrogen chloride from PVC decomposition to form hydrochloric acid molecules. These hydrochloric acid molecules thereby effectively convert Ni and Fe into nickel chloride and iron chloride. Subsequently, the formed nickel chloride and iron chloride are reduced by hydrogen, thereby facilitating the formation of FeNi alloy, as discussed in detail in Supplementary Materials (Supplementary Figs. 28–32).

The specific surface area of the regenerated catalyst also increases, which supports better dispersion of catalyst particles (Supplementary Fig. 33 and Supplementary Table 9). These findings demonstrate that the addition of PVC promotes the regeneration of the catalyst into a stable FeNi alloy, and significantly extends the catalytic stability, as shown in Fig. 1. Therefore, chlorine derived from PVC plastics reacts with Fe and Ni species in FeC-FeNi/Ni/C catalyst precursors to form ferric chloride and nickel chloride. These compounds are subsequently reduced to FeNi alloys by hydrogen generated during plastic decomposition, yielding stable FeNi/Ni/C catalysts that notably enhance catalytic stability and activity.

### Decomposition of chlorinated/fluorinated plastic waste mixtures

As shown above, the in-situ constructed FeNi/Ni/C catalyst demonstrates the transformative potential for real-world plastic decomposition. Real-world plastic waste typically comprises heterogeneous mixtures of LDPE, HDPE, PP, PS, PVC, and PTFE in variable ratios^6^. Conventional separation processes for individual plastic streams remain economically prohibitive^48,49^. To validate broad applicability, plastic mixtures of LDPE, HDPE, PP, PS, PVC, and PTFE waste in different mass ratios (including 15:7:2:5:7:5, 40:30:40:20:5:1, 2:6:4:6:4:1, 10:10:3:10:5:5, 5:10:3:15:10:3, and 5:2:15:5:5:1) were tested. Notably, polyester plastic waste was not included in this study as it can be more efficiently and easily converted into suitable monomer products^4,6^.

**Fig. 4:**
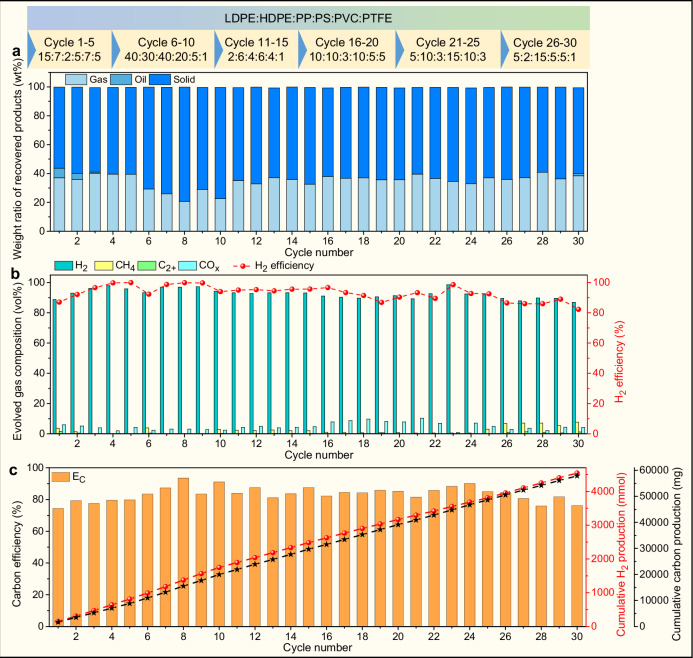
Successive cycles of microwave catalytic decomposition of LDPE, HDPE, PP, PS, PVC, and PTFE plastic mixtures with different ratios over FeNi/Ni/C catalyst. **a** The weight ratio of recovered gas, oil, and solid. **b** Corresponding evolved gas composition (vol%) and H_2_ efficiency (%). **c** Carbon efficiency (%) (E_C_), cumulative H_2_ production (mmol), and cumulative carbon production (mg) (dotted lines). Reaction conditions: 3 g mixture of LDPE, HDPE, PP, PS, PVC, and PTFE plastic and 3 g FeNi/Ni/C catalyst in the bottom layer; 3 g FeNi/Ni/C catalyst in the top layer.

As shown in Fig. 4, in 30 successive catalytic decomposition cycles, LDPE, HDPE, PP, PS, PVC, and PTFE plastic mixtures are efficiently converted into gas (weight ratio: 20-47 wt%) and solid carbon (weight ratio: 50–80 wt%) products after 10 min reaction (Fig. 4a and Supplementary Table 10). Importantly, H_2_ remains the dominant component in the gas phase, with efficiency and purity ranging from 83%-99.9% and 86 vol%-98 vol%, respectively (Fig. 4b). Furthermore, carbon efficiency (75-92%) remains stable across diverse plastic mixture compositions, demonstrating robust upcycling performance (Fig. 4c). Importantly, apart from LDPE, HDPE, PP, PS, PVC and PTFE plastic mixtures, more complex landfilled plastic mixture can also be steadily upcycled, as discussed in Supplementary Materials (Supplementary Figs. 34–36 and Supplementary Tables 11, 12). Moreover, for the functionality of fluorine, which may enhance the long-term stability of catalysts through two mechanisms: reconstructing FeNi active sites and shearing CNTs to expose additional catalytic sites, as discussed in Supplementary Materials (Supplementary Figs. 37, 38).

XRF analysis indicates that the Cl content is 0.17% after 30 cycles. No F is detected in the catalyst, and no Cl and F accumulation is observed, indicating that F left the reaction system and does not remain in the catalyst. Additionally, over 99% of Cl and 99.4% of F released from the reaction system were captured by a 1 M NaOH solution, thus preventing their emission into the environment (Supplementary Fig. 39).

Additionally, the decomposition reaction can proceed efficiently under mild conditions with a low catalyst loading (Plastic mixture: Catalyst = 10: 1, Supplementary Fig. 40). Moreover, the Cl^-^/F^-^ byproducts can also be captured via aqueous absorption, generating acidic solutions that enable CNT purification without the use of commercial acids, thereby reducing the impurity content in CNT, as discussed in Supplementary Figs. 41–63. This is a significant advancement because the purification of CNTs can be achieved without the use of commercial acids, bringing substantial economic and environmental benefits. Overall, FeNi/Ni/C catalysts demonstrate robust catalytic stability and facilitate the efficient conversion of multi-component plastic mixtures, including LDPE, HDPE, PP, PS, PVC, and PTFE, and landfilled plastic blends to hydrogen and carbon nanotubes.

### Microwave catalysis mechanism

The potential reaction mechanism of microwave-catalyzed plastic decomposition to H_2_ and CNTs was investigated. While CNTs produced during the reaction are well-known excellent microwave absorber materials, previous work proved that such materials have very weak activity during the plastic decomposition process to produce H_2_ and CNTs^17^. Based on these results, density functional theory (DFT) calculations were thus performed to elucidate the roles of Fe and Ni catalytic sites in driving H_2_ evolution and CNT growth.

**Fig. 5:**
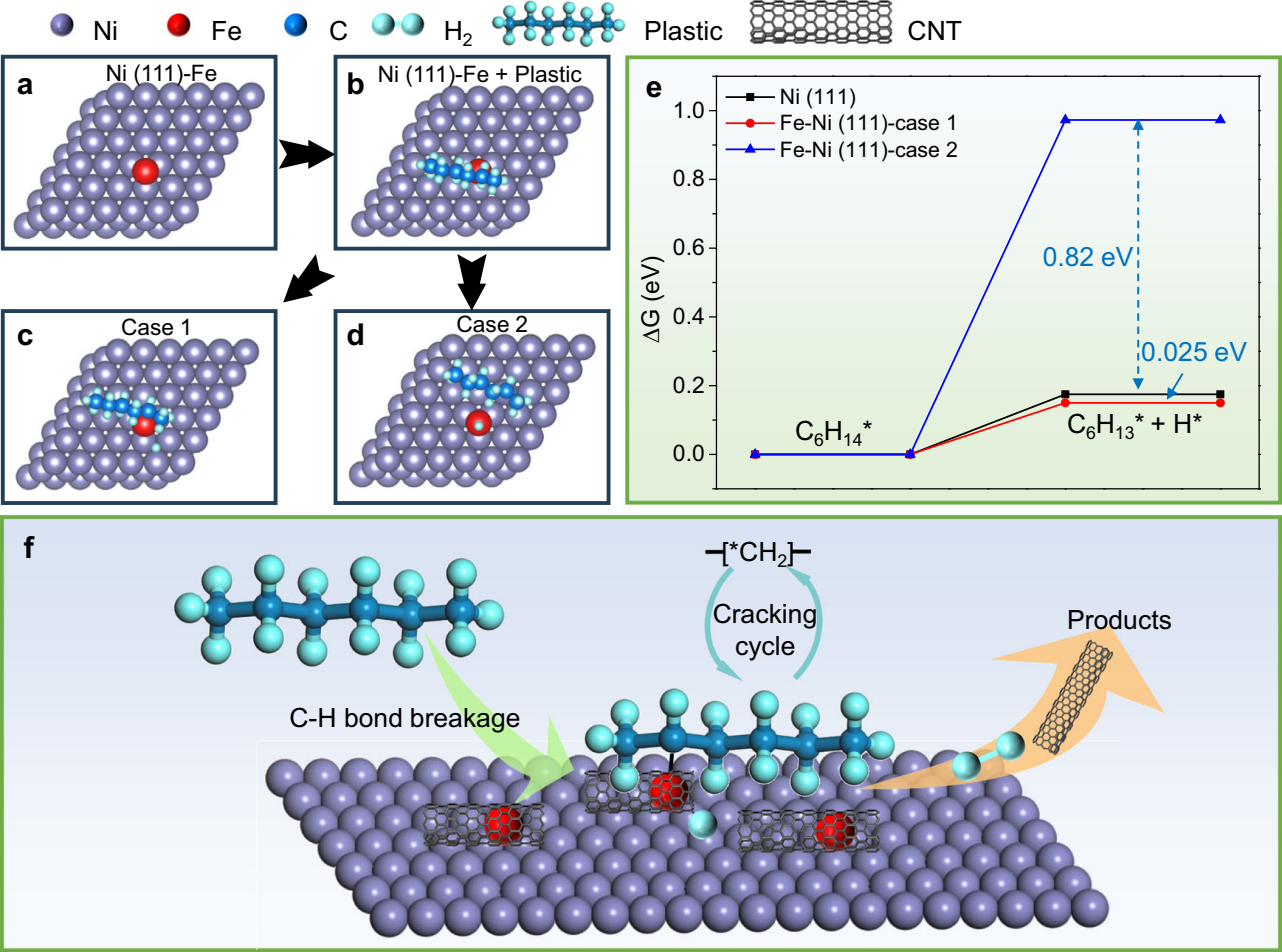
Proposed mechanism. The model of **a** Ni-Fe, **b** Ni-Fe + plastic, **c** case1 and **d** case 2. **e** The calculated free-energy changes for C-H bond breaking reaction of C_6_H_14_ on Ni (111), Fe-Ni (111)-case 1, and Fe-Ni (111)-case 2. **f** An illustration of microwave catalytic upcycling of plastic over FeNi/Ni/C catalyst.

Density functional theory (DFT) calculations were performed using the Vienna Ab Initio Simulation Package (VASP) with the generalized gradient approximation (GGA) and Perdew-Burke-Ernzerhof (rPBE) functional. Models simulating n-hexane^8,50^ decomposition over Ni/C and FeNi/Ni/C catalysts were constructed, focusing on the C-H bond scission process. For the Ni/C catalyst, n-hexane initially adsorbs onto the catalyst’s surface, where Ni atoms mediate sequential C-H bond cleavage. Initial C-H bond cleavage generates H^*^ and C_6_H_13_^*^ intermediates bound to Ni sites, followed by further decomposition to H_2_ and carbon with a free energy barrier (ΔG) of 0.175 eV (Fig. 5 and Supplementary Fig. 64).

The FeNi/Ni/C catalyst, featuring dual Ni and Fe active sites, enables two distinct intermediate adsorption configurations post C-H bond cleavage. In Case 1, H^*^ binds to Ni while C_6_H_13_ anchors to Fe, exhibiting a free energy barrier (ΔG) of 0.15 eV. Case 2 reverses this binding (H^*^ on Fe, C_6_H_13_ on Ni), yielding a significantly higher ΔG of 0.973 eV (Fig. 5a-e and Supplementary Fig. 65). The lower ΔG in Case 1 establishes it as the dominant pathway, driving efficient decomposition through Ni-Fe synergy. H atoms evolve as gaseous H_2_, while carbon deposits on Fe crystallize into cylindrical networks, ultimately growing into high-purity CNTs (Fig. 5f)^17^. According to XAS results, there is an enhanced electron cloud density of Fe and Ni, along with electron transfer from Fe to Ni. Increased electron cloud density of Fe and Ni can improve the adsorption of the reaction substrate and the activation of C-H bonds, driving hydrocarbon conversion to H^*^ and C_X_H_Y_^*^, and continuous C-H cleavage yields hydrogen and carbon tubes^51,52^. Compared to Ni, Fe exhibits superior carbon-adsorbing properties; enhanced Fe electron cloud density also likely strengthens its bonding with C atoms, promoting carbon diffusion and crystallization into long carbon nanotubes^53,54^. These results are consistent with the DFT calculation.

The synergistic catalytic effect of Ni-Fe in FeNi/Ni/C catalysts can be further validated by experiments. For the Ni/C catalyst, LDPE decomposition achieves 85.8% H_2_ efficiency and 89.9% carbon efficiency (Supplementary Fig. 66 and Supplementary Table 13). In contrast, FeNi/Ni/C significantly enhance these metrics to 98.2% and 99.7%, respectively, which support the DFT-calculated results (Supplementary Fig. 6). In brief, underlying mechanistic studies suggest that nickel facilitates C–H bond cleavage during plastic decomposition, whereas iron promotes carbon nanotube nucleation and growth; these complementary effects enable the efficient conversion of plastic to hydrogen and carbon nanotubes via synergistic catalytic interactions.

### Techno-economic analysis

**Fig. 6:**
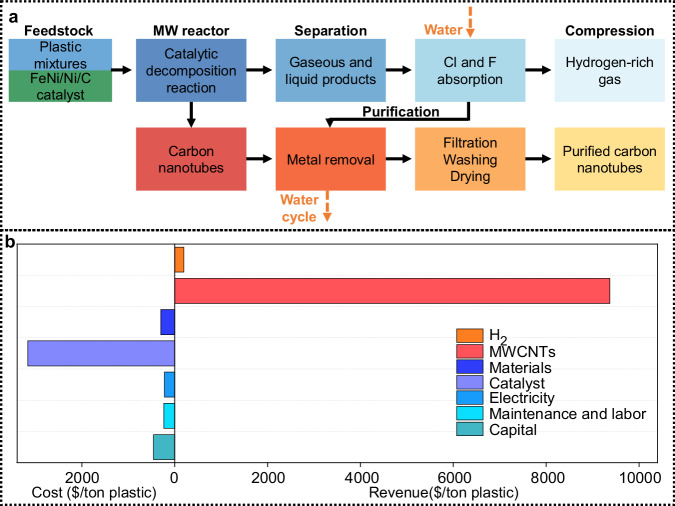
Techno-economic analysis. **a** Microwave catalytic decomposition of plastic mixtures and product posttreatment processes. **b** Techno-economic analysis of microwave-catalyzed decomposition of plastic mixtures.

To evaluate the economic feasibility of implementing a microwave catalytic decomposition process for plastic mixtures upcycling, a comprehensive techno-economic analysis (TEA) was conducted^55–57^ (Fig. 6 and Supplementary Tables 14, 15).

Figure 6a depicts the microwave-catalyzed decomposition of plastic mixtures into H_2_ and CNTs. This process was based on laboratory-scale findings and linearly scaled to process 1 tonne of plastic waste per hour. Specifically, the lifespan of the FeNi/Ni/C catalyst has been deemed as 35 successive cycles (i.e., 17.5 ton_plastic_ ton^-1^_catalyst_), but not limited to 35 cycles. H_2_ and CNT yields per tonne of plastic mixtures were calculated according to the average results from Fig. 4.

TEA identifies catalyst costs as the main economic factor (70% of total expenses), primarily driven by catalyst material expenses and consumption rates. However, this assessment assumes a conservative plastic-to-catalyst mass ratio of 1:2. Scaling to higher ratios (e.g., 10:1), would significantly reduce costs and make the process more economically feasible (Supplementary Fig. 40). Additionally, about 20% of the total cost comes from the procurement of plastic mixtures and capital investments. In contrast, costs for electricity, maintenance, and labor are relatively low. This is due to the energy-efficient and fast processing abilities of microwave catalysis, and the lack of need for commercial acid purification processes (Fig. 6a, Supplementary Figs. 12, 13 and Supplementary Figs. 41, 63).

Regarding product value, H_2_ ($ 1900 per tonne) and CNTs ($14,800 per tonne) both have substantial commercial potential^24,58^, with CNTs demonstrating superior commercial competitiveness. Even at 50% discount pricing, this microwave catalytic decomposition process retains robust market viability, demonstrating its potential economic feasibility (Fig. 6b). Furthermore, this microwave catalytic strategy outperforms existing approaches in upcycling chlorinated plastic waste, exhibiting superior feedstock adaptability across diverse plastic compositions (e.g., LDPE-PVC mixture, LDPE-HDPE-PP-PS-PVC-PTFE mixture and more complicated landfilled plastic mixture) while delivering nearly ten times higher product economic value (Supplementary Fig. 67 and Supplementary Table 16). Thus, this approach has substantial economic and social benefits, which support its wider application.

## Discussion

This work presents an in-situ constructed FeNi/Ni/C catalyst, which demonstrates exceptional efficiency in the selective upcycling of diverse chlorinated/fluorinated plastic waste mixtures (e.g., LDPE-PVC mixture, LDPE-HDPE-PP-PS-PVC-PTFE mixture, and more complicated landfilled plastic mixture) into valuable H_2_ and CNTs under microwave irradiation. This process achieves near-quantitative atom economy (both H_2_ and carbon efficiencies are nearly 100%), with H_2_ and CNTs yields reaching >922 mmol g^-1^_catalyst_ and >10,600 mg g^−^^1^_catalyst_, respectively. Notably, this microwave catalysis strategy exhibits unprecedented stability (>35 cycles) and energy efficiency (80% reduction vs. traditional thermal catalysis), alongside an unprecedented turnover number (107.6 mol _carbon_ mol^-1^_NiFe_).

Fundamental studies indicate that PVC-derived Cl^-^ and PTFE-derived F^-^ dynamically regenerate active sites, suppressing catalyst passivation while enhancing long-term stability, in which Ni is more likely to facilitate C-H breaking while Fe promotes CNT growth, together with CNT as the major microwave absorber. Techno-economic analysis indicates that this approach may have potential commercial viability and market competitiveness. These advances meet the critical challenge of upcycling hard-to-handle chlorinated/fluorinated plastic waste mixtures, indicating microwave catalysis as a significant advance for the high-value-added utilization of plastic.

## Methods

### Materials

Iron acetylacetonate (Fe(C_5_H_7_O_2_)_3_, ≥98%), and nickel acetate tetrahydrate (Ni(CH_3_COO)_2_·4H_2_O, ≥99%) were purchased from Aladdin Chemical Reagent Co., Ltd (Shanghai, China). Silicon carbide (SiC, 12 mesh) was purchased from Qing He Zhong Zhou Alloy Materials Co., Ltd (Hebei, China). All chemicals received are used without further purification. The LDPE was provided by the SINOPEC Tianjin Company. PVC and PTFE were purchased from Dongguan Zhangmutou Special Plastic Lang Chemical Raw Material Management Department. Plastic waste such as LDPE film, HDPE bottles, PP boxes, and PS foam were collected from the environment. Mixed plastic wastes were collected from landfilled sites in the UK.

### Preparation of catalysts

Catalysts were prepared by a solvent-free, fast, and energy-efficient microwave synthesis method.

First, the monometallic Ni/C catalyst was prepared by the following procedure: a mixture of Ni(CH_3_COO)_2_·4H_2_O and SiC (12 mesh) with a mass ratio of 3:1 (e.g., 30 g: 10 g) was placed in a quartz reaction flask, then treated at 400 W of microwave irradiation power under N_2_ atmosphere for 20 min. After reaction, the mixture was then sieved through a 100-mesh sieve to obtain the Ni/C catalyst as the SiC with a larger size left upon the sieve. Therefore, the Ni/C catalyst can be separated directly from the SiC. The SiC was collected and reused. For the synthesis of Fe_3_C, only the raw materials were replaced with Fe(C_5_H_7_O_2_)_3_, with other procedures remaining unchanged.

Second, the catalyst precursor FeC-FeNi/Ni/C was prepared by the following procedure: in a typical preparation process, 10 g of the above-synthesized Ni/C catalyst and 6.35 g iron acetylacetonate (Fe(C_5_H_7_O_2_)_3_) were mixed and placed in a quartz reaction flask, then treated at 400 W of microwave irradiation power under N_2_ atmosphere for 10 min to acquire the FeC-FeNi/Ni/C catalyst precursor. Catalysts with varying Ni/Fe molar ratios (19:1, 9.5:1, 6.3:1) were also synthesized using the above procedure by only adjusting the raw material ratio. It is worth noting that there is no need to add SiC as a microwave absorption medium to facilitate the synthesis of FeC-FeNi/Ni/C catalyst, as the above-synthesized Ni/C catalyst with excellent microwave absorption properties can quickly convert the incident electromagnetic wave energy into heat to realize the synthesis of FeC-FeNi/Ni/C catalyst.

### Characterizations

The crystallinity of the catalyst samples and carbon materials was characterized by X-ray diffraction (XRD) on a Bruker D8-Advance diffractometer using Cu Kɑ (*λ* = 1.54056 Å). The morphology of the catalyst samples and carbon materials was examined by QUANTA FEG 250 field emission scanning electron microscope (SEM) and high-resolution transmission electron microscopy (HRTEM) by JEM 2100 microscope, operated at an accelerating voltage of 200 kV. High-angle annular dark-field scanning TEM (HAADF-STEM) images were captured by an Aberration-corrected transmission electron microscope (JEM-ARM300F) operated at an accelerating voltage of 300 kV. X-ray absorption near-edge spectra (XANES) and extended X-ray absorption fine structure spectra (EXAFS) measurements of Ni and Fe were performed at the beamline BL17B of the Shanghai Synchrotron Radiation Facility (SSRF), Shanghai Advanced Research Institute, Chinese Academy of Sciences (CAS) in a transmission mode. The resulting carbon materials were detected by inVia-Qontor Raman spectroscopy with a 532 nm laser. The composition of the gas product was analyzed by gas chromatography (GC-7820). Permanent gases were detected by thermal conductivity detectors (TCD) with a 5 Å molecular sieve column, and hydrocarbons were detected by a flame-ionized detector (FID) with an HP-PLOT/Q column. Thermogravimetric analysis (TGA) was used to characterize the thermal stability of carbon compounds after the reaction and the degradation temperature of the plastic. Approximately 10 mg sample was heated from room temperature to 1000 °C with a heating rate of 10 °C min^–1^ under an air atmosphere. The elemental valence state information of catalysts’ surfaces was obtained by X-ray photoelectron spectroscopy (XPS ThermoFisher ESCALAB 250Xi). Electromagnetic adsorption was evaluated using a vector network analyzer (MS4642A) at the frequency of 2-18 GHz. The obtained carbon nanomaterial sample was mixed with paraffin with a weight percentage of the sample of 25%. Then, put the mixture into a coaxial ring with an inner diameter, outer diameter, and thickness of 5 mm, 7 mm, and 2 mm, respectively. The electrochemical properties of the acquired carbon materials after plastic decomposition were measured by a NEWARE battery testing system with a LiPF_6_/EC:DMC = 1: 1 as electrolytes. It is also worth noting that the materials used to test the lithium-ion storage and electromagnetic wave absorption properties were obtained after plastic catalytic decomposition, without undergoing any pretreatment or separation process before the test. To clarify the distribution of the product during PVC and PFTE decomposition, the pyrolysis products were analyzed by pyrolysis gas chromatography-mass spectrometry (Thermo Fisher-Exactive GC). Elemental composition was also measured using X-ray fluorescence on an AXIOS max. Thermogravimetric-mass spectrometry (TG-MS) analysis was performed on plastic catalytic cracking, with heating from room temperature to 600 °C at a rate of 10 °C min^-1^, and compounds with m/z 1-300 were detected.

### DFT calculation

Employing the Vienna Ab initio Simulation Package (VASP 6.3.2) to perform all density functional theory (DFT) calculations within the generalized gradient approximation (GGA) using the Perdew-Burke-Ernzerhof (rPBE) functional^59^. The projected augmented wave (PAW) potential was chosen to describe the ionic cores during the calculations, and the basis set of plane waves with a kinetic energy cut-off of 450 eV was used to account for the valence electrons. Geometrically optimized with a force convergence of less than 0.05 eV/Å with an energy convergence of 1 × 10^−^^5 ^eV. The original bulk structure of Ni was optimized before constructing the surface of the Monkhorst-Pack k point 8×8×8. The Ni (111) surface was constructed by cutting optimized bulk Ni into a four-layer slab. During the optimization of the overall adsorption structure, the top two layers of Ni were completely relaxed, and the other layers were immobilized. The supercell of Ni was 3 × 3 × 1 in the a-, b-, c- directions, and the vacuum space was 15 Å to avoid the interaction between slabs, α = β = 90°, γ = 120°, a = b = 14.726 Å, c = 21.012 Å. The Fe-Ni (111) model was constructed by putting the Fe atom on the Ni (111) surface and the spin polarization effect was also considered. The initial magnetic moment of the Ni atoms was set to +2 μB, and the initial magnetic moment of the Fe atoms was set to +5 μB^60,61^.

### Microwave catalytic decomposition of plastic

The mechanically pulverized plastic (3 g) was physically homogeneously mixed with catalyst particles (3 g) before the reaction. The mixture was then transferred into a quartz bottle and put in the bottom layer. Meanwhile, another 3 g catalyst particles were placed in the top layer as a tandem catalytic layer. After that, the quartz reactor was placed in the center of the microwave reactor, which has a microwave cavity of a 2.45 GHz magnetron, a multimode, and a maximum output power of 800 W (Qingdao Microwave Applied Technology Co., Ltd). As involved in previous works^25,26,62^, the IR thermometer is a widely used and relatively accurate method in the microwave field, which monitors the average temperature of the reaction bed in equilibrium. Therefore, the temperature in the mixture sample was measured using an infrared probe, and the maximum temperatures of the top layer and bottom layer were 450 °C and 415 °C, respectively. Since the top layer contains only the catalyst, microwave-induced heat is concentrated there, whereas heat from the catalyst in the bottom layer is transferred to the plastic, with partial energy consumption during plastic catalytic conversion, resulting in slightly lower temperatures in the bottom layer. Before microwave irradiation, N_2_ flow was applied for 10 min at a rate of 100 ml min^–1^ to purge oxygen from the reaction system. Then the catalytic plastic decomposition was performed at a microwave power of 600 W for 10-15 min. Two-stage cold traps (0 °C) were employed to collect oil products, and non-condensable products were collected in an airbag. 50 ml 1 M NaOH or H_2_O was used to absorb the released Cl and F compounds. Moreover, after each catalytic cycle, the catalyst and solid carbon mixture were taken out (without any treatment) and further mixed with fresh plastic for the next catalytic decomposition. It should be noted that if the carbon nanotubes increase too rapidly in the top or bottom layer, typically within the first three catalytic cycles, the top and bottom layer catalysts will need to be mixed. This mixture should then be evenly divided into two parts and applied in the subsequent >27 successive cycle experiments. This process helps prevent uneven distribution of microwave energy, since the resulting carbon nanotubes have better microwave absorption performance.

For comparison, the traditional thermal catalytic decomposition of plastic was carried out in an electric furnace. Reaction conditions: 3 g of catalyst and 3 g of plastic were put in the bottom layer; another 3 g of catalyst was placed in the top layer. The temperature set for the thermal catalysis was 600 °C with a heating rate of 10 °C min^–1^ under N_2_ atmosphere and kept at 600 °C for 10 min.

The volume percentage of the product composition in the evolved gases from gas chromatography analysis was defined as purity (e.g., H_2_, CO_x_, C_2+_, and CH_4_). The gas purity, H_2_ and C efficiency, and the weight ratio of gas, oil and carbon after plastic decomposition was calculated by the following equation, respectively:

1 $${{{\rm{H}}}}_{2}{{{\rm{efficiency}}}}=\frac{{{{\rm{Total}}}} \,{{{\rm{mass}}}} \,{{{\rm{of}}}} \,{{{\rm{Hin}}}} \,{{{\rm{H}}}}_{2}({{{\rm{g}}}})}{{{{\rm{The\,oretical}}}} \,{{{\rm{mass}}}} \,{{{\rm{of}}}} \,{{{\rm{H}}}} {{{\rm{in}}}} {{{\rm{plastic}}(g)}}}(\%)$$

2 $${{{\rm{Gas}}}} {{{\rm{purity}}}}=\frac{{{{\rm{X}}}}_{{{\rm{n}}}}}{{\sum }_{1}^{{{\rm{n}}}}{{{\rm{X}}}}_{{{\rm{n}}}}} \times 100\%({{{\rm{vol}}}}\%)$$

3 $${{{\rm{Carbon}}}} \,{{{\rm{efficiency}}}}=\frac{{{{\rm{Total}}}} \,{{{\rm{mass}}}} \,{{{\rm{of}}}} \,{{{\rm{C}}}} {{{\rm{in}}}} {{{\rm{carbon}}}} \,{{{\rm{materials}}}}({{{\rm{g}}}})}{{{{\rm{The\,oretical}}}} \,{{{\rm{mass}}}} \,{{{\rm{of}}}} \,{{{\rm{C}}}} {{{\rm{in}}}} {{{\rm{plastic (g)}}}}}(\%)$$

4 $${{{\rm{Gas}}}} \,{{{\rm{weight}}}} \,{{{\rm{ratio}}}}=\frac{{{{\rm{Mass}}}} \,{{{\rm{of}}}} \,{{{\rm{gas}}}}}{{{{\rm{Mass}}}} \,{{{\rm{of}}}}\, {{{\rm{plastic}}}}}\times 100\%({{{\rm{wt}}}}\%)$$

5 $${{{\rm{Carbon}}}} \,{{{\rm{weight}}}} \,{{{\rm{ratio}}}}=\frac{{{{\rm{Mass}}}} \,{{{\rm{of}}}} \,{{{\rm{spent}}}} \,{{{\rm{catalyst}}}}-{{{\rm{mass}}}} \,{{{\rm{of}}}} \,{{{\rm{fresh}}}}\,{{{\rm{catalyst}}}}}{{{{\rm{Mass}}}} \,{{{\rm{of}}}} \,{{{\rm{plastic}}}}}\times 100\%({{{\rm{wt}}}}\%)$$

6 $${{{\rm{Oil}}}} \,{{{\rm{weight}}}} \,{{{\rm{ratio}}}}=\frac{{{{\rm{Mass}}}} \,{{{\rm{of}}}} {{{\rm{oil}}}}}{{{{\rm{Mass}}}} \,{{{\rm{of}}}} \,{{{\rm{plastic}}}}}\times 100\%({{{\rm{wt}}}}\%)$$

7 $${{{\rm{TON}}}}=\frac{{\sum }_{1}^{{{{\rm{n}}}}}{{{\rm{m}}}}_{{{\rm{plastic}}}} {{{\rm{n}}}}\times {{{\rm{Y}}}}_{{{\rm{carbon}}}} {{{\rm{n}}}}}{{{{\rm{M}}}}_{{{\rm{carbon}}}}}\div\left(\frac{{{{{\rm{m}}}}}_{{{{\rm{Ni}}}}}}{{{{{\rm{M}}}}}_{{{{\rm{Ni}}}}}}+\frac{{{{{\rm{m}}}}}_{{{{\rm{Fe}}}}}}{{{{{\rm{M}}}}}_{{{{\rm{Fe}}}}}}\right)$$

8 $${{{\rm{Conversion}}}}=\frac{{{{\rm{Total}}}} \,{{{\rm{mass}}}}\, {{{\rm{of}}}} \,{{{\rm{gas}}}},{{{\rm{oil}}}} \,{{{\rm{and}}}} \,{{{\rm{carbon}}}}({{{\rm{g}}}})}{{{{\rm{Mass}}}} \,{{{\rm{of}}}} \,{{{\rm{plastic}}}}({{{\rm{g}}}})}\times 100(\%)$$

9 $${{{\rm{H}}}}_{2}{{{\rm{yield}}}}=\frac{{\sum }_{1}^{{{\rm{n}}}}{{{\rm{Efficiency}}}}_{{{{\rm{H}}}}_{{{\rm{2 n}}}}}\times {{{\rm{Theoretical}}}} \,{{{\rm{molar}}}} \,{{{\rm{content}}}} \,{{{\rm{of}}}}\,{{{\rm{H}}}}_{2}\,{{{\rm{in}}}}\,{{{\rm{plastic}}}}_{{{\rm{n}}}}}{{{{\rm{Mass}}}} \,{{{\rm{of}}}} \,{{{\rm{catalyst}}}}}({{{\rm{mmol}}}}{{{\rm{g}}}}_{{{\rm{catalyst}}}}^{-1})$$

10 $${{{\rm{CNT}}}} \,{{{\rm{yield}}}}=	 \frac{{\sum }_{1}^{{{\rm{n}}}}{{{\rm{Efficiency}}}}_{{{{\rm{C}}}} \,{{{\rm{n}}}}}\times {{{\rm{Theoretical}}}} \,{{{\rm{mass}}}} \,{{{\rm{content}}}} \,{{{\rm{of}}}} \,{{{\rm{C}}}} \,{{{\rm{in}}}}\,{{{\rm{plastic}}}}_{{{\rm{n}}}}}{{{{\rm{Mass}}}} \,{{{\rm{of}}}} \,{{{\rm{catalyst}}}}}\\ 	 \times {{{\rm{CNT}}}} \,{{{\rm{purity}}}}\,({{{\rm{mg}}}}\,\,{{{\rm{g}}}}_{{{\rm{catalyst}}}}^{-1})$$

Herein, CNT purity refers to the purity of CNTs in the carbon material from the final cycle, as determined by thermogravimetric analysis. The theoretical molar content of H_2_ in plastics was calculated by multiplying the respective masses of the plastic mixture by the sum of their corresponding hydrogen contents, then dividing by hydrogen’s molecular weight. The theoretical carbon content was determined by multiplying the respective masses of the plastic mixture by the sum of their corresponding carbon contents, then dividing by carbon’s atomic weight^17,63^.

TON refers to the turnover number, which represents the total molar carbon produced per mole of NiFe. *X*_*n*_ refers to the volume fraction of gaseous n. *m*_*plastic n*_ and *Y*_*carbon n*_ refer to the mass of plastic and the weight ratio of carbon in cycle n. *M*_*carbon*_, *M*_*Ni*_, *M*_*Fe*_, *m*_*Ni*,_ and *m*_*Fe*_ refer to the mole mass of carbon, the mole mass of Ni, the mole mass of Fe, the mass of Ni, and the mass of Fe.

### Techno-economic analysis

Employing comprehensive techno-economic analysis (TEA), based on the Cash Flow (CF) method, to evaluate the economic feasibility of implementing a commercial-scale process for microwave catalytic decomposition of plastic mixtures^56,57,64^. TEA considered all costs associated with the project, including investment costs (e.g., equipment purchases, construction expenses, labor costs, etc.) and operating costs (e.g., maintenance expenses, operating expenses, management expenses, etc.). Moreover, the life cycle costs of the project were also considered, including the cost of the investment phase, the operation phase, and the disposal phase. Meanwhile, to make the assessment more reasonable, changes in the currency over a time period were also taken into account. Finally, the expected economic benefits of the project were evaluated.

## Supplementary information

Supplementary Information

Description of Additional Supplementary Files

Supplementary Movie 1

Transparent Peer Review file

## Source data

Source Data

## Data Availability

The research data generated in this study are provided in the Supplementary Information/Source Data file. All data are available from the corresponding author upon request. Source data are provided with this paper.
