# Supplementary material for: ~100% upcycling of chlorinated/fluorinated plastic mixtures to H2 and nanotubes over FeNi/Ni/C by microwave catalysis
Source: Nat Commun. 2026 May 15;17:6481. doi: 10.1038/s41467-026-73141-w (PMC13376195; doi:10.1038/s41467-026-73141-w)
Supplement: Supplementary file 1 — Supplementary Information [file 41467_2026_73141_MOESM1_ESM.pdf]

Supplementary Materials for

~100% upcycling of chlorinated/fluorinated plastic mixtures to H_2 and nanotubes over FeNi/Ni/C by microwave catalysis

Jun Zhao [1], Jianlong Yang [2,3], Duanda Wang [4,5], Huanrong Zhang [5,6], Qianqian Jia [1,5], Lei Zhang [1], Zhenguo An [1], Mianqi Xue [6], Haijiao Xie [7], Wangjing Ma [1, *], Lu Zhang [1], Sui Zhao [1], Junwang Tang [2, *]

[1]State Key Laboratory of Cryogenic Science and Technology, Technical Institute of Physics and Chemistry, Chinese Academy of Sciences, Zhongguancun East Road, Haidian District, Beijing, 100190

[2]Industrial Catalysis Center, Department of Chemical Engineering, Tsinghua University, Beijing 100084, China

[3]Yulin Innovation Institute of Clean Energy, Yulin, 719000, China

[4]Laboratory of Bio-Inspired Smart Interface Science, Technical Institute of Physics and Chemistry, Chinese Academy of Sciences, Zhongguancun East Road, Haidian District, Beijing, 100190

[5]University of Chinese Academy of Sciences, Zhongguancun East Road, Haidian District, Beijing 100049, China

[6]National Engineering Research Center for Engineering Plastics, Technical Institute of Physics and Chemistry, Chinese Academy of Sciences, Zhongguancun East Road, Haidian District, Beijing, 100190

[7]Hangzhou Yanqu Information Technology Co., Ltd. No. 712 Wen'er West Road, Xihu District, Hangzhou 310003, China

*Corresponding Author. Email: wjma@mail.ipc.ac.cn; jwtang@tsinghua.edu.cn

1. Supplementary Figures

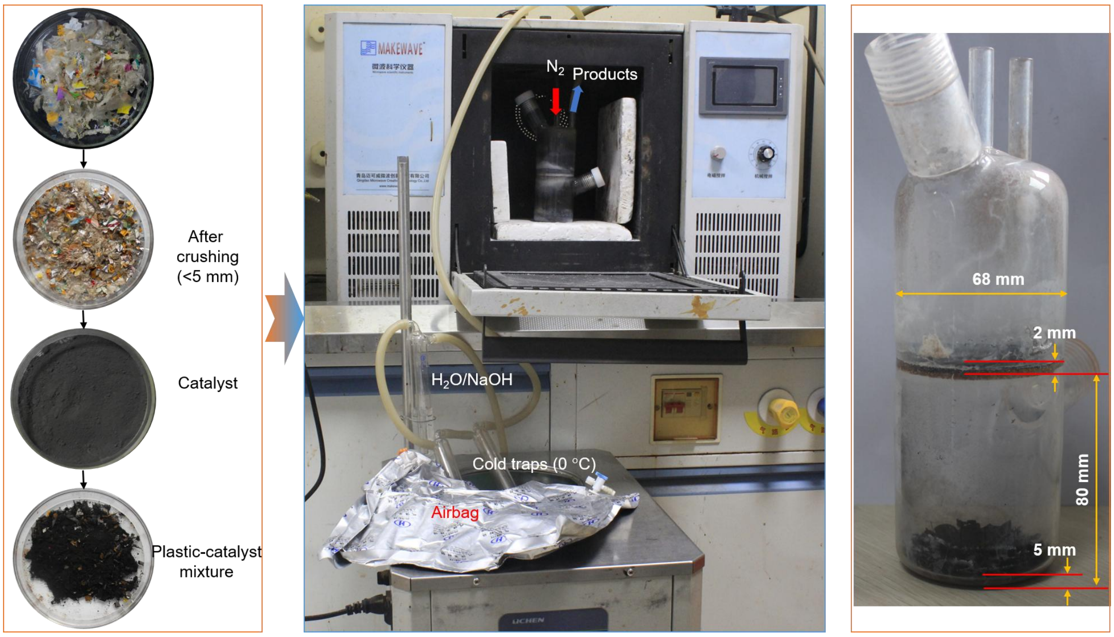

Supplementary Fig. 1. The diagram of the reaction instrument.

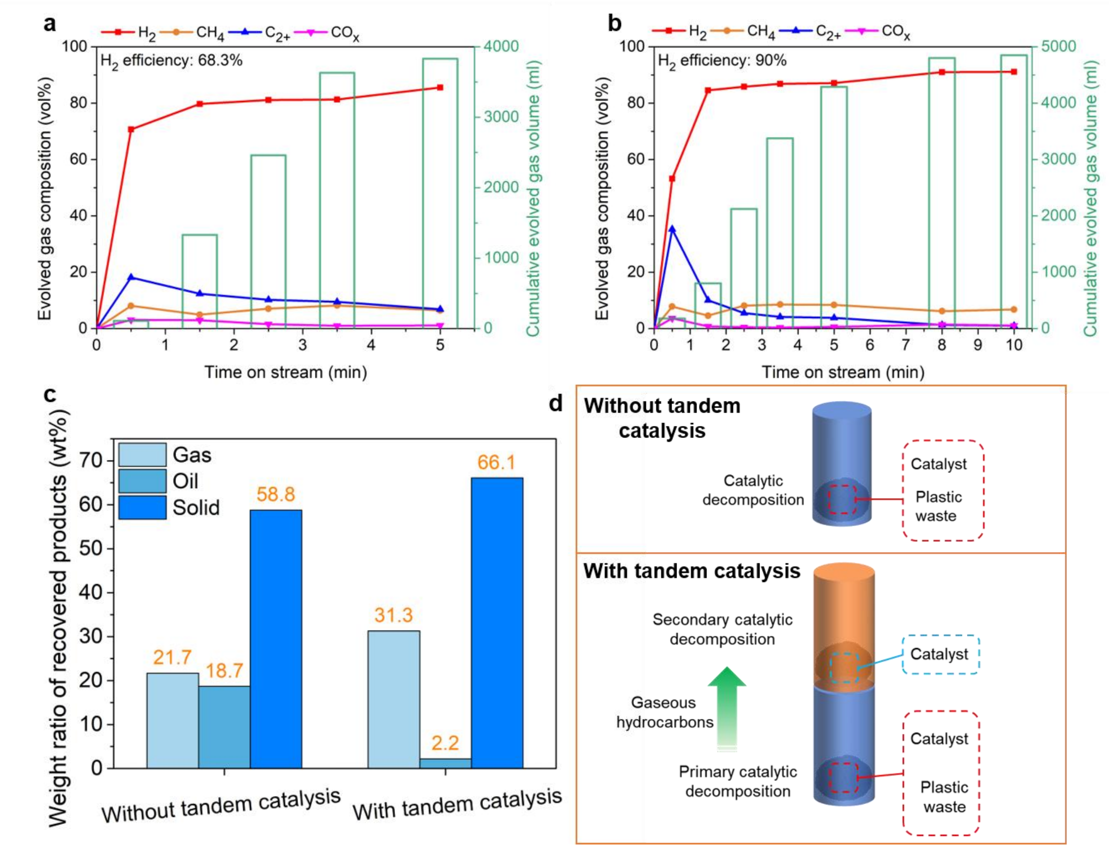

Supplementary Fig. 2. Comparison of the product distribution with and without tandem catalysis. A time-on-stream analysis of the microwave catalytic decomposition of LDPE using FeC-FeNi/Ni/C. (a) Without tandem catalysis. (b) With tandem catalysis (Reaction duration is lower than 8 min). (c) The weight ratio of recovered gas, oil and solid. (d) Schematic diagram of the reactor.

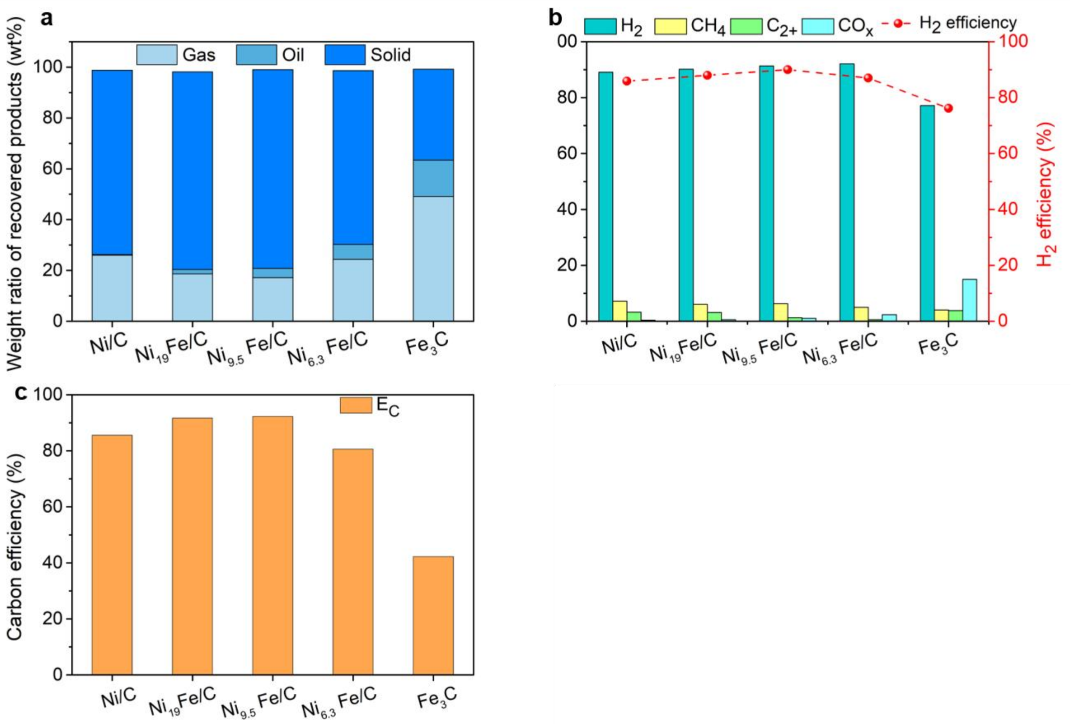

Supplementary Fig. 3. Optimization of the catalyst composition. (a) The weight ratio of recovered gas, oil and solid. (b) Corresponding evolved gas composition (vol%) and H_2 efficiency (%). (c) Carbon efficiency (%). Plastic: LDPE.

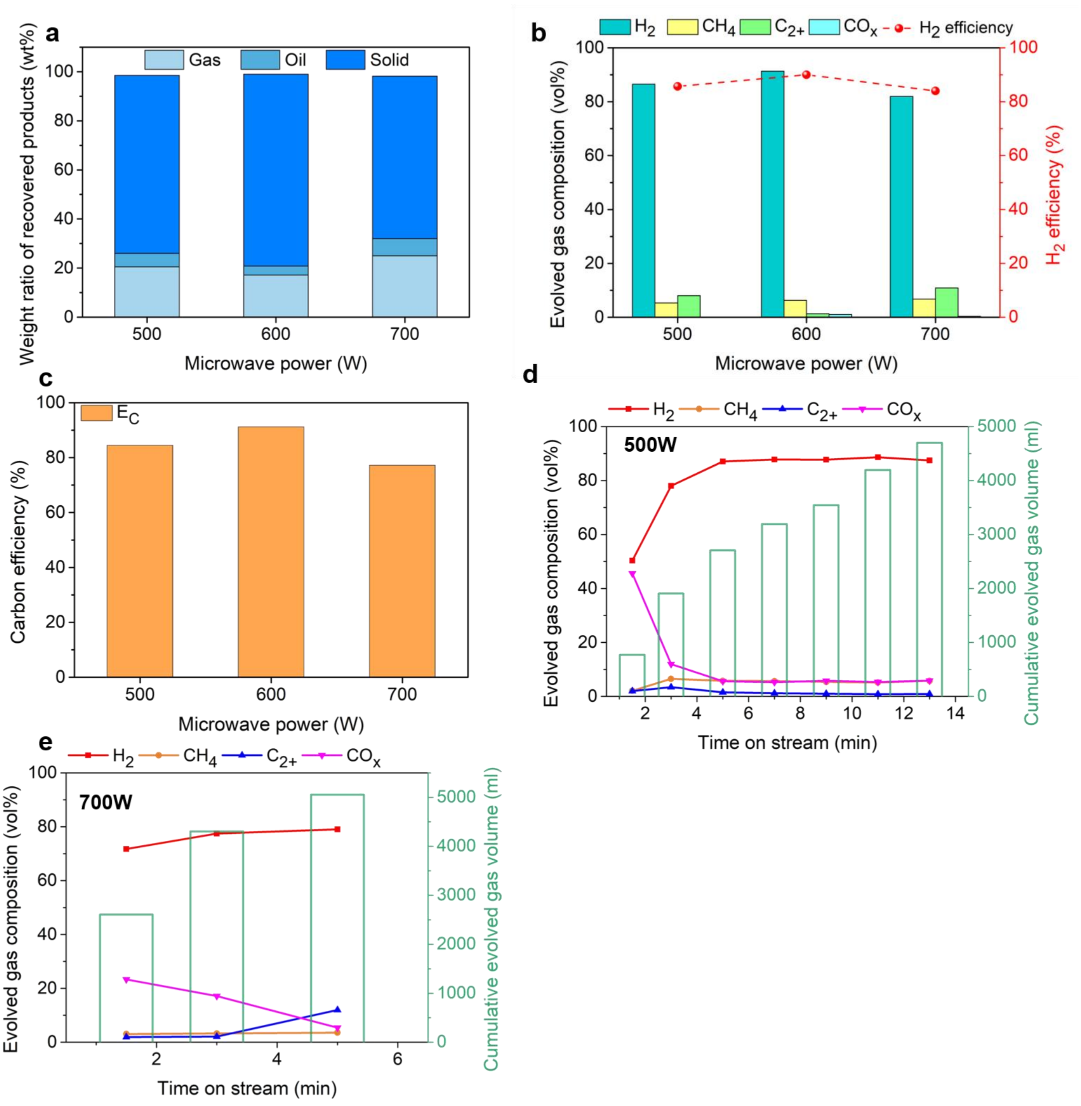

Supplementary Fig. 4. Optimization of the microwave irradiation power. (a) The weight ratio of recovered gas, oil and solid. (b) Corresponding evolved gas composition (vol%) and H_2 efficiency (%). (c) Carbon efficiency (%). (d) A time-on-stream analysis of the microwave catalytic decomposition of LDPE with the microwave irradiation power of 500 W (Reaction duration is about 13 min.). (e) A time-on-stream analysis of the microwave catalytic decomposition of LDPE with the microwave irradiation power of 700 W (Reaction duration is lower than 5 min.). Tandem catalysis method. Plastic: LDPE.

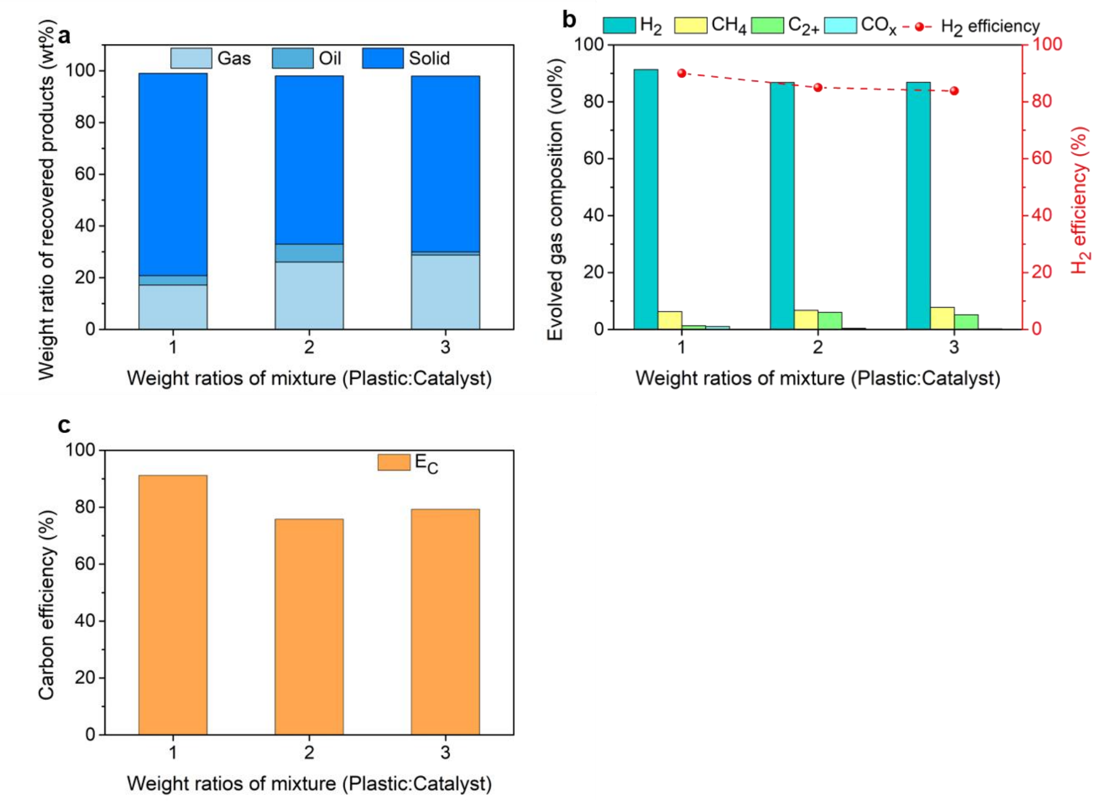

Supplementary Fig. 5. Optimization of the weight ratio of the mixture (plastic: catalyst) in the bottom layer. (a) The weight ratio of recovered gas, oil and solid. (b) Corresponding evolved gas composition (vol%) and H_2 efficiency (%). (c) Carbon efficiency (%). Tandem catalysis method. Plastic: LDPE.

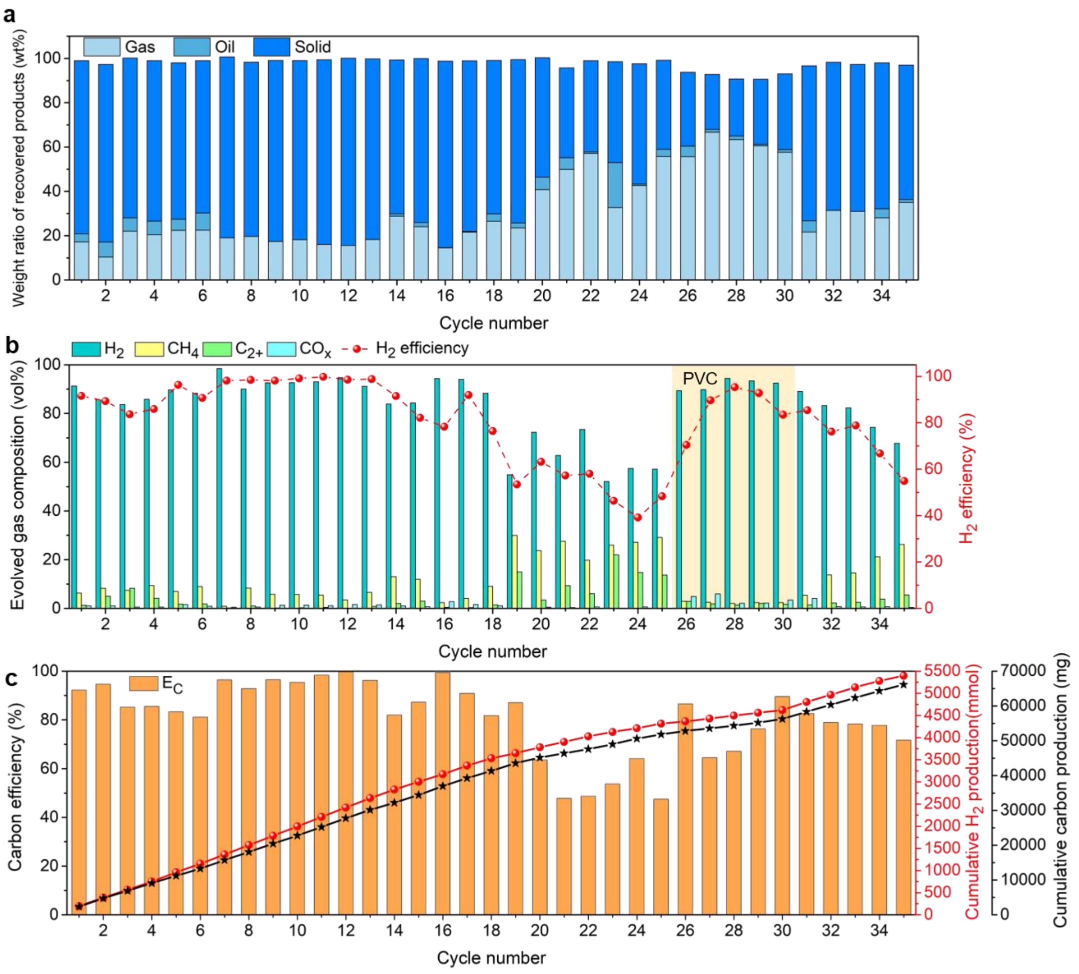

Supplementary Fig. 6. Successive cycles of microwave catalytic decomposition of LDPE or PVC using FeNi/Ni/C catalyst. (a) The weight ratio of recovered gas, oil and solid. (b) Evolved gas composition (vol%) and H_2 efficiency (%). (c) Carbon efficiency (%), cumulative H_2 production (mmol) and cumulative carbon production (mg). Tandem catalysis method. Plastic: LDPE (Cycle 1 ~ Cycle 25 and Cycle 31~Cycle 35), PVC (Cycle 26~Cycle 30). Reaction conditions: 3 g LDPE/PVC and 3 g FeNi/Ni/C catalyst in the bottom layer; 3 g FeNi/Ni/C catalyst in the top layer.

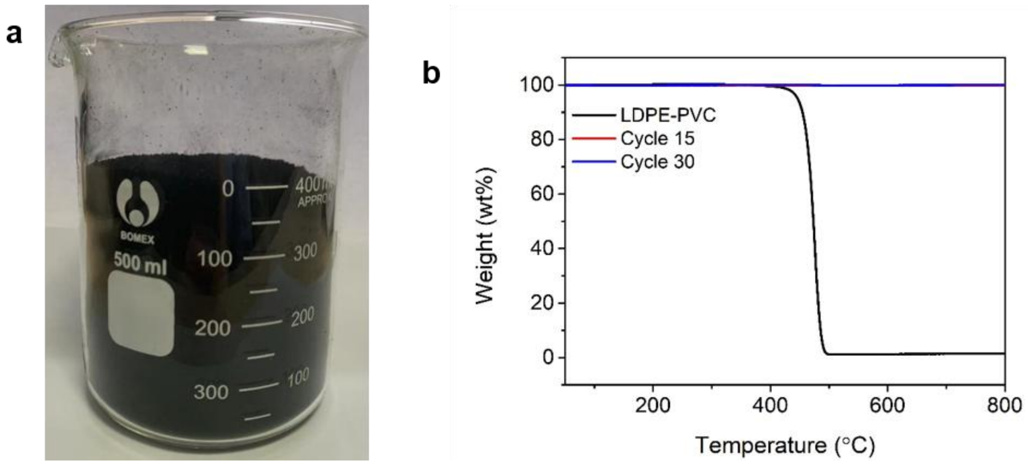

Supplementary Fig. 7. (a) The optical image of the CNTs produced after microwave catalytic decomposition of the LDPE and PVC mixture (9:1) with FeNi/Ni/C catalyst. (b) Thermogravimetric analysis results of the LDPE-PVC plastic mixture and the used catalyst after 15 and 30 catalytic cycles.

To verify the absence of unreacted plastic residues in the catalyst after each use, a new thermogravimetric analysis was performed on samples collected after different reaction cycles under a N_2 atmosphere. As illustrated in Supplementary Fig. 7b, the LDPE-PVC (9:1) mixture exhibits complete decomposition at approximately 500 °C, whereas the used catalyst samples after 15 and 30 cycles show negligible weight loss within this temperature range. This indicates minimal carryover of unreacted plastics into subsequent reaction cycles, supporting the sustained efficiency of the microwave-assisted catalytic plastic decomposition process.

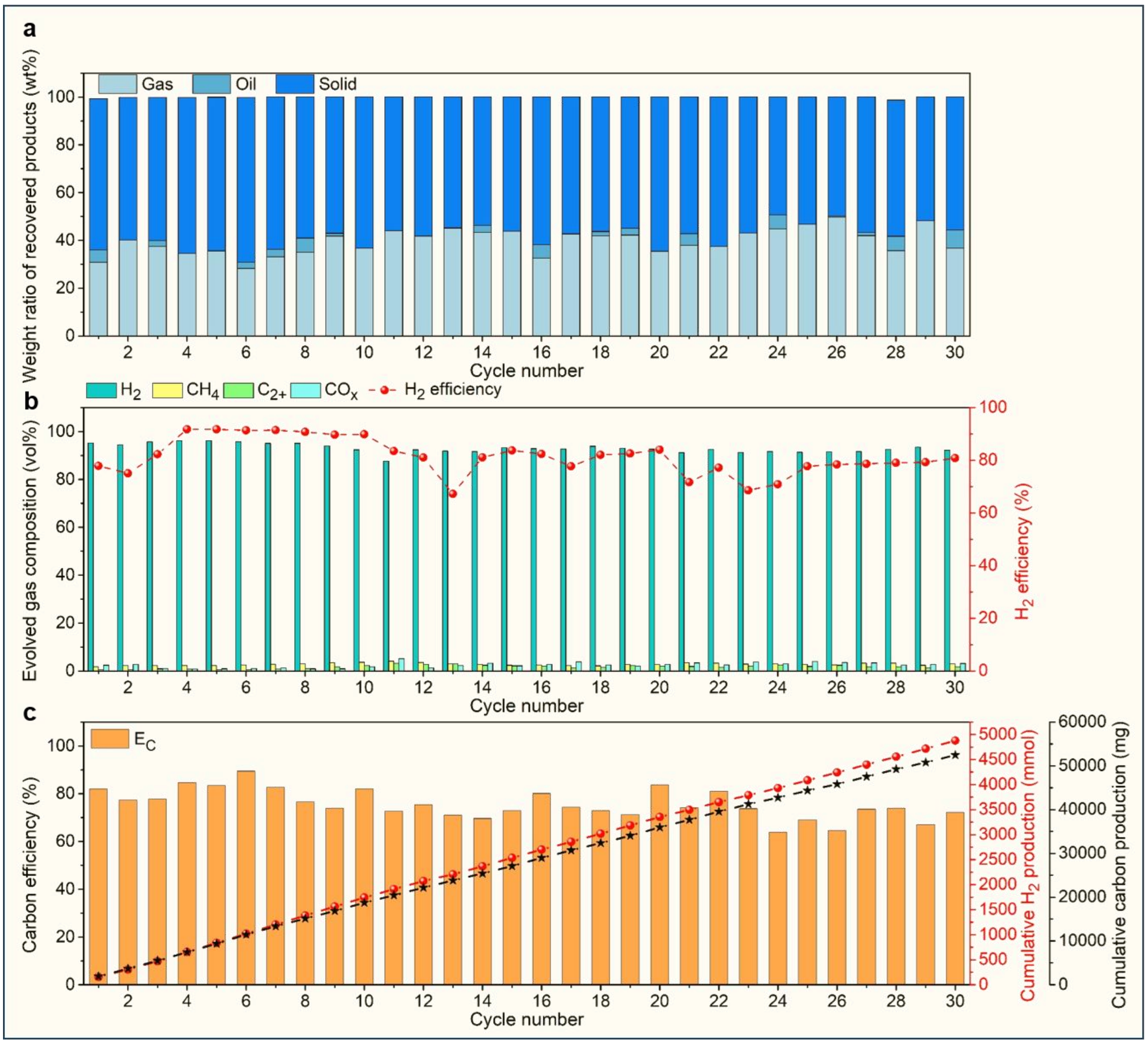

Supplementary Fig. 8. Successive cycles of microwave catalytic decomposition of plastic over FeNi/Ni/C catalyst. (a) The weight ratio of recovered gas, oil and solid. (b) Evolved gas composition (vol%) and H_2 efficiency (%). (c) Carbon efficiency (%) (E_C), cumulative H_2 production (mmol) and cumulative carbon production (mg). Reaction conditions: A mixture of 3 g plastic and 6 g FeNi/Ni/C catalyst. Plastic: LDPE and PVC mixture (the mass ratio of LDPE to PVC is 9:1).

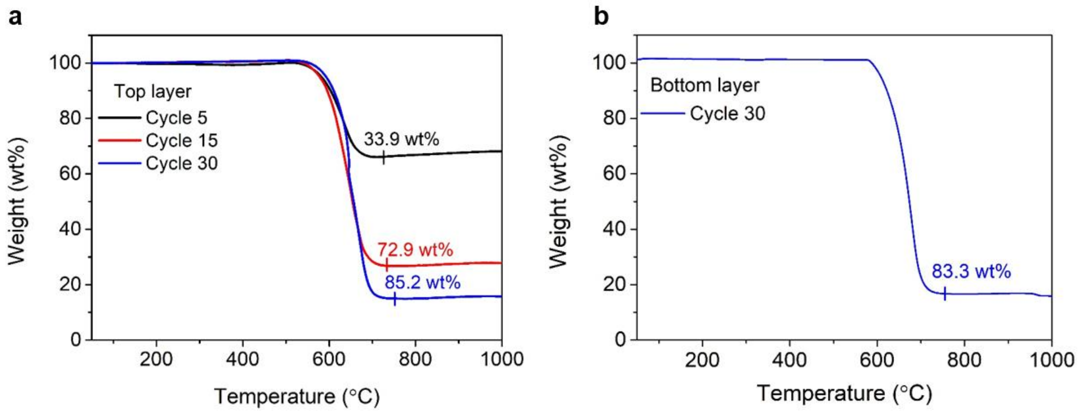

Supplementary Fig. 9. Thermogravimetric analysis of the top (a) and bottom (b) catalyst after use.

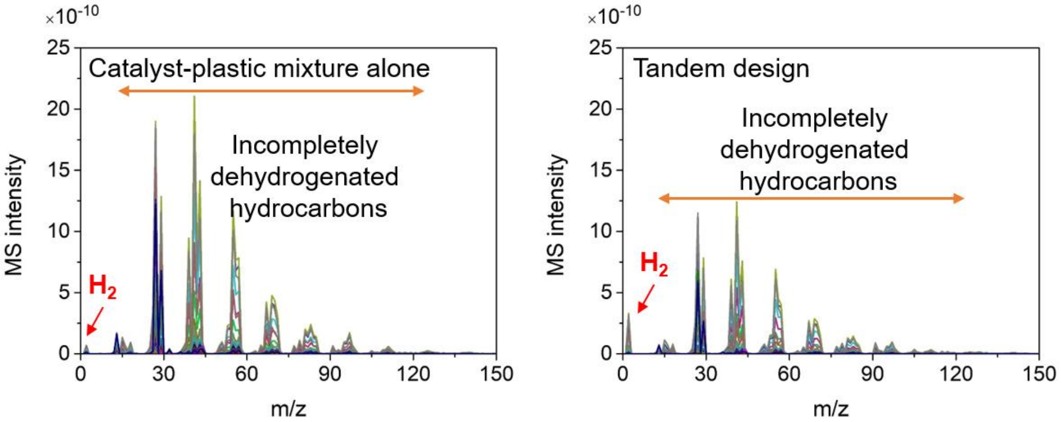

Supplementary Fig. 10. TG-MS analysis of the differences in the product distribution of only catalyst-plastic mixture reaction modes and tandem catalytic modes.

To demonstrate the superiority and functionality of tandem design, 30 new successive cycles of decomposition experiments were conducted using an LDPE and PVC mixture (LDPE: PVC = 9:1) under two conditions: with the tandem design and with only the bottom layer. In the tandem design, the catalytic system exhibits consistent performance: over 95 wt% of the plastic mixture is converted into gas and solid carbon in each cycle, with only small amounts of organic by-products generated. H_2 efficiency ranges between 85.7% and 98.2% with a purity of 89.4-97.0 vol%, while carbon efficiency ranges from 83.3% to 99.9%. Corresponding yields of H_2 and CNTs reach 930 mmol $g^{-1}_{catalyst}$ and 10608 mg $g^{-1}_{catalyst}$, respectively (Fig. 1).

In contrast, in the presence of the bottom layer, hydrogen efficiency is 67-92% (purity: 87-96 vol%) and carbon efficiency is 63-89%. The respective H_2 and carbon yields are 812.9 mmol $g^{-1}_{catalyst}$ and 8743 mg $g^{-1}_{catalyst}$, which are significantly lower than those of the tandem design (Supplementary Fig. 8). Air atmosphere thermogravimetric analysis reveals increased carbon content in both top and bottom catalyst layers with cycling, suggesting the top catalyst facilitates dehydrogenation of hydrocarbons generated by the bottom catalyst, improving the catalytic efficiency (Supplementary Fig. 9). In addition, thermogravimetric mass spectrometry (TG-MS) results further support the above discussion: relative to the tandem catalytic mode, the standalone catalyst-plastic mixture mode produces less hydrogen and more hydrocarbons in the 10-90 m/z range (Supplementary Fig. 10). This further indicates that the tandem mode can further catalyze the hydrocarbons generated during the reaction in the bottom layer, thereby enhancing hydrogen and carbon efficiency.

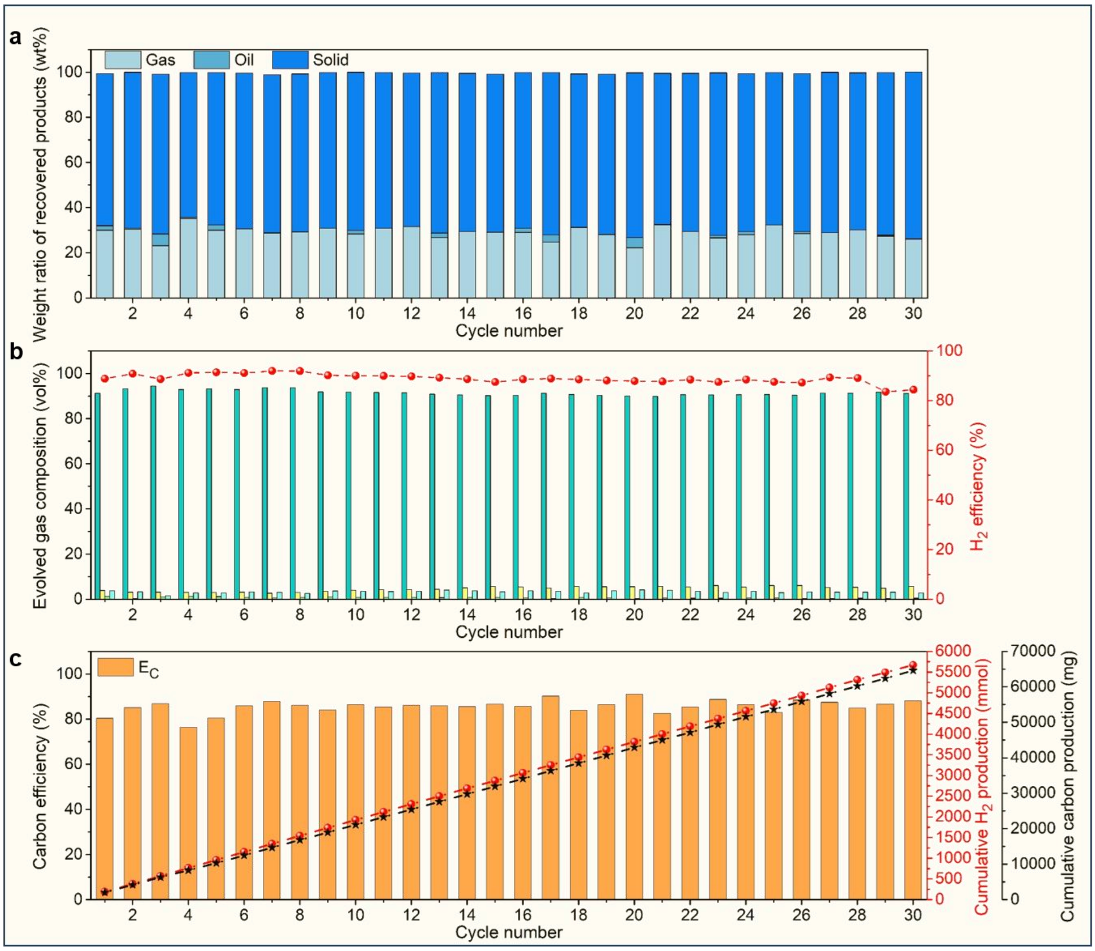

Supplementary Fig. 11. Successive cycles of microwave catalytic decomposition of LDPE-PVC plastic mixture over FeNi/Ni/C catalyst. (a) The weight ratio of recovered gas, oil and solid. (b) Evolved gas composition (vol%) and H_2 efficiency (%). (c) Carbon efficiency (%) (EC), cumulative H_2 production (mmol) and cumulative carbon production (mg). Reaction conditions: 3 g plastic and 3 g FeNi/Ni/C catalyst in the bottom layer; additional 3 g FeNi/Ni/C catalyst in the top layer. Plastic: LDPE and PVC mixture (the mass ratio of LDPE to PVC is 99:1).

To further investigate the catalytic performance of this strategy if PVC content is low, microwave catalytic decomposition experiments were conducted using LDPE-PVC mixtures with reduced PVC levels. As shown in Supplementary Fig. 11, the strategy maintains good catalytic performance even with PVC contents as low as 1% (LDPE: PVC=99:1) over 30 successive cycles. Notably, over 92.5 wt% of the mixture is converted into gas and solid carbon per cycle. H_2 efficiency ranges from 83.5% to 91.4% with a purity of 89.8-94.4 vol%, while carbon efficiency correspondingly ranges from 76.3% to 90.9%. Respective yields of H_2 and CNTs reach 945.2 mmol $g^{-1}_{catalyst}$ and 10,768 mg $g^{-1}_{catalyst}$. These results demonstrate that even with a minor PVC fraction, the strategy achieves relatively good catalytic decomposition of the plastic mixture into H_2 and carbon nanotubes.

Additionally, we have designed a process to simulate the energy absorbed by the sample, which involves heating the sample in a microwave field for a fixed duration, followed by rapid transfer to room-temperature water. By measuring the temperature rise of the water, the energy absorbed by the sample was calculated using the formula *$Ea=m\cdot c_p\cdot \Delta T$ (m, cp, and ΔT are the mass of water, the heat capacity of water, and the temperature difference before and after heating water, respectively)*. This value and reaction heat were subsequently compared with the total microwave input energy (*$Ei=P\cdot t$, P and t are microwave power and heating time, respectively*) to assess microwave energy utilization efficiency. Note: This procedure involves inherent risks.
Given LDPE's simple molecular structure and the relative ease of calculating its reaction enthalpy, catalytic decomposition experiments were conducted using a 99:1 mass ratio of LDPE-PVC plastic mixtures as feedstock. For Cycle 1, 3 g of the plastic mixture was combined with 3 g of FeNi/Ni/C catalyst and loaded into the bottom reactor layer, with an additional 3 g of FeNi/Ni/C catalyst placed in the upper layer; following 5 minutes of microwave irradiation at 600 W, the reactor was immediately immersed in room-temperature water, and once thermal equilibrium was reached, the water temperature was measured to calculate the temperature change relative to the initial value. For Cycle 15, 3 g of the plastic mixture was mixed with the used bottom layer catalyst from previous cycles and loaded into the bottom reactor chamber, with upper layer catalyst retained in the upper layer, and the same irradiation and temperature measurement protocol as Cycle 1 was followed. Experimental results indicate that the energy absorbed by the water is 24,660 J and 86,320 J for Cycle 1 and Cycle 15, respectively, while the total microwave input energy for each experiment is 180,000 J.
For the reaction heat of LDPE plastic decomposition, it is calculated by Benson's group additivity method[1,2].

$$(CH_2)_n \longrightarrow nH_2 + nC$$

$$\Delta_r H = n\Delta_r H_{H_2} + n\Delta_r H_C - n\Delta_r H_{CH_2}$$

$$\mathrm{n} = \frac{m_{PE}}{M_{CH_2}}$$

Wherein, $\Delta_r H$, $n\Delta_r H_{H_2}$, $n\Delta_r H_C$, and $n\Delta_r H_{CH_2}$ correspond to the reaction enthalpy, enthalpy of hydrogen formation, enthalpy of carbon formation, and enthalpy of polyethylene chain segment formation, respectively; m_{PE} and M_{CH_2} denote the mass of polyethylene plastic and the molar mass of the polyethylene repeating unit, respectively.

The heat of reaction for plastic decomposition calculated via the aforementioned process is 4395 J ($\Delta H_{f\ 298(H2)}^{o}$=0 kJ/mol, $\Delta H_{f\ 298(C)}^{o}$=0 kJ/mol, $\Delta H_{f\ 298(CH2)}^{o}$=-20.72 kJ/mol). Accordingly, the microwave energy absorbed by the samples should be 29055 J (24660 J + 4395 J, Cycle 1) and 90715 J (86320 J + 4395 J, Cycle 15), respectively, as the energy consumed for plastic decomposition is transferred from the catalyst materials[3]. Based on this, the microwave energy utilization efficiencies are

calculated to be 16.1% (100% × 29055 / 180000, Cycle 1) and 50.4% (100% × 90715 / 180000, Cycle 15), respectively. The enhanced microwave energy utilization efficiency after cycle 15 compared to that after cycle 1 is mainly attributed to the large quantity of CNTs generated during catalytic plastic decomposition, which is an excellent microwave-absorbing material, thus improving microwave absorption efficiency. On the other hand, the failure to achieve nearly full microwave energy utilization is due to the large volume of the reaction chamber and the insufficient dosage of microwave-absorbing materials, which results in incomplete absorption of microwave energy.

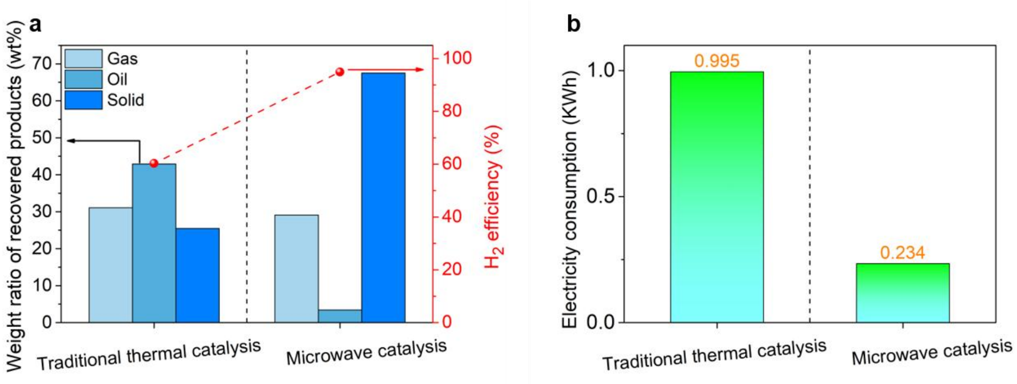

Supplementary Fig. 12. Comparison of thermal catalysis and microwave catalysis in terms of product distribution, H_2 efficiency (%) (a), and electricity consumption for decomposing 3 gram plastic waste (b).

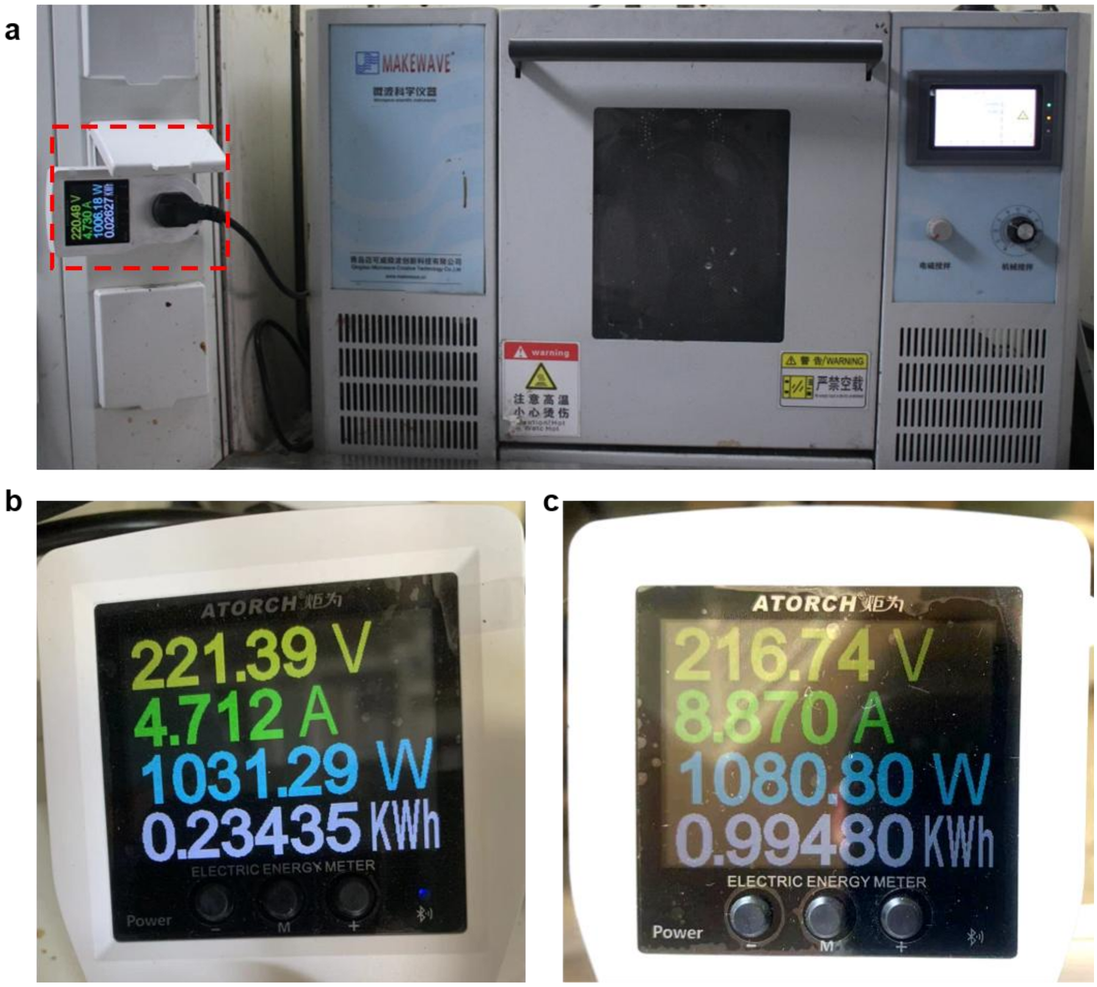

Supplementary Fig. 13. Measurement method of electricity consumption in microwave catalysis (a). Comparison of electricity consumption in traditional thermal catalysis and microwave catalysis. Microwave catalysis (b). Thermal catalysis (c).

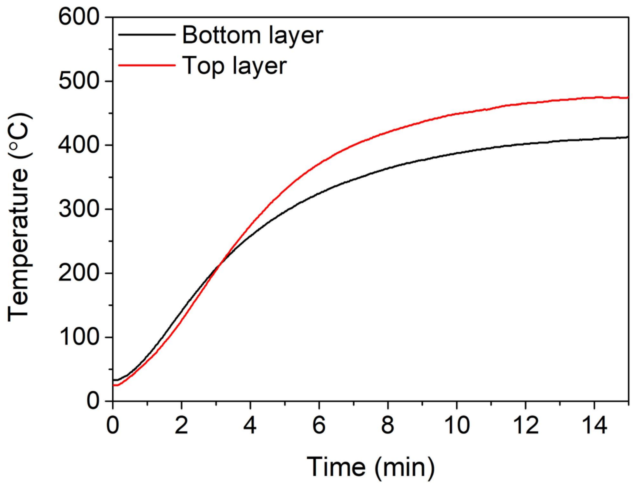

Supplementary Fig. 14. The bed temperature curves of the top and bottom layers during microwave catalysis.

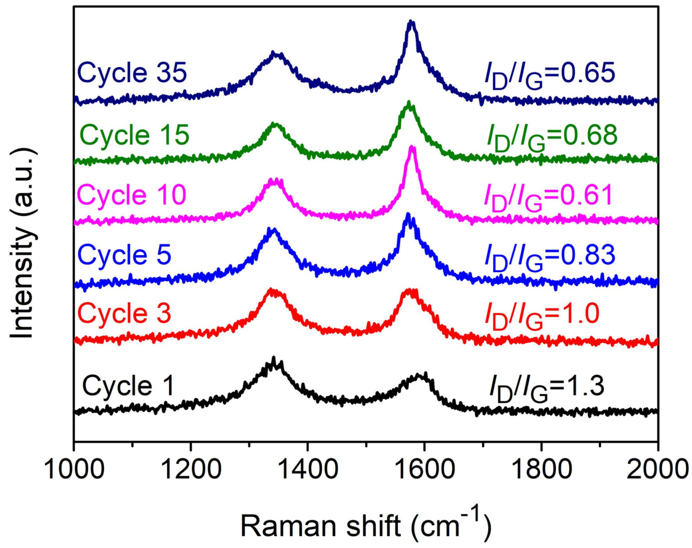

Supplementary Fig. 15. Raman analysis of the resulting carbon materials after different successive cycles.

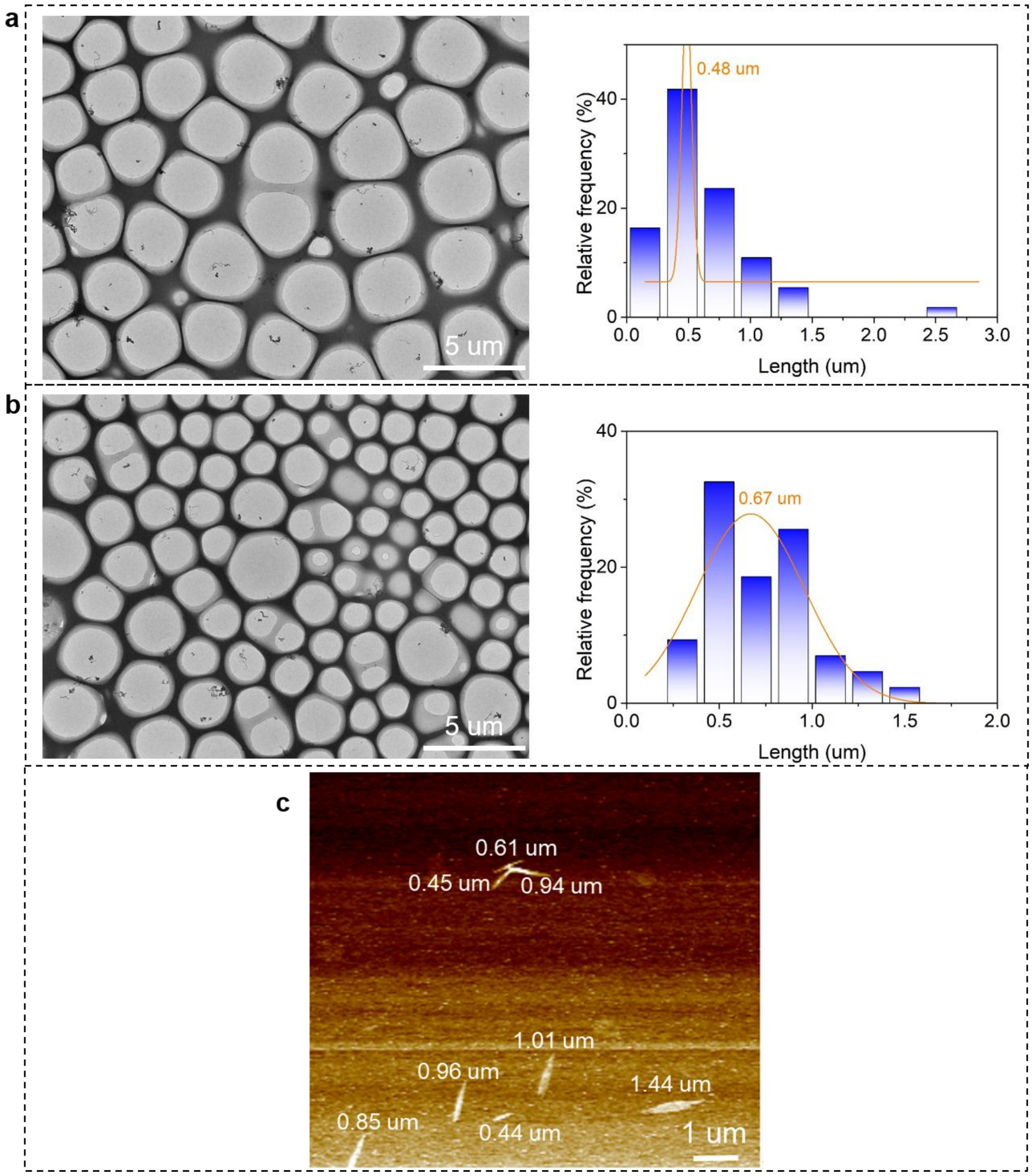

Supplementary Fig. 16. TEM analysis of the length distribution of CNTs obtained from cycle 1 (a) and cycle 30 (b). AFM analysis of the length distribution of CNTs obtained from cycle 30 (c).

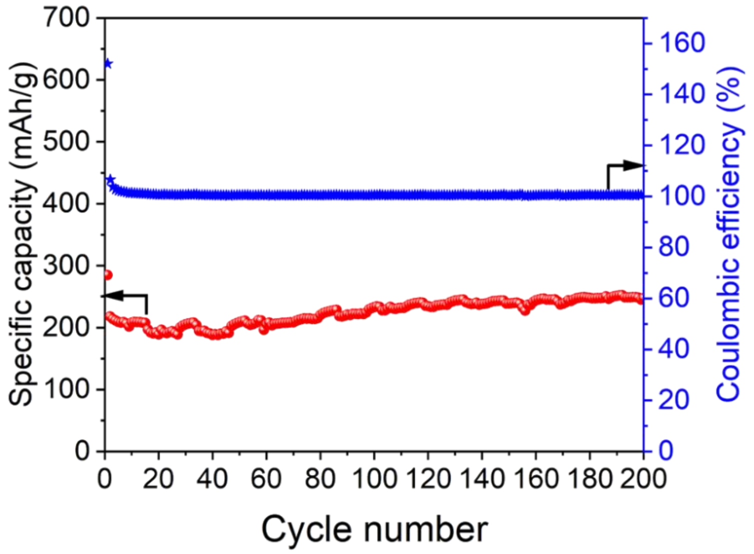

Supplementary Fig. 17. Lithium-ion storage performances of CNTs (as the cathode) acquired from microwave catalytic decomposition of LDPE or PVC using FeNi/Ni/C as a catalyst.

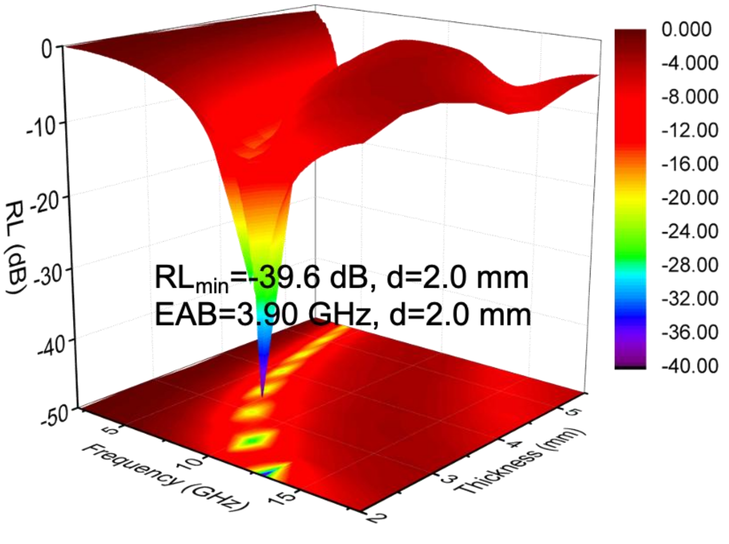

Supplementary Fig. 18. The electromagnetic wave absorption performance of CNTs acquired from microwave catalytic decomposition of LDPE or PVC using FeNi/Ni/C as a catalyst.

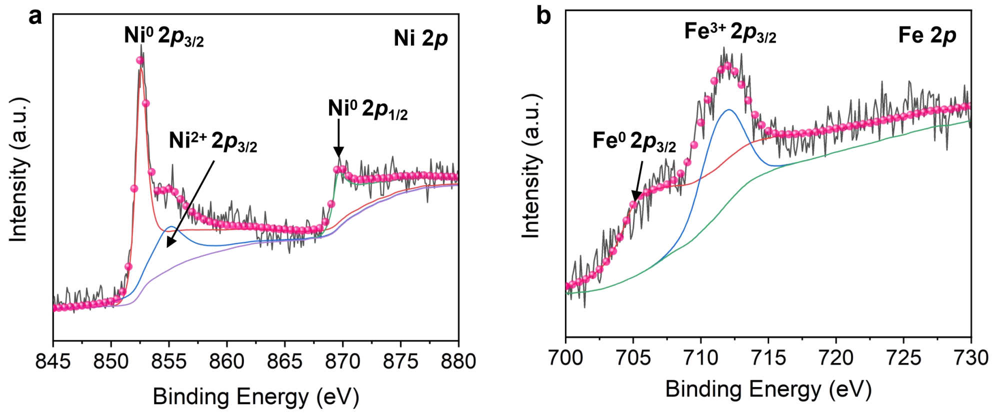

Supplementary Fig. 19. XPS analysis of FeC-FeNi/Ni/C. a) Ni 2p spectra. b) Fe 2p spectra.

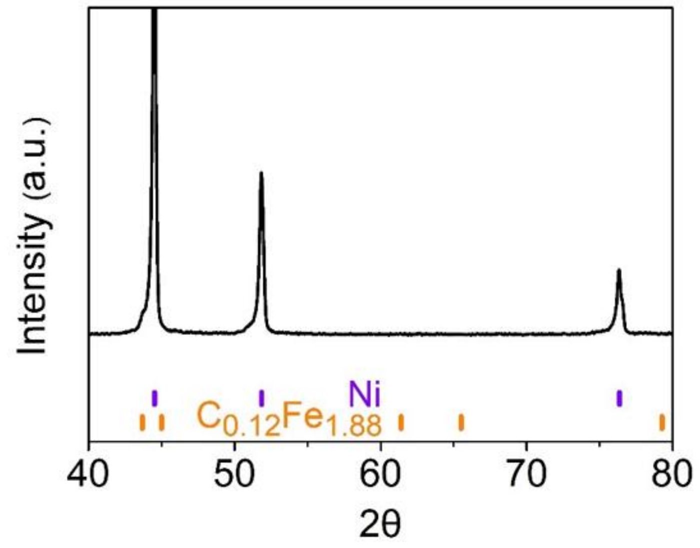

Supplementary Fig. 20. XRD pattern of FeC-FeNi/Ni/C.

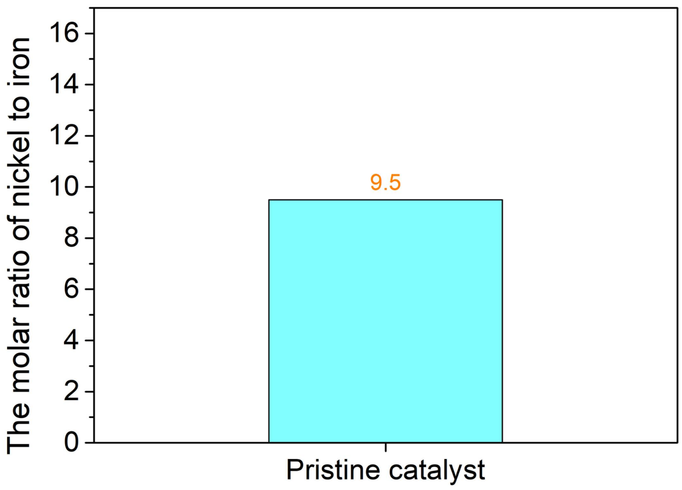

Supplementary Fig. 21. XRF analysis of FeC-FeNi/Ni/C.

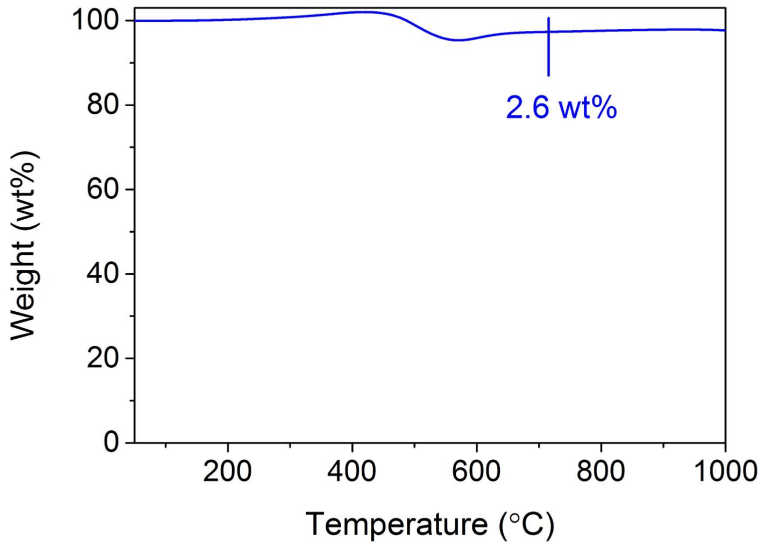

Supplementary Fig. 22. TGA curve of FeC-FeNi/Ni/C.

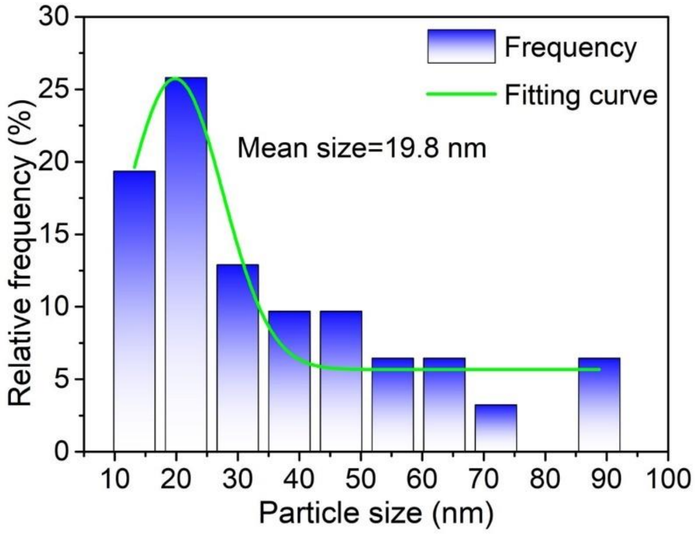

Supplementary Fig. 23. The particle size distribution of FeC-FeNi/Ni/C was determined by counting 30 particles. The size range and average size are 9-95 nm and 19.8 nm, respectively.

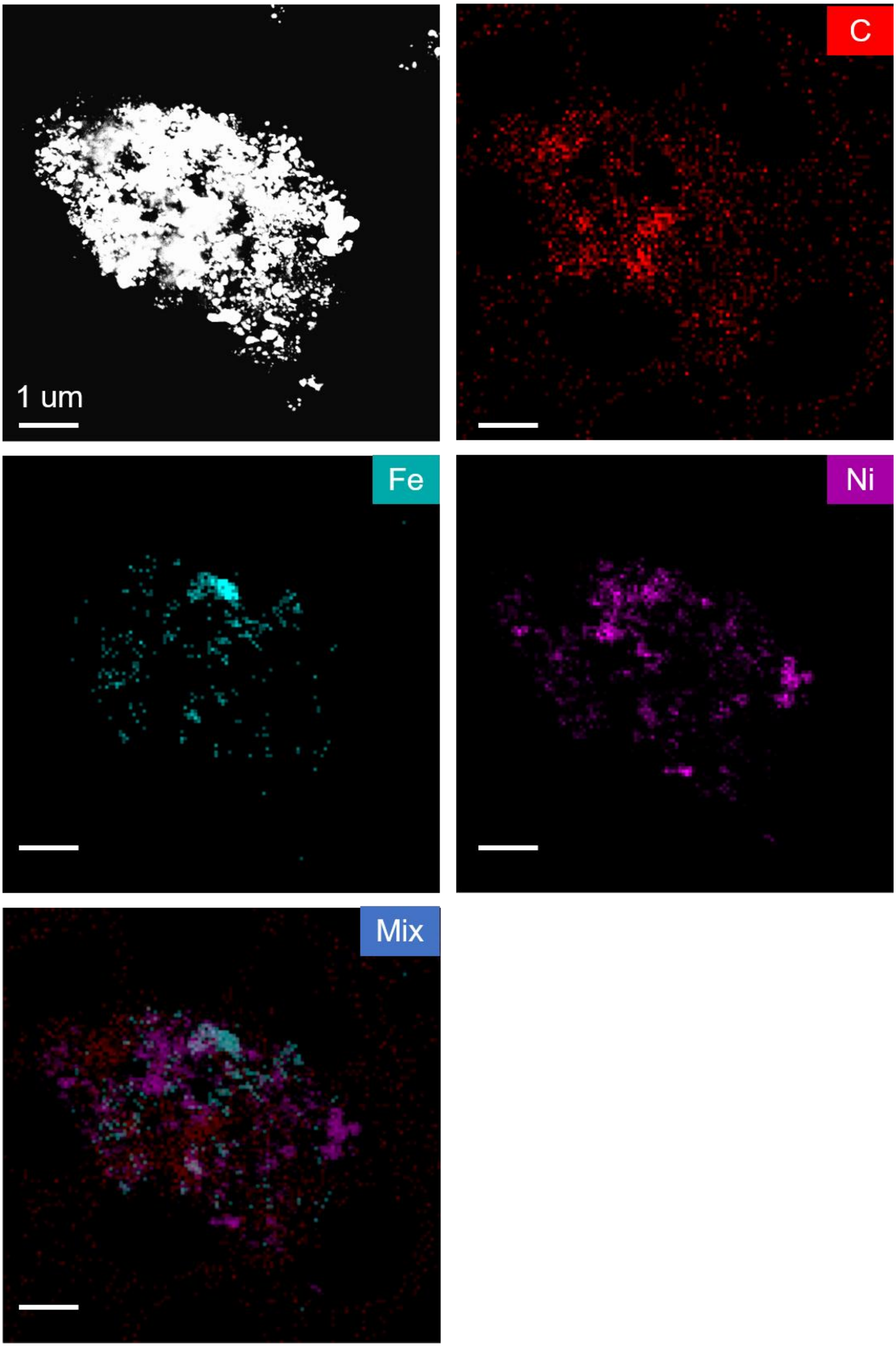

Supplementary Fig. 24. EDX mapping of FeC-FeNi/Ni/C. Ni and Fe atoms are separately dispersed.

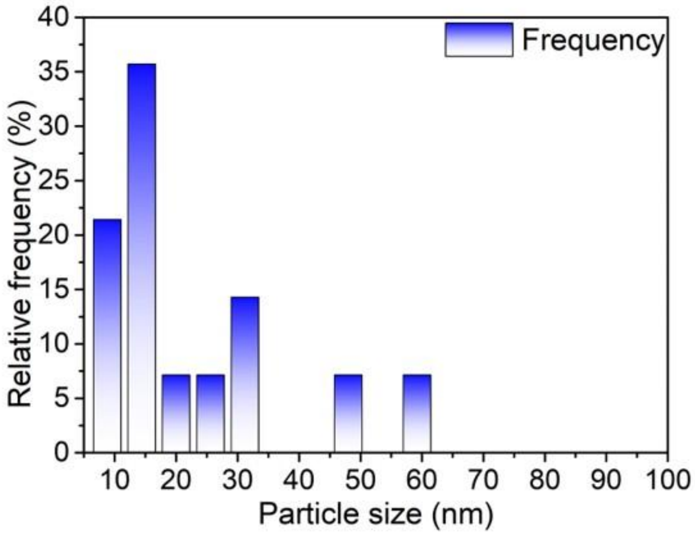

Supplementary Fig. 25. The particle size distribution of the FeNi/Ni/C catalyst after 15 successive cycles, which was determined by counting 30 particles. The size range and average size are 6-62 nm and 11.8 nm, respectively.

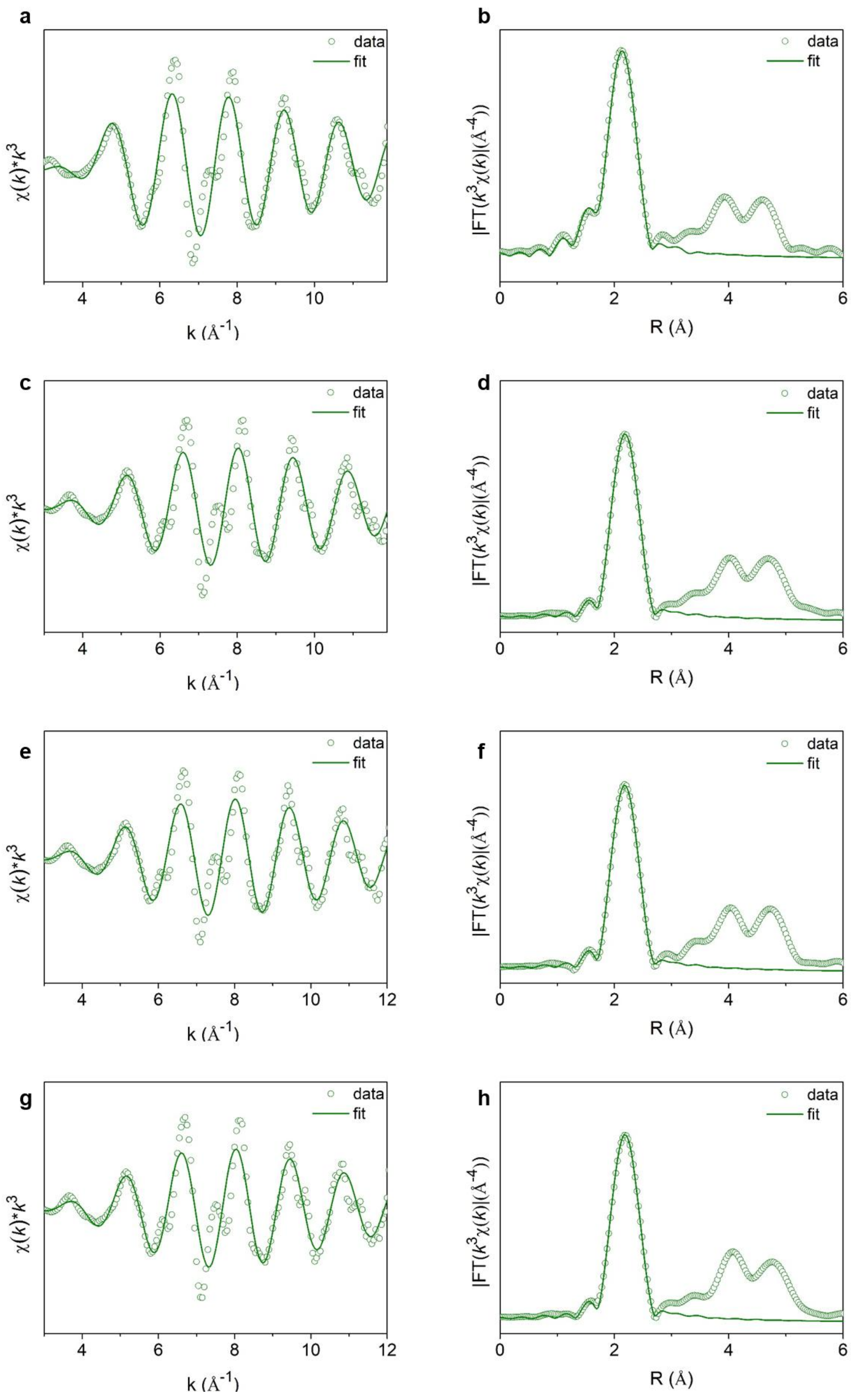

Supplementary Fig. 26. The EXAFS fits (Fe) for precursor (a-b) and regenerated catalysts (Cycle 5 (c-d), Cycle 15 (e-f) and Cycle 30 (g-h)) in k and R spaces.

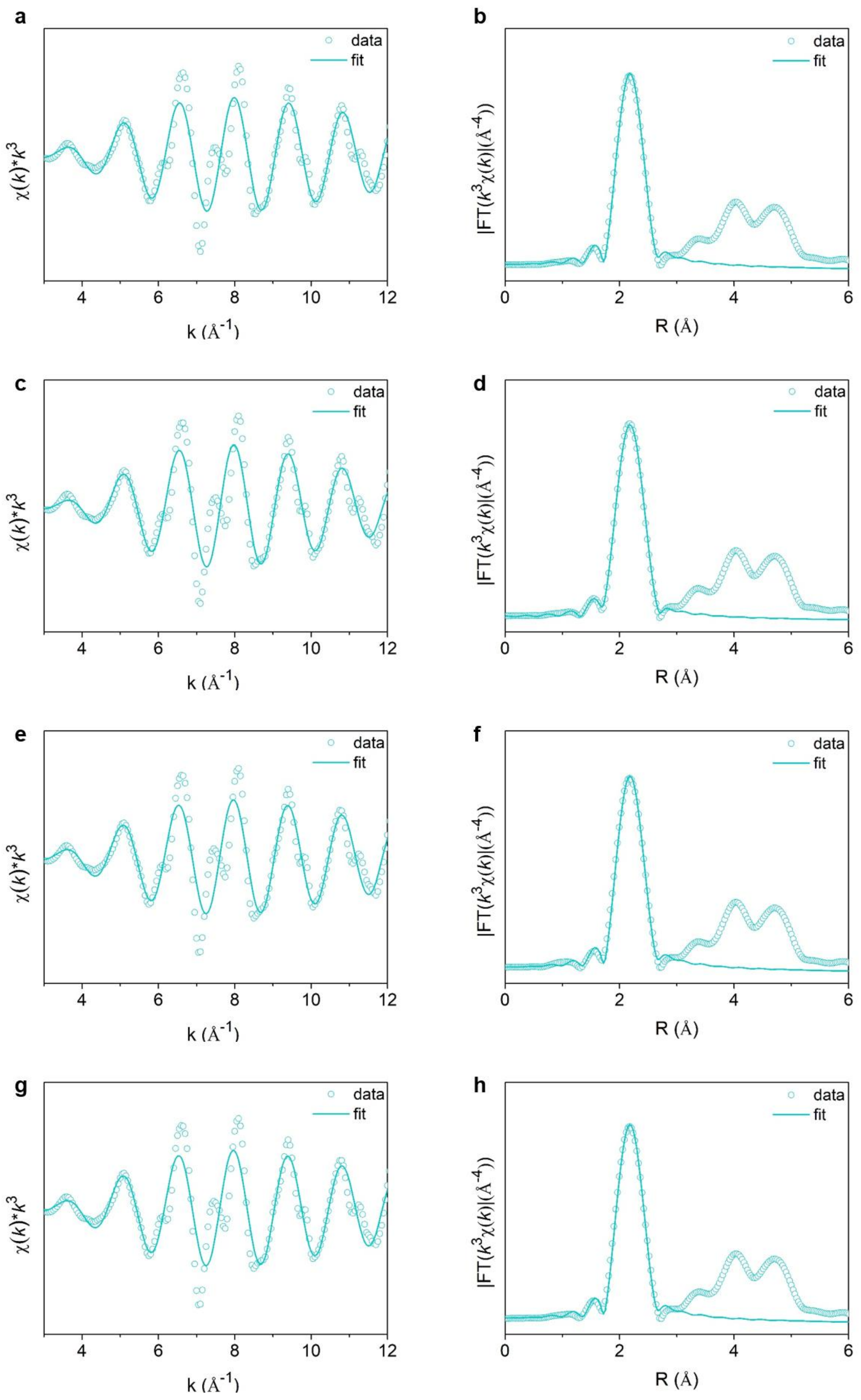

Supplementary Fig. 27. The EXAFS fits (Ni) for precursor (a-b) and regenerated catalysts (Cycle 5 (c-d), Cycle 15 (e-f) and Cycle 30 (g-h)) in k and R spaces.

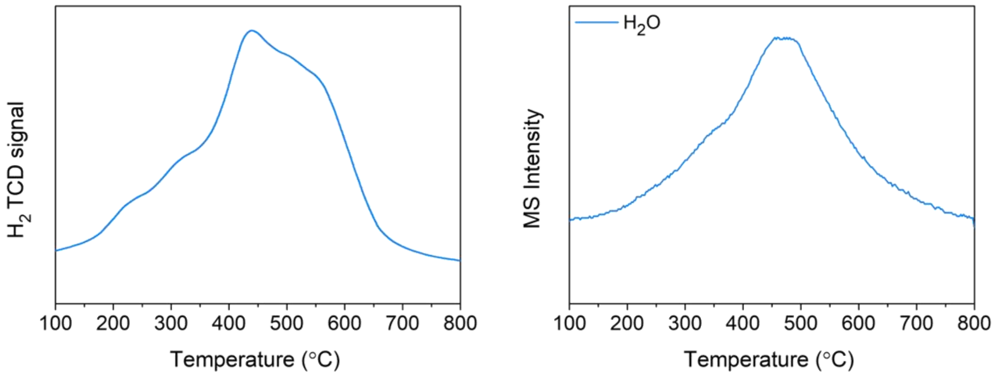

Supplementary Fig. 28. The H_2-TPR-MS results of the FeC-FeNi/Ni/C catalyst precursor.

Supplementary Fig. 28 presents the H_2-TPR-MS results of the FeC-FeNi/Ni/C catalyst precursor, where the TPR profile and mass spectrometry data indicate that this precursor is reduced and water molecules form during hydrogen reduction, supporting hydrogen's role as a reducing medium in the reaction.

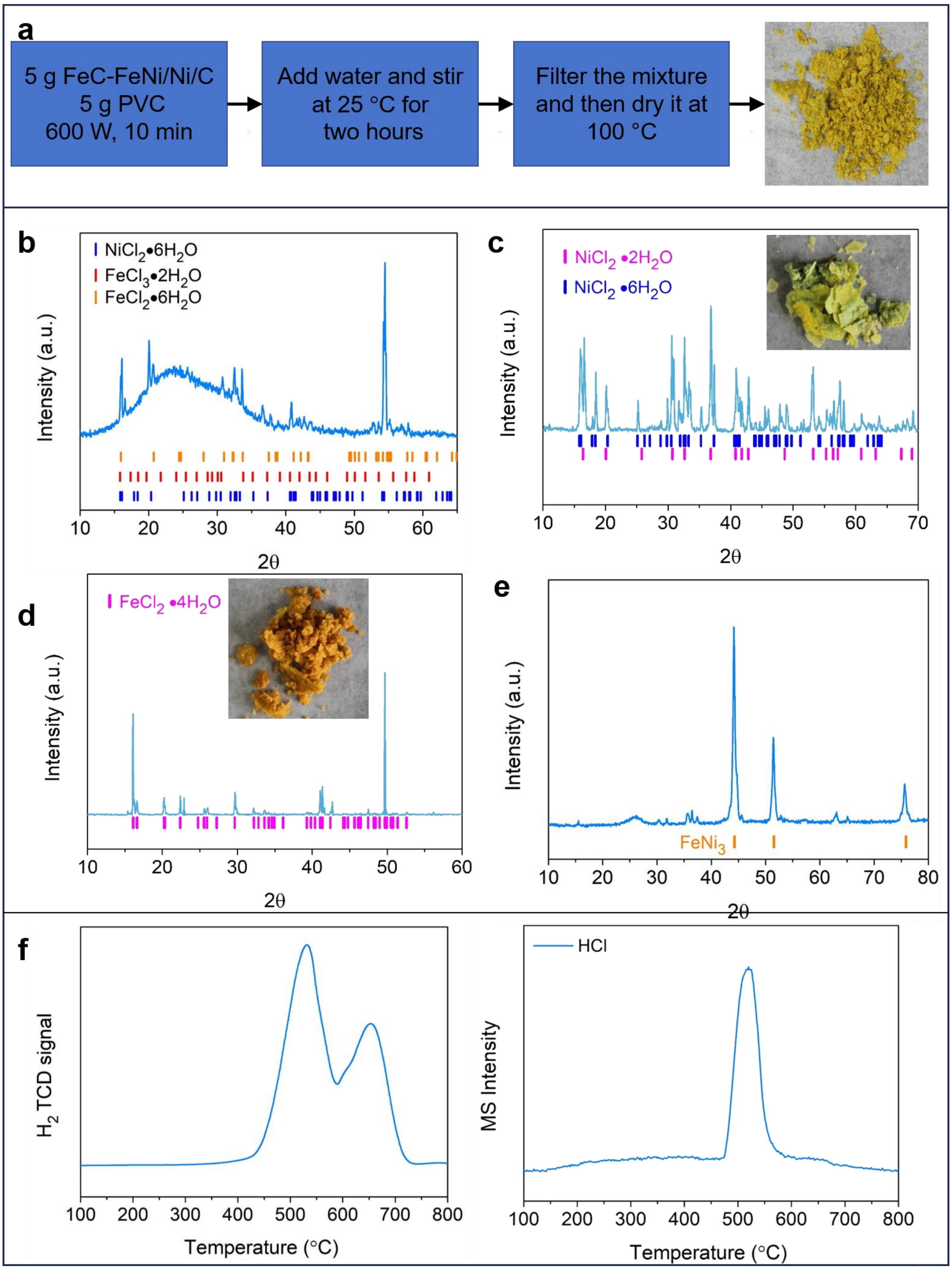

Supplementary Fig. 29. (a) Schematic diagram of FeC-FeNi/Ni/C and PVC reaction and post-treatment. (b) XRD results of the yellow crystals. Optical images and corresponding XRD patterns of the green (c) and yellow (d) crystals. (e) XRD results of the crystals after being treated with H_2. (f) The H_2-TPR-MS results of the yellow crystals.

To further verify that PVC decomposition can promote the formation of iron chlorides and nickel chlorides, the following experiment was conducted: 5 g of FeC-FeNi/Ni/C

and 5 g of PVC were mixed uniformly and reacted under 600 W microwave power for 10 minutes. Subsequently, 30 mL of deionized water was added and stirred at 25 °C for 2 hours. Afterwards, the above mixture was filtered, and the filtrate was dried completely in an oven at 100 °C, ultimately obtaining yellow crystals (Supplementary Fig. 29a). XRD results of the yellow crystals indicate that they are composed of $NiCl_2$ and FeClx. Moreover, to further and more clearly elucidate the formation of iron chloride and nickel chloride compounds, Ni/C and Fe_3C catalysts were synthesized via microwave-assisted methods, then mixed with PVC at a 1:1 mass ratio (5 g each) and reacted under 600 W microwave irradiation for 10 minutes. Next, 30 mL of deionized water was added, and the mixture was stirred at 25 °C for 2 hours. The resulting suspension was filtered, and the filtrate was dried completely in a 100 °C oven, yielding green nickel chloride crystals and yellow ferric chloride crystals, respectively (Supplementary Fig. 29c-d, insert). Subsequent XRD characterization confirms the identity of the green crystals as nickel chloride and yellow crystals as ferric chloride, verifying the formation of these chloride compounds (Supplementary Fig. 29c-d). These results directly demonstrate that PVC decomposition does promote the formation of $NiCl_2$ and FeClx.

To verify that $NiCl_2$ and FeClx can form FeNi alloy under hydrogen, 0.3 g of yellow crystals and 0.1 g of commercial multi-walled carbon nanotubes were mixed uniformly and heated under 600 W microwave power and 10% H_2 atmosphere (flow rate 300 ml/min) for 10 minutes. XRD analysis confirms the formation of FeNi alloy (Supplementary Fig. 29e). Meanwhile, treatment of the obtained yellow crystals by H_2-TPR-MS reveals the production of HCl, which is consistent with the aforementioned de-chlorine process of $NiCl_2$ and FeClx under hydrogen (Supplementary Fig. 29f). These results support the above discussion regarding the PVC-assisted reconstruction of the catalyst to form FeNi alloy.

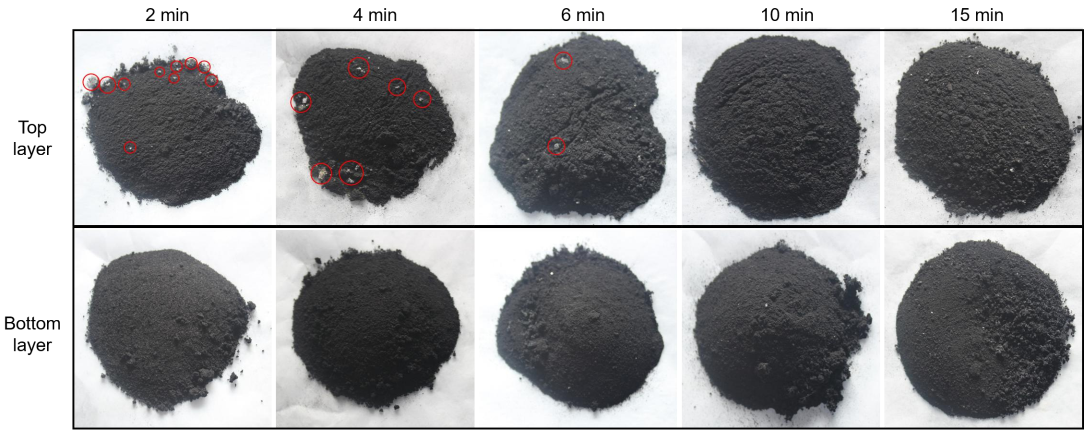

Supplementary Fig. 30. Time-dependent optical images of PVC decomposition catalyzed by FeC-FeNi/Ni/C. The white crystals are metal chlorides, while the black substance is the catalyst.

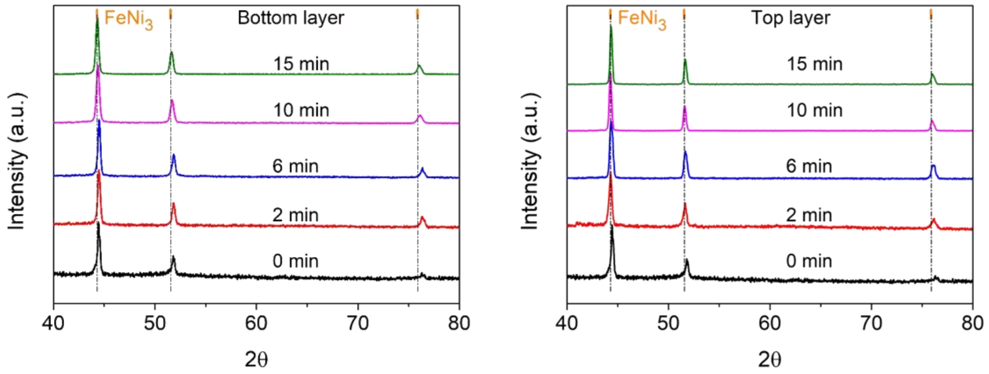

Supplementary Fig. 31. Time-dependent XRD patterns of PVC decomposition catalyzed by FeC-FeNi/Ni/C.

Ex-situ time-dependent optical images and XRD patterns of PVC decomposition catalyzed by FeC-FeNi/Ni/C further reveal details of the FeNi alloy formation process. In this process, Cl generated from PVC decomposition induces the conversion of Fe and Ni into metal chlorides. Subsequently, under the action of hydrogen produced from PVC decomposition, these metal chlorides are reduced and converted into FeNi alloys, thereby completing catalyst reconstruction. This process is manifested in Supplementary Fig. 30 as the gradual exposure of tiny white crystals, which subsequently disappear over time. Simultaneously, the corresponding XRD shows diffraction peaks of the FeNi alloy (Supplementary Fig. 31).

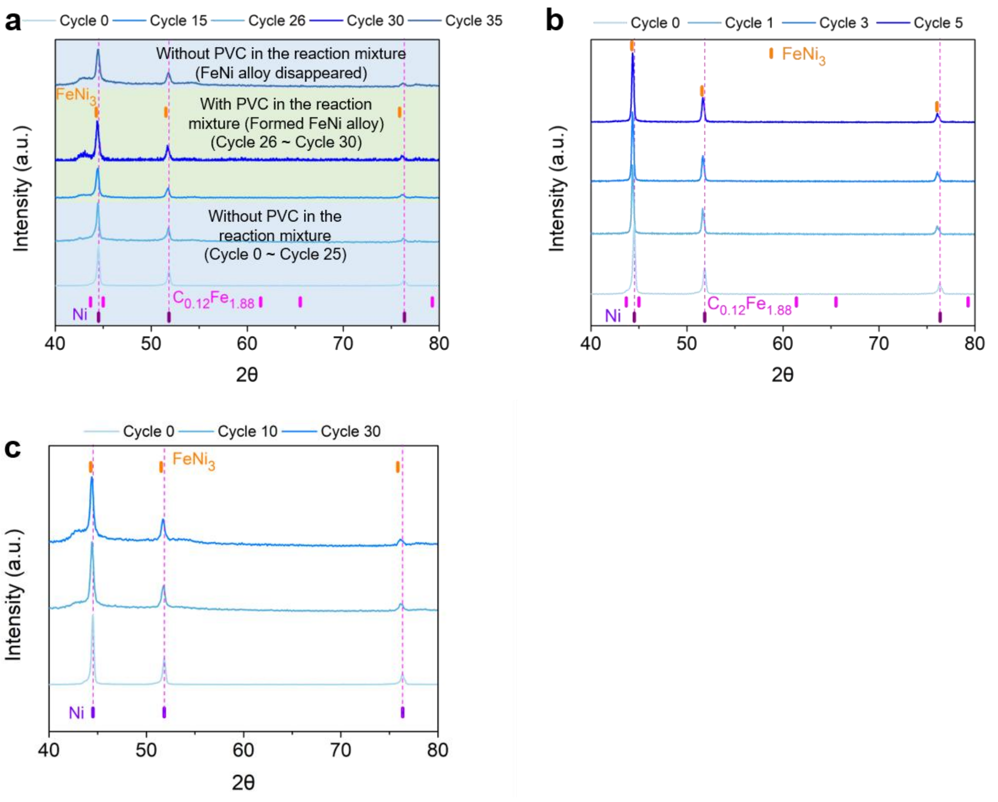

Supplementary Fig. 32. XRD analysis of the catalyst structure in the reaction mixture with and without PVC. (a) Plastic: LDPE (Cycle 1 ~ Cycle 25 and Cycle 31~Cycle 35), PVC (Cycle 26~Cycle 30). (b) Plastic: PVC. PVC promotes NiFe alloy formation. (c) Plastic: LDPE and PVC mixture, with the mass ratio of LDPE to PVC being 9:1. The XRD results show that when catalyzing the decomposition of LDPE and PVC plastic mixture, the catalyst maintains the NiFe alloy state. This further proves that the presence of PVC is helpful for catalyst regeneration, thus significantly prolonging the catalyst stability.

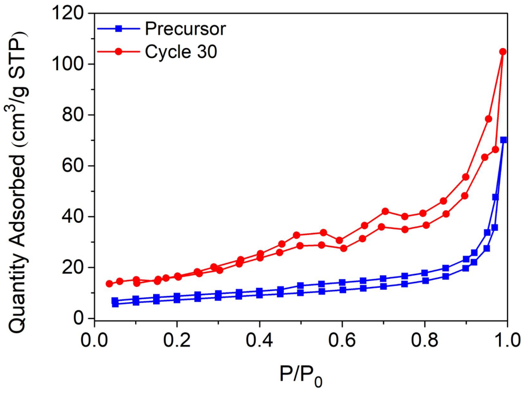

Supplementary Fig. 33. BET patterns of precursor and long-term used (cycle 30) catalysts.

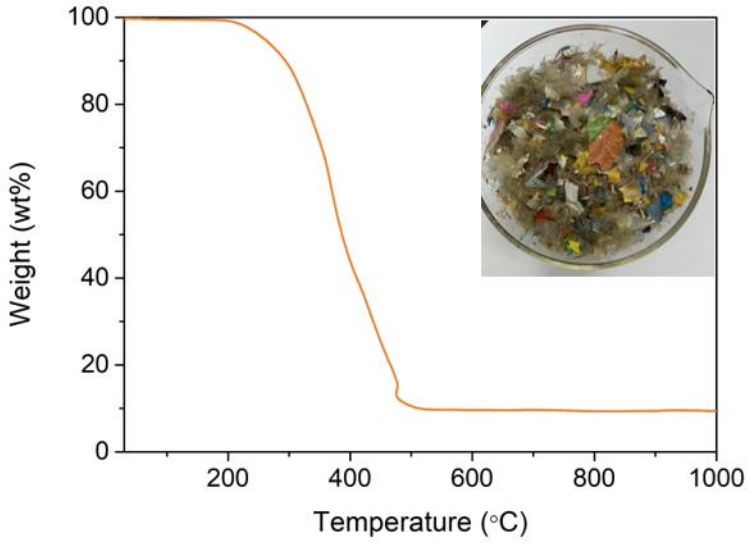

Supplementary Fig. 34. TGA results of landfilled mixed plastics waste and optical image.

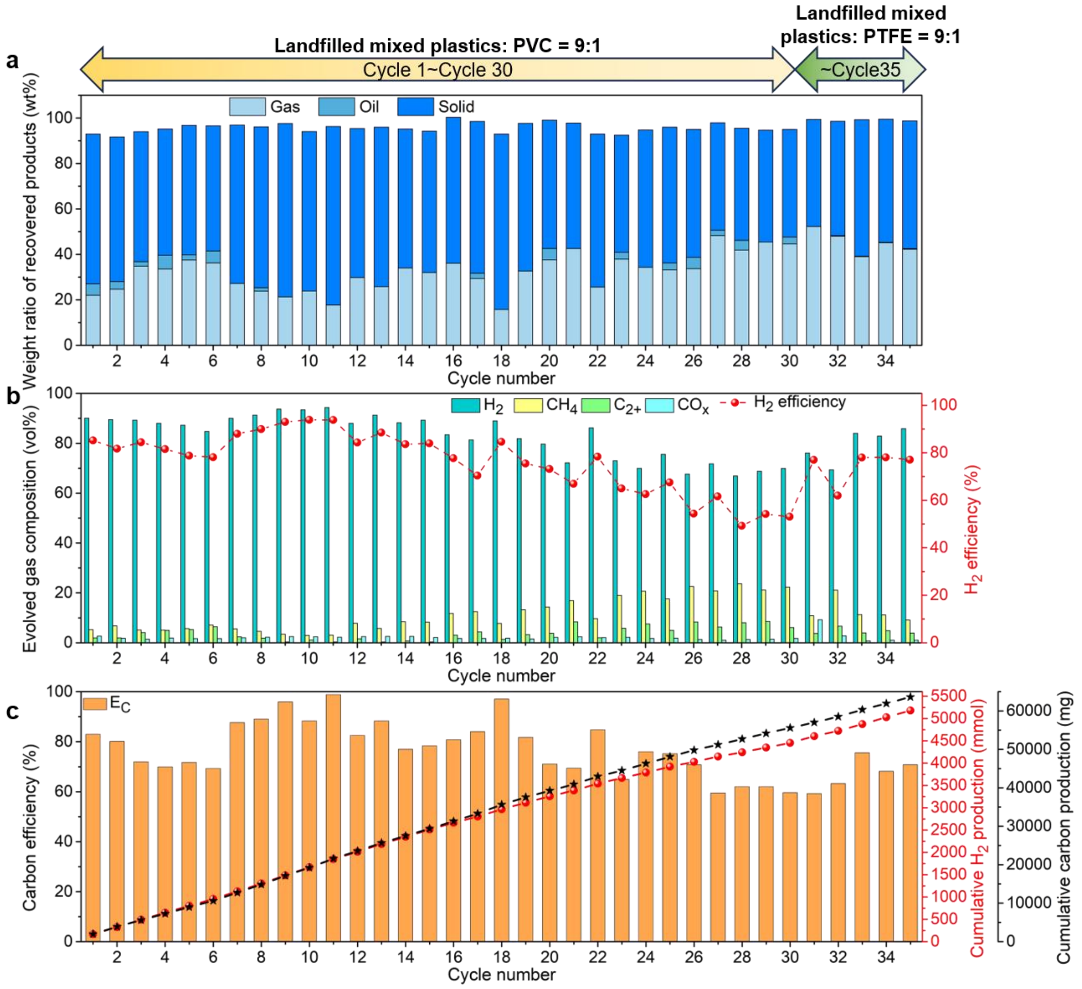

Supplementary Fig. 35. Successive cycles of microwave catalytic decomposition of chlorinated/fluorinated landfilled plastic waste mixtures over FeNi/Ni/C catalyst. (a) The weight ratio of recovered gas, oil and solid. (b) Corresponding evolved gas composition (vol%) and H_2 efficiency (%). (c) Carbon efficiency (%), cumulative H_2 production (mmol) and cumulative carbon production (mg). Reaction conditions: 2.7 g landfilled plastic mixtures, 0.3 g PVC/PTFE and 3 g FeNi/Ni/C catalyst in the bottom layer; 3 g FeNi/Ni/C catalyst in the top layer.

The landfilled plastic mixtures were mechanically pulverized and first mixed with PVC at a mass ratio of 9:1. Then they were evenly mixed with the FeNi/Ni/C catalyst and reacted under 600 W microwave power. In the first 30 successive catalytic decomposition cycles, around 17-45 wt% of the plastic is swiftly converted, with solid carbon products constituting a weight ratio of 50-80 wt% (Supplementary Fig. 35(a) and Supplementary Table 11). In addition, H_2 is still the dominating component in gas products. The purity and efficiency of H_2 produced by the decomposition of PVC-contained plastic mixtures are about 70 vol%-95 vol% and 50%-94% in the 30 successive cycles, respectively, demonstrating a good decomposition performance (Supplementary Fig. 35(b)). Besides, carbon efficiency (60-99%) further reveals that even complex landfilled plastic mixtures can still be steadily upcycled to H_2 and

carbon materials, providing an alternative route for plastic waste recycling (Supplementary Fig. 35(c) and Supplementary Fig. 36).

Fluoride, commonly formed during the decomposition of PTFE (Supplementary Table 12). Therefore, to investigate whether F could function as a catalyst regenerant analogous to Cl, new comparative experiments were performed: 5 g FeC-FeNi/Ni/C were homogenized with 5 g PVC or PTFE, then reacted under 600 W microwave irradiation for 10 min. XRD analysis of the catalyst recovered after catalytic decomposition of PTFE over the FeC-FeNi/Ni/C precursor reveals a primary composition of Ni and NiF_2, as shown in Supplementary Fig. 37a. Peaks at 2θ values of 44.5°, 51.8°, and 76.3° are assigned to metallic Ni, while those at 2θ = 27.1°, 34.8°, 38.6°, 40°, 43.4°, 53°, 55.8°, 59.9°, 63.1°, 66.9°, 67.5°, 70.9°, and 73.6° correspond to NiF_2. However, no iron fluoride compounds are detected in post-reaction catalysts. When Fe_3C was employed as a catalyst for PTFE decomposition at 600 W for 10 minutes, XRD analysis confirmed the catalyst retained its Fe_3C structure, with no iron fluoride formation observed (Supplementary Fig. 37b). Therefore, fluorine in PTFE primarily reacts with nickel.

To further investigate whether PTFE can induce the transformation of FeC-FeNi/Ni/C catalyst precursors into FeNi/Ni/C, analogous to PVC's effect, five consecutive catalytic cycles were performed using FeC-FeNi/Ni/C precursors with an LDPE-PTFE mixture (9:1) as feedstock. Post-reaction XRD analysis suggests PTFE appears to promote FeNi alloy formation similar to PVC (Supplementary Fig. 37c). Taken together, these results indicate fluorine facilitates FeNi alloy formation via the following pathway: during reaction, fluorine from PTFE combines with nickel to form NiF_2, which is then reduced by hydrogen generated from LDPE decomposition, combining with iron to form FeNi alloys. Concurrently, H_2-TPR-MS analysis of the material obtained post-PTFE reaction detects HF formation, which aligns with the aforementioned defluorination process of NiF_2 under a hydrogen atmosphere (Supplementary Fig. 37d**)**. Complemented by TEM observations (Supplementary Fig. 38), fluorine species may enhance catalyst long-term stability through two mechanisms: reconstructing FeNi active sites and shearing CNTs to expose additional catalytic sites[4].

Based on the above discussion, to further extend the catalyst life, the reaction material was changed to landfilled plastic mixtures and PTFE mixtures (mass ratio of 9:1) during cycle 31-cycle 35 for continuous catalytic decomposition. As shown in Supplementary Fig. 35(b-c) and Supplementary Table 11, the decomposition efficiency increased by nearly 1.6 times after replacing PVC with PTFE.

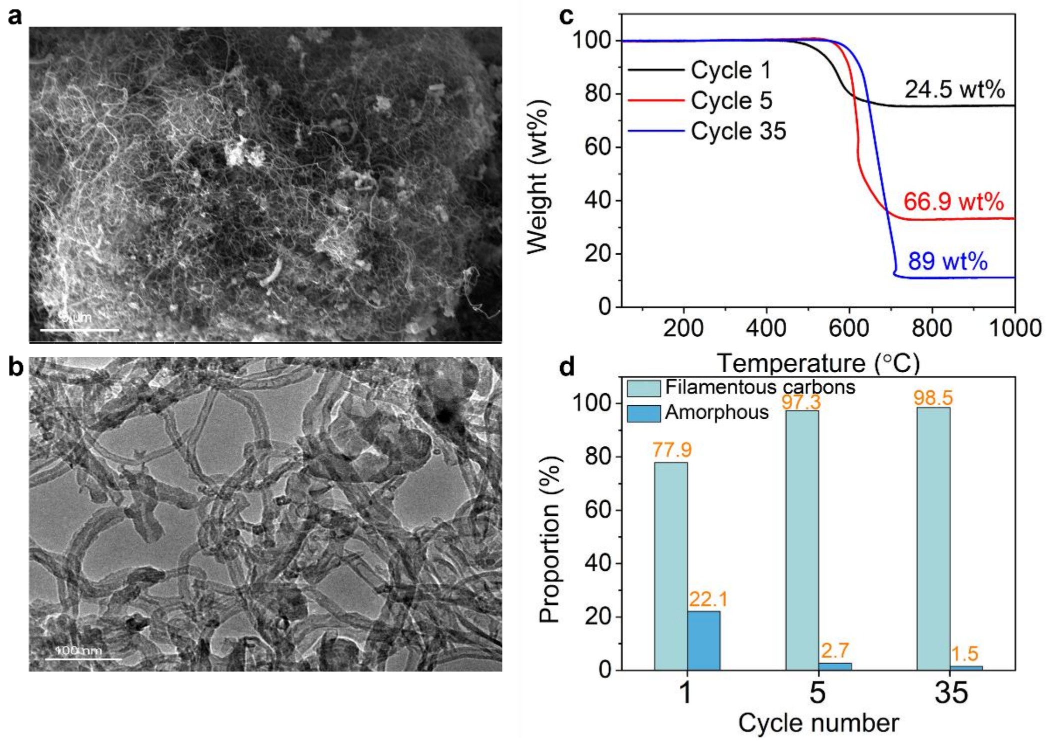

Supplementary Fig. 36. Characterization of the carbon product acquired from the catalytic decomposition of landfilled plastic waste mixtures. (a) SEM image. (b) TEM image. (c) TGA analysis. (d) Proportion of different carbon types in the carbon product.

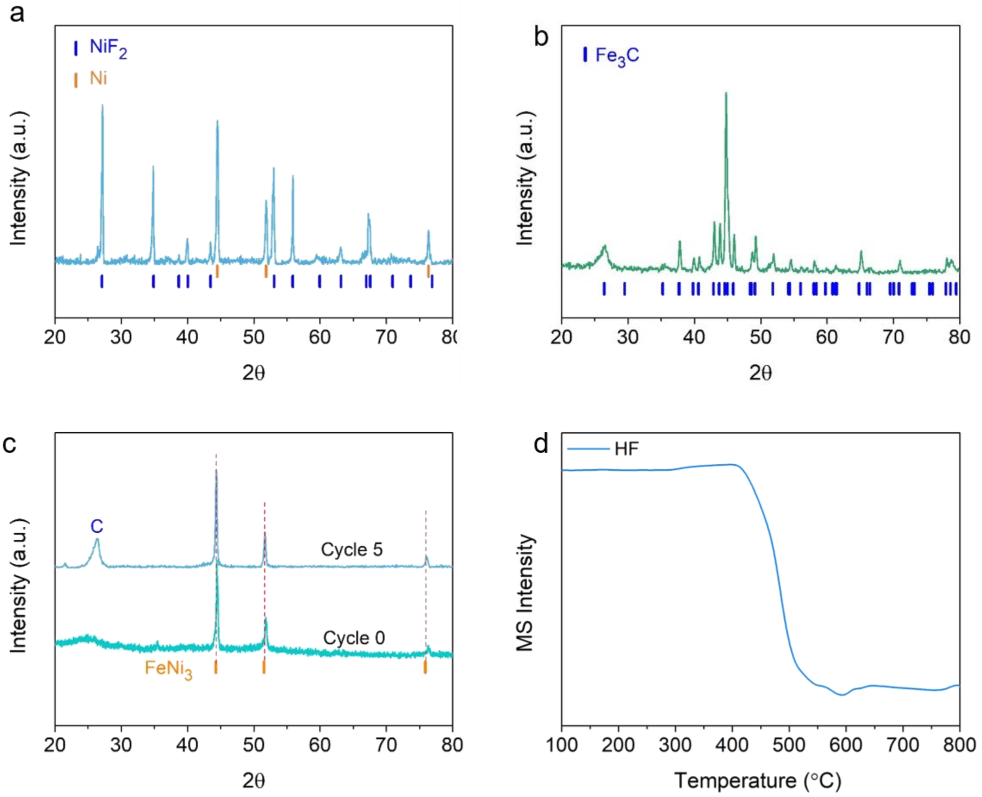

Supplementary Fig. 37. (a) XRD pattern of the sample obtained after catalytic decomposition of PTFE over the FeC-FeNi/Ni/C precursor. (b) XRD pattern of the sample obtained after catalytic decomposition of PTFE over the Fe_3C. (c) XRD patterns of the sample obtained after different cycles of catalytic decomposition of PTFE over the FeC-FeNi/Ni/C precursor. (d) H_2-TPR-MS characterization of the sample obtained after catalytic decomposition of PTFE over the FeC-FeNi/Ni/C precursor.

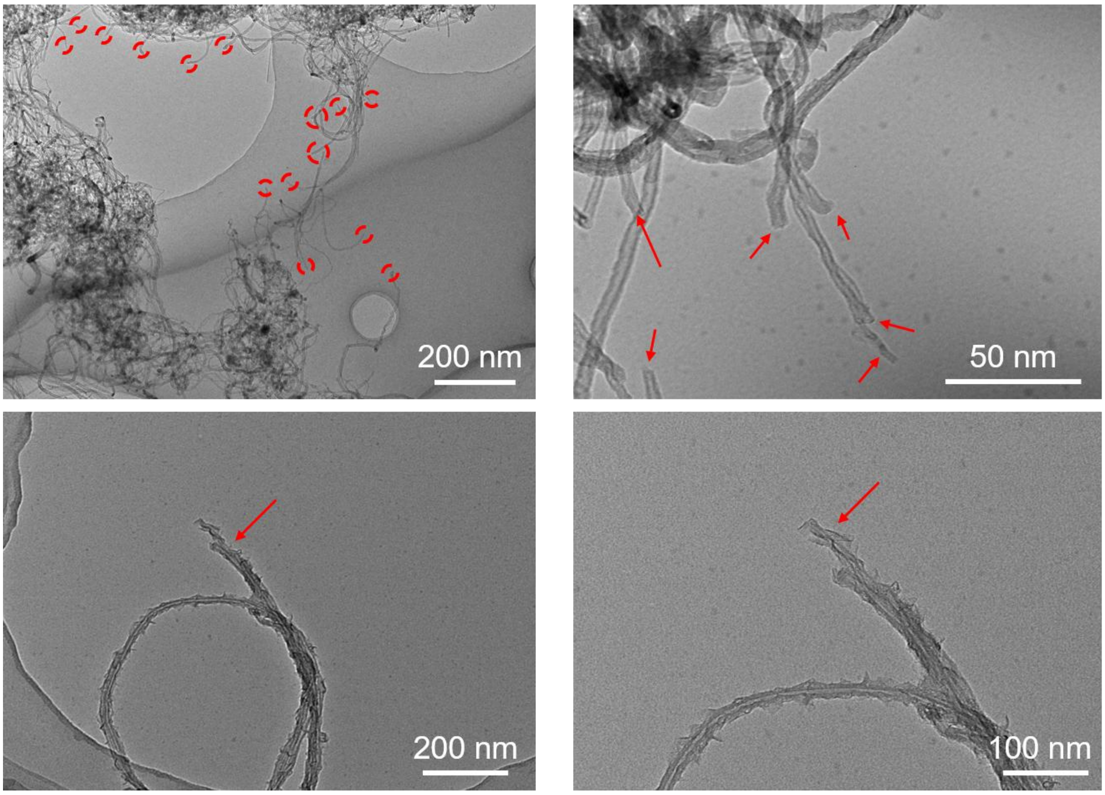

Supplementary Fig. 38. TEM images of the CNTs after reaction with PTFE show that the CNTs have been cut off.

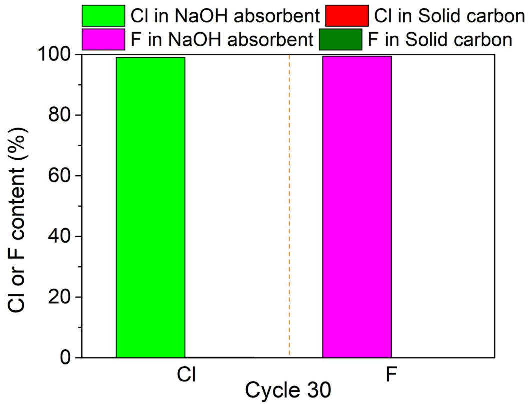

Supplementary Fig. 39. The distribution of Cl and F after the microwave catalytic decomposition of LDPE-HDPE-PP-PS-PVC-PTFE plastic mixtures over the FeNi/Ni/C catalyst during the 30th cycle.

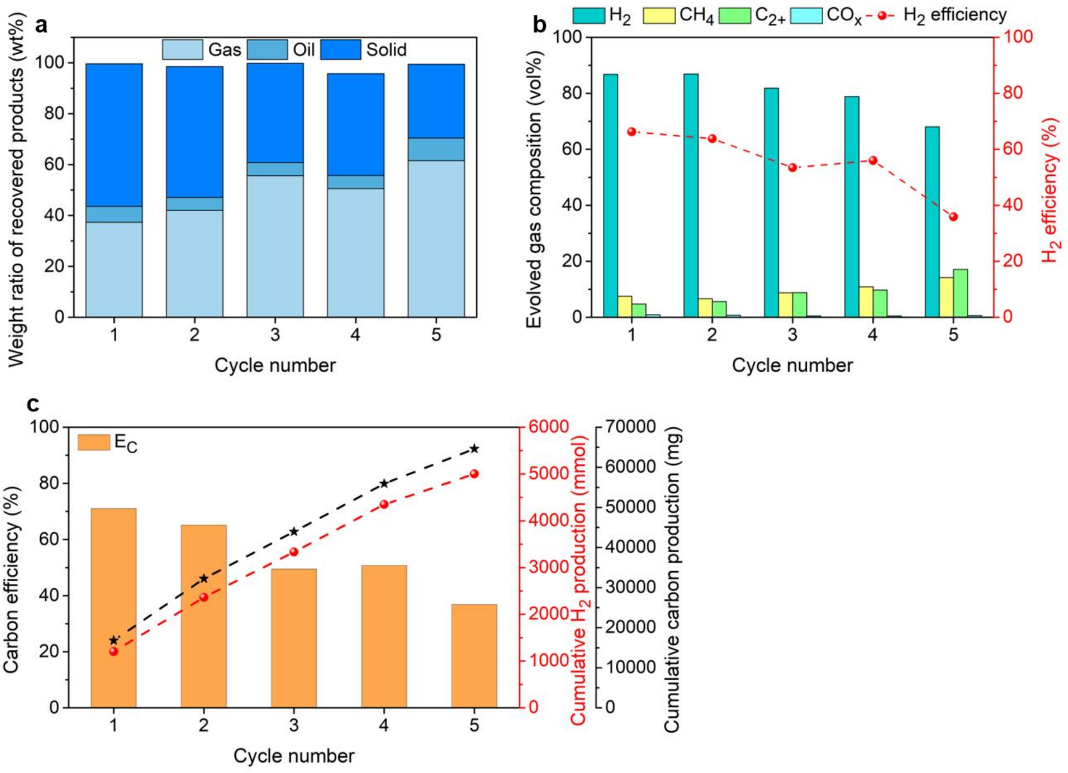

Supplementary Fig. 40. Successive cycles of microwave catalytic decomposition of LDPE, HDPE, PP, PS, PVC and PTFE plastic mixtures (875:615:965:330:510:12) over FeNi/Ni/C catalyst. (**a**) The weight ratio of recovered gas, oil and solid. (**b**) Corresponding evolved gas composition (vol%) and H_2 efficiency (%). (**c**) Carbon efficiency (%), cumulative H_2 production (mmol) and cumulative carbon production (mg). Reaction conditions: 30 g LDPE, HDPE, PP, PS, PVC and PTFE plastic mixtures and 1.5 g FeNi/Ni/C catalyst in the bottom layer; 1.5 g FeNi/Ni/C catalyst in the top layer.

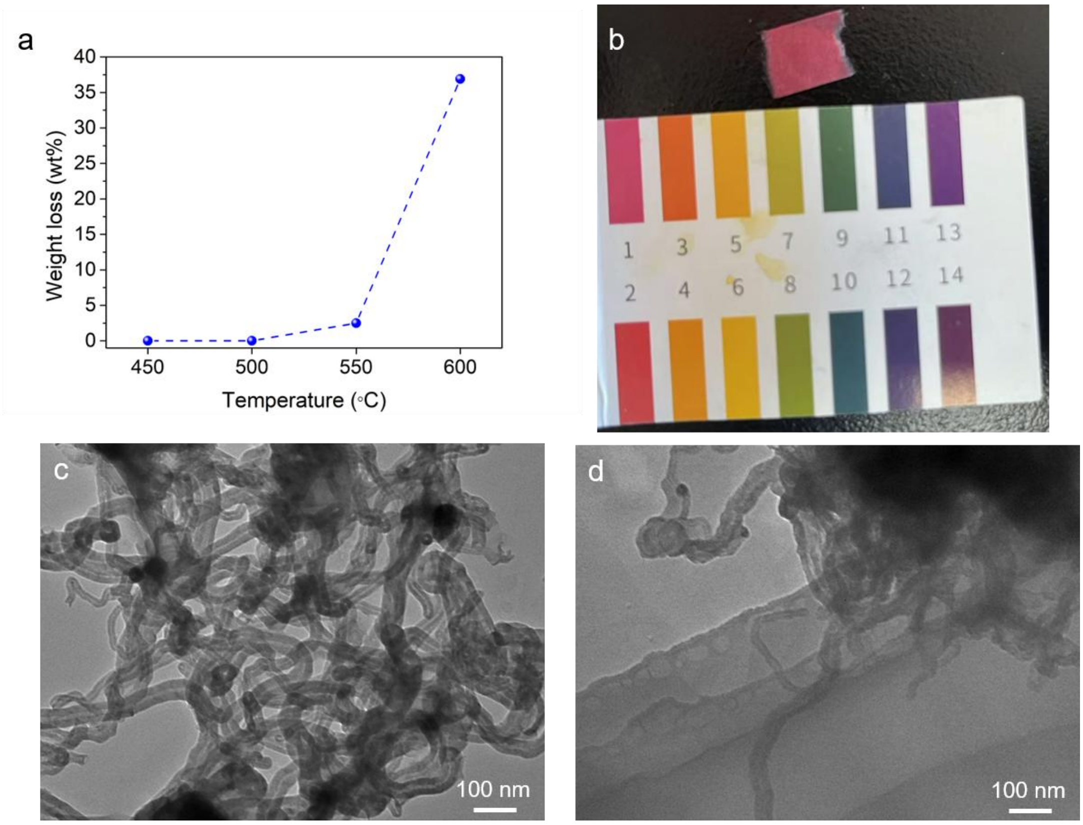

Supplementary Fig. 41. (**a**) CNTs' weight loss (Cycle 30) after being processed in an air atmosphere at different temperatures for 0.5 h (450 °C, 500 °C, 550 °C and 600 °C). (**b**) pH of the aqueous solution (~2.5 mol/L) after Cl and F compounds were absorbed during the reaction. (**c**) TEM images of the prepared CNTs treated in an air atmosphere at 550 °C for 0.5 h and then acid treated at 50 °C for 2 h. (**d**) TEM images of the prepared CNTs treated in an air atmosphere at 600 °C and then acid treated at 50 °C for 2 h.

CNTs production conditions: using FeNi/Ni/C as the catalyst. Feedstock: LDPE and PVC mixture with a mass ratio of 9:1. 3 g plastic mixture and 3 g FeNi/Ni/C catalyst in the bottom layer; 3 g FeNi/Ni/C catalyst in the top layer.

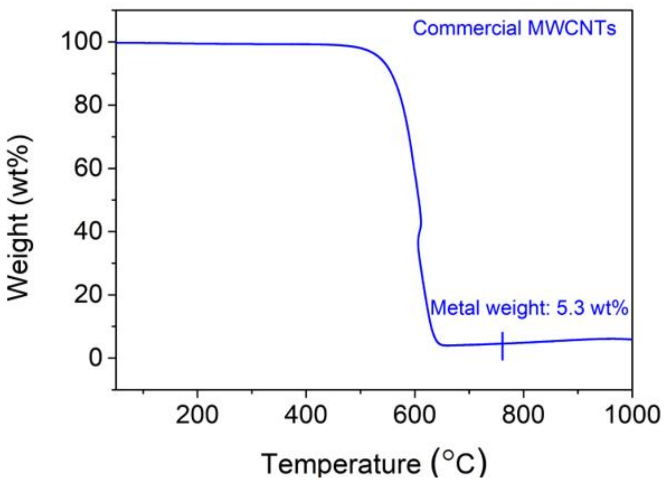

Supplementary Fig. 42. Weight of metals (~5.3 wt%) in the industrial-grade multi-walled carbon nanotubes.

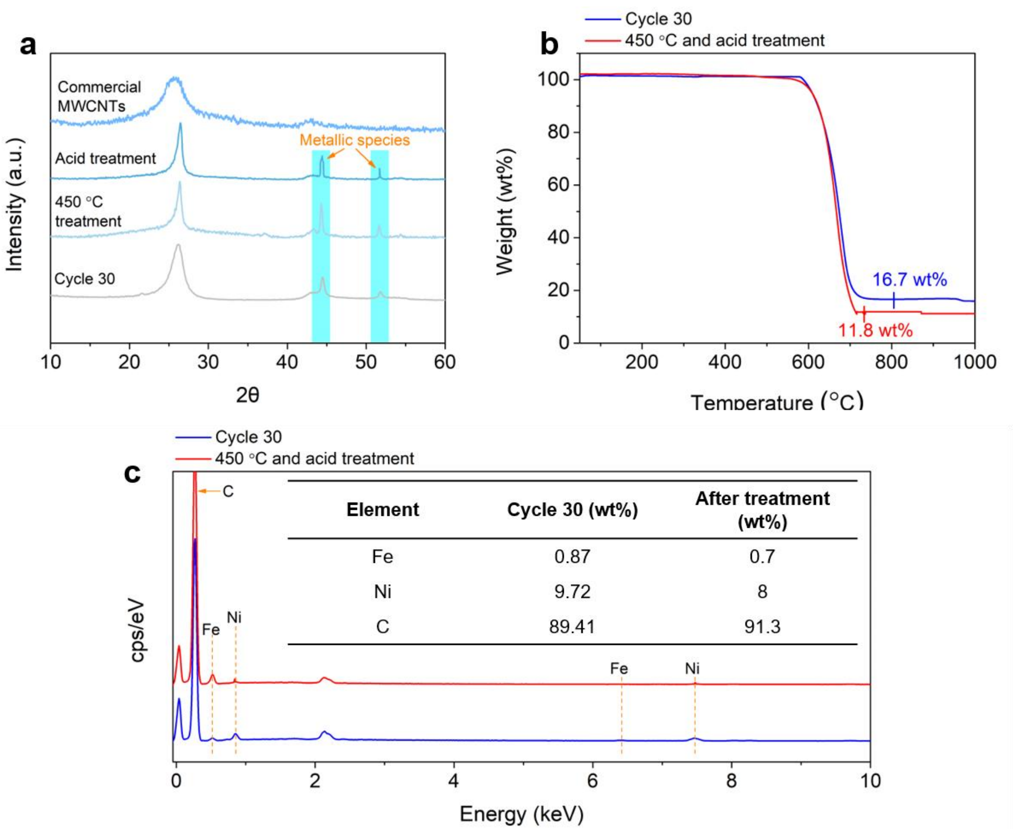

Supplementary Fig. 43. (**a**) XRD patterns of the prepared CNTs (Cycle 30) before and after treatment, with the XRD pattern of industrial-grade multi-walled carbon nanotubes as a reference. (**b**) The metal content of the CNTs before and after treatment is approximately 16.7 wt% and 11.8 wt%, respectively. (**c**) EDX spectra and elemental composition of the prepared CNTs before and after treatment. The produced CNTs were treated in an air atmosphere at 450 °C for 0.5 h and then acid treated (~2.5 mol/L) at 50 °C for 2 h.

CNTs production conditions: using FeNi/Ni/C as the catalyst. Feedstock: LDPE and PVC mixture with a mass ratio of 9:1. 3 g plastic mixture and 3 g FeNi/Ni/C catalyst in the bottom layer; 3 g FeNi/Ni/C catalyst in the top layer.

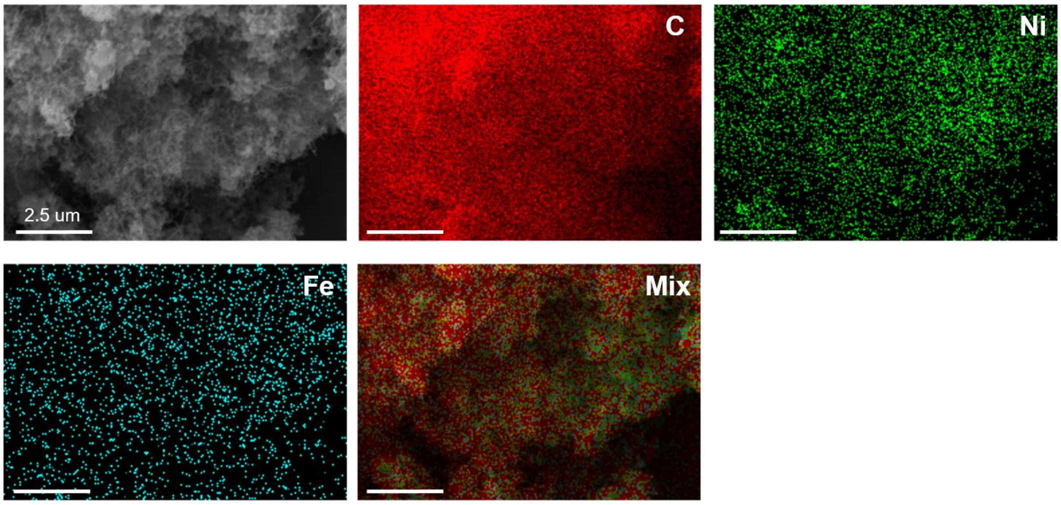

Supplementary Fig. 44. EDX elemental mapping of the obtained CNTs after 30 successive cycles.

CNTs production conditions: using FeNi/Ni/C as the catalyst. Feedstock: LDPE and PVC mixture with a mass ratio of 9:1. 3 g plastic mixture and 3 g FeNi/Ni/C catalyst in the bottom layer; 3 g FeNi/Ni/C catalyst in the top layer.

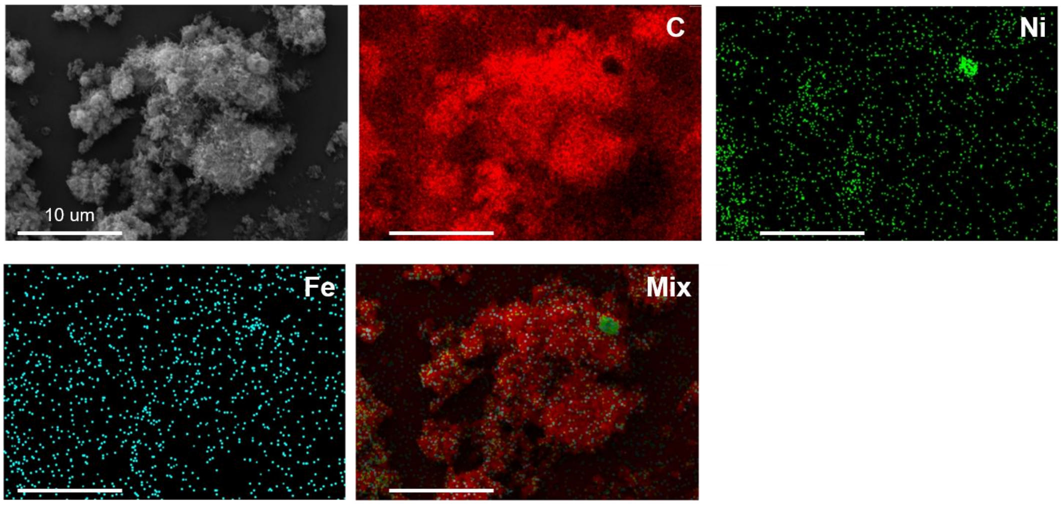

Supplementary Fig. 45. EDX element mapping of the CNTs obtained after 30 successive cycles. The produced CNTs were treated in an air atmosphere at 450 °C for 0.5 h and then acid treated (~2.5 mol/L) at 50 °C for 2 h.

CNTs production conditions: using FeNi/Ni/C as the catalyst. Feedstock: LDPE and PVC mixture with a mass ratio of 9:1. 3 g plastic mixture and 3 g FeNi/Ni/C catalyst in the bottom layer; 3 g FeNi/Ni/C catalyst in the top layer.

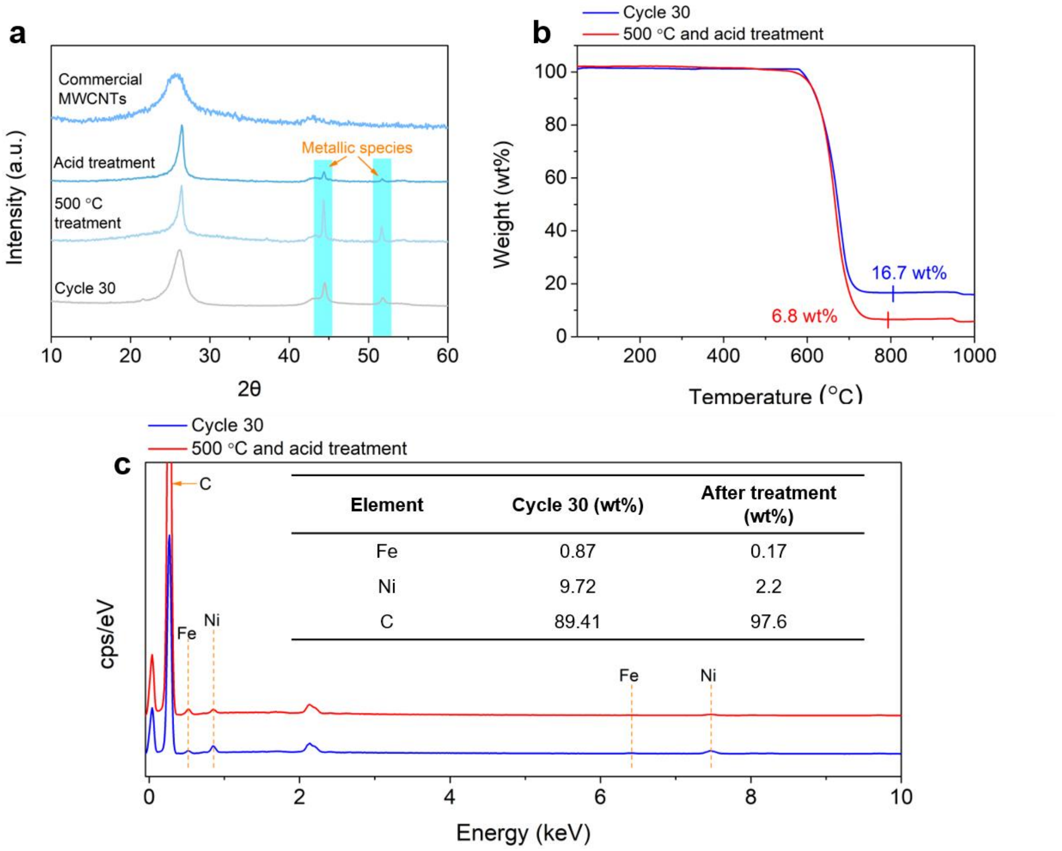

Supplementary Fig. 46. (**a**) XRD patterns of the prepared CNTs (Cycle 30) before and after treatment, with the XRD pattern of industrial-grade multi-walled carbon nanotubes as a reference. (**b**) The metal content of the CNTs before and after treatment is approximately 16.7 wt% and 6.8 wt%, respectively. (**c**) EDX spectra and elemental composition of the prepared CNTs before and after treatment. The produced CNTs were treated in an air atmosphere at 500 °C for 0.5 h and then acid treated (~2.5 mol/L) at 50 °C for 2 h.

CNTs production conditions: using FeNi/Ni/C as the catalyst. Feedstock: LDPE and PVC mixture with a mass ratio of 9:1. 3 g plastic mixture and 3 g FeNi/Ni/C catalyst in the bottom layer; 3 g FeNi/Ni/C catalyst in the top layer.

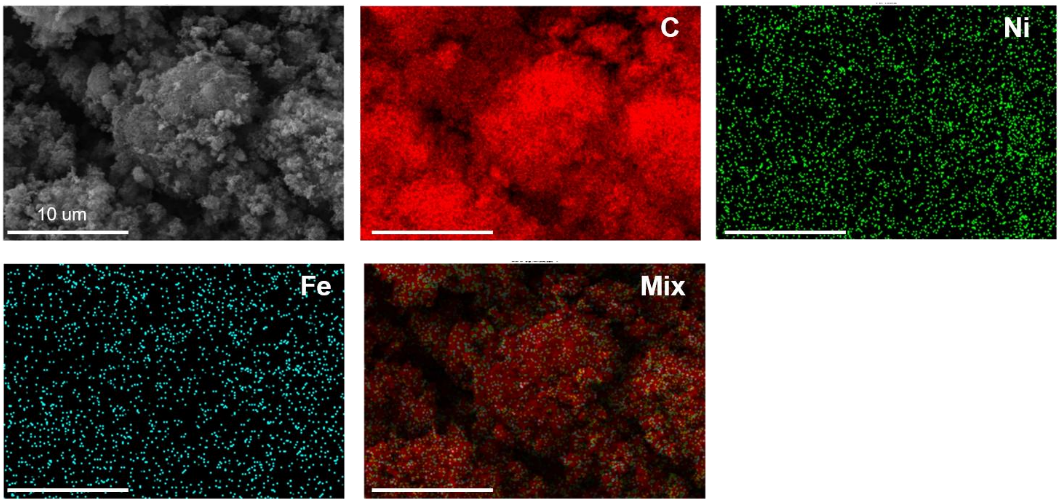

Supplementary Fig. 47. EDX element mapping of the CNTs obtained after 30 successive cycles. The produced CNTs were treated in an air atmosphere at 500 °C for 0.5 h and then acid treated (~2.5 mol/L) at 50 °C for 2 h.

CNTs production conditions: using FeNi/Ni/C as the catalyst. Feedstock: LDPE and PVC mixture with a mass ratio of 9:1. 3 g plastic mixture and 3 g FeNi/Ni/C catalyst in the bottom layer; 3 g FeNi/Ni/C catalyst in the top layer.

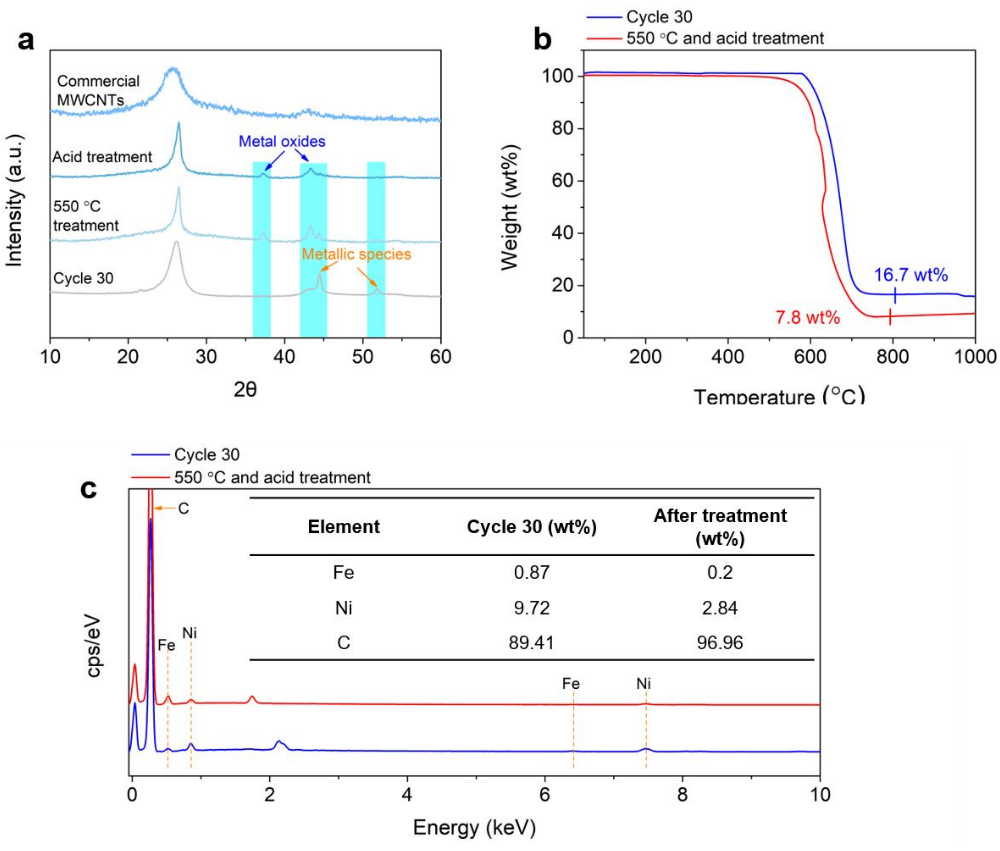

Supplementary Fig. 48. (**a**) XRD patterns of the prepared CNTs (Cycle 30) before and after treatment, with the XRD pattern of industrial-grade multi-walled carbon nanotubes as a reference. (**b**) The metal content of the CNTs before and after treatment is approximately 16.7 wt% and 7.8 wt%, respectively. (**c**) EDX spectra and elemental composition of the prepared CNTs before and after treatment. The produced CNTs were treated in an air atmosphere at 550 °C for 0.5 h and then acid treated (~2.5 mol/L) at 50 °C for 2 h.

CNTs production conditions: using FeNi/Ni/C as the catalyst. Feedstock: LDPE and PVC mixture with a mass ratio of 9:1. 3 g plastic mixture and 3 g FeNi/Ni/C catalyst in the bottom layer; 3 g FeNi/Ni/C catalyst in the top layer.

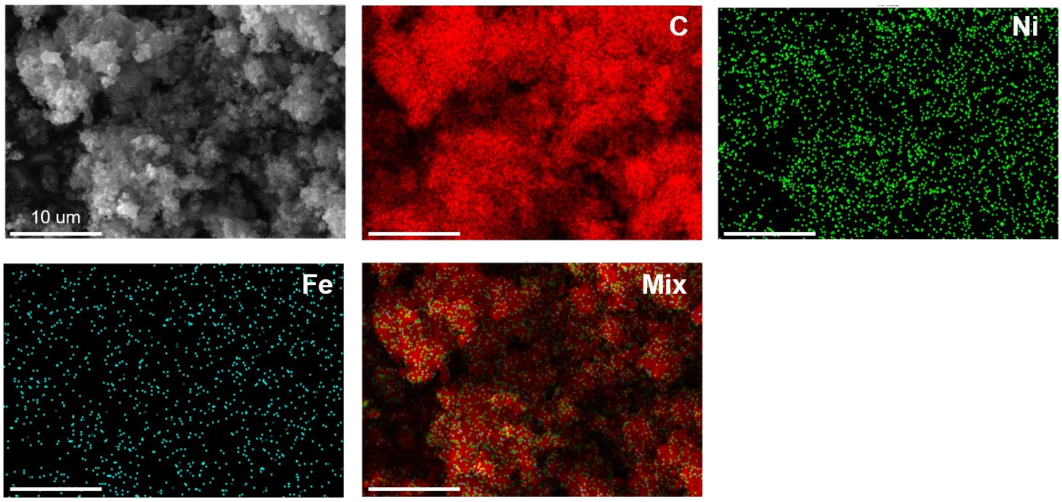

Supplementary Fig. 49. EDX element mapping of the CNTs obtained after 30 successive cycles. The produced CNTs were treated in an air atmosphere at 550 °C for 0.5 h and then acid treated (~2.5 mol/L) at 50 °C for 2 h.

CNTs production conditions: using FeNi/Ni/C as the catalyst. Feedstock: LDPE and PVC mixture with a mass ratio of 9:1. 3 g plastic mixture and 3 g FeNi/Ni/C catalyst in the bottom layer; 3 g FeNi/Ni/C catalyst in the top layer.

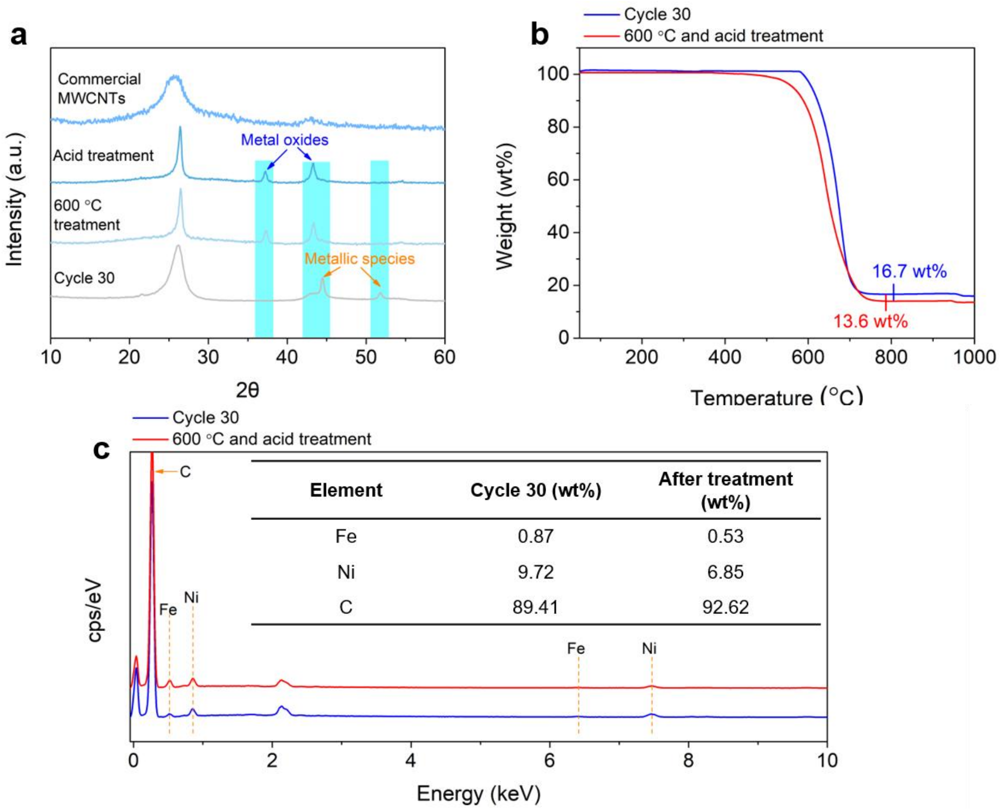

Element	Cycle 30 (wt%)	After treatment (wt%)
Fe	0.87	0.53
Ni	9.72	6.85
C	89.41	92.62

Supplementary Fig. 50. (**a**) XRD patterns of the prepared CNTs (Cycle 30) before and after treatment, with the XRD pattern of industrial-grade multi-walled carbon nanotubes as a reference. (**b**) The metal content of the CNTs before and after treatment is approximately 16.7 wt% and 13.6 wt%, respectively. (**c**) EDX spectra and elemental composition of the prepared CNTs before and after treatment. The produced CNTs were treated in an air atmosphere at 600 °C for 0.5 h and then acid treated (~2.5 mol/L) at 50 °C for 2 h.

CNTs production conditions: using FeNi/Ni/C as the catalyst. Feedstock: LDPE and PVC mixture with a mass ratio of 9:1. 3 g plastic mixture and 3 g FeNi/Ni/C catalyst in the bottom layer; 3 g FeNi/Ni/C catalyst in the top layer.

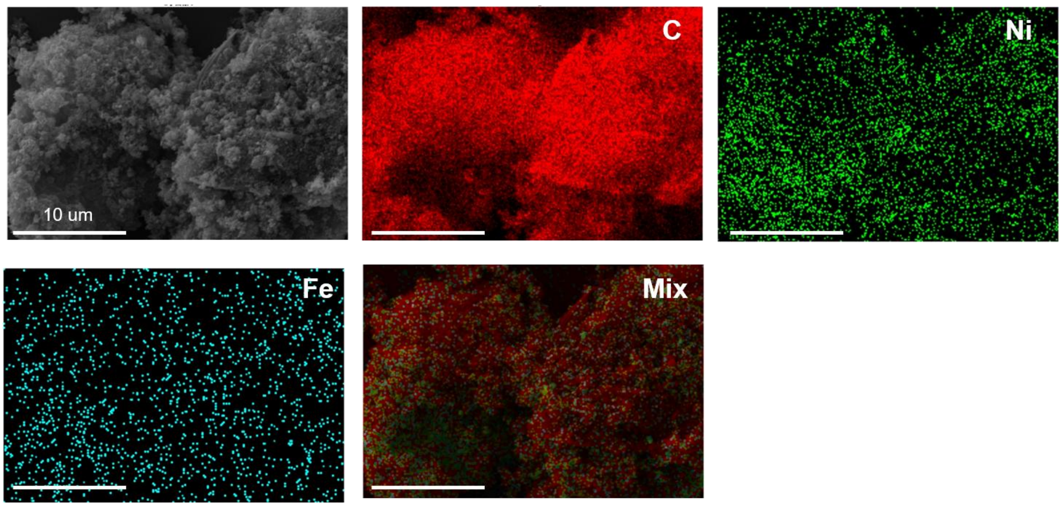

Supplementary Fig. 51. EDX element mapping of the CNTs obtained after 30 successive cycles. The produced CNTs were treated in an air atmosphere at 600 °C for 0.5 h and then acid treated (~2.5 mol/L) at 50 °C for 2 h.

CNTs production conditions: using FeNi/Ni/C as the catalyst. Feedstock: LDPE and PVC mixture with a mass ratio of 9:1. 3 g plastic mixture and 3 g FeNi/Ni/C catalyst in the bottom layer; 3 g FeNi/Ni/C catalyst in the top layer.

To further purify the resulting CNTs, they were pretreated at different temperatures (450 °C, 500 °C, 550 °C and 600 °C) in an air atmosphere to expose the embedded metal catalysts and facilitate the subsequent acid dissolution process. Supplementary Fig. 41a shows that no significant mass loss is observed when the CNTs are processed at 450 °C or 500 °C, which is insufficient for exposing the metal species. When the temperature is increased to 550 °C, a mass loss of 2.5 wt% is observed, indicating the metal species are likely to be successfully exposed. However, at 600 °C, the mass loss rises sharply to 37 wt%, suggesting excessive oxidation that may damage the CNT structure. Meanwhile, although 600 °C also oxidizes metal species to metal oxides, it is difficult to remove them by acid treatment, which may be due to the sintering of metal species caused by high processing temperatures. Therefore, a treatment temperature of 550 °C is determined to be optimal as it balances metal exposure and preserves the CNT structure, as illustrated in Supplementary Fig. 41 and Supplementary Figs. 42-51.

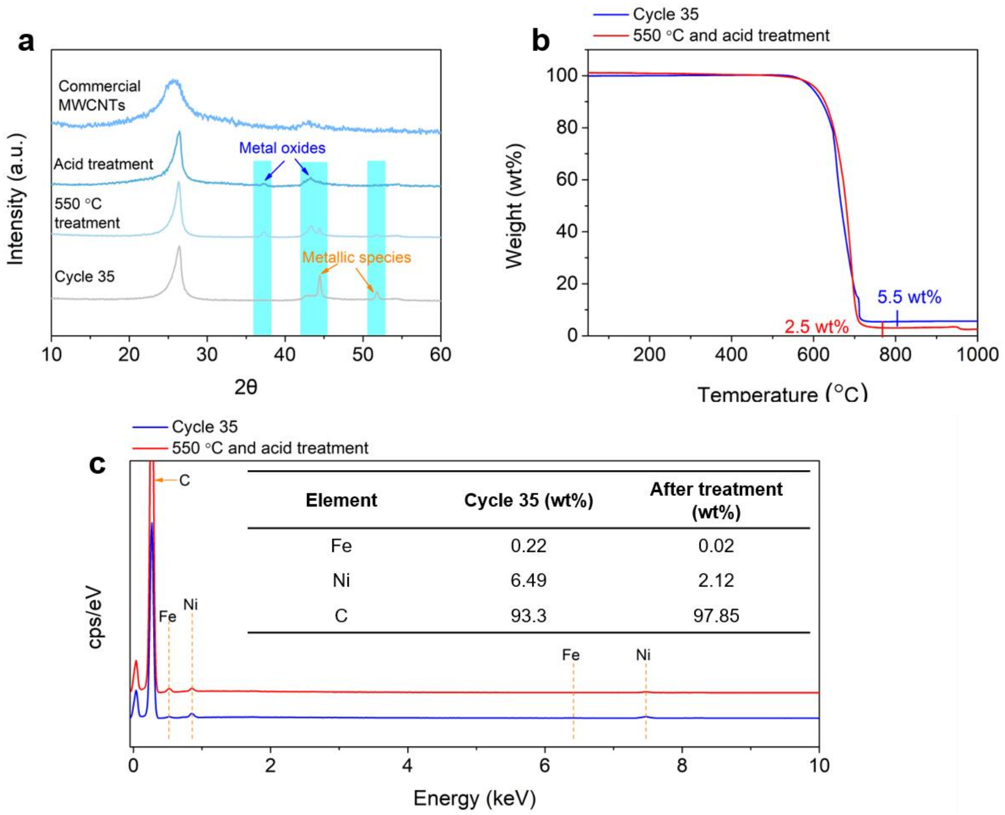

Supplementary Fig. 52. (**a**) XRD patterns of the prepared CNTs (Cycle 35) before and after treatment, with the XRD pattern of industrial-grade multi-walled carbon nanotubes as a reference. (**b**) The metal content of the CNTs before and after treatment is approximately 5.5 wt% and 2.5 wt%, respectively. (**c**) EDX spectra and elemental composition of the prepared CNTs before and after treatment. The produced CNTs were treated in an air atmosphere at 550 °C for 0.5 h and then acid treated (~2.5 mol/L) at 50 °C for 2 h.

CNTs production conditions: using FeNi/Ni/C as the catalyst. Feedstock: LDPE (Cycle 1~Cycle 25, Cycle 31~Cycle 35), PVC (Cycle 26~Cycle 30). 3 g plastic and 3 g FeNi/Ni/C catalyst in the bottom layer; 3 g FeNi/Ni/C catalyst in the top layer.

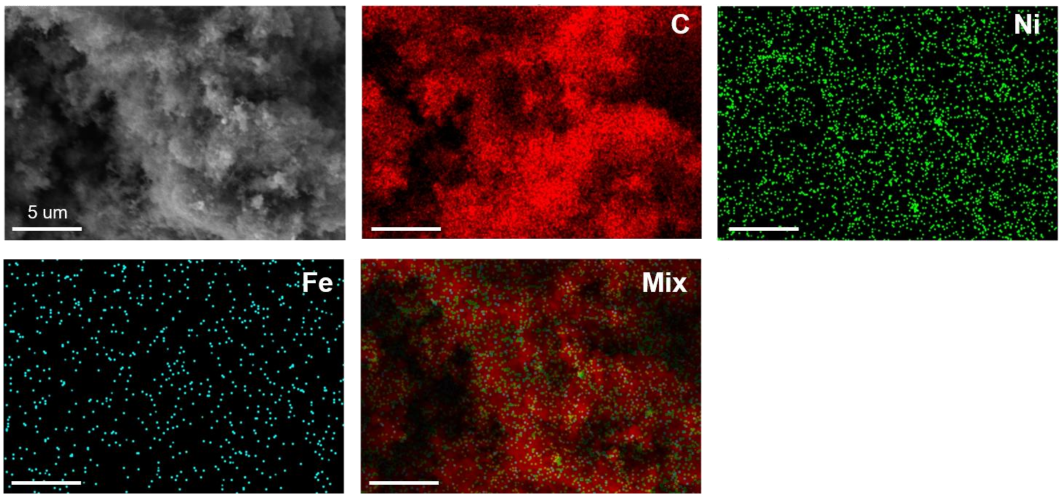

Supplementary Fig. 53. EDX elemental mapping of the obtained CNTs after 35 successive cycles.

CNTs production conditions: using FeNi/Ni/C as the catalyst. Feedstock: LDPE (Cycle 1~Cycle 25, Cycle 31~Cycle 35), PVC (Cycle 26~Cycle 30). 3 g plastic and 3 g FeNi/Ni/C catalyst in the bottom layer; 3 g FeNi/Ni/C catalyst in the top layer.

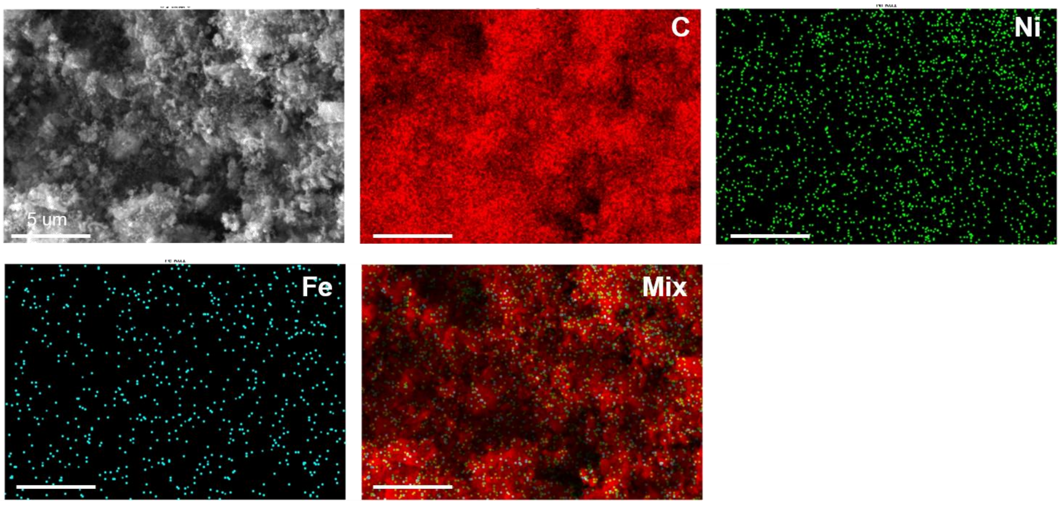

Supplementary Fig. 54. EDX element mapping of the CNTs obtained after 35 successive cycles. The produced CNTs were treated in an air atmosphere at 550 °C for 0.5 h and then acid treated (~2.5 mol/L) at 50 °C for 2 h.

CNTs production conditions: using FeNi/Ni/C as the catalyst. Feedstock: LDPE (Cycle 1~Cycle 25, Cycle 31~Cycle 35), PVC (Cycle 26~Cycle 30). 3 g plastic and 3 g FeNi/Ni/C catalyst in the bottom layer; 3 g FeNi/Ni/C catalyst in the top layer.

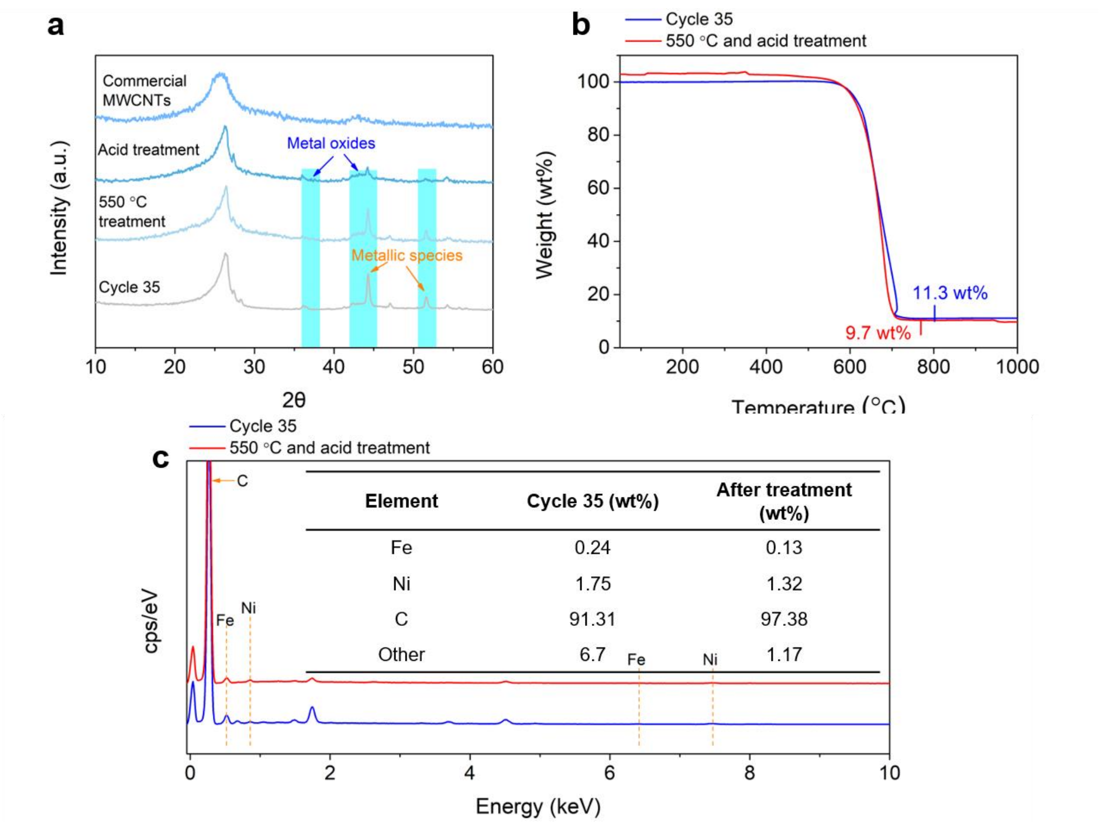

Supplementary Fig. 55. (**a**) XRD patterns of the prepared CNTs (Cycle 35) before and after treatment, with the XRD pattern of industrial-grade multi-walled carbon nanotubes as a reference. (**b**) The metal content of the CNTs before and after treatment is approximately 11.3 wt% and 9.7 wt%, respectively. (**c**) EDX spectra and elemental composition of the prepared CNTs before and after treatment. The produced CNTs were treated in an air atmosphere at 550 °C for 0.5 h and then acid treated (~2.5 mol/L) at 50 °C for 2 h.

CNTs production conditions: using FeNi/Ni/C as the catalyst. Feedstock: PVC and landfilled plastic waste mixtures with a mass ratio of 1:9 (cycle 1 - cycle 30), PTFE and landfilled plastic waste mixtures with a mass ratio of 1:9 (cycle 31 - cycle 35). 3 g plastic mixtures and 3 g FeNi/Ni/C catalyst in the bottom layer; 3 g FeNi/Ni/C catalyst in the top layer.

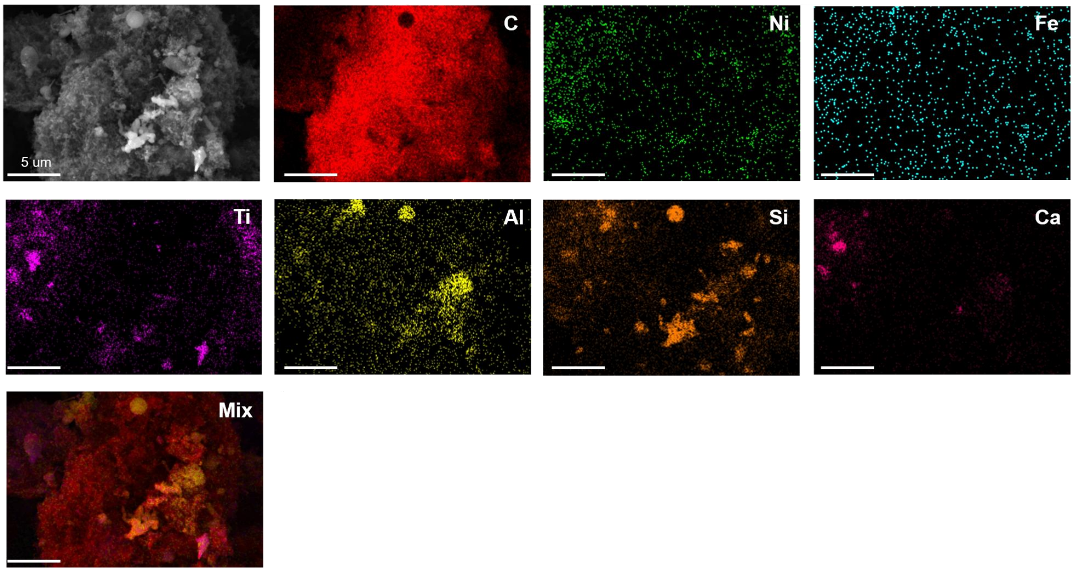

Supplementary Fig. 56. EDX elemental mapping of the obtained CNTs after 35 successive cycles.

CNTs production conditions: using FeNi/Ni/C as the catalyst. Feedstock: PVC and landfilled plastic waste mixtures with a mass ratio of 1:9 (cycle 1 - cycle 30), PTFE and landfilled plastic waste mixtures with a mass ratio of 1:9 (cycle 31 - cycle 35). 3 g plastic mixtures and 3 g FeNi/Ni/C catalyst in the bottom layer; 3 g FeNi/Ni/C catalyst in the top layer.

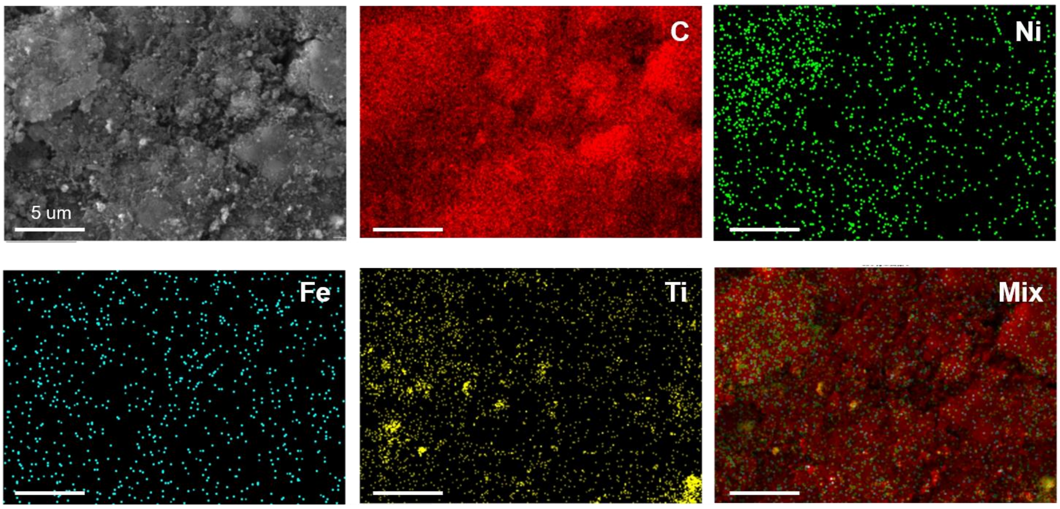

Supplementary Fig. 57. EDX element mapping of the CNTs obtained after 35 successive cycles. The produced CNTs were treated in an air atmosphere at 550 °C for 0.5 h and then acid treated (~2.5 mol/L) at 50 °C for 2 h.

CNTs production conditions: using FeNi/Ni/C as the catalyst. Feedstock: PVC and landfilled plastic waste mixtures with a mass ratio of 1:9 (cycle 1 - cycle 30), PTFE and landfilled plastic waste mixtures with a mass ratio of 1:9 (cycle 31 - cycle 35). 3 g plastic mixtures and 3 g FeNi/Ni/C catalyst in the bottom layer; 3 g FeNi/Ni/C catalyst in the top layer.

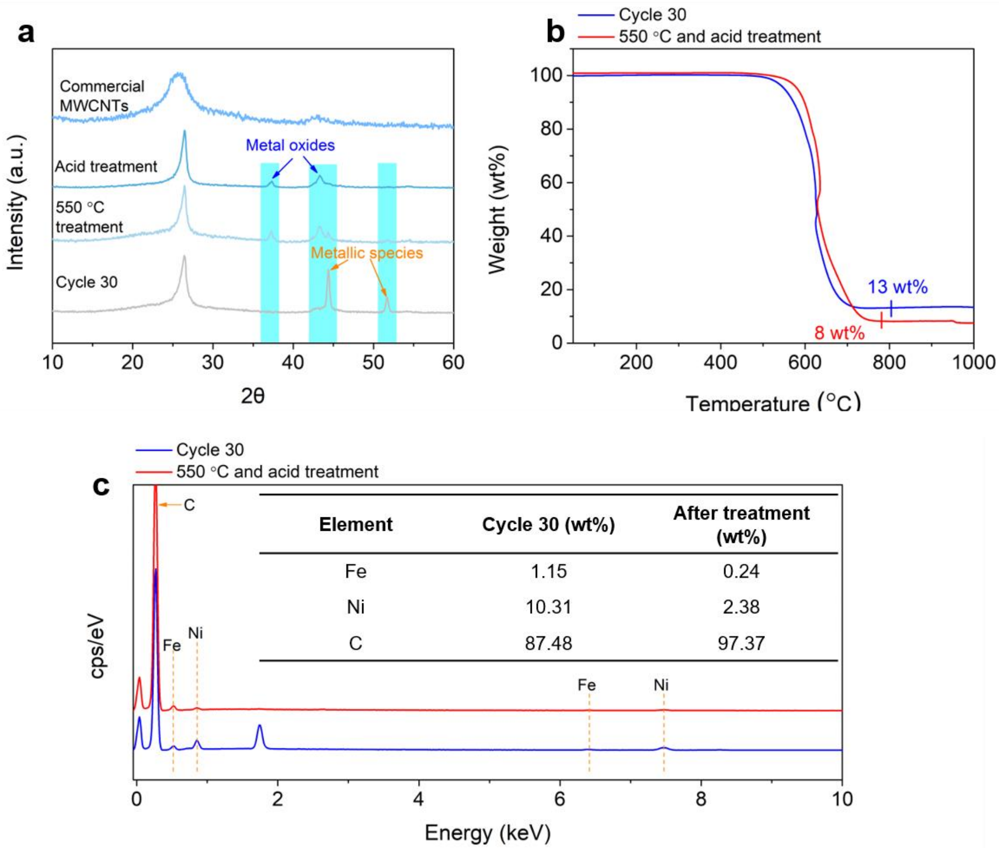

Supplementary Fig. 58. (**a**) XRD patterns of the prepared CNTs (Cycle 30) before and after treatment, with the XRD pattern of industrial-grade multi-walled carbon nanotubes as a reference. (**b**) The metal content of the CNTs before and after treatment is approximately 13 wt% and 8 wt%, respectively. (**c**) EDX spectra and elemental composition of the prepared CNTs before and after treatment. The produced CNTs were treated in an air atmosphere at 550 °C for 0.5 h and then acid treated (~2.5 mol/L) at 50 °C for 2 h.

CNTs production conditions: using FeNi/Ni/C as the catalyst. Feedstock: plastic mixtures of LDPE, HDPE, PP, PS, PVC and PTFE with mass ratios of 15:7:2:5:7:5 (cycle 1 - cycle 5), 40:30:40:20:5:1 (cycle 6 - cycle 10), 2:6:4:6:4:1 (cycle 11 - cycle 15), 10:10:3:10:5:5 (cycle 16 - cycle 20), 5:10:3:15:10:3 (cycle 21 - cycle 25) and 5:2:15:5:5:1 (cycle 26 - cycle 30). 3 g LDPE, HDPE, PP, PS, PVC and PTFE plastic mixtures and 3 g FeNi/Ni/C catalyst in the bottom layer; 3 g FeNi/Ni/C catalyst in the top layer.

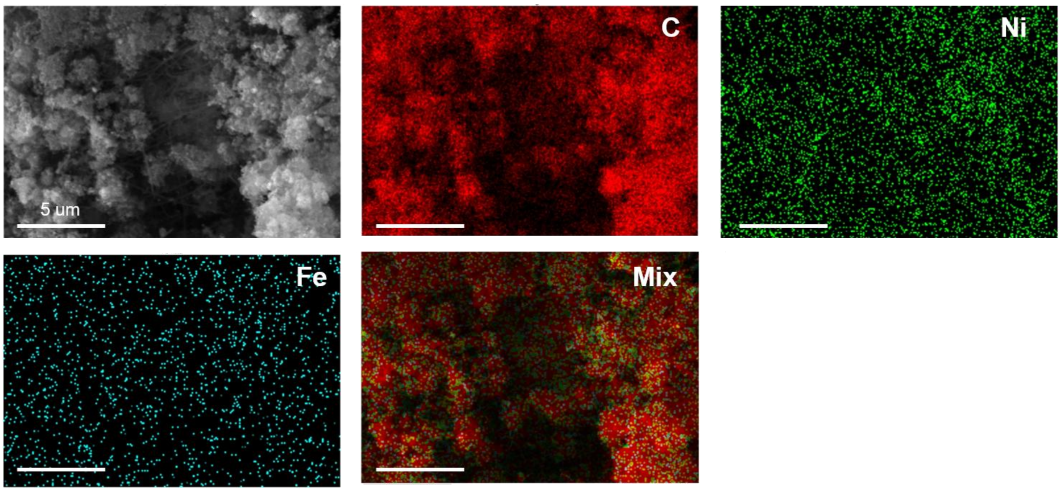

Supplementary Fig. 59. EDX elemental mapping of the obtained CNTs after 30 successive cycles.

CNTs production conditions: using FeNi/Ni/C as the catalyst. Feedstock: plastic mixtures of LDPE, HDPE, PP, PS, PVC and PTFE with mass ratios of 15:7:2:5:7:5 (cycle 1 - cycle 5), 40:30:40:20:5:1 (cycle 6 - cycle 10), 2:6:4:6:4:1 (cycle 11 - cycle 15), 10:10:3:10:5:5 (cycle 16 - cycle 20), 5:10:3:15:10:3 (cycle 21 - cycle 25) and 5:2:15:5:5:1 (cycle 26 - cycle 30). 3 g LDPE, HDPE, PP, PS, PVC and PTFE plastic mixtures and 3 g FeNi/Ni/C catalyst in the bottom layer; 3 g FeNi/Ni/C catalyst in the top layer.

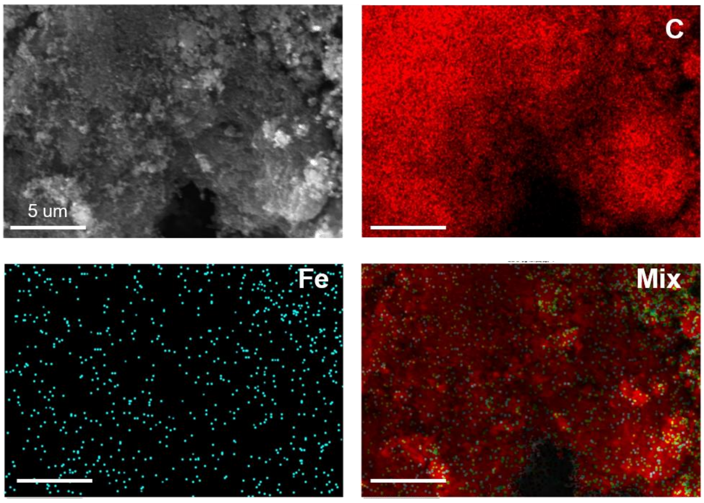

Supplementary Fig. 60. EDX elemental mapping of the obtained CNTs after 30 successive cycles. The produced CNTs were treated in an air atmosphere at 550 °C for 0.5 h and then acid treated (~2.5 mol/L) at 50 °C for 2 h.

CNTs production conditions: using FeNi/Ni/C as the catalyst. Feedstock: plastic mixtures of LDPE, HDPE, PP, PS, PVC and PTFE with mass ratios of 15:7:2:5:7:5 (cycle 1 - cycle 5), 40:30:40:20:5:1 (cycle 6 - cycle 10), 2:6:4:6:4:1 (cycle 11 - cycle 15), 10:10:3:10:5:5 (cycle 16 - cycle 20), 5:10:3:15:10:3 (cycle 21 - cycle 25) and 5:2:15:5:5:1 (cycle 26 - cycle 30). 3 g LDPE, HDPE, PP, PS, PVC and PTFE plastic mixtures and 3 g FeNi/Ni/C catalyst in the bottom layer; 3 g FeNi/Ni/C catalyst in the top layer.

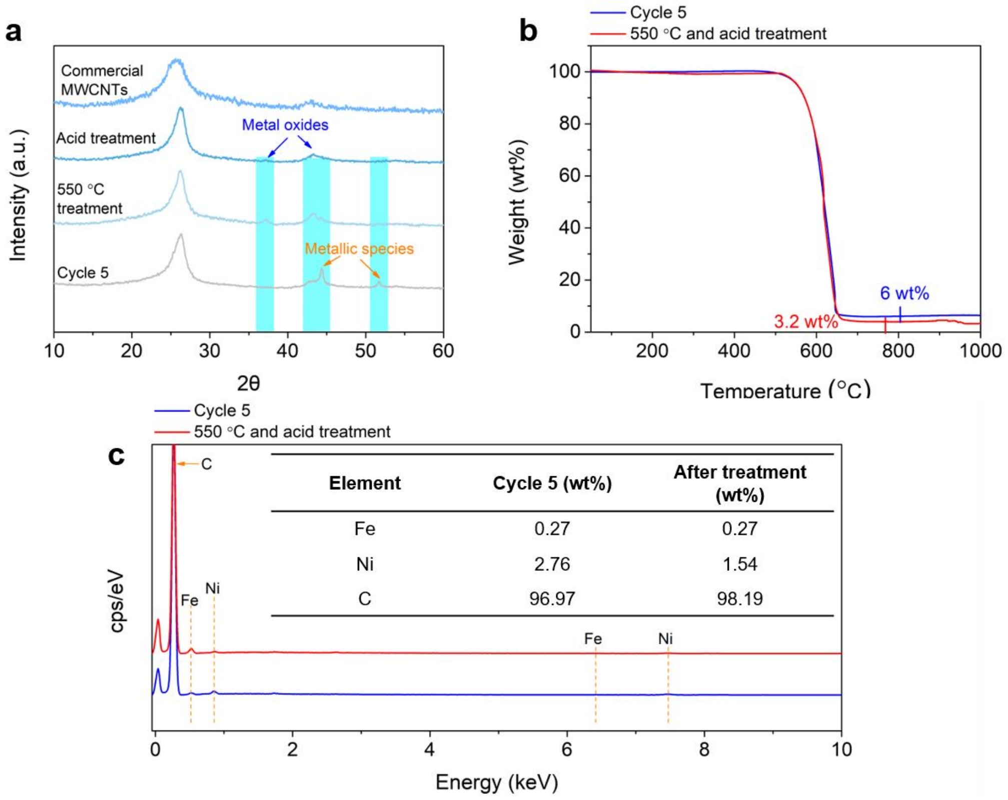

Supplementary Fig. 61. (**a**) XRD patterns of the prepared CNTs (Cycle 5) before and after treatment, with the XRD pattern of industrial-grade multi-walled carbon nanotubes as a reference. (**b**) The metal content of the CNTs before and after treatment is approximately 6 wt% and 3.2 wt%, respectively. (**c**) EDX spectra and elemental composition of the prepared CNTs before and after treatment. The produced CNTs were treated in an air atmosphere at 550 °C for 0.5 h and then acid treated (~2.5 mol/L) at 50 °C for 2 h.

CNTs production conditions: using FeNi/Ni/C as the catalyst. Feedstock: LDPE, HDPE, PP, PS, PVC and PTFE plastic mixtures (875:615:965:330:510:12). 30 g LDPE, HDPE, PP, PS, PVC and PTFE plastic mixtures and 1.5 g FeNi/Ni/C catalyst in the bottom layer; 1.5 g FeNi/Ni/C catalyst in the top layer.

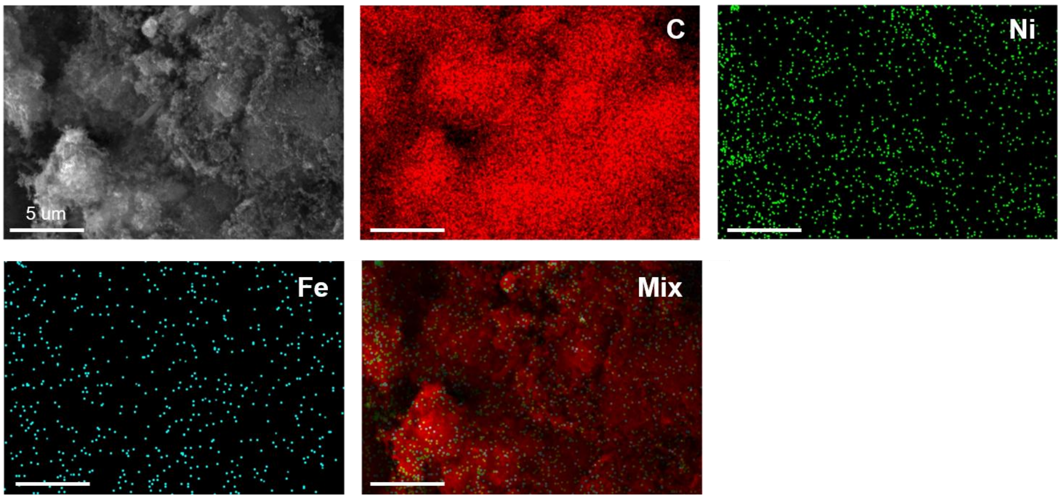

Supplementary Fig. 62. EDX elemental mapping of the obtained CNTs after 5 successive cycles.

CNTs production conditions: using FeNi/Ni/C as the catalyst. Feedstock: LDPE, HDPE, PP, PS, PVC and PTFE plastic mixtures (875:615:965:330:510:12). 30 g LDPE, HDPE, PP, PS, PVC and PTFE plastic mixtures and 1.5 g FeNi/Ni/C catalyst in the bottom layer; 1.5 g FeNi/Ni/C catalyst in the top layer.

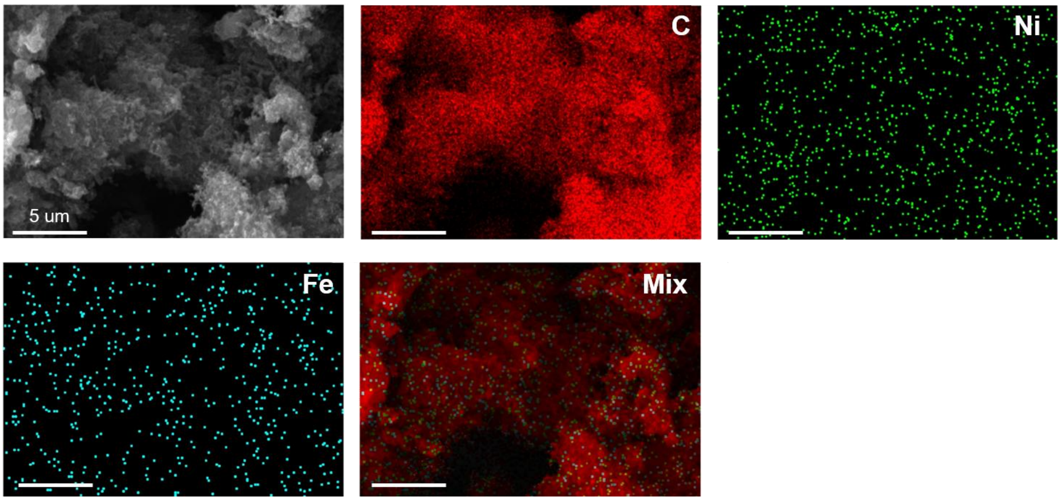

Supplementary Fig. 63. EDX element mapping of the CNTs obtained 5 successive cycles. The produced CNTs were treated in an air atmosphere at 550 °C for 0.5 h and then acid treated (~2.5 mol/L) at 50 °C for 2 h.

CNTs production conditions: using FeNi/Ni/C as the catalyst. Feedstock: LDPE, HDPE, PP, PS, PVC and PTFE plastic mixtures (875:615:965:330:510:12). 30 g LDPE, HDPE, PP, PS, PVC and PTFE plastic mixtures and 1.5 g FeNi/Ni/C catalyst in the bottom layer; 1.5 g FeNi/Ni/C catalyst in the top layer.

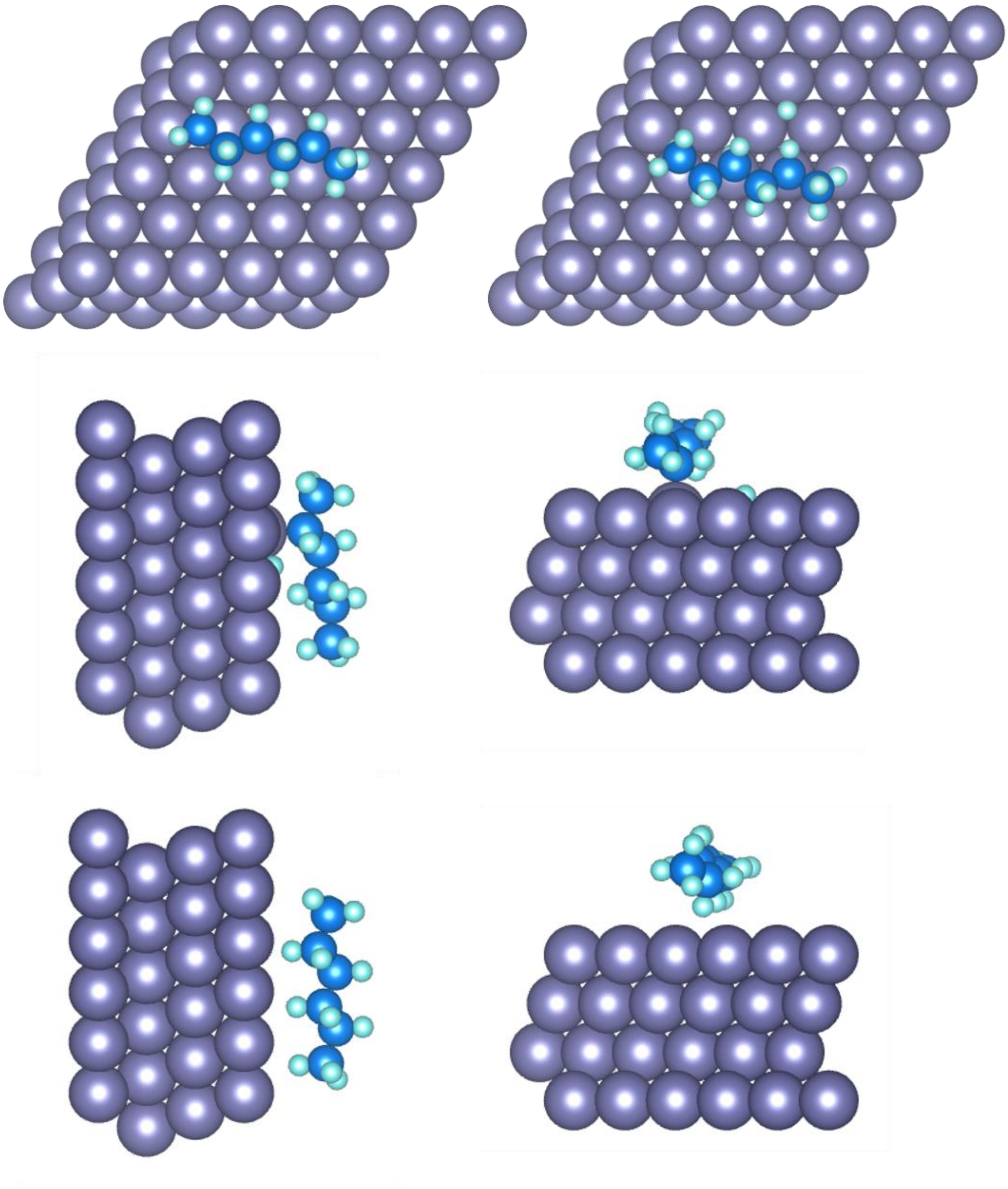

Supplementary Fig. 64. Models and different side-views of the catalytic decomposition of n-hexane by Ni/C.

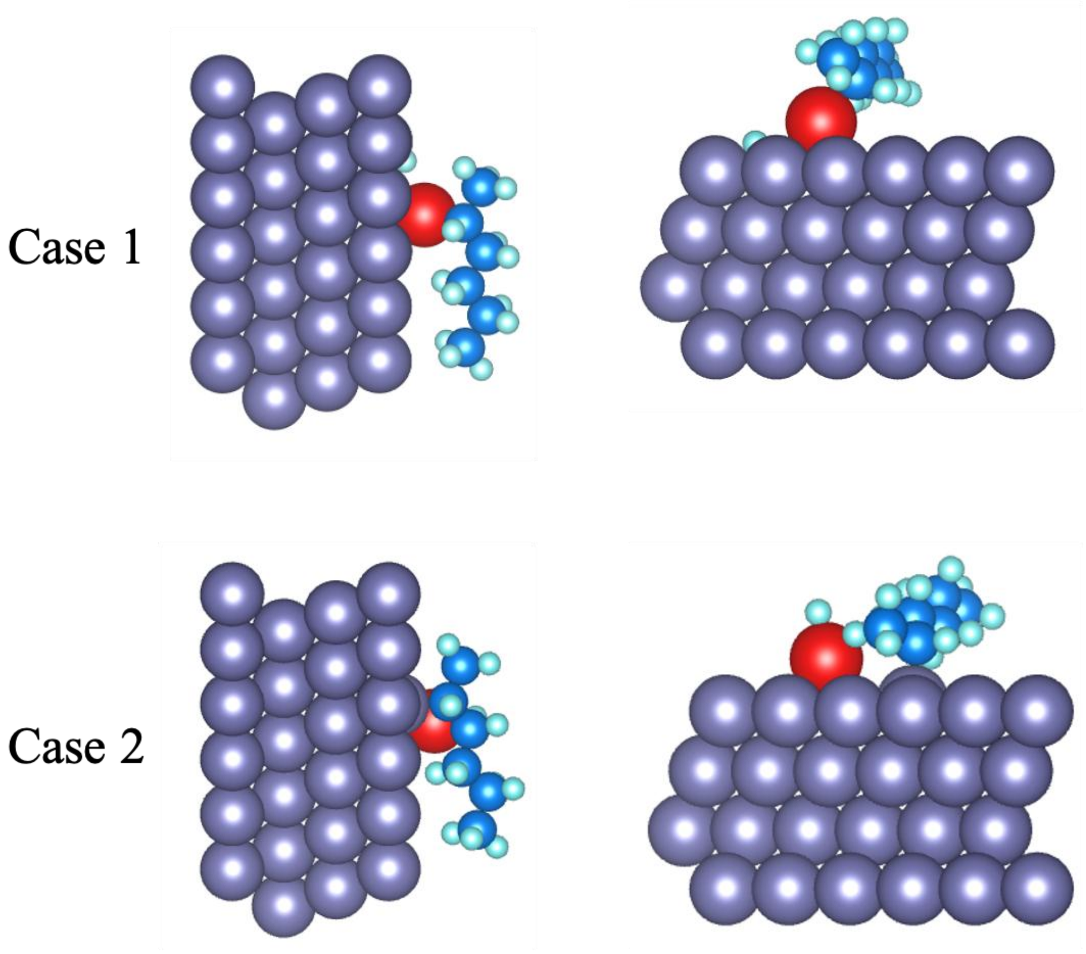

Supplementary Fig. 65. Models and different side-views of the catalytic decomposition of n-hexane by FeNi/Ni/C.

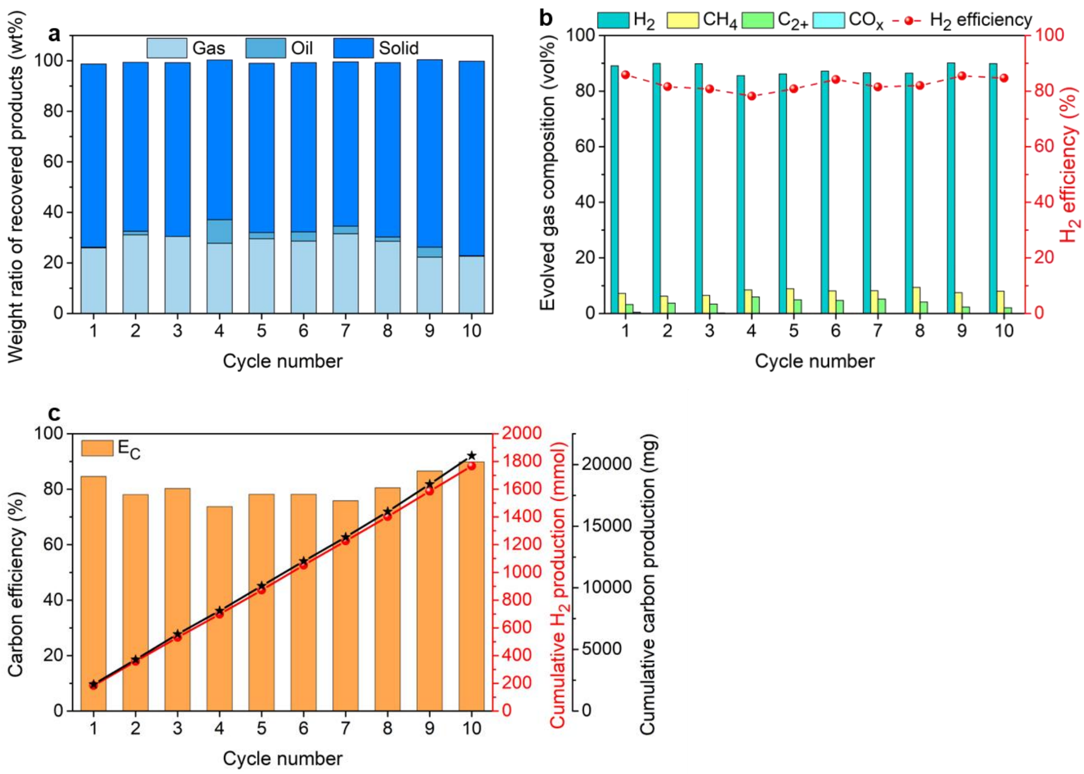

Supplementary Fig. 66. Successive cycles of microwave catalytic decomposition of LDPE over Ni/C catalyst. (a) The weight ratio of recovered gas, oil and solid. (b) Corresponding evolved gas composition (vol%) and H_2 efficiency (%). (c) Carbon efficiency (%), cumulative H_2 production (mmol) and cumulative carbon production (mg). Tandem catalysis method.

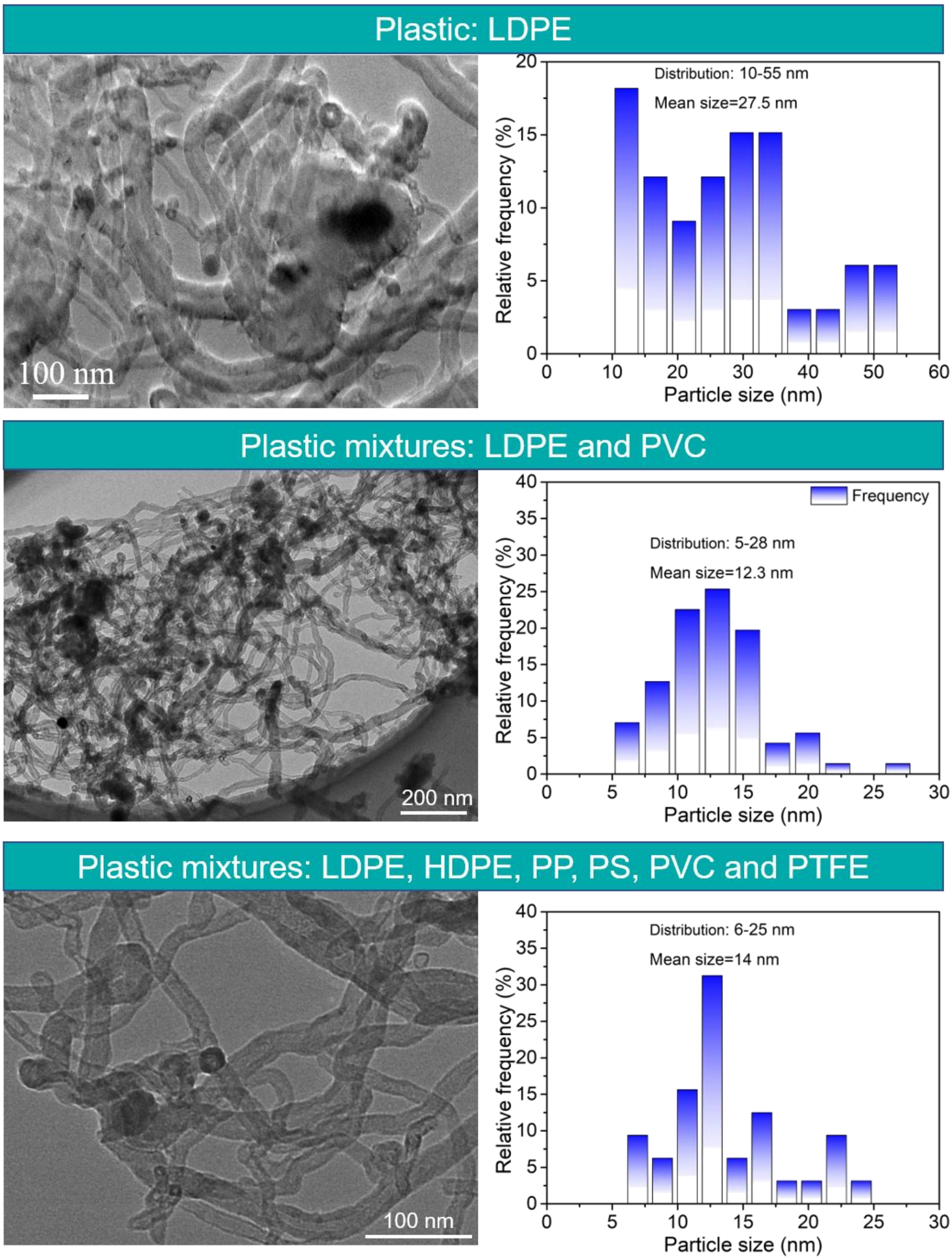

Supplementary Fig. 67. The outer diameter distribution of the resulting carbon nanotubes after microwave catalytic decomposition of different types of plastics over FeNi/Ni/C catalyst.

2. Supplementary tables

Supplementary Table 1. Experimental results for the microwave catalytic decomposition of low-density polyethylene (LDPE) using different catalysts.

	Evolved gas composition (vol%)				H_2 efficiency (%)	Gas weight ratio (wt%)	Oil weight ratio (wt%)	Solid weight ratio (wt%)	Mass balance (%)
	CH_4	C_{2+}	H_2	CO_x					
Ni/C	7.2	3.3	89.1	0.4	85.8	26	0.2	72.5	98.7
$Ni_{19}Fe/C$	6.1	3.1	90.1	0.7	88	18.7	1.7	77.7	98.1
$Ni_{9.5}Fe/C$	5	0.5	92	2.5	90	17.1	3.6	78.2	98.9
$Ni_{6.3}Fe/C$	6.3	1.3	91.3	1.1	87	24.4	5.9	68.3	98.6
Fe_3C	4	3.9	77.1	15	76	49.1	14.3	35.8	99.2

Supplementary Table 2. Experimental results for the microwave catalytic decomposition of low-density polyethylene (LDPE) using different microwave irradiation power.

	Evolved gas composition (vol%)				H_2 efficiency (%)	Gas weight ratio (wt%)	Oil weight ratio (wt%)	Solid weight ratio (wt%)	Mass balance (%)
	CH_4	C_{2+}	H_2	CO_x					
500 W	5.8	0.9	87.5	5.8	85.7	24	8.2	67.1	99.3
600 W	5	0.5	92	2.5	90	17.1	3.6	78.2	98.9
700 W	3.5	12.1	79	5.4	84	25.6	6.9	66.1	98.6

Supplementary Table 3. Experimental results for the microwave catalytic decomposition of low-density polyethylene (LDPE) during the optimization of the weight ratio of the mixture (plastic: catalyst) in the bottom layer.

Plastic: Catalyst	Evolved gas composition (vol%)				H_2 efficiency (%)	Gas weight ratio (wt%)	Oil weight ratio (wt%)	Solid weight ratio (wt%)	Mass balance (%)
	CH_4	C_{2+}	H_2	CO_x					
1:1	5	0.5	92	2.5	90	17.1	3.6	78.2	98.9
2:1	6.8	6	86.8	0.4	84.9	26.1	6.9	65	98
3:1	7.8	5.2	86.8	0.2	83.8	29	1.2	68	98.2

Supplementary Table 4. Experimental results for 35 successive cycles of the microwave catalytic decomposition of low-density polyethylene (LDPE) or polyvinyl chloride (PVC) using FeNi/Ni/C as the catalyst. Feedstock: LDPE (Cycle 1~Cycle 25, Cycle 31~Cycle 35), PVC (Cycle 26~Cycle 30). Tandem catalysis method.

	Evolved gas composition (vol%)				H_2 efficiency (%)	Gas weight ratio (wt%)	Oil weight ratio (wt%)	Solid weight ratio (wt%)	Mass balance (%)
	CH_4	C_{2+}	H_2	CO_x					
Cycle 1	5	0.5	92	2.5	90	17.1	3.6	78.2	98.9
Cycle 2	8.3	5.	85.6	1.1	83.5	10.4	6.7	80.2	ca.100
Cycle 3	7.4	8.3	83.7	0.6	83.7	22	6	72.2	99
Cycle 4	9.4	4.2	85.8	0.6	82	20.4	6.1	72.5	98
Cycle 5	6.9	1.7	89.7	1.7	86.2	22.4	5	70.6	99
Cycle 6	9	1.8	88.3	0.9	82	22.5	7.8	68.7	ca.100
Cycle 7	0.9	0.2	98.4	0.5	98.2	19	0	81.7	98.4
Cycle 8	8.4	0.9	90.1	0.6	84	19.7	0	78.7	99
Cycle 9	5.8	0.2	92.6	1.4	88.6	17.3	0	81.7	99
Cycle 10	5.8	0.2	92.7	1.3	88.7	18.2	0	80.8	99.4
Cycle 11	5.5	0.4	93	1.1	89.2	16	0	83.4	ca.100
Cycle 12	3.5	0.2	94.8	1.5	93	15.5	0	84.6	99.8
Cycle 13	6.5	0.8	91.1	1.6	87	18.3	0	81.5	99.3
Cycle 14	13	2.1	83.9	1	76.2	28.8	1	69.5	99.9
Cycle 15	11.9	3	84.3	0.8	77.8	24.1	1.8	74	98.8
Cycle 16	2.4	0.4	94.4	2.8	78.3	14.5	0.1	84.2	98.9
Cycle 17	4.1	0.3	94	1.6	91.8	21.6	0.3	77	99.1

Cycle 18	9.1	1.5	88.3	1.1	76.4	26.5	3.3	69.3	99.5
Cycle 19	30	15	54.9	0.1	46	23.5	2.2	73.8	ca.100
Cycle 20	23.7	3.4	72.3	0.6	60	40.8	5.6	53.9	95.7
Cycle 21	27.6	9.3	62.8	0.3	53	49.9	5.3	40.5	99
Cycle 22	19.8	6.1	73.5	0.6	57.9	57.1	0.7	41.2	98.5
Cycle 23	25.9	22	52.1	-	46.3	50.1	2	45.5	97.6
Cycle 24	27.2	14.8	57.5	0.5	39.2	42.6	0.7	54.3	99.2
Cycle 25	29.1	13.5	57.1	0.3	48.3	55.7	3.3	40.2	93.7
Cycle 26	2.9	2.9	89.3	4.9	70.4	55.6	4.8	33.3	92.8
Cycle 27	2.5	1.8	89.7	6	89.7	66.7	1.3	24.8	90.8
Cycle 28	2.1	1.4	94.4	2.1	95.3	63.4	1.6	25.8	90.5
Cycle 29	2.4	2.1	93.4	2.1	92.8	60.5	0.7	29.3	93
Cycle 30	2.4	1.7	92.5	3.4	83.5	57.6	1.2	34.2	96.6
Cycle 31	5.4	1.5	89	4.1	85.4	21.6	5	70	98.3
Cycle 32	13.8	2.2	83.3	0.7	74	31.4	-	66.9	97.3
Cycle 33	14.6	2.5	82.3	0.6	73	30.9	-	66.4	98
Cycle 34	21.2	3.8	74.3	0.7	63	28	4.1	65.9	97
Cycle 35	26.3	5.5	67.8	0.4	54.9	35	1.3	60.7	ca.100

Supplementary Table 5. The product distribution of PVC following pyrolysis under catalyst-free conditions.

	Time (min)	Product	Percentage (%)
1	1.89	HCl	17.86
2	2.16	C_5H_6	1.07
3	2.65	C_6H_6	17.48
4	3.54	C_7H_8	5.18
5	5.11	C_8H_8	2.69
6	7.46	C_9H_8	2.13
7	9.32	$C_{10}H_{10}$	2.06
8	9.65	$C_{10}H_8$	4.78
9	11.22	$C_{11}H_{10}$	1.9
10	12.52	$C_{13}H_{12}$	1.25
11	17.19	$C_{14}H_{10}$	1.67

Supplementary Table 6. Experimental results for 30 successive cycles of the microwave catalytic decomposition of low-density polyethylene (LDPE) and polyvinyl chloride (PVC) mixture (LDPE: PVC=9:1) using FeNi/Ni/C as the catalyst.

	Evolved gas composition (vol%)				H_2 efficiency (%)	Gas weight ratio (wt%)	Oil weight ratio (wt%)	Solid weight ratio (wt%)	Mass balance (%)
	CH_4	C_{2+}	H_2	CO_x					
Cycle 1	6.2	1.3	89.4	3.1	87.3	29.1	3.4	67.5	ca.100
Cycle 2	3.4	0.9	93.1	2.6	89.8	29	1.6	69.5	ca.100
Cycle 3	4.3	0.8	93.4	1.5	91	29.4		70	99.4
Cycle 4	4.4	0.9	93.4	1.3	91.2	26.06	1.3	73.3	ca.100
Cycle 5	5.1	0.9	92.2	1.8	90	30	3	67.1	ca.100
Cycle 6	4.7	1	93.3	1	90.4	30.4	2.6	66.5	99.5
Cycle 7	1.8	0.5	94.5	3.2	89.6	35	2.3	62	99.3
Cycle 8	1.6	0.5	95	2.9	96	22		75.6	97.6
Cycle 9	1.1	0.2	96.3	2.4	97	20.6		77	97.6
Cycle 10	2.7	3.3	90.5	3.5	94.1	23		77	ca.100
Cycle 11	4.7	0.4	91.9	3	90.5	35.3		65.2	ca.100
Cycle 12	1.0	0.1	96.4	2.5	97.6	18	1.6	77.1	96.7
Cycle 13	4.2	0.8	92.4	2.6	91.3	38.7		61.8	ca.100
Cycle 14	1.2	0.1	95.3	3.4	97.1	23.6		75.5	99.1
Cycle 15	0.7		95.9	3.4	98.2	22.6		77	99.6
Cycle 16	1.5		95.7	2.8	96.3	27.4		72.9	ca.100
Cycle 17	5.2		92.4	2.4	89.6	35.3	1	64.5	ca.100
Cycle 18	4.4		92.6	3	91.1	32.3		67.4	99.7

Cycle 19	2.5		95	2.5	94.8	25		75.1	ca.100
Cycle 20	2.8		94.3	2.9	94	25.6		74.6	ca.100
Cycle 21	6.5	0.2	91	2.3	87.2	34.3		65.9	ca.100
Cycle 22	2.3		94.3	3.4	95.1	21.5		77	98.5
Cycle 23	7.3		89.9	2.8	85.7	35.7		64.3	ca.100
Cycle 24	6.8		90.3	2.9	86.5	32		67.4	99.4
Cycle 25	2.6		93.9	3.5	94.2	24.3		75.3	99.6
Cycle 26	1.9		93.7	4.4	94	26		74.7	ca.100
Cycle 27	2.1		95.3	2.6	95.6	24.5		75.2	99.7
Cycle 28	5.1		91.9	3	89.8	31	1.6	67.8	ca.100
Cycle 29	3.6		92.7	3.7	90.6	31.1		68	99.1
Cycle 30	5.5		91.8	2.7	89	29.1	1	69.3	99.4

Supplementary Table 7. Comparison of catalytic performance of hydrogen production from plastic waste with other works.

Type of reactor	Catalyst	Plastic	Steam (Y/N)	Gas weight ratio (wt%)	Oil weight ratio (wt%)	Solid weight ratio (wt%)	Evolved gas composition (Vol%)				H_2 yield (mmol·g$^{-1}_{plastic}$)	Hydrogen efficiency (%)	Ref.
							H_2	CH_4	C_{2+}	CO_x			
Microwave reactor	FeNi/Ni/C	Mixed plastics	N	29.1	3.4	67.5	89.4	6.2	1.3	3.1	58.2 (Cycle 1)	87.3	This work
				30.0	3.0	67.1	92.2	5.1	0.9	1.8	60 (Cycle 5)	90	
				23.0		77.0	90.5	2.7	3.3	3.5	62.7 (Cycle 10)	94.1	
				22.6		77.0	95.9	0.7		3.4	65.5 (Cycle 15)	98.2	
				25.6		74.6	94.3	2.8		2.9	62.7 (Cycle 20)	94	
				24.3		75.3	93.9	2.6		3.5	62.8 (Cycle 25)	94.2	
				29.1	1.0	69.3	91.8	5.5		2.7	59.3 (Cycle 30)	89	
	$FeAlO_x$	HDPE	N	62.7	2.2	35.1	74.3	5.8	2.8	17	55.5 (Cycle 1)	77.7	3
				27.3	73.1		88.0	5.0	0.9	6.1	50.5 (Cycle 2)	70.7	
				28.4	72.0		83.1	7.8	3.4	5.7	40.5 (Cycle 3)	56.7	
				28.0	68.1	3.9	82.6	8.1	3.5	5.8	39.0 (Cycle 4)	54.6	
				34.2	63.1	2.6	78.4	5.9	10.4	5.3	35.0 (Cycle 5)	49.0	
	Fe-Co-Al	LDPE	N	36.1	1.5	62.4	82.4	5.1	2.6	9.9	61.39 (Cycle 1)	85.9	5
	Ti_3AlC_2	LDPE	N	47.2	2.1	50.7	74.9	3.8	6.7	14.6	51.3 (Cycle 1)	71.8	6
				58.0	2.0	40.0	80.0	3.6	12.9	3.5	55.0 (Cycle 2)	77.0	
				59.0	5.0	36.0	68.0	12.0	17.0	3.0	43.0 (Cycle 3)	60.2	
				53.0	1.0	46.0	73.0	1.0	10.0	16.0	44.0 (Cycle 4)	61.6	
				64.0	3.5	32.5	70.0	1.0	7.0	22.0	45.0 (Cycle 5)	63.0	
	Fe/Ni-CeO_2 @CNTs	LDPE	N	28	4.1	67.9	91.5	5.0	0.5	3.0	50.2 (Cycle 1)	70.3	7
							87.0	8.5	0.5	4.0	48.0 (Cycle 2)	67.2	
							90.0	7.0	1.0	2.0	46.0 (Cycle 3)	64.4	
							83.0	11.0	2.0	4.0	41.5 (Cycle 4)	58.1	
							83.0	13.0	0.5	3.5	37.5 (Cycle 5)	52.5	
	Fe/AC	PE	N				>70				27.6	38.6	8
	Fe/$FeAl_2O_4$	HDPE	N	32.05			84.96	2.42	5.57	7.05	47.0	65.8	9
	$Al_iFe_jO_K$	LDPE	N				87.5				60.2	84.3	10
Two-stage fixed bed reactor	$La_{0.6}Ca_{0.4}Co_{0.2}Fe_{0.8}O_{3-\delta}$	PP	N	45.1	24.5	29.0	55.1	32.5	12.4		34.3	48.0	11
	Ni-Fe/MCM-41	Waste plastic	N	30.8	16.3	55.6	77.46	14.41	1.62	6.51	38.1	53.4	12

Fe/γ-Al_2O_3	PP	N	40.2	15.9	39.5	82.0				62.0	86.8	13
NiMnAl444	PP	Y	34.6	6.3	57.7	75.6	12.7	10.7	1.0	37.2		14
Ni–Mn–Al			131.1		23.0	62.7	9.2	22.7	5.4	71.5		
Ni–Ca–Al			148.2		9.5	58.3	9.4	27.9	4.4	68.5		
Ni–Mg–Al	PP	Y	168.4		3.5	56.9	9.8	29.8	3.5	75.5		15
Ni–Ce–Al			148.6		8.0	55.6	10.6	29.9	3.9	63.0		
Ni–Zn–Al			120.9		10.0	52.7	13.4	28.9	5.0	46.0		

Supplementary Table 8. Fitting results of the precursor and regenerated catalyst.

Names	Shell	CN	R (Å)	$\sigma^{\wedge 2}$ (10^{-2} Å^2)	ΔE_0 (eV)	r-factor
Precursor (Fe)	Fe-C	3.1(±0)	2.0(±0)	0.006(set)	-3(±0)	0.015
	Fe-Ni	6.7(±0.01)	2.48(±0)			
Precursor (Ni)	Ni-Fe	6.5(±0.4)	2.49(±0.004)	0.39(±0.057)	7.3(±0.7)	0.006
	Ni-Ni	8.7(±0.3)	2.5(±0.002)	0.6(±0.03)		
Cycle 5 (Fe)	Fe-Ni	8.5(±0.3)	2.49(±0.002)	0.6(±0.037)	7.8(±0.44)	0.002
Cycle 5 (Ni)	Ni-Fe	6.8(±0.4)	2.5(±0.004)	0.4(±0.05)	6.1(±0.4)	0.0018
	Ni-Ni	9.1(±0.3)	2.49(±0.002)	0.63(±0.03)		
Cycle 15 (Fe)	Fe-Ni	8.2(±0.4)	2.49(±0.003)	0.6(±0.04)	6.98(±0.57)	0.003
Cycle 15 (Ni)	Ni-Fe	6.6(±0.4)	2.5(±0.004)	0.4(±0.056)	6.8(±0.7)	0.006
	Ni-Ni	8.9(±0.3)	2.51(±0.003)	0.6(±0.03)		
Cycle 30 (Fe)	Fe-Ni	8.7(±0.4)	2.49(±0.003)	0.65(±0.045)	8.1(±0.54)	0.003
Cycle 30 (Ni)	Ni-Fe	6.8±0.4	2.5(±0.004)	0.4(±0.055)	6.8(±0.7)	0.006
	Ni-Ni	9.0±0.3	2.51(±0.002)	0.63(±0.03)		

CN: coordination number; R: distance between absorber and backscatter atoms; σ^2: Debye-Waller factor to account for both thermal and structural disorders; ΔE_0: Inner potential correction; r factor (%) indicates the goodness of the fit. Fitting conditions: k around 3.0-12.0. R around 1.0-3.0. Rbkg=1. kw=1/2/3

Supplementary Table 9. The textural properties of precursor and long-term used (cycle 30) catalysts.

	BET surface area (m^2/g)	BJH adsorption cumulative volume of pores (cm^3/g)	BJH adsorption average pore diameter (nm)
Precursor	25.36	0.102	3.82
Cycle 30	60.48	0.158	3.06

Supplementary Table 10. Experimental results for 30 successive cycles of the microwave catalytic decomposition of LDPE, HDPE, PP, PS, PVC and PTFE plastic mixtures using FeNi/Ni/C as the catalyst. Feedstock: plastic mixtures of LDPE, HDPE, PP, PS, PVC and PTFE with mass ratios of 15:7:2:5:7:5 (cycle 1 - cycle 5), 40:30:40:20:5:1 (cycle 6 - cycle 10), 2:6:4:6:4:1 (cycle 11 - cycle 15), 10:10:3:10:5:5 (cycle 16 - cycle 20), 5:10:3:15:10:3 (cycle 21 - cycle 25) and 5:2:15:5:5:1 (cycle 26 - cycle 30).

	Evolved gas composition (vol%)				H_2 efficiency (%)	Gas weight ratio (wt%)	Oil weight ratio (wt%)	Solid weight ratio (wt%)	Mass balance (%)
	CH_4	C_{2+}	H_2	CO_x					
Cycle 1	3.6	1.5	88.8	6.1	87	37.1	6.6	56.1	99.8
Cycle 2	1.4	0.4	93	5.2	92.1	36	4	59.8	99.8
Cycle 3			96	4	96.6	40	1	58.5	99.5
Cycle 4			98	2	99.8	39.6	0	60	99.6
Cycle 5			95.8	4.2	99.9	39.5	0	60.2	99.7
Cycle 6	3.9	0.5	93.3	2.3	92.3	29.3	0	70.5	99.8
Cycle 7			96.9	3.1	98.7	25.9	0	73.8	99.7
Cycle 8			96.9	3.1	99.9	20.8	0	79	99.8
Cycle 9			97.2	2.8	99.7	29.1	0	70.5	99.6
Cycle 10	3	0.3	94.2	2.5	94	22.6	0	77	99.6
Cycle 11	2.3	0.1	93.2	4.4	95	35.2	0	64.3	99.5
Cycle 12	2.2	0.2	92.8	4.8	95.3	32.8	0	67	99.8
Cycle 13	2.5	0.3	93.2	4	94.5	37.2	0	62.1	99.3
Cycle 14	2	0.3	93.3	4.4	95.6	35.9	0	64	99.9
Cycle 15	2	0.1	93.1	4.8	95.7	32.6	0	67	99.6
Cycle 16	1		91	8	96.7	38	0	61.3	99.3

Cycle 17	0.9		90.2	8.9	93.3	36.7	0	63	99.7
Cycle 18	0.6		86.9	12.5	91.4	37	0	62.8	99.8
Cycle 19	0.7	0.6	90.5	8.2	86.9	35.7	0	64	99.7
Cycle 20	0.8	0	91.3	7.9	90.3	35.8	0	63.5	99.3
Cycle 21	0.5		89.2	10.3	93.3	39.6	0	60	99.6
Cycle 22	0.5	0	92.5	7	89.5	36.6	0	63.1	99.7
Cycle 23	0.5	0	98.6	0.9	98.6	34.6	0	65	99.6
Cycle 24	0.5		92.4	7.1	92.7	33	0	66.3	99.3
Cycle 25	3	0.1	91.9	5	92.5	37.1	0	62.5	99.6
Cycle 26	6.8	0.9	89.4	2.9	86.5	36	0	63.9	99.9
Cycle 27	7.1	1.4	87.8	3.7	86	37.2	0	62.6	99.8
Cycle 28	7	0.9	89.8	2.3	86	40.8	0	59	99.8
Cycle 29	5.4	0.6	89.6	4.4	89	36.3	0	63.5	99.8
Cycle 30	7.7	1.2	86.9	4.2	82.2	38.5	1.7	59.2	99.4

Supplementary Table 11. Experimental results for 35 successive cycles of the microwave catalytic decomposition of landfilled plastic waste mixtures using FeNi/Ni/C as the catalyst. Feedstock: PVC and landfilled plastic waste mixture with a mass ratio of 1:9 (cycle 1 - cycle 30), PTFE and landfilled plastic waste mixture with a mass ratio of 1:9 (cycle 31 - cycle 35).

	Evolved gas composition (vol%)				H_2 efficiency (%)	Gas weight ratio (wt%)	Oil weight ratio (wt%)	Solid weight ratio (wt%)	Mass balance (%)
	CH_4	C_{2+}	H_2	CO_x					
Cycle 1	5.2	1.9	90.1	2.8	85.2	22	5	66	93
Cycle 2	6.8	1.9	89.5	1.8	81.7	24.7	3.3	63.7	91.7
Cycle 3	5.2	4.1	89.3	1.4	84.4	34.8	2	57.2	94
Cycle 4	5.1	5	88	1.9	81.5	33.6	6	55.6	95.2
Cycle 5	5.7	5.3	87.3	1.7	78.7	37.5	2.3	57	96.8
Cycle 6	7.1	6.4	84.7	1.8	78.1	36.2	5.3	55.1	96.6
Cycle 7	5.6	2.4	90	2	88	27.2	0	69.7	96.9
Cycle 8	4.6	1.8	91.3	2.3	90	23.8	1.5	70.8	96.1
Cycle 9	3.	0.3	93.7	3	93	21.3	0	76.3	97.6
Cycle 10	3	1.1	93.4	2.5	93.9	23.9	0	70.2	94.1
Cycle 11	3.1	0.3	94.6	2	93.8	17.7	0	78.6	96.3
Cycle 12	7.9	1.6	88	2.5	84.3	29.8	-	65.6	95.4
Cycle 13	5.8	0.3	91.3	2.6	88.5	25.8	0	70.2	96
Cycle 14	8.4	0.8	88.2	2.6	83.6	34	0	61.2	95.2
Cycle 15	8.3	0.3	89.3	2.1	84	32	0	62.3	94.3
Cycle 16	11.8	3.1	83.4	1.7	77.7	36.1	0	64.2	ca.100
Cycle 17	12.5	4.4	81.4	1.7	70.4	29.4	2.3	66.8	98.5

Cycle 18	7.8	1.4	89	1.8	84.6	15.7	0	77.2	92.9
Cycle 19	13.2	3.3	81.9	1.6	75.5	32.7	0	65	97.7
Cycle 20	14.3	3.8	79.7	2.2	73.2	37.6	5	56.5	99.1
Cycle 21	16.9	8.4	72.2	2.5	66.9	42.6	-	55.2	97.8
Cycle 22	9.7	2	86.2	2.1	78.4	25.6	-	67.4	93
Cycle 23	19	5.8	73	2.2	65	37.9	3	51.6	92.5
Cycle 24	20.7	7.6	70	1.7	62.6	34.3	0	60.4	94.7
Cycle 25	17.6	5	75.6	1.8	67.5	33.2	3	59.8	96
Cycle 26	22.6	8.3	67.7	1.4	54.4	33.7	5	56.3	95
Cycle 27	20.8	6.3	71.8	1.1	61.6	48.3	2.3	47.3	97.9
Cycle 28	23.7	8.1	66.9	1.3	49.2	41.9	4.3	49.3	95.5
Cycle 29	21.2	8.5	68.8	1.5	54.2	45.4	0	49.3	94.7
Cycle 30	22.3	6.1	69.9	1.7	53.1	44.6	3	47.4	95
Cycle 31	10.8	3.7	76.1	9.4	77	52.3	0	47.1	99.4
Cycle 32	21.1	6.6	69.4	2.9	62	48	0.3	50.3	98.6
Cycle 33	11.2	4	84	0.8	78	39	0.2	60.1	99.3
Cycle 34	11.1	4.9	82.9	1.1	78.1	45.1	0.2	54.2	99.5
Cycle 35	9.1	3.9	85.9	1.1	77.1	42.3	0.2	56.3	98.8

Supplementary Table 12. The product distribution of PTFE following pyrolysis under catalyst-free conditions.

	Time (min)	Product	Percentage (%)
1	2.07	C_2F_4	98.97
2	2.53	$C_4H_3F_7O$	0.14
3	2.6	$C_4H_3F_7O$	0.05
4	2.75	C_8F_{18}	0.23
5	3.41	$C_{12}HF_{23}O_2$	0.02
6	3.44	$C_8HF_{17}O_3$	0.02
9	4.61	$C_4H_2O_3$	0.34
10	4.97	C_6F_{14}	0.03
11	5.21	$C_{12}HF_{23}O_2$	0.2

Supplementary Table 13. Experimental results for 10 successive cycles of the microwave catalytic decomposition of low-density polyethylene (LDPE) using Ni/C as the catalyst.

	Evolved gas composition (vol%)				H_2 efficiency (%)	Gas weight ratio (wt%)	Oil weight ratio (wt%)	Solid weight ratio (wt%)	Mass balance (%)
	CH_4	C_{2+}	H_2	CO_x					
Cycle 1	7.2	3.3	89.1	0.4	85.8	26	0.23	72.5	98.73
Cycle 2	6.2	3.8	90	-	81.6	31.1	1.4	66.9	99.4
Cycle 3	6.5	3.4	89.8	0.3	80.6	30.5	-	68.8	99.3
Cycle 4	8.4	5.9	85.6	0.1	78.1	27.7	9.4	63.2	ca.100
Cycle 5	8.9	4.9	86.2	-	80.8	29.6	2.5	67	99.1
Cycle 6	8.1	4.7	87.2	-	84.2	28.6	3.7	67	99.3
Cycle 7	8.2	5.2	86.6	-	81.5	31.6	3	65	99.6
Cycle 8	9.3	4.1	86.5	0.1	82	28.6	1.7	69	99.3
Cycle 9	7.5	2.3	90.1	0.1	85.5	22.3	4	74.2	ca.100
Cycle 10	8	2.1	89.9	-	84.7	22.7	0.1	77	99.8

Supplementary Table 14. Equipment parameters.

Equipment	Microwave reactor	Filter[16]	Compressor[17]	Condenser[17]
Ref cost ($)	3000000	96223	3197	5526
Ref scale		2 t_{solid}/h	7.2 m^3/h	11.88 m^3/h
Scale		0.63 t/h	1153.6 m^3/h[a]	1153.6 m^3/h[b]
Scaling factor		0.5	0.77	0.6
Equipment	Desiccator[18]	Acid treatment equipment[18]		
a	591236	473892		
b	0.6	0.4481		
Scale	0.63	0.26[c]		

a: 0.103(t_{H2}/h)/2(g/mol)*1000000(g/t)*22.4(l/mol)/1000(l/m^3)

b: 0.2 t_{water}/h*1.3 (30 wt% Cl and F/t_{water})

Equipment prices for microwave reactor, filter, compressor, and condenser are calculated according to the following formula[19]:

$$\text{Equipment cost} = \text{ref cost}*\left(\frac{\text{scale}}{\text{ref capacity}}\right)^{\text{scaling factor}}$$

Equipment prices for desiccator and acid treatment equipment are calculated according to the following formula[18]:

$$\text{Equipment cost} = \text{a}*\text{scale}^{\text{b}}$$

Supplementary Table 15. Techno-economic analysis parameters.

**Basic data**						
Capacity	1.00	$t_{plastic}$/h	Shift position		3	
Plant life	20	y	Number of operators per shift		3	
Annual operating days	333.33	d/y	Operating labor cost		30000	$/y
Annual operating hours	8000	h/y	Nominal Interest Rate		0.1	
YIELD ESTIMATE			CAPITAL COSTS			
Scale of production set to $t_{plastic}$/h<br>product unit: t						$
			ISBL			18550754
			OSBL			7420301
			Engineering and construction costs			2597106
			Contingency charges			2597106
			**Fixed Capital Investment (FCI)**			31165266
ANNUALIZED CAPITAL CHARGES						
	$	Nominal Interest Rate	Plant life	Annual capital charge ratio (ACCR)	$/y	$/unit product
**Fixed capital investment (FCI)**	31165266	0.1	20	0.117	3660660	458
Total Annual Capital Charge (ACC)					3660660	458
FIXED OPERATING COSTS						
					$/y	$/unit product
Operating labor	9	Operators	30000	$/y each	270000	34
Supervision	25%	of Operating Labor			67500	8
Direct salary overhead	40%	of Operating Labor & Supervision			135000	17
Maintenance	3%	of ISBL			556523	70
Property taxes and insurance	1%	of ISBL			185508	23
General plant overhead	65%	of Labor + Superv+Direct salary overhead +Maint.			668865	84
Fixed costs of production (FCOP)					1883395	235
UTILITIES						

	Units	Units/Unit product	Units/y	Price $/units	$/y	$/unit product
Microwave reactor	kWh	1600	12800000	0.1	1280000	160
Condenser	kWh	51	404800	0.1	40480	5
Desiccator	kWh	459	3674160	0.1	367416	46
Compressor	kWh	89	708640	0.1	70864	9
Total Utilities (UTS)					1758760	220
CONSUMABLES						
	Units	Units/Unit product	Units/y	Price $/units	$/y	$/unit product
Catalyst	t	0.0570	456	55500	25308000	3164
Total Consumables(CONS)					25308000	3164
RAW MATERIAL COSTS						
	Units	Units/Unit product	Units/y	Price $/units	$/y	$/unit product
Plastic mixtures	t	1.0000	8000	300	2400000	300
Water	t	0.2000	1600	1.18	1888	0.24
Total Raw Materials (RM)					2401888	300
REVENUES						
Products	Units	Units/Unit product	Units/y	Price $/units	$/y	$/unit product
CNTs	t	0.6300	5040	14876	74975040	9372
H_2	t	0.103	824	1900	1565600	196
Total Revenues					76540640	9568
SUMMARY						
					$/y	$/unit product
Variable Cost of Production (VCOP = RM + CONS + UTS)					29468648	3684
Fixed Cost of Production(FCOP)					1883395	235
Cash Cost of Production(CCOP=VCOP+FCOP)					31352043	3919

Total Annual Capital Charge(ACC)	36606<br>60	458
Gross Profit(GP=REV-CCOP)	43622<br>997	5453
Total Cost of Production	35012<br>703	4377

Supplementary Table 16. Compared with other chlorinated/fluorinated plastic mixtures upcycling strategies.

Catalyst	Type of plastic waste	Temp. (°C)	Products	Achieved product value ($\$/ton_{plastic}$)	Pressure	Time (h)	Ref.
FeNi/Ni/C	LDPE, HDPE, PP, PS, PVC, PTFE mixtures	450	H_2 and CNTs	9568	1 bar	0.16	This work
Ni/SiO_2	PP, PET, PE, PVC, PS	430	CH_4	1016	1 bar (12 ml/min H_2)	4	20
$Mg_3AlO_{4.5}$ Pt/WO_3/ZrO_2	PP, PVC	250	Lubricants	5193	30 bar H_2	22	21
[C_4Py]Cl-$AlCl_3$	PP, PVC	25	C_4-C_{12} alkanes and alkenes	892	1 bar	3-17	22
Bu_4PCl $ZnCl_2$	PET, PVC	230	TPA and DHPVC	350	1 bar	10	23

3. Supplementary References

1 Cohen, N. Revised Group Additivity Values for Enthalpies of Formation (at 298 K) of Carbon–Hydrogen and Carbon–Hydrogen–Oxygen Compounds. *J. Phys. Chem. Ref. Data.* **25**, 1411-1481 (1996).

2 Bi, T. *et al.* Closed-loop recycling of polyethylene to ethylene and propylene via a kinetic decoupling–recoupling strategy. *Nat. Chem. Eng.* **2**, 650-661 (2025).

3 Jie, X. *et al.* Microwave-initiated catalytic deconstruction of plastic waste into hydrogen and high-value carbons. *Nat. Catal.* **3**, 902-912 (2020).

4 Gu, Z. *et al*. Cutting Single-Wall Carbon Nanotubes through Fluorination. *Nano Lett.* **2**, 1009-1013 (2002).

5 Li, W. *et al.* Promotion effect of cobalt doping on microwave-initiated plastic deconstruction for hydrogen production over iron catalysts. *App. Catal. B: Environ.* **327**, 122451 (2023).

6 Cao, Q. *et al.* Microwave-initiated MAX Ti_3AlC_2-catalyzed upcycling of polyolefin plastic wastes: Selective conversion to hydrogen and carbon nanofibers for sodium-ion battery. *App. Catal. B: Environ.* **318**, 121828 (2022).

7 Wang, J. *et al*. A high-quality hydrogen production strategy from waste plastics through microwave-assisted reactions with heterogeneous bimetallic iron/nickel/cerium catalysts. *J. Anal. Appl. Pyrol.* **166**, 105612 (2022).

8 Zhang, P. *et al.* High-efficient microwave plasma discharging initiated conversion of waste plastics into hydrogen and carbon nanotubes. *Energ. Convers. Manage.* **268**, 116017 (2022).

9 Yao, L. *et al.* Microwave-assisted decomposition of waste plastic over $Fe/FeAl_2O_4$ to produce hydrogen and carbon nanotubes. *J. Anal. Appl. Pyrol.* **165**, 105577 (2022).

10 Xie, M. *et al.* Constructing bifunctional porous nanosheets for efficient conversion of waste plastics into valuable hydrogen and carbons. *Chem. Eng. J.* **471**, 144460 (2023).

11 Yu, X. *et al.* Catalytic recycling of medical plastic wastes over $La_{0.6}Ca_{0.4}Co_{1-x}Fe_xO_{3-\delta}$ pre-catalysts for co-production of H_2 and high-value added carbon nanomaterials. *App. Catal. B: Environ.* **334**, 122838 (2023).

12 Yao, D. *et al.* Carbon nanotubes from post-consumer waste plastics: Investigations into catalyst metal and support material characteristics. *App. Catal. B: Environ.* **280**, 119413 (2021).

13 Cai, N. *et al.* Influence of the ratio of Fe/Al_2O_3 on waste polypropylene pyrolysis for high value-added products. *J. Clean. Prod.* **315**, 128240 (2021).

14 Wu, C. *et al*. Production and application of carbon nanotubes, as a co-product of hydrogen from the pyrolysis-catalytic reforming of waste plastic. *Process Saf. Environ.* **103**, 107-114 (2016).

15 Nahil, M. A. *et al*. Influence of metal addition to Ni-based catalysts for the

co-production of carbon nanotubes and hydrogen from the thermal processing of waste polypropylene. *Fuel Process. Technol.* **130**, 46-53 (2015).

16 Warren D. Seider, *et al*. *Product and Process Design Principles: Synthesis, Analysis, and Evaluation*. (2020).

17 Woods, D. R. *Rules of thumb in engineering practice*, *Appendix D: Capital Cost Guidelines*. John Wiley & Sons (2007).

18 Dai, Q. *et al.* EverBatt: A closed-loop battery recycling cost and environmental impacts model. (Argonne National Laboratory (ANL), Argonne, IL (United States), 2019).

19 Towler;, G. & Sinnott, R. *Chemical Engineering Design: Principles, Practice and Economics of Plant and Process Design*. Butterworth-Heinemann (2021).

20 Wang, M. *et al.* Complete hydrogenolysis of mixed plastic wastes. *Nat. Chem. Eng.* **1**, 376-384 (2024).

21 Kots, P. A. *et al*. A two-stage strategy for upcycling chlorine-contaminated plastic waste. *Nat. Sustain.* **6**, 1258-1267 (2023).

22 Gao, Z. *et al.* Room-temperature co-upcycling of polyvinyl chloride and polypropylene. *Nat. Sustain.* **7**, 1691-1698 (2024).

23 Cao, R. *et al.* Co-upcycling of polyvinyl chloride and polyesters. *Nat. Sustain.* **6**, 1685–1692 (2023).
