# Supplementary material for: ~100% upcycling of chlorinated/fluorinated plastic mixtures to H2 and nanotubes over FeNi/Ni/C by microwave catalysis
Source: Nat Commun. 2026 May 15;17:6481. doi: 10.1038/s41467-026-73141-w (PMC13376195; doi:10.1038/s41467-026-73141-w)
Supplement: Supplementary file 2 — Description of Additional Supplementary Files [file 41467_2026_73141_MOESM2_ESM.pdf]

Description of Additional Supplementary Files

Title: Supplementary Movie 1

Description: Supplementary Movie 1 demonstrates the decomposition process of plastic mixtures driven by microwave catalysis.
