# Supplementary material for: ~100% upcycling of chlorinated/fluorinated plastic mixtures to H2 and nanotubes over FeNi/Ni/C by microwave catalysis
Source: Nat Commun. 2026 May 15;17:6481. doi: 10.1038/s41467-026-73141-w (PMC13376195; doi:10.1038/s41467-026-73141-w)
Supplement: Supplementary file 4 — Transparent Peer Review file [file 41467_2026_73141_MOESM4_ESM.pdf]

nature portfolio

Peer Review File

~100% upcycling of chlorinated/fluorinated plastic mixtures to H2 and nanotubes over FeNi/Ni/C by microwave catalysis

Corresponding Author: Professor Junwang Tang

This file contains all reviewer reports in order by version, followed by all author rebuttals in order by version.

Version 0:

Reviewer comments:

Reviewer #1

(Remarks to the Author)
this paper introduced pyrolysis of cl/f contained plastics to produce h2 and carbon material by using FeNi/Ni@CNTs catalysts under microwave irradiation. impressive H2 purity and carbon conversion efficiency was reported. Revision is essential before further consideration of publication. following are several concerns should be addressed properly.
(1) how did author place the two layer of catalyst should in indicated in the diagram of, , the reaction instrument. such as the layer thickness and the gap length between two layers
(2) Also in the Supplementary Fig. 1, the size of the mixed plastics pieces was larger than the catalyst power. How did author mix them to ensure that all plastic can contact with catalyst during reaction?
(3) why heating temperature of bottom and upper layer given in Supplementary Fig. 10 was different? Is the curve belong to catalyst with or without CNTs? The fig. 10 was not mentioned in main content
(4) what's the contribution of these two catalyst layer during reaction?
(5) what's the ratio of the high-value CNTs in the decomposed carbon on the catalyst surface?
(6) as author mentioned, cl can help to reconstruct catalyst. But cl was released from PVC at around 280 oC, at such low temperature, how did the FeNi alloy form?
(7) in Line 337, as mentioned 20% of Cl and 2% of F was remained in CNTs. what' the chemical formation of these remained Cl and F? Will These Cl and F accumulate after 30 cycles usage?
(8) when calculating the energy efficiency, did author take the efficiency of electricity of microwave into consideration. How much microwave energy was absorbed by the samples?
(9) what's the length of the formed CNTs. was it changing during 30 cycles?
(10) as author mentioned, almost 100% of carbon was converted into CNTs, so what's the purity of the CNTs comparing to CNTs produced from CVD method?

Reviewer #2

(Remarks to the Author)
General Comments: This manuscript introduces a microwave-assisted thermolysis strategy employing an in-situ constructed FeNi/Ni@CNTs catalyst to efficiently decompose plastic waste mixtures into H2 and CNTs. Importantly, the process enables effective conversion of chlorinated/fluorinated plastic waste, which is normally difficult to recycle due to toxic gas formation. The authors show that Cl^- and F^- released from PVC and PTFE dynamically regenerate the catalyst active sites, thereby enhancing catalyst stability. Furthermore, a techno-economic analysis suggests that the approach may have potential commercial viability.
However, revisions and clarifications are needed in the catalyst characterization and activity evaluation sections, particularly regarding the explanation of the catalyst regeneration mechanism mediated by Cl^-. As this aspect represents the key novelty of the manuscript, a more comprehensive and detailed discussion is essential. While the study is certainly interesting, it currently has several critical shortcomings that must be addressed before it can be considered for publication in Nature Communications. Therefore, I recommend rejection of the manuscript in its current form.

1. How was the ratio between filamentous carbon and amorphous carbon quantitatively determined? (Fig. 2. c)
2. It is demonstrated that Cl^- derived from PVC adsorbs on Fe to form $FeCl_3$, which subsequently decomposes to facilitate FeNi alloy formation. However, the manuscript does not discuss the role of F^-. Could the authors clarify whether F^- plays an

analogous role in this regeneration mechanism?
3. In addition, the relationship between the formation of the Ni–Fe alloy and the catalytic activity toward H_2 production and CNTs growth remains unclear. Further explanation on this point appears necessary.
4. PVC and PTFE typically constitute only a minor fraction in real-world plastic waste streams. The catalytic activity appears to decrease in the absence of PVC, could the authors comment on whether similar performance can be expected at process scale given the much lower PVC content compared to laboratory-scale conditions?
5. Based on the unit of the turnover number (molcarbon mol-1Ni), it appears that the authors defined the active site as Ni. However, it is unclear why Fe was not considered. As discussed in the section on microwave catalysis mechanisms, both Ni and Fe act as active sites, and the catalytic activity varies depending on the Ni-to-Fe ratio in the catalyst. A related study published in Nature Catalysis, 2020, 3, 902–912, which investigated microwave-assisted conversion of plastic waste to H2 and CNTs using an Fe-based catalyst, also demonstrated that Fe plays a key role as an active site. The authors should clarify why Fe was excluded from the turnover number calculation in the present study.
6. In the reusability tests, it appears that the spent catalyst and solid carbon mixture from the previous reaction were not removed, and fresh plastic was simply added in each cycle. In this case, wouldn't the unreacted plastic from the previous cycle also be carried over into the subsequent one? In this case, can the yield and selectivity for each cycle be considered reliable? Since the initial state of each cycle is not well-defined, the reproducibility and accuracy of the reported values are questionable. Furthermore, after the first cycle, the top layer in the tandem system is no longer a fresh catalyst layer, but rather a spent catalyst mixed with solid carbon mixture. This may significantly deviate from the initially designed tandem system. Given this change in the system structure, it is important that the authors clearly explain how such deviations were addressed.
7. In the section Mechanism of catalyst regeneration by Cl^-, the FeC–FeNi/Ni@CNT (precursor) catalyst is introduced. However, it is unclear how this catalyst was prepared. It would be helpful if the authors could provide a description of the synthesis method used for this catalyst.
8. There appears to be a lack of consistency in the data related to the regenerated FeNi/Ni@CNT catalyst. In Fig. 2g–f, the regenerated catalyst corresponds to that obtained after 15 cycles, whereas in Fig. 3, the regenerated catalyst is from after only 5 cycles. Using regenerated catalysts from different cycles across analyses may significantly hinder the overall interpretation of the results. It is recommended that the authors ensure consistency in the characterization of regenerated catalysts throughout the manuscript.
9. Please provide additional EDS image related to Fig. 2f to confirm the uniform distribution of carbon throughout the FeC–FeNi/Ni@CNTs catalyst.
10. In the EXAFS analysis, it seems necessary to more clearly distinguish between Fe–Ni /Fe–C and Ni–Ni/Ni–Fe bonds. To achieve this, it would be beneficial to include WT-EXAFS data in addition to the FT-EXAFS results, as it may provide better resolution and allow for more precise identification of the coordination environments.
11. In Supplementary Fig. 23, the XRD data presented to demonstrate the formation of FeCl3 is not fully convincing for the main FeC–FeNi/Ni@CNT sample, as the broad CNT-related XRD peak spanning 15–35° obscures the FeCl3 peaks. Therefore, it is difficult to conclude that FeCl3 formation is dominant compared to NiCl2. This raises concerns about the adequacy of the presented analysis in supporting the claimed catalyst regeneration mechanism. Moreover, the core claim of this study—that Cl^- generated from PVC decomposition facilitates catalyst regeneration—appears to be insufficiently substantiated. While catalyst characterization before and after reaction is provided, there is a lack of real-time analysis linking Cl^- formation with structural changes in the catalyst. This gap limits the mechanistic understanding of the regeneration process.

Reviewer #3

(Remarks to the Author)
The presented work is interesting; however, the current form of this article requires that the following observation be addressed for publication.

The novelty of this work in terms of new mechanistic insight and comprehensive understanding of polymer degradation processes has not been included.
The abstract seems very superficial; it does not highlight innovative results that were obtained in summary. Although the title seems generic, the catalyst played a key role in this conversion process, so why wasn't it included?
The structure of introduction is very poor (there is no introduction), says existing histories of plastic degradation lacking an in-depth analysis that explains the innovative aspects of the present work.
The materials and methodology are presented in supplementary; it should be in the main article so that they can help researchers to reproduce.
What is 100% conversion equivalent to, what is defined as yield in terms of a specific compound?
The purification of FeNi-integrated carbon nanotubes is not discussed.
In general, the present work does not provide a deep insight into polymer degradation phenomena based on the basic science of polymers, as every polymer will degrade under elevated temperatures, high energy exposure, other radiation sources, or similar conditions. The current format of this article does not have the characteristics of a scientific article, but rather that of a technical report.

Version 1:

Reviewer comments:

Reviewer #1

(Remarks to the Author)
most previous raised problems have been addressed properly, except for the calculating of energy efficiency. The explanation is not convincible. The absorbed energy should be calculated from the aspect of sample instead of microwave, i.e. how much energy was used to heat up the sample and maintain the reaction, including latent heat, sensible heat and reaction heat. So maybe author should reconsider the energy calculation formula.

Reviewer #2

(Remarks to the Author)
General Comments: While the authors have addressed our previous comments to some extent, several points remain insufficiently resolved. Accordingly, substantial revisions are required before the manuscript can be considered for publication in Nature Communications.

1. Direct evidence demonstrates that the metal oxide surface is re-exposed due to the role of fluorine is required. The current explanation, which attributes this effect solely to CNT shearing, remains speculative and insufficiently supported. Given that a major strength of this work lies in the effective recycling of chlorinated and fluorinated plastics, a more rigorous and explicit discussion of this mechanism is necessary. In addition, when PTFE is used as the feedstock, the authors should clearly assign and explain each XRD peak observed for the FeC–FeNi/Ni/CNT catalyst, including what each peak represents.
2. The magnified XANES region (8340–8341 eV) shown in the inset does not correspond to the edge onset or the edge energy (E_0) of the Ni K-edge but rather lies outside the edge onset region (Fig. R17b). As a result, the relative spectral positions in this range cannot be reliably used to discuss oxidation state differences. In particular, the fact that the Ni foil does not appear at the lowest energy may lead to confusion. In addition, the authors attribute the observed behavior to electron transfer from Fe to Ni induced by the formation of NiFe species. However, based on the XANES results, both Fe and Ni appear to progressively approach a more metallic state. Such a trend does not support the presence of directional electron transfer between Fe and Ni. If electron transfer were occurring, one would expect Fe to become more electron-deficient while Ni becomes more electron-rich, a trend that is not observed in the present analysis. Therefore, the observed changes in the electronic states are more likely attributable to factors other than intermetallic electron transfer between Fe and Ni. This point requires clearer clarification and more rigorous discussion.
3. The EXAFS fitting results exhibit large uncertainties in the Debye–Waller factors (σ^2). For example, the reported value ($\sigma^2 = 0.093 \times 10^{-2}$ Å^2) has an uncertainty ($\pm 0.29 \times 10^{-2}$ Å^2) that significantly exceeds the fitted value itself, indicating that this parameter is not statistically well constrained and cannot be considered quantitatively meaningful. The authors are therefore requested to report the uncertainties for all other fitted parameters as well, to properly assess the robustness and reliability of the EXAFS analysis.
4. In the TGA data shown in Fig. R21, no distinct weight loss is observed for cycles 15 and 30. Based on the described reaction setup, the catalyst and feedstock are mixed during the reaction, and the conversion is evaluated by analyzing the compositional changes of the resulting mixture. This implies that unreacted plastic is recovered together with the catalyst after the reaction. Consequently, the post-reaction plastic mixture should contain catalyst components, including CNTs, for which a measurable weight loss would normally be expected during TGA analysis. The absence of any observable weight loss under these conditions is therefore unexpected and requires clarification.
5. In Fig. R30, the formation of metal chlorides is supported by color changes and XRD analysis. However, additional evidence would be beneficial to more convincingly demonstrate the formation of metal chlorides. In particular, emphasizing the yellow color alone primarily suggests the formation of Fe-based chlorides, and does not provide sufficient evidence for the presence of $NiCl_2$, which is typically associated with a greenish color. Therefore, color observation should be regarded only as a supplementary indicator for chloride formation. Moreover, the XRD patterns appear to contain multiple overlapping peaks, making it difficult to unambiguously identify the formation of individual chloride species. In this regard, XAFS analysis of the yellow crystalline phase could provide more definitive evidence for the formation of $FeCl_2$, $FeCl_3$, and $NiCl_2$, and is suggested to strengthen the conclusions.

Reviewer #3

(Remarks to the Author)
After reviewing the revised manuscript, I recommend the following correction before publication:

Indicate clearly where the Introduction section starts and place the results and discussion of this work under the Results and Discussion section.

Additionally, summarize the information in each section so that readers can grasp the key points of the work more quickly.

Version 2:

Reviewer comments:

Reviewer #2

(Remarks to the Author)
This manuscript has been substantially upgraded after including sufficient response to reviewer comments. This manuscript is considered suitable for publication in the journal.

Open Access This Peer Review File is licensed under a Creative Commons Attribution 4.0 International License, which permits use, sharing, adaptation, distribution and reproduction in any medium or format, as long as you give appropriate credit to the original author(s) and the source, provide a link to the Creative Commons license, and indicate if changes were made.
In cases where reviewers are anonymous, credit should be given to 'Anonymous Referee' and the source.
The images or other third party material in this Peer Review File are included in the article's Creative Commons license, unless indicated otherwise in a credit line to the material. If material is not included in the article's Creative Commons license and your intended use is not permitted by statutory regulation or exceeds the permitted use, you will need to obtain permission directly from the copyright holder.
To view a copy of this license, visit https://creativecommons.org/licenses/by/4.0/

We extend our sincere gratitude to the three reviewers for their invaluable comments. In response to this constructive feedback, we have conducted 25 new case studies and engaged in thorough discussions to address their concerns. We think that these efforts would significantly enhance the quality and scientific rigor of our work. All modifications are highlighted with a yellow background in both the main text and supplementary information.

Reviewer #1: this paper introduced pyrolysis of cl/f contained plastics to produce h2 and carbon material by using FeNi/Ni@CNTs catalysts under microwave irradiation. impressive H2 purity and carbon conversion efficiency was reported. Revision is essential before further consideration of publication. following are several concerns should be addressed properly.

1. how did author place the two layer of catalyst should in indicated in the diagram of, , the reaction instrument. such as the layer thickness and the gap length between two layers

Response: We sincerely thank the reviewer for proposing this insightful comment. The diagram of the two catalyst layers is provided in **Fig. R1**. The bottom layer, consisting of 3 g of plastic and 3 g of catalyst, has a thickness of 5 mm, while the top layer (3 g of catalyst alone) has a thickness of 2 mm; the distance between the two layers is 80 mm; the diameter of the reactor is 68 mm.

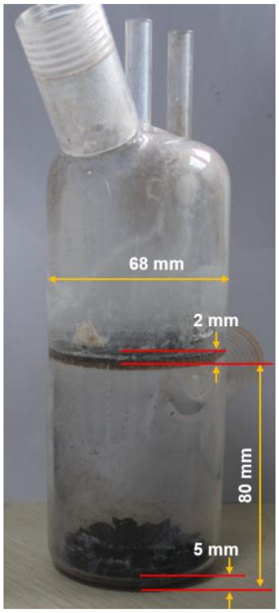

Fig. R1. Optical image of the reactor and material placement.

Fig. R1 has been included as Supplementary Fig. 1 in the supplementary material.

2. Also in the Supplementary Fig. 1, the size of the mixed plastics pieces was larger than the catalyst power. How did author mix them to ensure that all plastic can contact with catalyst during reaction?

Response: We appreciate the reviewer for pointing out this crucial issue. We apologize for the unclear description. To clarify, prior to mixing the catalyst with the plastic, the plastic feedstock was crushed into particles (<5 mm) and mechanically blended with the catalyst (**Fig. R2)**. Additionally, during the reaction, heat generated via selective interaction between the catalyst and microwaves is transferred to plastic particles, causing them melting. This shifts the reaction system from initial solid-solid contact to solid-liquid contact, thereby enhancing the contact efficiency between the plastic and catalyst (Nature Catalysis 3, 902-912 (2020). Nature Communications 16, 1726 (2025).).

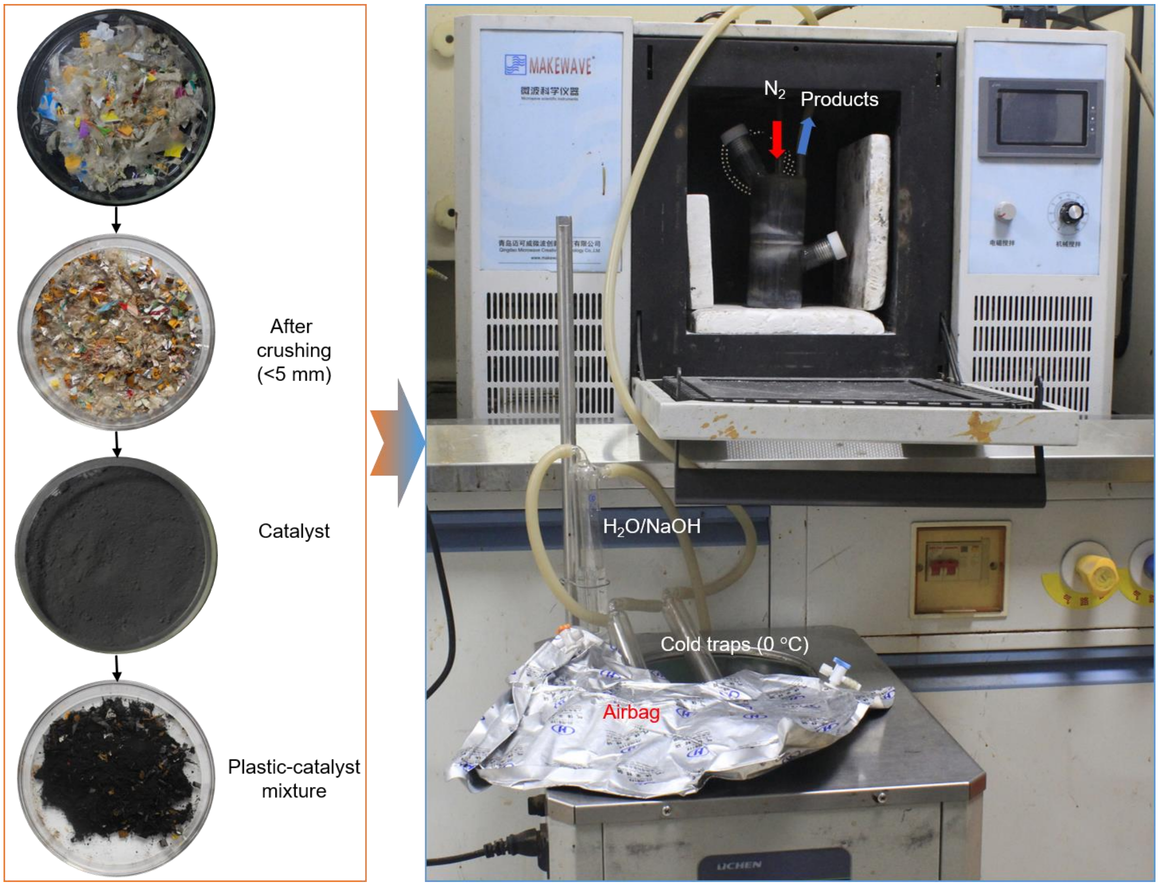

Fig. R2. The diagram of the reaction instrument.

Fig. R2 has been included as Supplementary Fig. 1 in the supplementary material.

3. why heating temperature of bottom and upper layer given in Supplementary Fig. 10 was different? Is the curve belong to catalyst with or without CNTs? The fig. 10 was not mentioned in main content

Response: Thank the reviewer for raising this key point. The heating curve was measured during reaction using a configuration with 3 g of plastic with 3 g of catalyst in the bottom layer and 3 g of catalyst alone in the top layer. Since the top layer contains only the catalyst, microwave-induced heat is concentrated there, whereas heat from the catalyst in the bottom layer is transferred to the plastic, with partial energy consumption during plastic catalytic conversion, resulting in slightly lower temperatures in the bottom layeras mentioned before (Nature Catalysis 3, 902-912 (2020)). That is, the catalyst alone can elevate the bed temperature to higher than that of the catalyst with plastic mixture. During reaction, plastic is catalytically converted into hydrogen and carbon nanotubes, with the latter remaining in the catalyst; thus, this heating curve corresponds to a catalyst containing carbon nanotubes. **Supplementary Fig. 10** was updated as **Supplementary Fig. 14,** and the related description is mentioned on **page 6** and **page 15** in the revised manuscript.

4. what's the contribution of these two catalyst layer during reaction?

Response: We thank the reviewer for raising the valuable comment. The contribution of tandem catalysis can be explained as follows: a bottom catalyst layer first decomposes plastics into hydrogen and carbon, with accompanying C-C bond cleavage generating small-molecule hydrocarbons that escape with hydrogen. These small-molecule hydrocarbons are then further dehydrogenated by the top catalyst layer, thereby enhancing hydrogen purity, hydrogen efficiency, and carbon efficiency.

To demonstrate the superiority and functionality of tandem design, 30 new successive cycles of decomposition experiments were conducted using an LDPE and PVC mixture (LDPE: PVC = 9:1) under two conditions: with the tandem design and with only the bottom layer. In the tandem design, the catalytic system exhibits consistent performance: over 95 wt% of the plastic mixture is converted into gas and solid carbon in each cycle, with only small amounts of organic by-products generated. H_2 efficiency ranges between 85.7% and 98.2% with a purity of 89.4-97.0 vol%, while carbon efficiency ranges from 83.3% to 99.9%. Corresponding yields of H_2 and CNTs reach 930 mmol $g^{-1}_{catalyst}$ and 10608 mg $g^{-1}_{catalyst}$, respectively (**Fig. R3**).

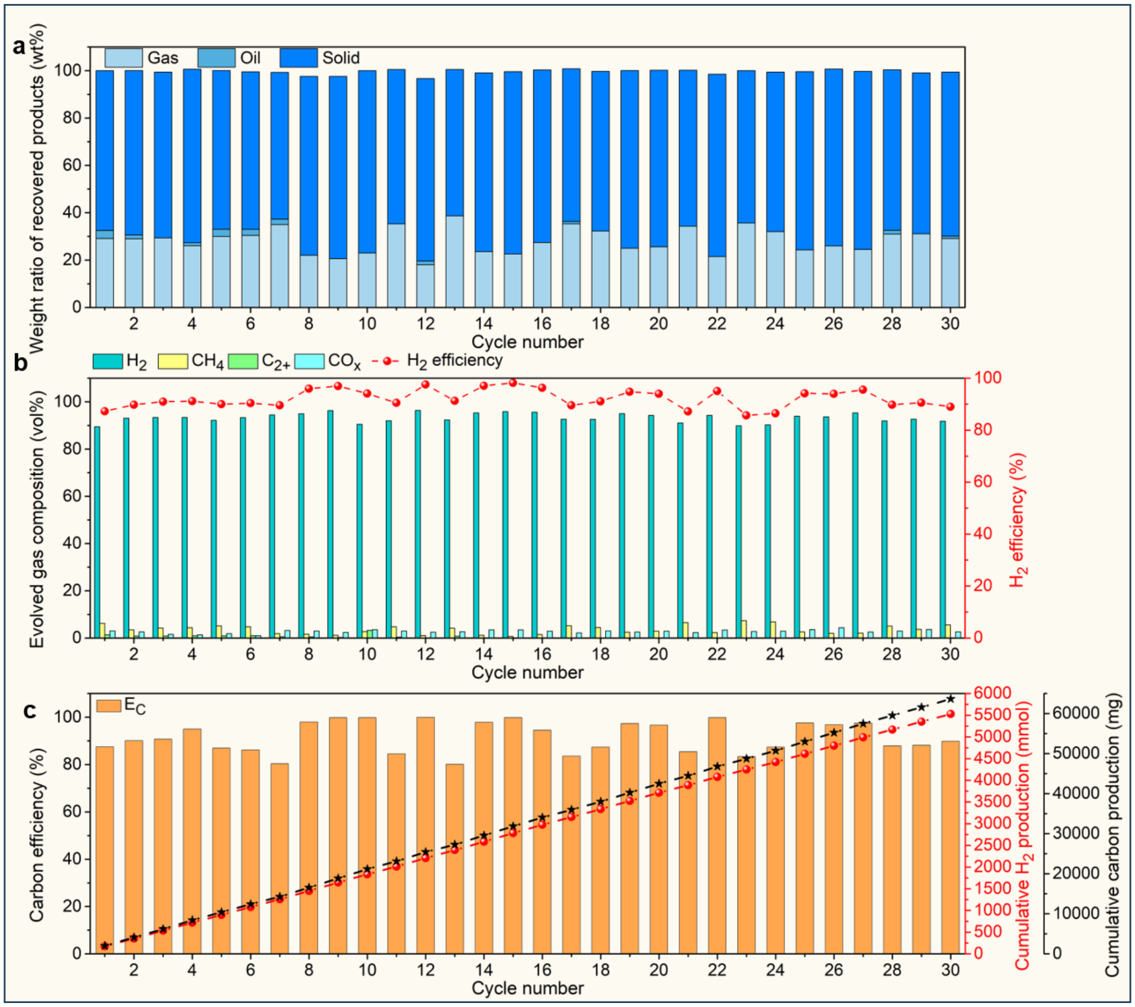

Fig. R3. Successive cycles of microwave catalytic decomposition of plastic over FeNi/Ni/CNTs catalyst. (a) The weight ratio of recovered gas, oil and solid. (b) Evolved gas composition (vol%) and H_2 efficiency (%). (c) Carbon efficiency (%) (E_C), cumulative H_2 production (mmol) and cumulative carbon production (mg). Reaction conditions: 3 g plastic and 3 g FeNi/Ni/CNTs catalyst in the bottom layer; additional 3 g FeNi/Ni/CNTs catalyst in the top layer. Plastic: LDPE and PVC mixture (the mass ratio of LDPE to PVC is 9:1).

In contrast, in the presence of the bottom layer, hydrogen efficiency is 67-92% (purity: 87-96 vol%) and carbon efficiency is 63-89%. The respective H_2 and carbon yields are 812.9 mmol $g^{-1}_{catalyst}$ and 8743 mg $g^{-1}_{catalyst}$, which are significantly lower than those of the tandem design (**Fig. R4**). Air atmosphere thermogravimetric analysis reveals increased carbon content in both top and bottom catalyst layers with cycling, suggesting the top catalyst facilitates dehydrogenation of hydrocarbons generated by the bottom catalyst, improving the catalytic efficiency (**Fig. R5**). In addition, thermogravimetric mass spectrometry (TG-

MS) results further support the above discussion: relative to the tandem catalytic mode, the standalone catalyst-plastic mixture mode produces less hydrogen and more hydrocarbons in the 10-90 m/z range (**Fig. R6**). This further indicates that the tandem mode can further catalyze the hydrocarbons generated during the reaction in the bottom layer, thereby enhancing hydrogen and carbon efficiency.

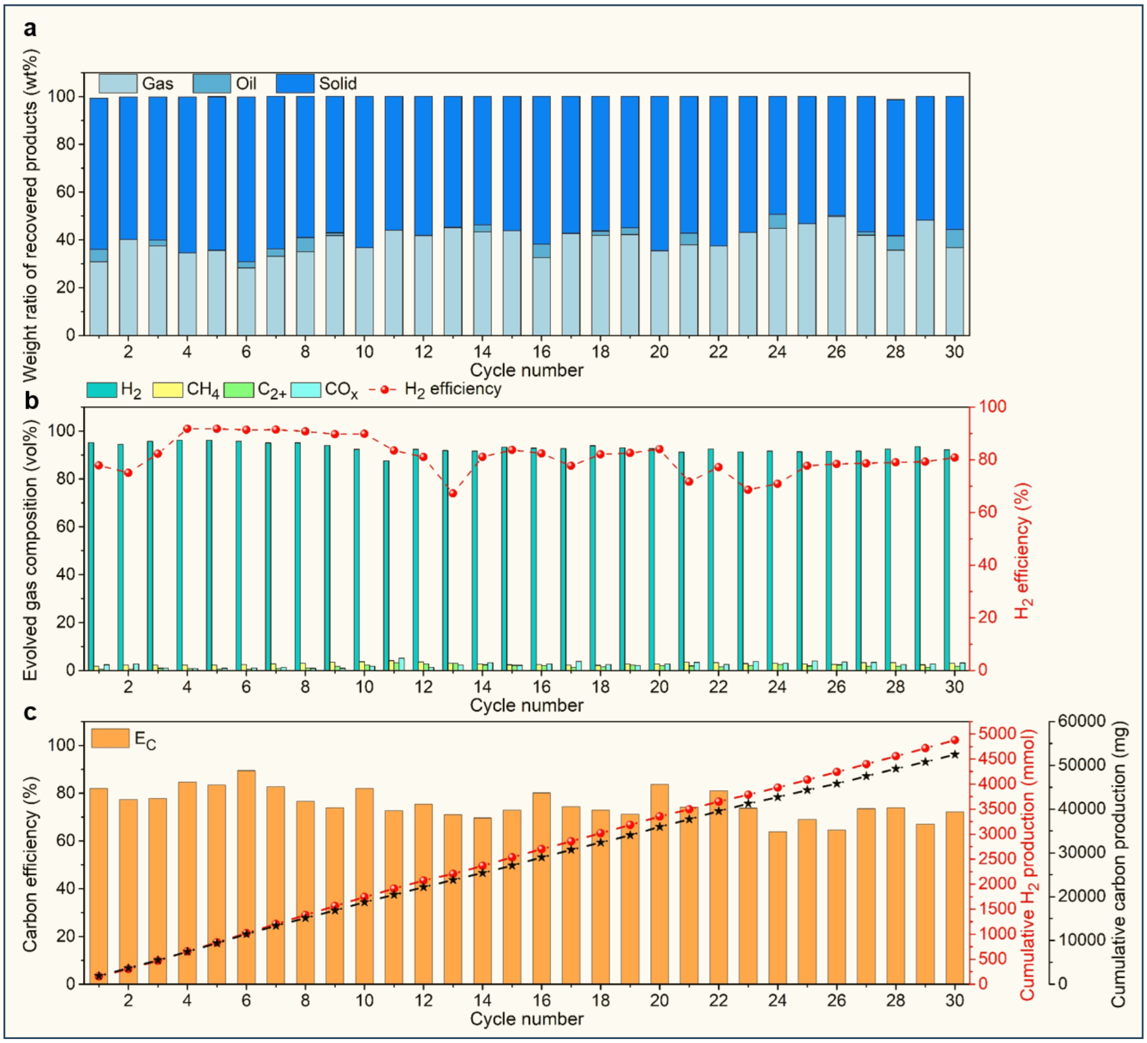

Fig. R4. Successive cycles of microwave catalytic decomposition of plastic over FeNi/Ni/CNTs catalyst. (a) The weight ratio of recovered gas, oil and solid. (b) Evolved gas composition (vol%) and H_2 efficiency (%). (c) Carbon efficiency (%) (E_C), cumulative H_2 production (mmol) and cumulative carbon production (mg). Reaction conditions: A mixture of 3 g plastic and 6 g FeNi/Ni/CNTs catalyst. Plastic: LDPE and PVC mixture (the mass ratio of LDPE to PVC is 9:1).

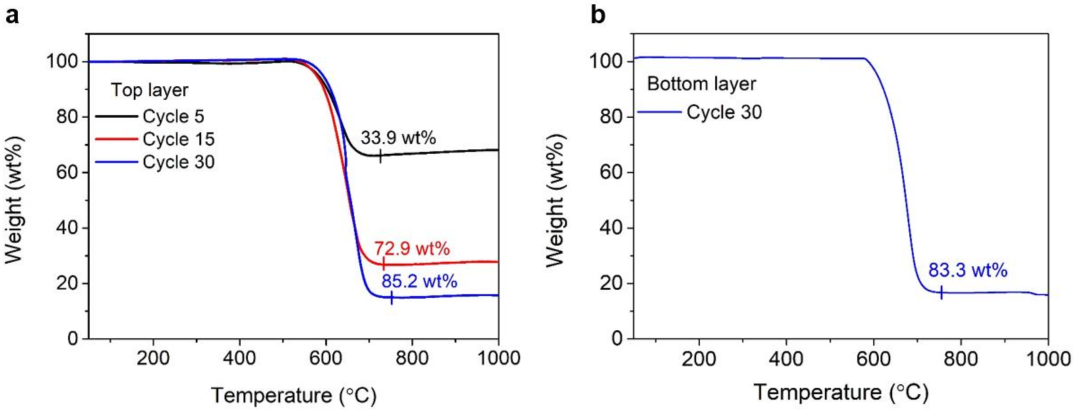

Fig. R5. Thermogravimetric analysis of the top (a) and bottom (b) catalyst after use.

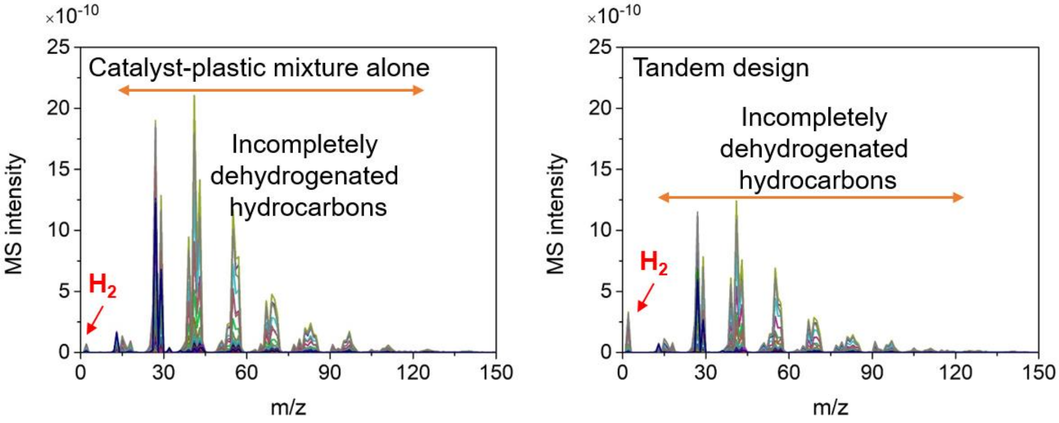

Fig. R6. TG-MS analysis of the differences in the product distribution of only catalyst-plastic mixture reaction modes and tandem catalytic modes.

Figs. R3-6 have been marked as Fig. 1, Supplementary Figs. 8-10 in the manuscript and supplementary material.

5. what's the ratio of the high-value CNTs in the decomposed carbon on the catalyst surface?

Response: We sincerely appreciate the reviewer's constructive comment. As reported in the literature (Nature Catalysis 3, 902-912 (2020), Proceedings of the National Academy of Sciences 120, e2305078120 (2023), Carbon 46, 2003-2025 (2008), Applied Catalysis B: Environmental 318, 121828 (2022)), thermogravimetric analysis (TGA) is a commonly employed method for the quantitative evaluation of carbon nanotube quality. Through the combustion of carbon nanotube samples in air and analyzing oxidation temperature curves, TGA enables the direct determination of the respective ratios of carbon nanotubes and

amorphous carbon. This is because carbon nanotubes with higher structural stability exhibit oxidation temperatures exceeding 535 °C.

As shown in **Fig. R7**, the carbon content in the used catalyst reaches 94.5 wt%, 83.3 wt%, 94 wt%, and 89 wt% after successive cycles using LDPE or PVC, LDPE-PVC blend, LDPE-HDPE-PP-PS-PVC-PTFE blend, and landfill mixed plastics as feedstocks, respectively. Furthermore, TGA oxidation temperature curves were employed to determine the ratio of carbon nanotubes (oxidation temperatures > 535 °C) to amorphous carbon (oxidation temperatures < 535 °C) in the products. Carbon nanotubes account for 99.1% in the resulting carbon products after successive catalytic cycles with LDPE or PVC as feedstock, 99.3% with LDPE-PVC blend as feedstock, 97.3% with LDPE-HDPE-PP-PS-PVC-PTFE blend as feedstock, and 98.5% with landfill mixed plastics as feedstock. These results demonstrate that this microwave catalytic strategy enables the conversion of various plastic waste into high-purity carbon nanotubes.

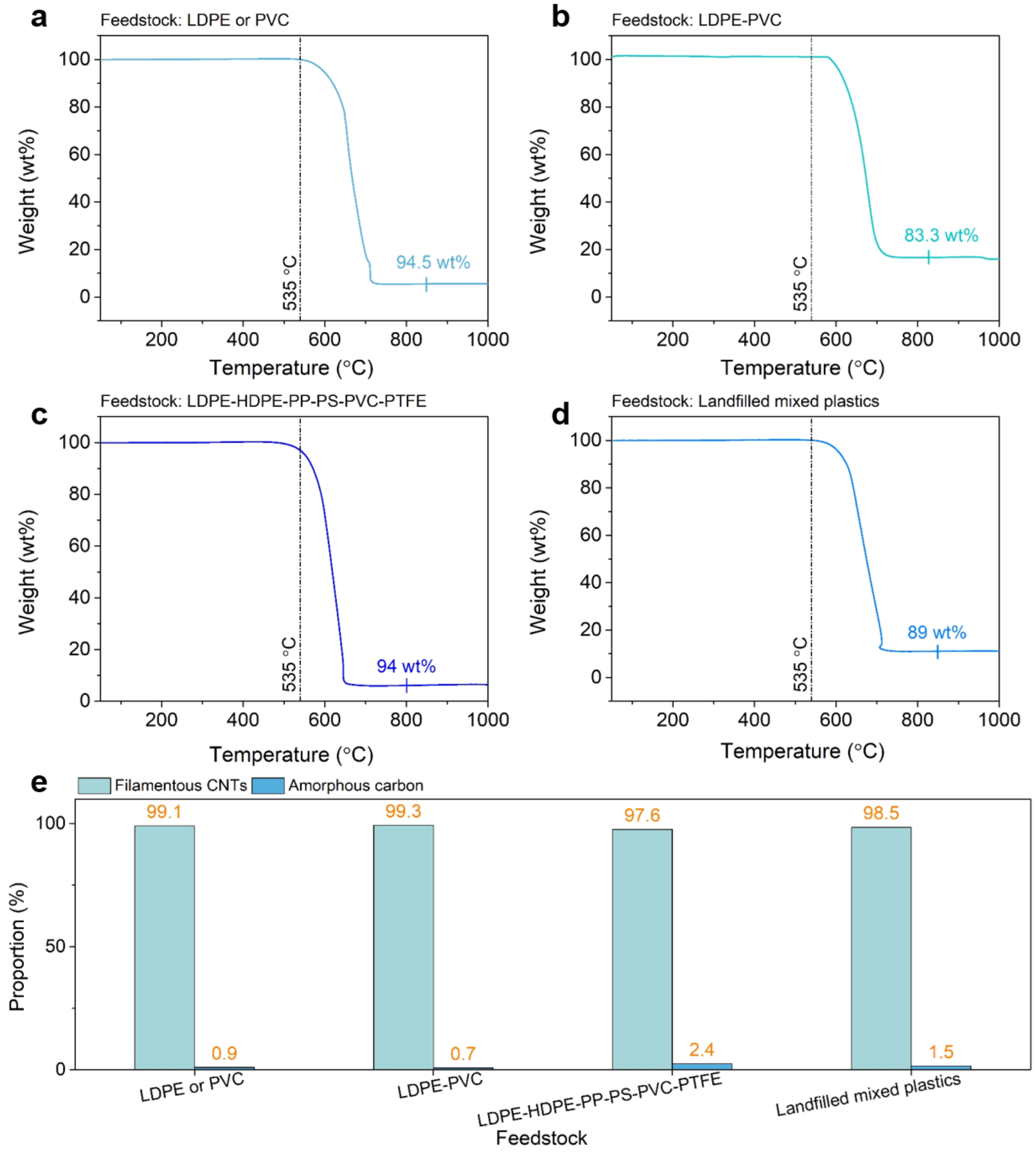

Fig. R7. TGA curves and the proportion of different types of carbon. (a) The weight ratio of carbon in the used catalyst after 35 successive catalytic cycles, using LDPE (Cycle 1-Cycle 25, Cycle 30-Cycle 35) or PVC (Cycle 26-Cycle 30) as the feedstock. Reaction condition: 3 g plastic and 3 g FeNi/Ni/CNTs catalyst in the bottom layer; 3 g FeNi/Ni/CNTs catalyst in the top layer. (b) The weight ratio of carbon in the used catalyst after 30 successive catalytic cycles, using the LDPE-PVC mixture (mass ratio of 9:1) as the feedstock. Reaction condition: 3 g plastic and 3 g FeNi/Ni/CNTs catalyst in the bottom layer; 3 g FeNi/Ni/CNTs catalyst in the top layer. (c) The weight ratio of carbon in the used catalyst after 5 successive catalytic cycles, using the LDPE-HDPE-PP-PS-PVC-PTFE mixture as the feedstock. Plastic mixtures of LDPE, HDPE, PP, PS, PVC and PTFE with mass ratios of 875:615:965:330:510:12. Reaction condition: 30 g plastic and 1.5 g

FeNi/Ni/CNTs catalyst in the bottom layer; 1.5 g FeNi/Ni/CNTs catalyst in the top layer. (d) The weight ratio of carbon in the used catalyst after 35 successive catalytic cycles, using the landfilled mixed plastics as the feedstock. Feedstock: PVC and landfilled plastic waste mixtures with a mass ratio of 1:9 (Cycle 1-Cycle 30), PTFE and landfilled plastic waste mixtures with a mass ratio of 1:9 (Cycle 31-Cycle 35). Reaction condition: 3 g plastic and 3 g FeNi/Ni/CNTs catalyst in the bottom layer; 3 g FeNi/Ni/CNTs catalyst in the top layer. (e) The proportion of filamentous CNTs and amorphous carbon in carbon materials obtained from the catalytic decomposition of different plastic feedstock.

Fig. R7 has been included as Supplementary Fig. 52b, Supplementary Fig. 48b, Supplementary Fig. 61b, and Supplementary Fig. 55b in the supplementary material.

6. as author mentioned, cl can help to reconstruct catalyst. But cl was released from PVC at around 280 oC, at such low temperature, how did the FeNi alloy form?

Response: We thank the reviewer for proposing this key point. The reconstruction of FeNi alloy in the PVC-assisted FeC-FeNi/Ni/CNTs catalyst follows the procedure: hydrogen generated during plastic decomposition reduces the catalyst to produce water molecules, which combine with hydrogen chloride from PVC decomposition to form hydrochloric acid molecules. These hydrochloric acid molecules thereby effectively convert Ni and Fe into nickel chloride and iron chloride. Subsequently, the formed nickel chloride and iron chloride are reduced by hydrogen, thereby facilitating the formation of FeNi alloy (**Page 9**).

This discussion is supported by the following new studies **(Figs. 8-9)**. **Fig. R8** presents the H_2-TPR-MS results of the FeC-FeNi/Ni/CNTs catalyst precursor, where the TPR profile and mass spectrometry data indicate that this precursor is reduced below 300 °C and water molecules form during hydrogen reduction, supporting hydrogen's role as a reducing medium in the reaction.

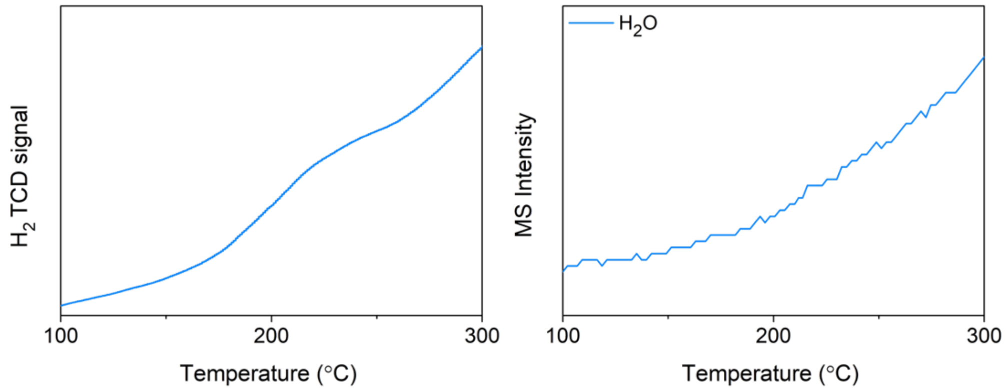

Fig. R8. The H_2-TPR-MS results of the FeC-FeNi/Ni/CNTs catalyst precursor.

To further verify that PVC decomposition can promote the formation of iron chlorides and nickel chlorides, the following new experiment was conducted: 5 g of FeC-FeNi/Ni/CNTs and 5 g of PVC were mixed uniformly and reacted under 600 W microwave power for 10 minutes. Subsequently, 30 mL of deionized water was added and stirred at 25 °C for 2 hours. Afterwards, the above mixture was filtered, and the filtrate was dried completely in an oven at 100 °C, ultimately obtaining yellow crystals (**Fig. R9a**). XRD results of the yellow crystals indicate that they are composed of $NiCl_2$ and FeClx, directly demonstrating that PVC decomposition does promote the formation of $NiCl_2$ and FeClx (**Fig. R9b**).

To verify that $NiCl_2$ and FeClx can form FeNi alloy under hydrogen, 0.3 g of yellow crystals and 0.1 g of commercial multi-walled carbon nanotubes were mixed uniformly and heated under 600 W microwave power and 10% H_2 atmosphere (flow rate 300 ml/min) for 10 minutes. XRD analysis confirms the formation of FeNi alloy (**Fig. R9c**). Meanwhile, treatment of the obtained yellow crystals by H_2-TPR-MS reveals the production of HCl, which is consistent with the aforementioned de-chlorine process of $NiCl_2$ and FeClx under hydrogen (**Fig. R9d**). These results support the above discussion regarding the PVC-assisted reconstruction of the catalyst to form FeNi alloy.

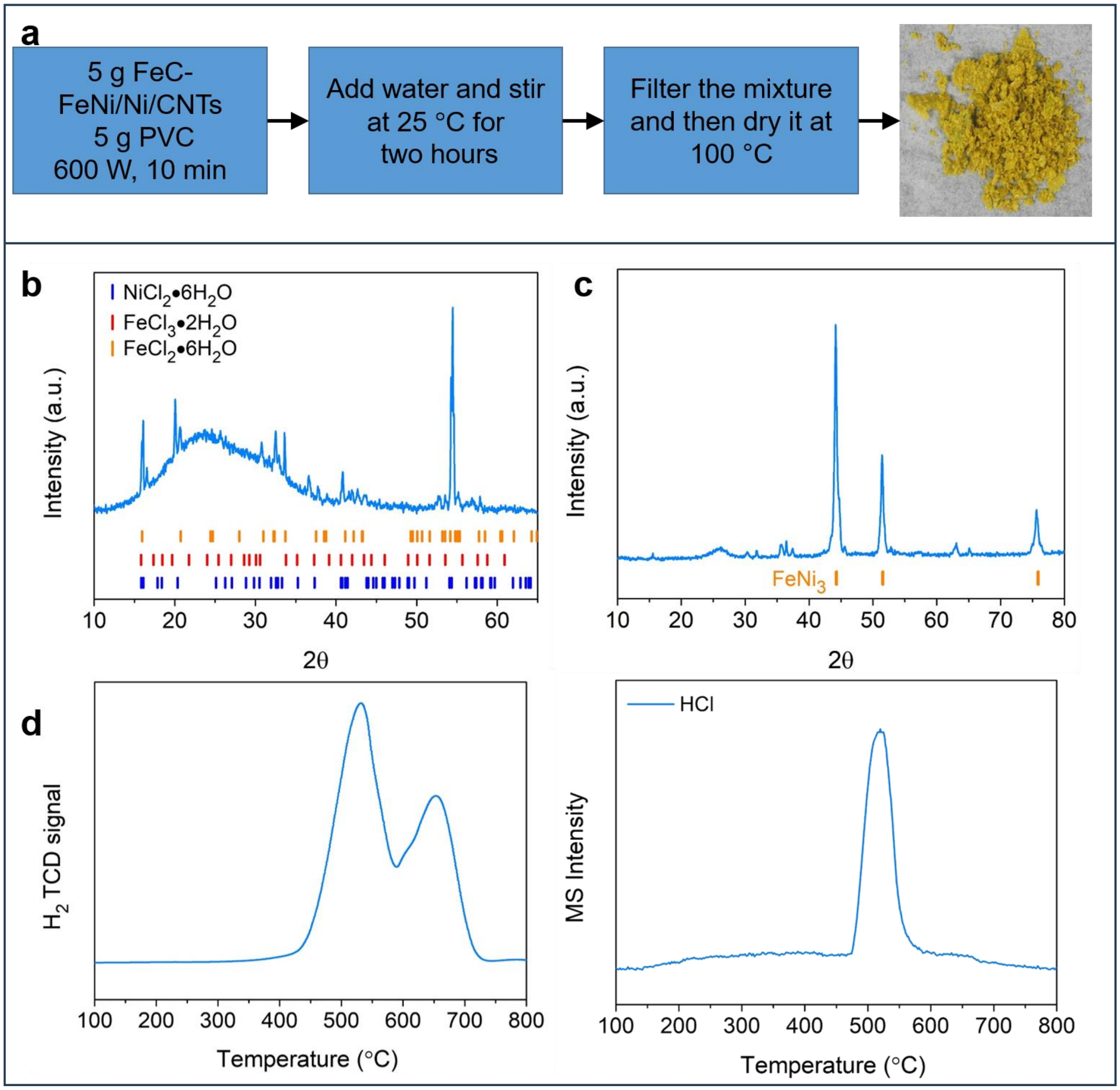

Fig. R9. (a) Schematic diagram of FeC-FeNi/Ni/CNTs and PVC reaction and post-treatment. (b) XRD results of the yellow crystals. (c) XRD results of the yellow crystals after being treated with H_2. (d) The H_2-TPR-MS results of the yellow crystals.

Figs. R8-9 have been marked as Supplementary Figs. 28-29 in the supplementary material.

7. in Line 337, as mentioned 20% of Cl and 2% of F was remained in CNTs. what' the chemical formation of these remained Cl and F? Will These Cl and F accumulate after 30 cycles usage?

Response: We appreciate the reviewer for raising this important point and apologize for the incomplete description in the previous version regarding Cl and F analysis in CNTs.

The previously reported values were derived solely from the sample after the first catalytic cycle, leading to an uncomplete results. Following this comment, Cl and F amounts in the samplesafter the 30th catalytic cycle were analyzed. As shown in **Fig. R10**, the results indicate that the Cl content is 0.17% after 30 cycles. No F is detected in the catalyst, indicating that F left the reaction system and does not remain in the catalyst. The low Cl content is likely due to a combination of hydrogen and chlorine from plastic decomposition to form hydrogen chloride, which is adsorbed on the catalyst, consistent with the mechanism proposed in **Comment 6**. Additionally, over 99% of Cl and 99.4% of F released from the reaction system were captured by a 1 M NaOH solution, thus preventing their emission into the environment (**Fig. R10**).

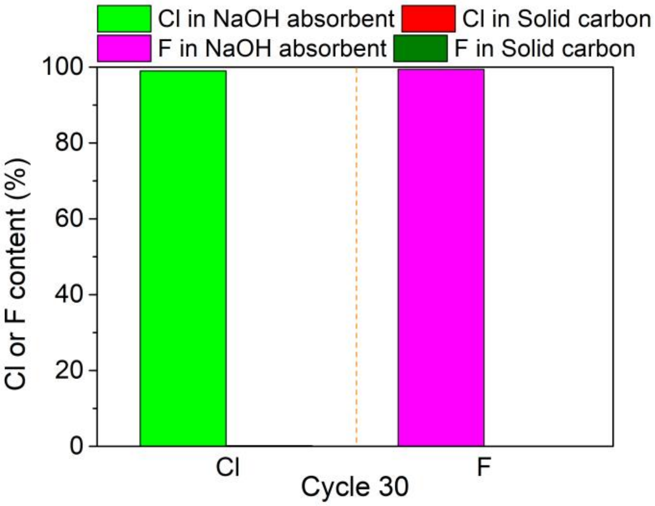

Fig. R10. The distribution of Cl and F after the microwave catalytic decomposition of LDPE-HDPE-PP-PS-PVC-PTFE plastic mixtures over the FeNi/Ni/CNTs catalyst during the 30th cycle.

Fig. R10 has been included as Supplementary Fig. 39 in the supplementary material and the following discussion was added on page 10 in the revised manuscript.

X-ray fluorescence (XRF) analysis indicates that the Cl content is 0.17% after 30 cycles. No F is detected in the catalyst and no Cl and F accumulation is observed, indicating that F left the reaction system and does not remain in the catalyst. Additionally, over 99% of Cl and 99.4% of F released from the reaction system were captured by a 1 M NaOH solution, thus preventing their emission into the environment.

8. when calculating the energy efficiency, did author take the efficiency of electricity of microwave into consideration. How much microwave energy was absorbed by the samples?

Response: We appreciate the reviewer for pointing out the critical issue. Electrical-to-microwave conversion efficiency has been considered when comparing electricity consumption between traditional thermal catalysis and microwave catalysis processes, as the measured electricity consumption reflects the total energy consumed during microwave reactor operation. As shown in **Fig. R11**, the applied microwave power was 600 W, whereas the total electricity power used was 1031.29 W, encompassing power consumption from all reaction systems, including the microwave generator, cooling device, and control system.

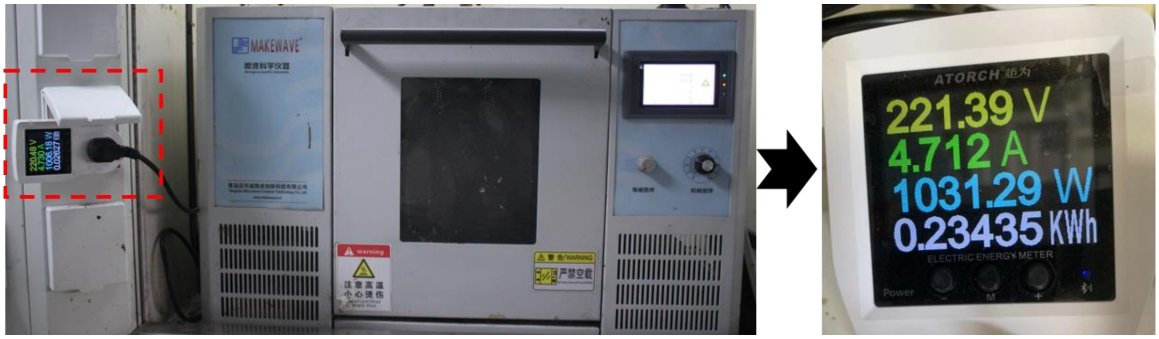

Fig. R11. Measurement method of electricity consumption in microwave catalysis.

Microwave absorption efficiency of the reaction material mixture is widely determined via real-time measurement of the voltage standing wave ratio (VSWR) (https://www.antenna-theory.com/definitions/vswr.php). VSWR, a key indicator of impedance matching between a transmission line (e.g., waveguide) and a load (e.g., absorbing material), approaches 1 (ideal state) under optimal matching with minimal reflected waves. Increased VSWR indicates a significant impedance mismatch, resulting in higher microwave reflection and lower absorption.

Fig. R12 presents a new study on the microwave absorption efficiency of the reaction material mixture. As shown in **Fig. R12a**, VSWR, input microwave power (Po), and reflected microwave power (Pr) are denoted accordingly. **Fig. R12b** illustrates the relationship between various VSWR values, reflected power, and absorbed power, where absorbed power (%) =100 - reflected power (%). **Fig. R12c** presents time-dependent VSWR values of the material after different cycles, allowing the microwave absorption efficiency of the material to be derived for each cycle. As shown in **Fig. R12d**, microwave absorption efficiency of the reaction material increases gradually from 40% to ~80% in the first catalytic cycle, stabilizing at ~90% in cycles 5, 15, and 30. This improvement arises from efficient catalytic decomposition of the LDPE-PVC (9:1) mixture into hydrogen and carbon nanotubes by FeNi/Ni/CNTs catalysts. The carbon nanotubes, effective microwave

absorbers, in turn enhance the microwave absorption properties of the catalyst system (Nature Catalysis 3, 902-912 (2020). Carbon, 46, 1935-1941 (2008).).

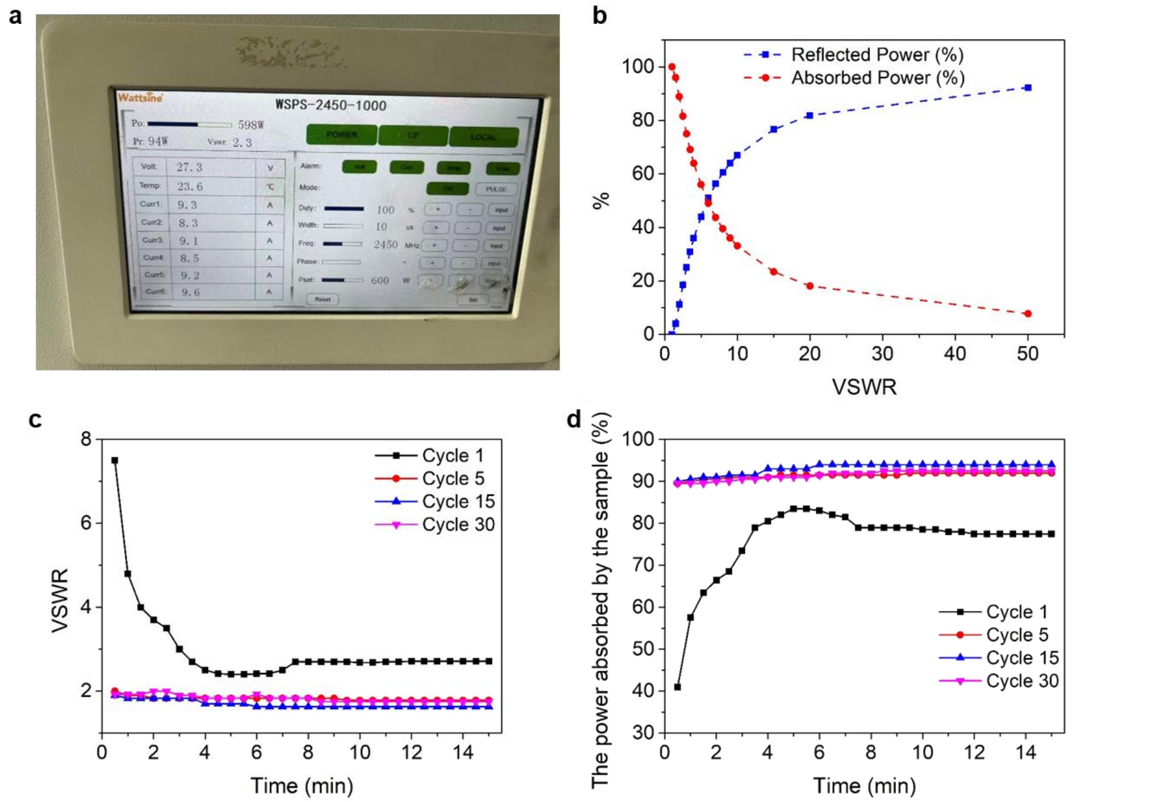

Fig. R12. Microwave absorption efficiency measurement (a) and results (b-d).

Fig. R11 has been included as Supplementary Fig. 13 in the supplementary material.

9. what's the length of the formed CNTs. was it changing during 30 cycles?

Response: Thank the reviewer for pointing out this comment. To determine the length of the generated CNTs, new TEM and AFM measurements were conducted. It should be noted that the measured length of CNTs is underestimated due to strong CNT interaction forces (e.g., van der Waals forces), which hinder dispersion, particularly for longer CNTs. Nonetheless, the length distribution of CNTs obtained after different cycles was measured.

CNTs from the first cycle exhibit a length distribution of 0.15-2.55 μm, with an average length of 0.48 μm (**Fig. R13a**). For CNTs from 30 cycles, TEM measurements reveal a length distribution of 0.3-1.5 μm and an average length of 0.67 μm, higher than that of first-cycle CNTs (**Fig. R13b**). Similarly, AFM characterization confirms a length

distribution of 0.44-1.44 μm, which is largely consistent with TEM results (**Fig. R13c**). This new information about the CNT length was added to **page 7** in the revised manuscript.

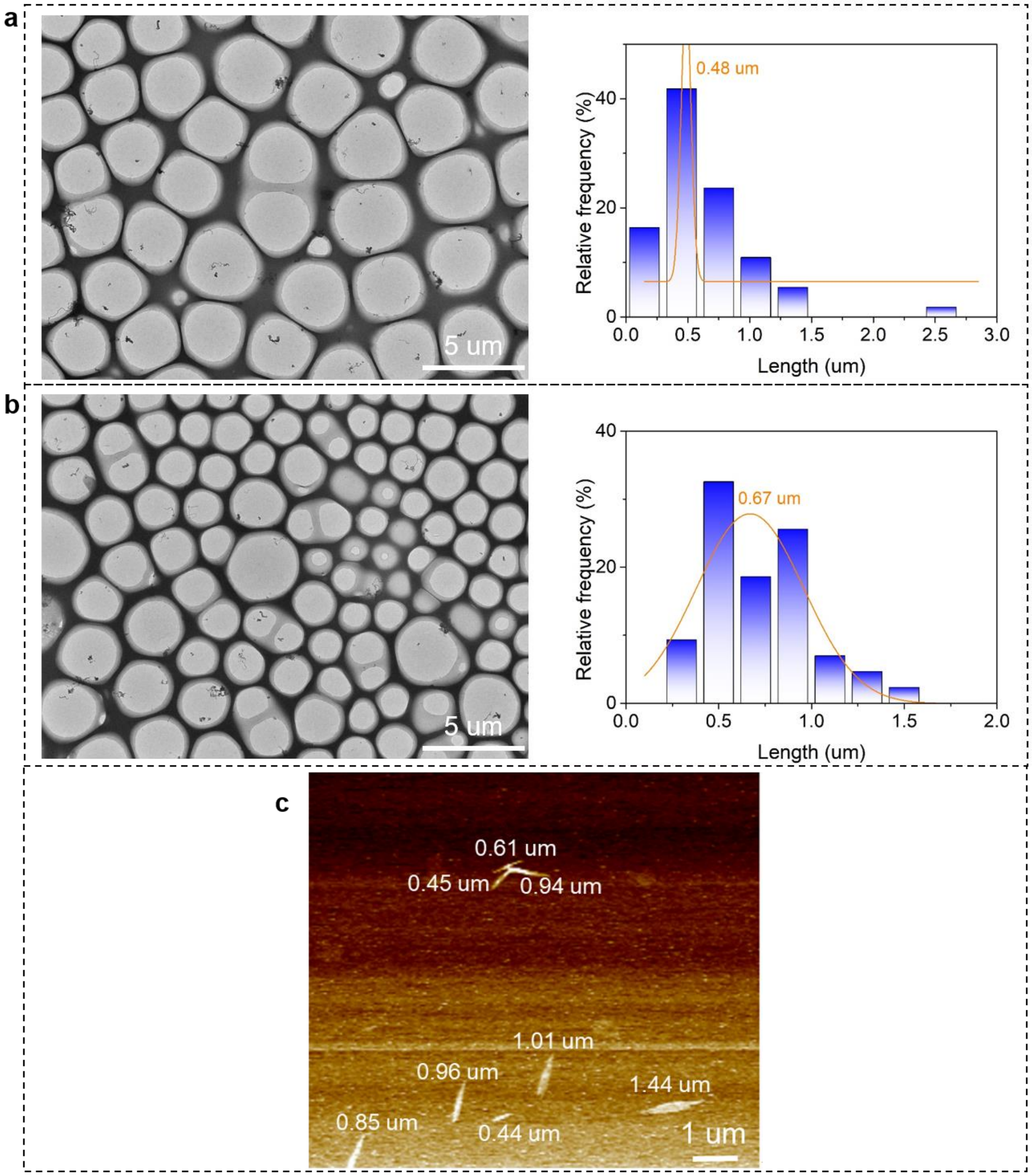

Fig. R13. TEM analysis of the length distribution of CNTs obtained from cycle 1 (a) and cycle 30 (b). AFM analysis of the length distribution of CNTs obtained from cycle 30 (c).

Fig. R13 has been included as Supplementary Fig. 16 in the supplementary material.

10. as author mentioned, almost 100% of carbon was converted into CNTs, so what's the purity of the CNTs comparing to CNTs produced from CVD method?

Response: We appreciate the reviewer for raising this important point. As shown in **Table R1**, CNTs produced via CVD exhibit a purity range of 75-96.3 wt%, while those generated in this study have a purity of 82.7-93.6 wt%. These results collectively indicate that the CNTs prepared in this study have purity comparable to those synthesized via CVD. Additionally, plastic waste is utilized as a carbon source here instead of the fossil resources typically employed in CVD, facilitating carbon source substitution for CNT synthesis and offering dual benefits: effective plastic waste management and fossil resource conservation.

Table R1 Comparison of CNTs produced by CVD with CNTs derived from LDPE, LDPE-PVC, LDPE-HDPE-PP-PS-PVC-PTFE, and landfill waste plastic

Strategy	Carbon source	Carbonaceous content (wt%)	CNTs purity (wt%)	Ref.
CVD	Camphor or acetylene	89.9<br>94.1	87.6<br>92.8	Journal of Cleaner Production 129 (2016) 384-394
CVD	Acetylene	97.7	96.3	Chemical Engineering Science 134 (2015) 496-503
CVD	Methane	95	75	Chemical Engineering Journal 377 (2019) 1198
This work	LDPE	94.5	93.6	This work
	LDPE-PVC	83.3	82.7	This work
	LDPE-HDPE-PP-PS-PVC-PTFE	94	91.7	This work
	Landfilled mixed plastics	89	87.7	This work

Reviewer #2: General Comments: This manuscript introduces a microwave-assisted thermolysis strategy employing an in-situ constructed FeNi/Ni@CNTs catalyst to efficiently decompose plastic waste mixtures into H2 and CNTs. Importantly, the process enables effective conversion of chlorinated/fluorinated plastic waste, which is normally difficult to recycle due to toxic gas formation. The authors show that Cl^- and F^- released from PVC and PTFE dynamically regenerate the catalyst active sites, thereby enhancing catalyst stability. Furthermore, a techno-economic analysis suggests that the approach may have potential commercial viability.

However, revisions and clarifications are needed in the catalyst characterization and activity evaluation sections, particularly regarding the explanation of the catalyst regeneration mechanism mediated by Cl^-. As this aspect represents the key novelty of the manuscript, a more comprehensive and detailed discussion is essential. While the study is certainly interesting, it currently has several critical shortcomings that must be addressed before it can be considered for publication in Nature Communications. Therefore, I recommend rejection of the manuscript in its current form.

1. How was the ratio between filamentous carbon and amorphous carbon quantitatively determined? (Fig. 2. c)

Response: We sincerely appreciate the reviewer's constructive comment. As reported in the literature (Nature Catalysis 3, 902-912 (2020), Proceedings of the National Academy of Sciences 120, e2305078120 (2023), Carbon 46, 2003-2025 (2008), Applied Catalysis B: Environmental 318, 121828 (2022)), thermogravimetric analysis (TGA) is a commonly employed method for the quantitative evaluation of carbon nanotube quality. Through the combustion of carbon nanotube samples in air and analyzing oxidation temperature curves, TGA enables the direct determination of the respective ratios of carbon nanotubes and amorphous carbon. This is because carbon nanotubes with higher structural stability exhibit oxidation temperatures exceeding 535 °C.

As shown in **Fig. R14**, the carbon content in the used catalyst reaches 94.5 wt%, 83.3 wt%, 94 wt%, and 89 wt% after successive cycles using LDPE or PVC, LDPE-PVC blend, LDPE-HDPE-PP-PS-PVC-PTFE blend, and landfill mixed plastics as feedstocks, respectively. Furthermore, TGA oxidation temperature curves were employed to determine the ratio of carbon nanotubes (oxidation temperatures > 535 °C) to amorphous carbon (oxidation temperatures < 535 °C) in the products. Carbon nanotubes account for 99.1% in the resulting carbon products after successive catalytic cycles with LDPE or PVC as feedstock, 99.3% with LDPE-PVC blend as feedstock, 97.6% with LDPE-HDPE-PP-PS-PVC-PTFE blend as feedstock, and 98.5% with landfill mixed plastics as feedstock. These results demonstrate that this microwave catalytic strategy enables the conversion of various plastic waste into high-purity carbon nanotubes.

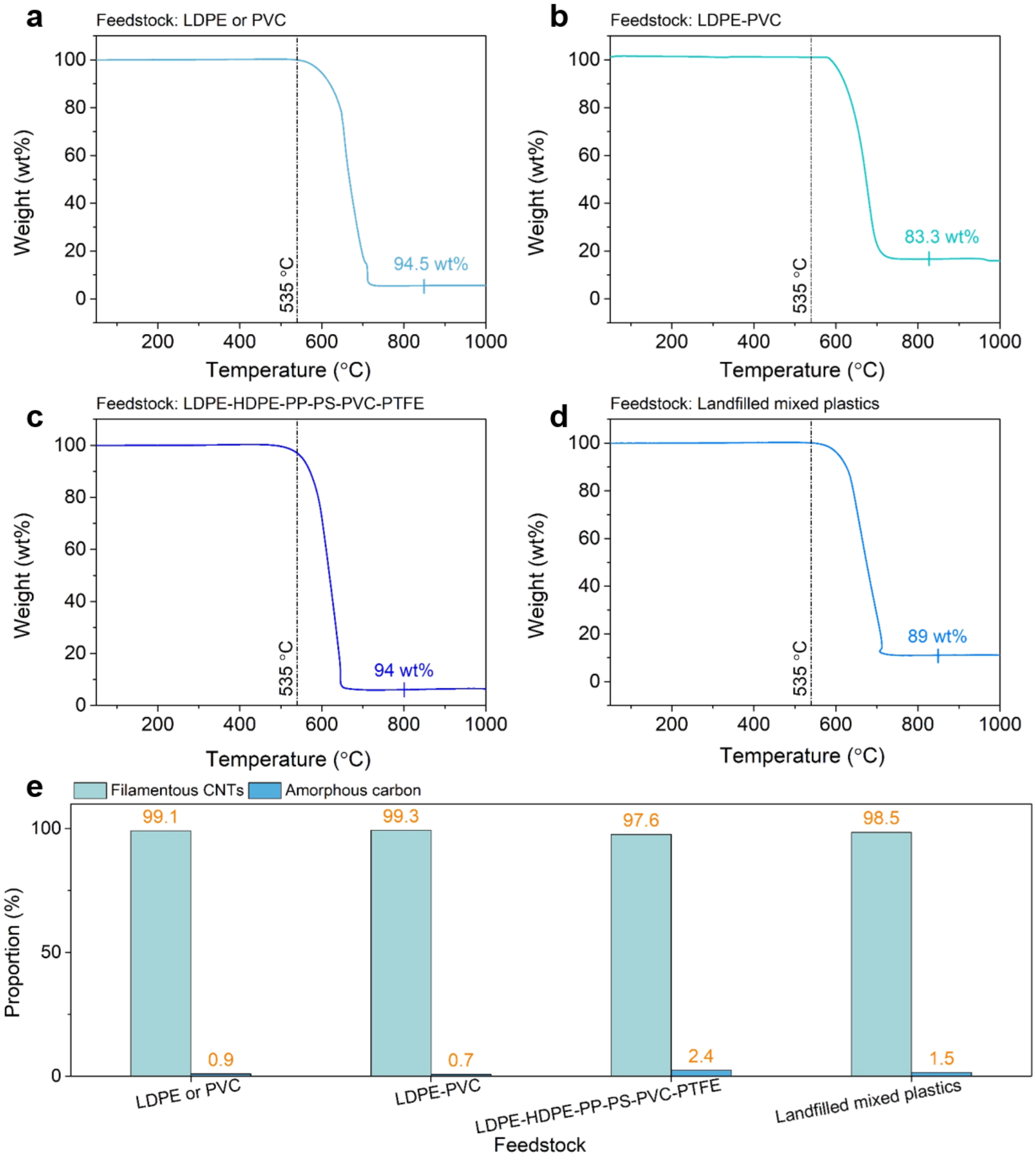

Fig. R14. TGA curves and the proportion of different types of carbon. (a) The weight ratio of carbon in the used catalyst after 35 successive catalytic cycles, using LDPE (Cycle 1-Cycle 25, Cycle 30-Cycle 35) or PVC (Cycle 26-Cycle 30) as the feedstock. Reaction condition: 3 g plastic and 3 g FeNi/Ni/CNTs catalyst in the bottom layer; 3 g FeNi/Ni/CNTs catalyst in the top layer. (b) The weight ratio of carbon in the used catalyst after 30 successive catalytic cycles, using the LDPE-PVC mixture (mass ratio of 9:1) as the feedstock. Reaction condition: 3 g plastic and 3 g FeNi/Ni/CNTs catalyst in the bottom layer; 3 g FeNi/Ni/CNTs catalyst in the top layer. (c) The weight ratio of carbon in the used catalyst after 5 successive catalytic cycles, using the LDPE-HDPE-PP-PS-PVC-PTFE mixture as the feedstock. Plastic mixtures of LDPE, HDPE, PP, PS, PVC and PTFE with mass ratios of 875:615:965:330:510:12. Reaction condition: 30 g plastic and 1.5 g

FeNi/Ni/CNTs catalyst in the bottom layer; 1.5 g FeNi/Ni/CNTs catalyst in the top layer. (d) The weight ratio of carbon in the used catalyst after 35 successive catalytic cycles, using the landfilled mixed plastics as the feedstock. Feedstock: PVC and landfilled plastic waste mixtures with a mass ratio of 1:9 (Cycle 1-Cycle 30), PTFE and landfilled plastic waste mixtures with a mass ratio of 1:9 (Cycle 31-Cycle 35). Reaction condition: 3 g plastic and 3 g FeNi/Ni/CNTs catalyst in the bottom layer; 3 g FeNi/Ni/CNTs catalyst in the top layer. (e) The proportion of filamentous CNTs and amorphous carbon in carbon materials obtained from the catalytic decomposition of different plastic feedstock.

Fig. R14 has been included as Supplementary Fig. 52b, Supplementary Fig. 48b, Supplementary Fig. 61b, and Supplementary Fig. 55b in the supplementary material.

2. It is demonstrated that Cl^- derived from PVC adsorbs on Fe to form $FeCl_3$, which subsequently decomposes to facilitate FeNi alloy formation. However, the manuscript does not discuss the role of F^-. Could the authors clarify whether F^- plays an analogous role in this regeneration mechanism?

Response: We appreciate the reviewer for raising this very insightful comment. To investigate whether F could function as a catalyst regenerant analogous to Cl, new comparative experiments were performed: 5 g FeC-FeNi/Ni/CNTs were homogenized with 5 g PVC or PTFE, then reacted under 600 W microwave irradiation for 10 min. Post-reaction XRD analysis of the catalyst reveals that PVC facilitates FeNi alloy formation, whereas F does not exert a comparable effect (**Fig. R15**).

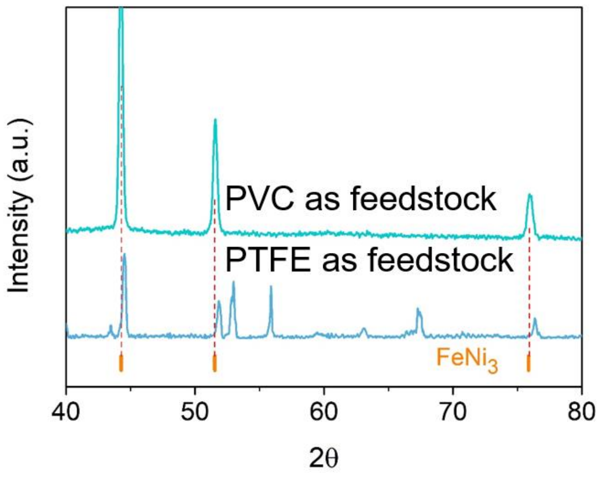

Fig. R15. The XRD pattern of the FeC-FeNi/Ni/CNTs catalyst after its reaction with PVC and PTFE.

Based on previous findings (Nano Letters 2002, 2, 9, 1009-1013), F species can efficiently cleave carbon nanotubes. Additionally, consistent with TEM observations, the surface structure of PTFE-treated CNTs (mass ratio of 6:1, 600 W for 10 min) progressively degraded with fracturing (**Fig. R16**). Consequently, F may re-expose encapsulated metal species by shearing CNTs, thereby enhancing catalytic activity and stability through catalyst activation (**Page 10**).

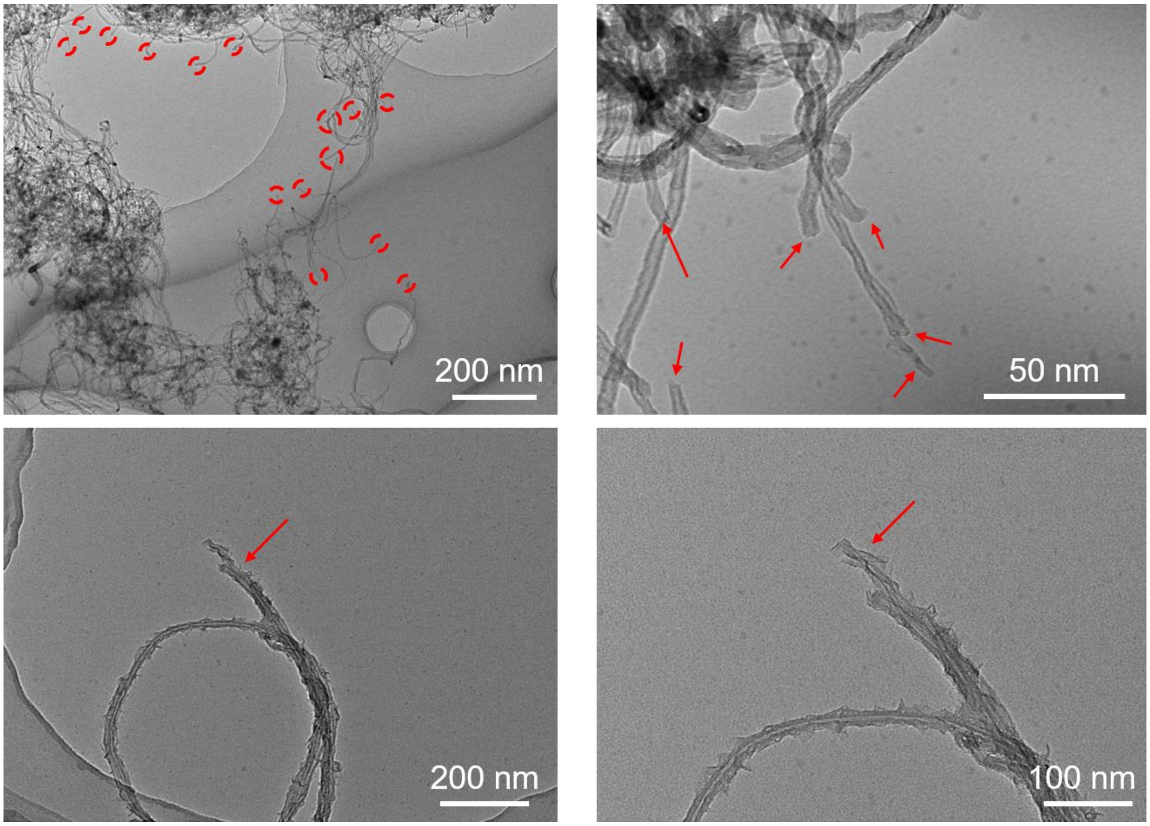

Fig. R16. The TEM images of the CNTs after the reaction with PTFE.

Figs. R15-16 have been included as Supplementary Figs. 37-38 in the supplementary material.

3. In addition, the relationship between the formation of the Ni–Fe alloy and the catalytic activity toward H_2 production and CNTs growth remains unclear. Further explanation on this point appears necessary.

Response: We sincerely thank the reviewer for the critical comments. FeNi alloy formation enhances waste plastic decomposition for hydrogen and carbon nanotube production,

attributed to the enhanced electron cloud density and the electron transfer effect between Fe and Ni. As shown in **Fig. R17a**, compared with the catalyst precursor, the K-edge of Fe in the regenerated catalyst is significantly shifted toward the position of the Fe foil, indicating an increase in the electron cloud density of Fe in the regenerated catalyst. Similarly, **Fig. R17b** shows that the K-edge energy of Ni in both the catalyst precursor and regenerated catalyst is lower than that of the Ni foil, suggesting electron transfer from Fe to Ni, which enhances the electron cloud density of Ni. Increased electron cloud density of Fe and Ni can improve the adsorption of the reaction substrate and the activation of C-H bonds, driving hydrocarbon conversion to H* and C_XH_Y*, and continuous C-H cleavage yields hydrogen and carbon tubes (J. Am. Chem. Soc., 147, 38141-38157 (2025). Applied Catalysis B: Environmental 327, 122451 (2023)). Compared to Ni, Fe exhibits superior carbon-adsorbing properties; enhanced Fe electron cloud density also likely strengthens its bonding with C atoms, promoting carbon diffusion and crystallization into long carbon nanotubes (Energy 262, 125415 (2023). Applied Catalysis B: Environmental 208, 44-59 (2017). Journal of Catalysis 222, 250-254 (2004)). The above discussions are also consistent with the DFT calculation and were added to **Page 11** in the revised manuscript (**Fig. R18**).

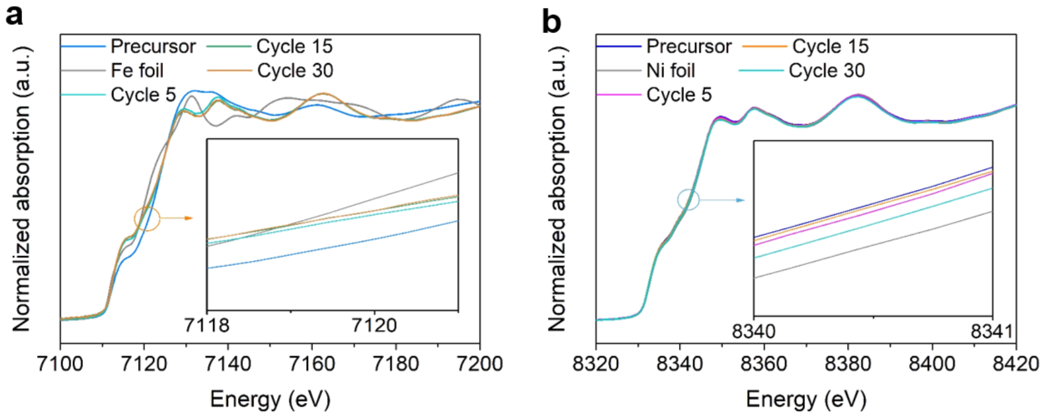

Fig. R17. XAS analysis. (a) Fe K-edge XANES spectra of FeC-FeNi/Ni/CNTs (precursor) and FeNi/Ni/CNTs catalysts as well as references. (b) Ni K-edge XANES spectra of FeC-FeNi/Ni/CNTs and FeNi/Ni/CNTs catalysts as well as references.

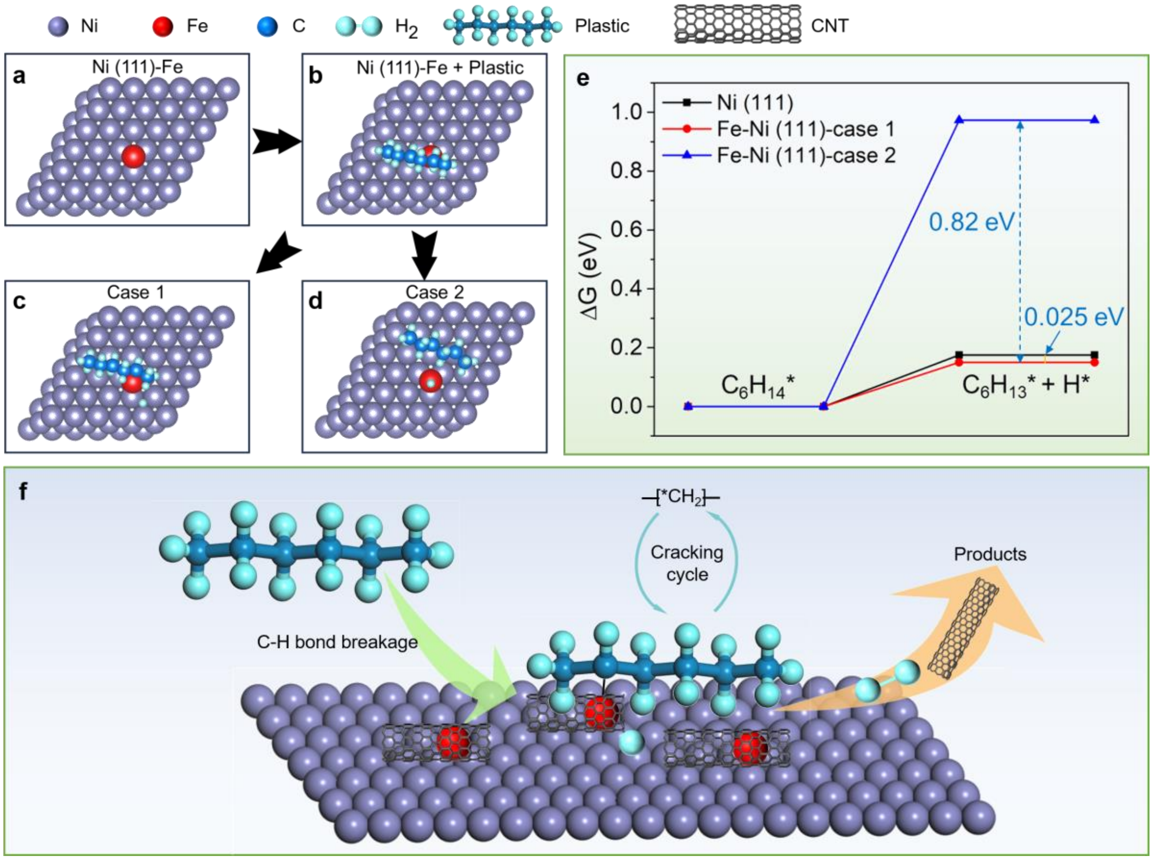

Fig. R18. Proposed mechanism. The model of (a) Ni-Fe, (b) Ni-Fe + plastic, (c) case1 and (d) case 2. (e) The calculated free-energy changes for C-H bond breaking reaction of C_6H_{14} on Ni (111), Fe-Ni (111)-case 1 and Fe-Ni (111)-case 2. (f) An illustration on microwave catalytic upcycling of plastic over FeNi/Ni/CNTs catalyst.

Figs. R17-18 have been marked as Fig. 3 and Fig. 5 in the manuscript.

4. PVC and PTFE typically constitute only a minor fraction in real-world plastic waste streams. The catalytic activity appears to decrease in the absence of PVC, could the authors comment on whether similar performance can be expected at process scale given the much lower PVC content compared to laboratory-scale conditions?

Response: We appreciate the reviewer for raising this important point. PVC, the third most produced plastic after PE and PP, accounts for 12.8% of the global annual production of plastic products. According to Plastics Europe's 2023 report, the global plastic recycling rate stood at less than 10% in 2023, indicating that over 90% of post-consumer plastics are discarded as waste (https://plasticseurope.org/es/knowledge-hub/plastics-the-fast-facts-2024/). In this work, plastic mixing ratios were formulated based on production ratios,

which is consistent with the previous works (Nature Sustainability 6, 1258-1267 (2023). Nature Chemical Engineering 1, 376-384 (2024)), and a series of mixed plastics with varying proportions were studied to account for the compositional complexity of mixed waste plastics, thereby demonstrating the technology's adaptability. Notably, relevant studies have also reported that PVC comprises approximately 5% of plastic waste (in Plastic Waste and Recycling (ed. Letcher, T. M.) Ch. 2, 13-32 (Academic Press, 2020). Nature Chemical Engineering 1, 376-384 (2024)).

To further investigate the catalytic performance of this strategy if PVC content is low, new microwave catalytic decomposition experiments were conducted using LDPE-PVC mixtures with reduced PVC levels. As shown in **Fig. R19**, the strategy maintains good catalytic performance even with PVC contents as low as 1% (LDPE: PVC=99:1) over 30 successive cycles. Notably, over 92.5 wt% of the mixture is converted into gas and solid carbon per cycle. H_2 efficiency ranges from 83.5% to 91.4% with a purity of 89.8-94.4 vol%, while carbon efficiency correspondingly ranges from 76.3% to 90.9%. Respective yields of H_2 and CNTs reach 945.2 mmol $g^{-1}_{catalyst}$ and 10,768 mg $g^{-1}_{catalyst}$. These results demonstrate that even with a minor PVC fraction, the strategy achieves relatively good catalytic decomposition of the plastic mixture into H_2 and carbon nanotubes.

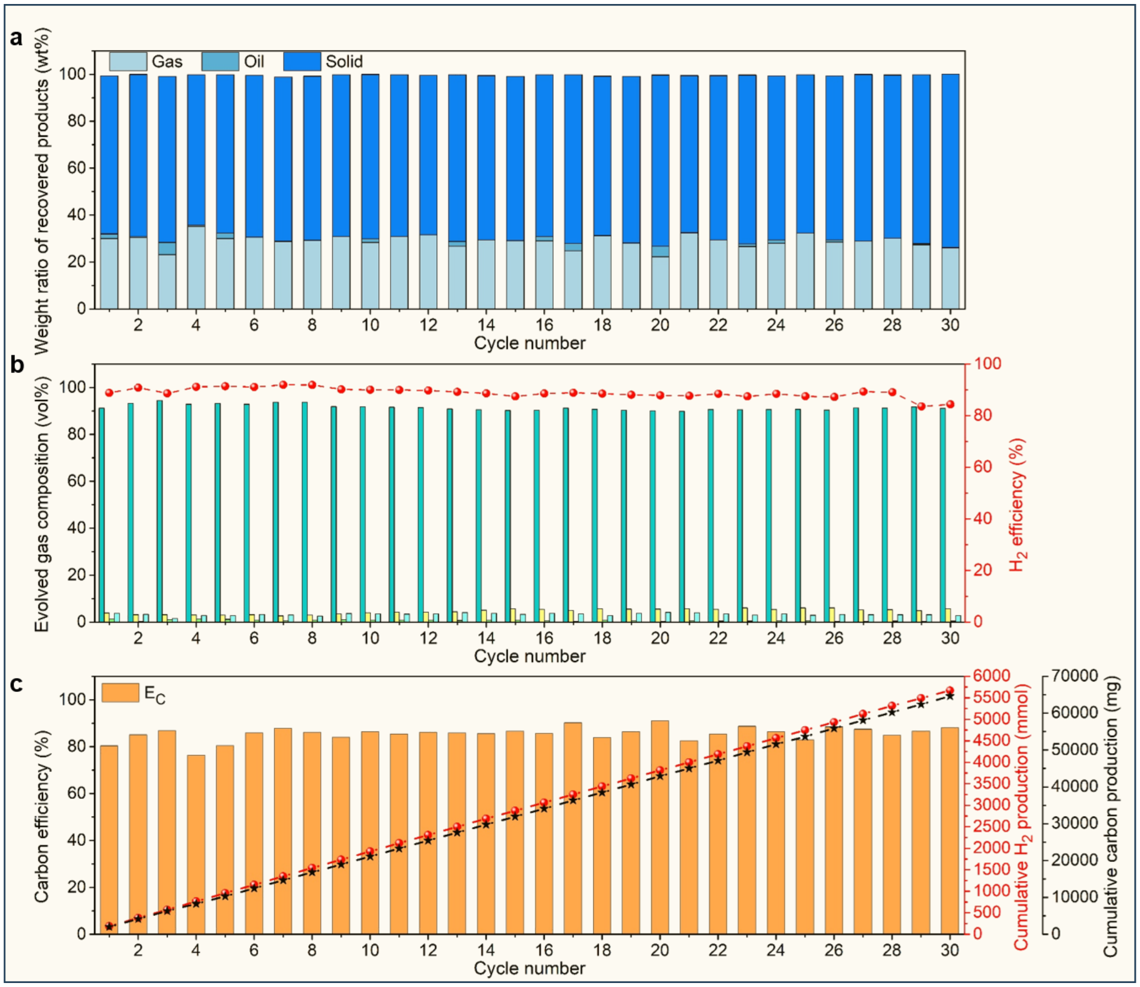

Fig. R19. Successive cycles of microwave catalytic decomposition of LDPE-PVC plastic mixture over FeNi/Ni/CNTs catalyst. (a) The weight ratio of recovered gas, oil and solid. (b) Evolved gas composition (vol%) and H_2 efficiency (%). (c) Carbon efficiency (%) (E_C), cumulative H_2 production (mmol) and cumulative carbon production (mg). Reaction conditions: 3 g plastic and 3 g FeNi/Ni/CNTs catalyst in the bottom layer; additional 3 g FeNi/Ni/CNTs catalyst in the top layer. Plastic: LDPE and PVC mixture (the mass ratio of LDPE to PVC is 99:1).

Fig. R19 has been marked as Supplementary Fig. 11 in the supplementary materials. The new findings with a very low PVC ratio were underlined on page 6 in the revised manuscript, further demonstrating the effectiveness and applicability of this strategy.

5. Based on the unit of the turnover number (molcarbon mol-1Ni), it appears that the authors defined the active site as Ni. However, it is unclear why Fe was not considered. As discussed in the section on microwave catalysis mechanisms, both Ni and Fe act as active

sites, and the catalytic activity varies depending on the Ni-to-Fe ratio in the catalyst. A related study published in Nature Catalysis, 2020, 3, 902–912, which investigated microwave-assisted conversion of plastic waste to H2 and CNTs using an Fe-based catalyst, also demonstrated that Fe plays a key role as an active site. The authors should clarify why Fe was excluded from the turnover number calculation in the present study.

Response: We sincerely thank the reviewer for pointing out this issue. We apologize for this oversight. Based on the reviewer's comments, the turnover number was re-calculated with respect to total molar amount of Fe and Ni, which is 107.6 mol_{carbon} mol^{-1}_{NiFe}. Consequently, nearly 100% of the plastic waste can be continuously upcycled into H_2 and CNTs. Accordingly, the related information was corrected (**Page 1 and Page 12**).

6. In the reusability tests, it appears that the spent catalyst and solid carbon mixture from the previous reaction were not removed, and fresh plastic was simply added in each cycle. In this case, wouldn't the unreacted plastic from the previous cycle also be carried over into the subsequent one? In this case, can the yield and selectivity for each cycle be considered reliable? Since the initial state of each cycle is not well-defined, the reproducibility and accuracy of the reported values are questionable. Furthermore, after the first cycle, the top layer in the tandem system is no longer a fresh catalyst layer, but rather a spent catalyst mixed with solid carbon mixture. This may significantly deviate from the initially designed tandem system. Given this change in the system structure, it is important that the authors clearly explain how such deviations were addressed.

Response: We appreciate the reviewer for raising this important point. As shown in **Fig. R20**, the reaction process completes at approximately 8 min, as product distribution, hydrogen efficiency, and cumulative evolved gas volume level off. To ensure the complete decomposition of the input plastic in this study, the reaction time was further extended to 15 minutes in each cycle.

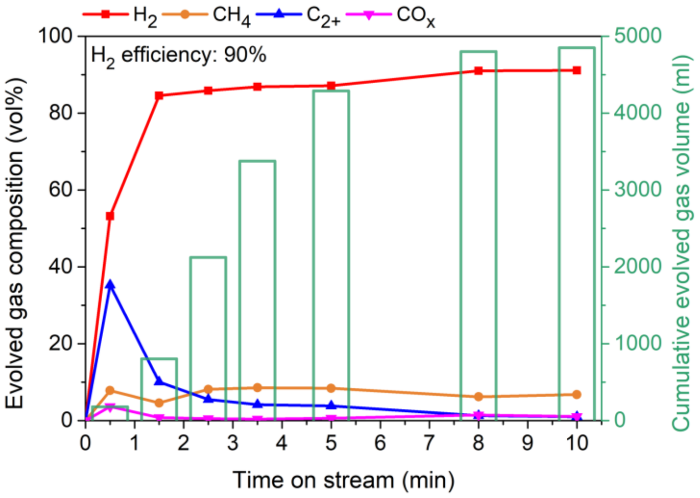

Fig. R20. A time-on-stream analysis of the microwave catalytic decomposition of plastic.

To verify the absence of unreacted plastic residues in the catalyst after each use, a new thermogravimetric analysis was performed on samples collected after different reaction cycles under a N_2 atmosphere. As illustrated in **Fig. R21**, the LDPE-PVC (9:1) mixture exhibits complete decomposition at approximately 500 °C, whereas the used catalyst samples after 15 and 30 cycles show negligible weight loss within this temperature range. This indicates minimal carryover of unreacted plastics into subsequent reaction cycles, supporting the sustained efficiency of the microwave-assisted catalytic plastic decomposition process.

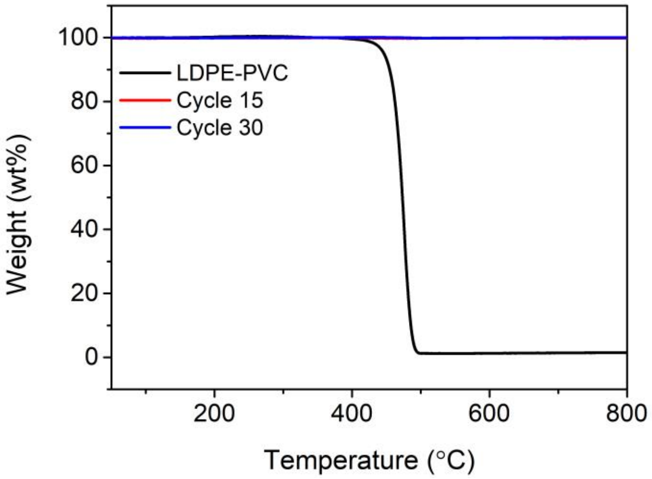

Fig. R21. Thermogravimetric analysis results of the LDPE-PVC plastic mixture and the used catalyst after 15 and 30 catalytic cycles.

The reproducibility and accuracy of the reported values can be supported by data from multiple successive 30 microwave catalytic plastic decomposition cycles (**Figs. R22-24**), which demonstrate good reproducibility and accuracy of the study.

While the catalyst is no longer in its initial fresh state after a few cycles, the carbon generated during the reaction does not alter the structure of metal species in the FeNi/Ni/CNTs catalyst (**Fig. R25**). It only serves to support and disperse these metal species (**Fig. R26**). Additionally, the reaction-derived carbon nanotubes are recognized as excellent microwave absorbers to improve the catalyst microwave absorption and they exhibit minimal direct activity in plastic decomposition, as the reported (Nature Catalysis 3, 902-912 (2020). The FeNi metal still remains the catalytic center across all cycles, so it does not substantially affect the designed tandem catalysis.

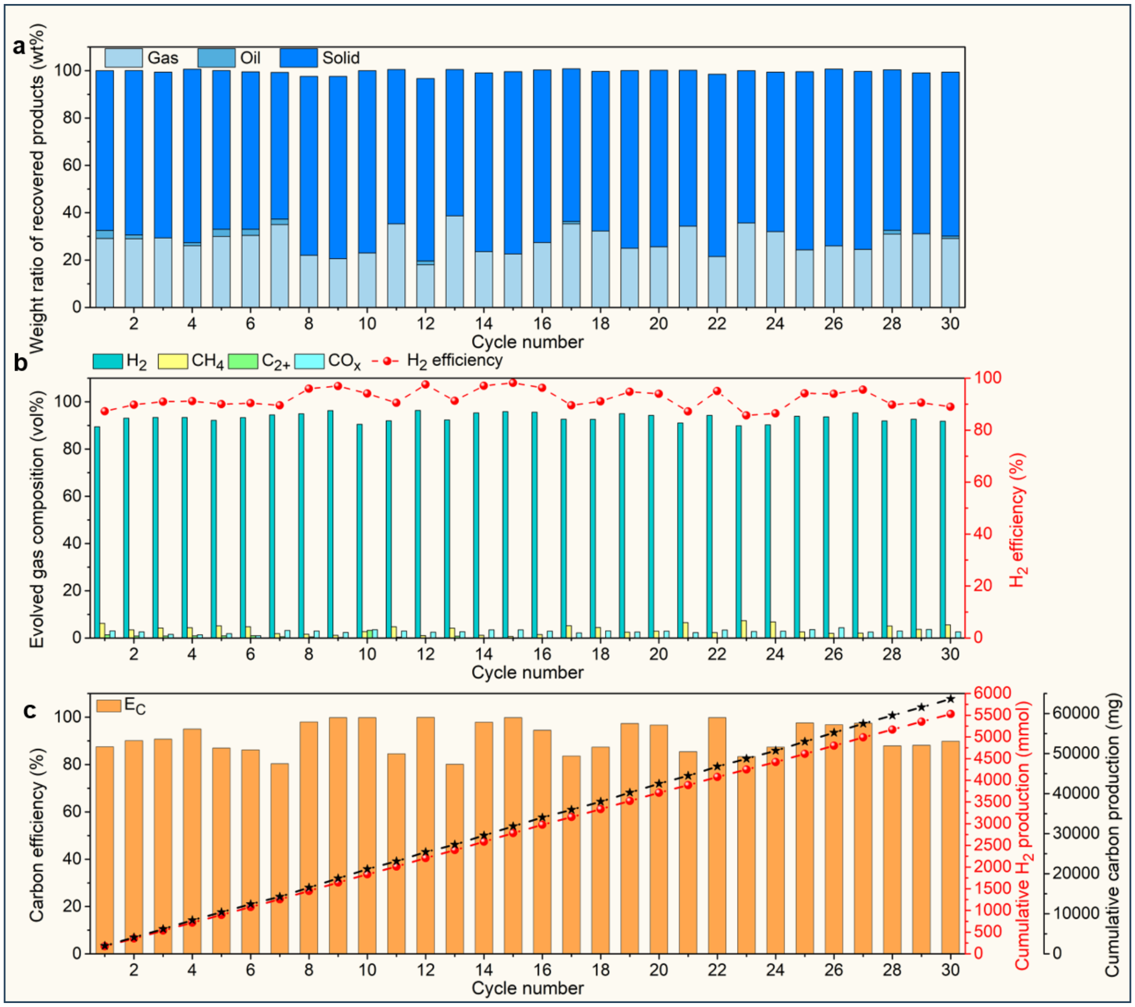

Fig. R22. Successive cycles of microwave catalytic decomposition of LDPE and PVC mixture **plastic over FeNi/Ni/CNTs catalyst**. (a) The weight ratio of recovered gas, oil and solid. (b) Evolved gas composition (vol%) and H_2 efficiency (%). (c) Carbon efficiency

(%) (E_C), cumulative H_2 production (mmol) and cumulative carbon production (mg). Reaction conditions: 3 g plastic and 3 g FeNi/Ni/CNTs catalyst in the bottom layer; additional 3 g FeNi/Ni/CNTs catalyst in the top layer. Plastic: LDPE and PVC mixture (the mass ratio of LDPE to PVC is 9:1).

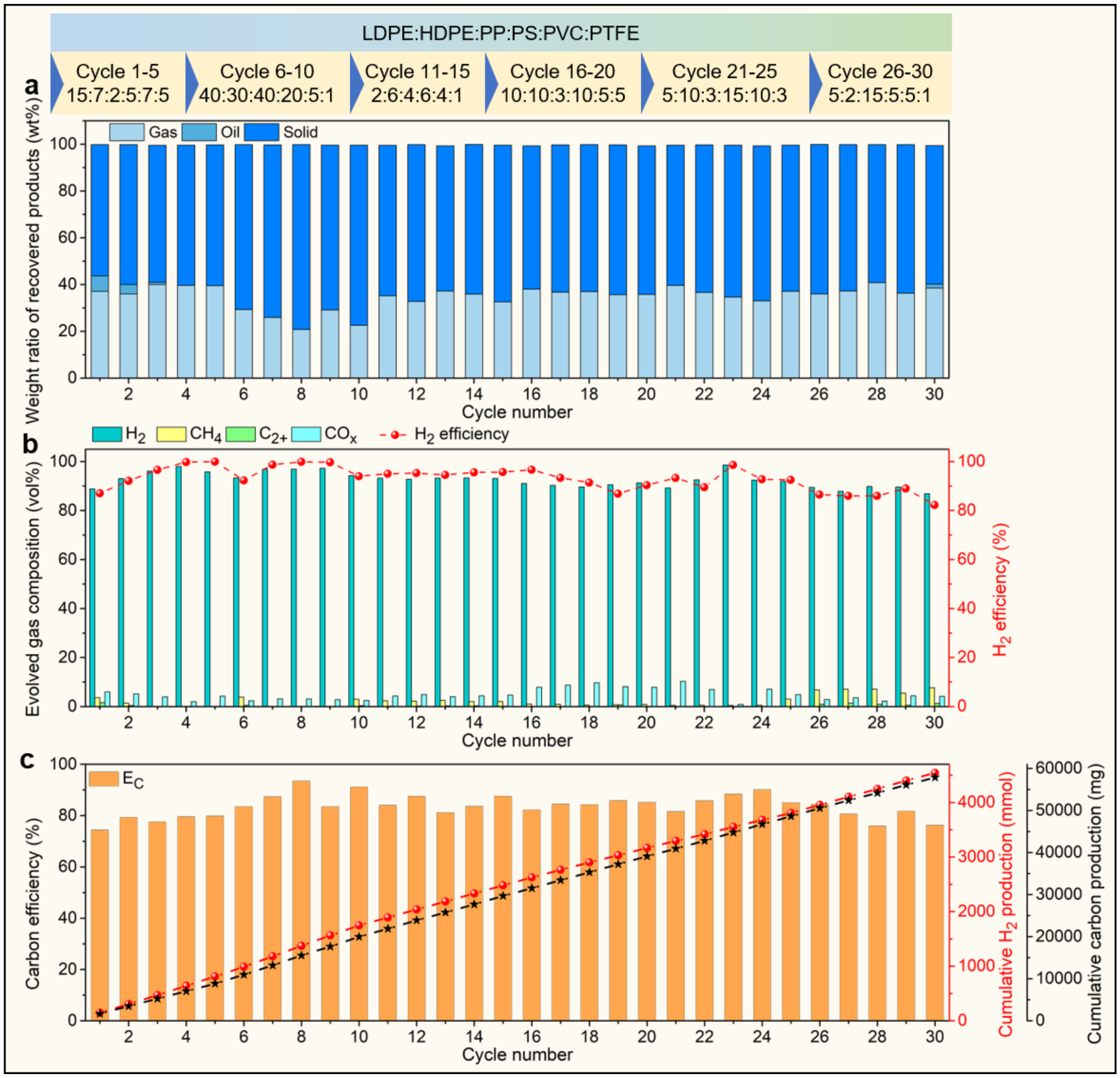

Fig. R23. Successive cycles of microwave catalytic decomposition of LDPE, HDPE, PP, PS, PVC and PTFE plastic mixtures with different ratios over FeNi/Ni/CNTs catalyst. (a) The weight ratio of recovered gas, oil and solid. (b) Corresponding evolved gas composition (vol%) and H_2 efficiency (%). (c) Carbon efficiency (%) (E_C), cumulative H_2 production (mmol) and cumulative carbon production (mg) (dotted lines). Reaction conditions: 3 g mixture of LDPE, HDPE, PP, PS, PVC and PTFE plastic and 3 g FeNi/Ni/CNTs catalyst in the bottom layer; 3 g FeNi/Ni/CNTs catalyst in the top layer.

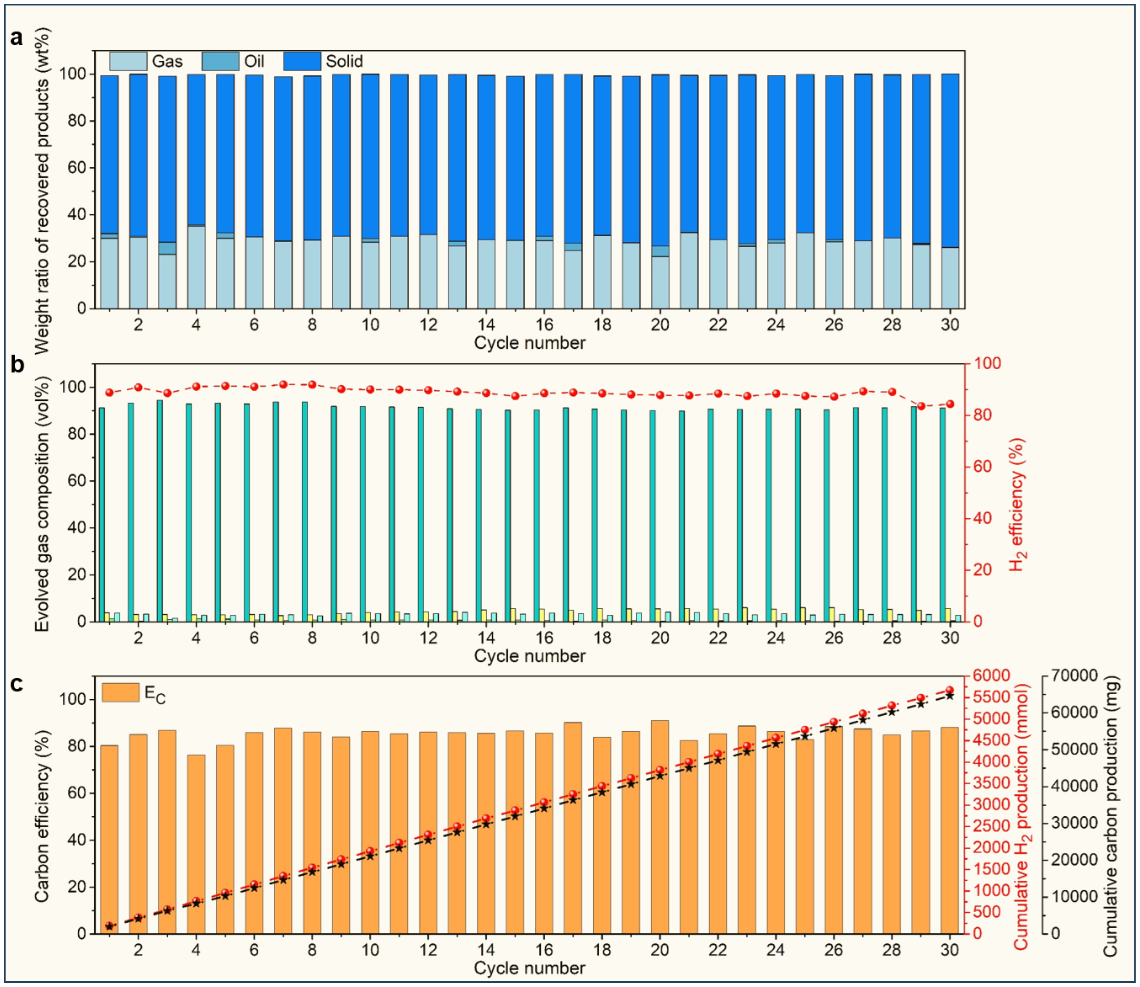

Fig. R24. Successive cycles of microwave catalytic decomposition of LDPE-PVC plastic mixture over FeNi/Ni/CNTs catalyst. (a) The weight ratio of recovered gas, oil and solid. (b) Evolved gas composition (vol%) and H_2 efficiency (%). (c) Carbon efficiency (%) (E_C), cumulative H_2 production (mmol) and cumulative carbon production (mg). Reaction conditions: 3 g plastic and 3 g FeNi/Ni/CNTs catalyst in the bottom layer; additional 3 g FeNi/Ni/CNTs catalyst in the top layer. Plastic: LDPE and PVC mixture (the mass ratio of LDPE to PVC is 99:1).

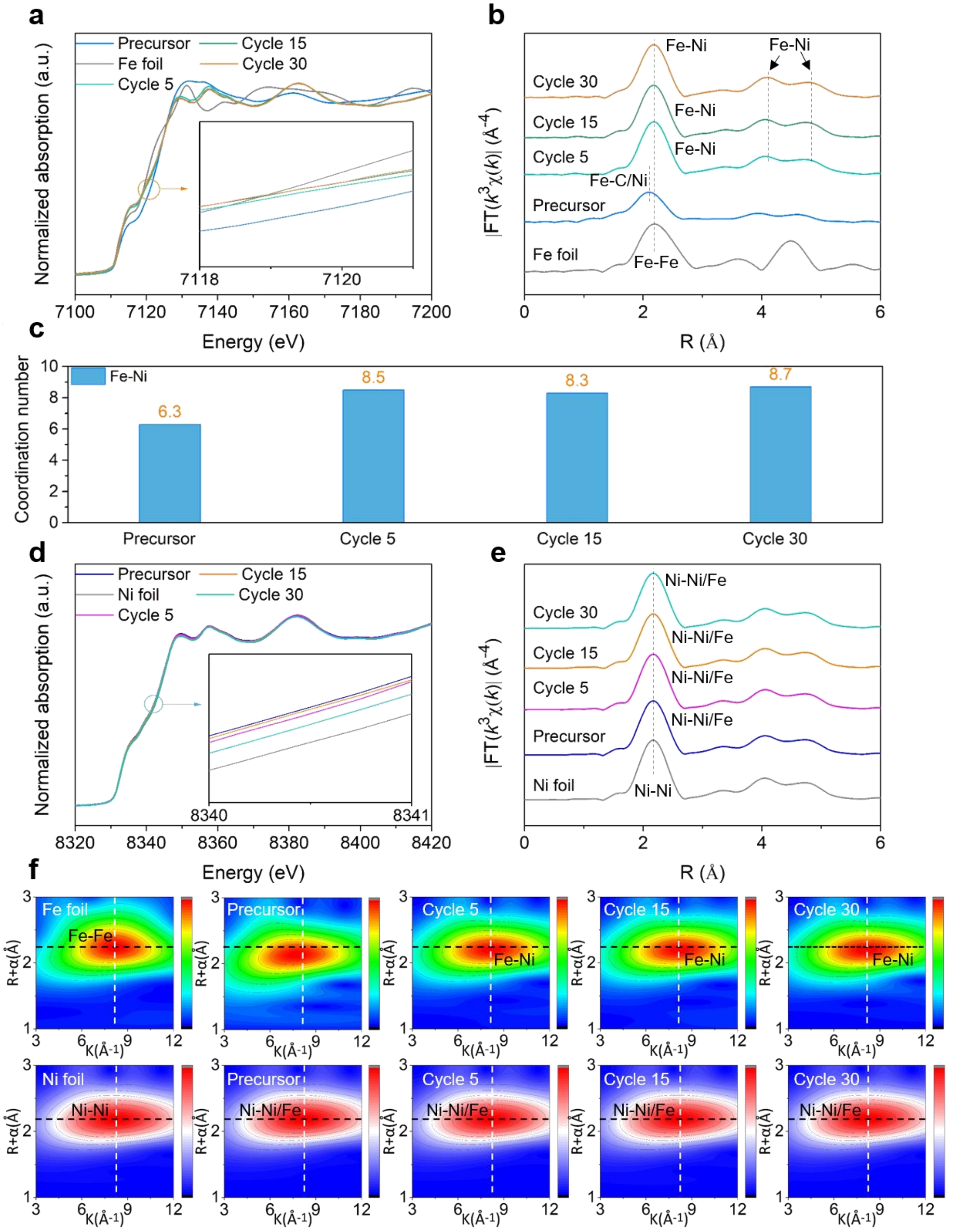

Fig. R25. XAS analysis. (a) Fe K-edge XANES spectra of FeC-FeNi/Ni/CNTs (precursor) and FeNi/Ni/CNTs (regenerated) catalysts as well as references. (b) Fourier transforms (FT) of Fe EXAFS spectra of FeC-FeNi/Ni/CNTs and FeNi/Ni/CNTs catalysts and references. (c) Changes of coordination number in FeC-FeNi/Ni/CNTs and FeNi/Ni/CNTs catalysts. (d) Ni K-edge XANES spectra of FeC-FeNi/Ni/CNTs and FeNi/Ni/CNTs catalysts as well

as references. (e) Fourier transforms (FT) of EXAFS spectra (Ni) of FeC-FeNi/Ni/CNTs and FeNi/Ni/CNTs catalysts and references. (f) WT-EXAFS of Fe and Ni.

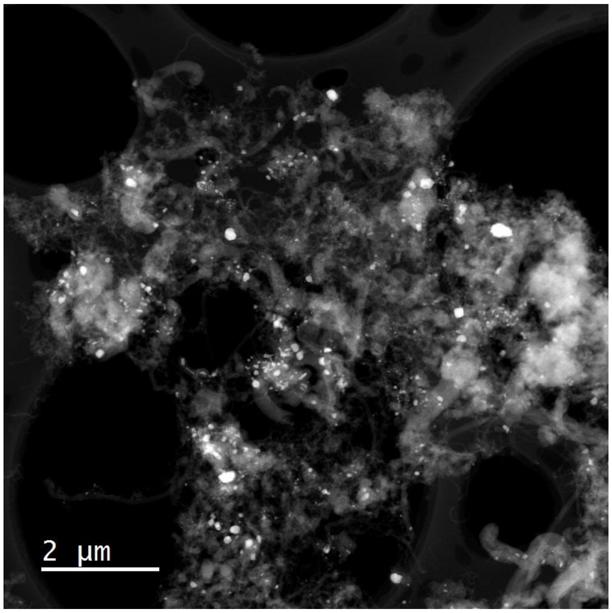

Fig. R26. TEM image of the FeNi/Ni/CNTs catalyst after the reactions.

Fig. R20, Fig. R22, Fig. R23, Fig. R24 and Fig. R25 have been labelled as Supplementary Fig. 2b, Fig. 1, Fig. 4, Supplementary Fig. 11 and Fig. 3 in the manuscript and supplementary material.

7. In the section Mechanism of catalyst regeneration by Cl^-, the FeC–FeNi/Ni@CNT (precursor) catalyst is introduced. However, it is unclear how this catalyst was prepared. It would be helpful if the authors could provide a description of the synthesis method used for this catalyst.

Response: We appreciate the reviewer for raising this point. The synthesis method for the FeC-FeNi/Ni/CNT precursor is detailed below:

First, the monometallic Ni/CNTs catalyst was prepared by the following procedure: a mixture of $Ni(CH_3COO)_2{\cdot}4H_2O$ and SiC (12 mesh) with a mass ratio of 3:1 (e.g., 30 g: 10 g) was placed in a quartz reaction flask, then treated at 400 W of microwave irradiation power under N_2 atmosphere for 20 min. After reaction, the mixture was then sieved through a 100-mesh sieve to obtain the Ni/CNTs catalyst as the SiC with larger size left upon the sieve. Therefore, the Ni/CNTs catalyst can be separated directly from the SiC. The SiC was collected and reused.

Second, the catalyst precursor FeC-FeNi/Ni/CNTs was prepared by the following procedure: in a typical preparation process, 10 g of the above synthesized Ni/CNTs catalyst and 6.35 g iron acetylacetonate ($Fe(C_5H_7O_2)_3$) were mixed and placed in a quartz reaction flask, then treated at 400 W of microwave irradiation power under N_2 atmosphere for 10 min to acquire the FeC-FeNi/Ni/CNTs catalyst precursor.

This was added to the experimental section on page 13 in the revised manuscript.

8. There appears to be a lack of consistency in the data related to the regenerated FeNi/Ni@CNT catalyst. In Fig. 2g–f, the regenerated catalyst corresponds to that obtained after 15 cycles, whereas in Fig. 3, the regenerated catalyst is from after only 5 cycles. Using regenerated catalysts from different cycles across analyses may significantly hinder the overall interpretation of the results. It is recommended that the authors ensure consistency in the characterization of regenerated catalysts throughout the manuscript.

Response: We appreciate the reviewer for raising this valuable suggestion. Initially, sufficient synchrotron radiation characterization time was not obtained to adequately characterize the catalyst structure, leading to desynchronization issues in the characterization data presented in Fig. 2 and Fig. 3. In this revision, to maintain consistency and offer more systematically structured information about the catalyst, additional new XAS characterizations (e.g., Cycle 15 and Cycle 30) were conducted.

As shown in **Fig. R27** and **Table R2**, the coordination numbers of Fe-Ni in cycle 5 and cycle 15 are largely comparable, indicating consistent catalyst structures across these two cycles.

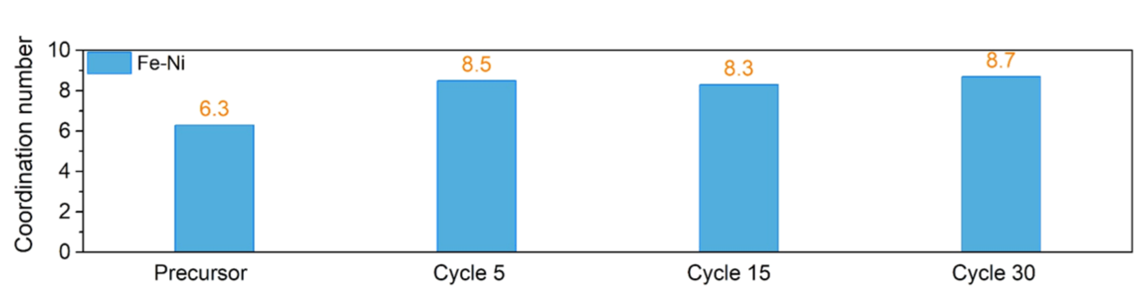

Fig. R27. XAS analysis. Changes of coordination number in FeC-FeNi/Ni/CNTs and FeNi/Ni/CNTs catalysts.

Table R2. Fitting results of the precursor and regenerated catalyst.

**Names**	**Shell**	**CN**	**R (Å)**	**σ ^2 (10^{-2} Å^2)**	**ΔE_0 (eV)**	**r-factor**
Precursor (Fe)	Fe-C	3.1	2.01	1.5(±2.3)	-4.8(±2)	0.013
	Fe-Ni	6.3	2.51	0.76(±0.17)		
Precursor (Ni)	Ni-Fe	1.3	2.41	0.037(±0.23)	6.9(±0.86)	0.0013
	Ni-Ni	6.5	2.5	0.4(±0.33)		
Cycle 5 (Fe)	Fe-Ni	8.5	2.49	0.6(±0.037)	7.8(±0.44)	0.002
Cycle 5 (Ni)	Ni-Fe	1.7	2.42	0.099(±0.24)	6.89(±0.85)	0.0013
	Ni-Ni	6.3	2.51	0.43(±0.35)		
Cycle 15 (Fe)	Fe-Ni	8.25	2.49	0.6(±0.04)	6.98(±0.57)	0.003
Cycle 15 (Ni)	Ni-Fe	1.6	2.42	0.082(±0.29)	6.0(±0.045)	0.0017
	Ni-Ni	6.4	2.51	0.4(±0.4)		
Cycle 30 (Fe)	Fe-Ni	8.7	2.49	0.65(±0.045)	8.1(±0.54)	0.003
Cycle 30 (Ni)	Ni-Fe	1.5	2.42	0.093(±0.29)	5.96(±0.95)	0.001
	Ni-Ni	6.5	2.51	0.4(±0.37)		

CN: coordination number; R: distance between absorber and backscatter atoms; σ^2: Debye-Waller factor to account for both thermal and structural disorders; ΔE_0: Inner potential correction; r factor (%) indicates the goodness of the fit. Fitting conditions: k around 3.0-12.0. R around 1.0-3.0. Rbkg=1. kw=1/2/3

Fig. R27 and Table R2 have been labelled as Fig. 3c and Supplementary Table 8 in the manuscript and supplementary material.

9. Please provide additional EDS image related to Fig. 2f to confirm the uniform distribution of carbon throughout the FeC–FeNi/Ni@CNTs catalyst.

Response: We appreciate the reviewer for pointing out this point. An additional EDS image corresponding to Fig. 2f was taken and is presented below. The EDS image indicates that

the carbon is distributed relatively uniformly within the FeC-FeNi/Ni/CNTs catalyst (**Figs. R28)**.

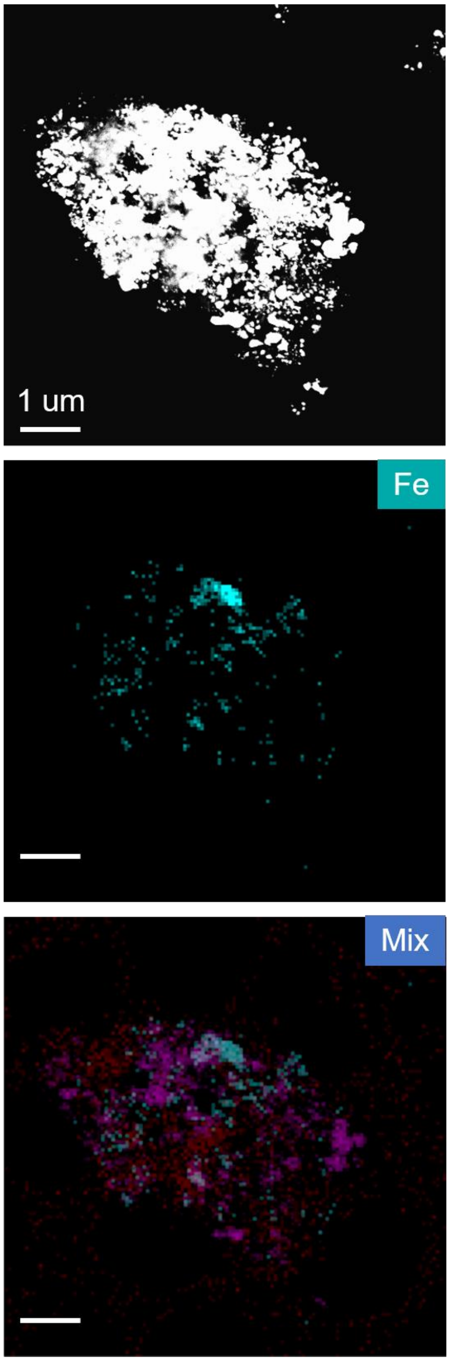

Fig. R28. EDX mapping of FeC-FeNi/Ni/CNTs.

Fig. R28 has been included as Supplementary Fig. 24 in the supplementary material.

10. In the EXAFS analysis, it seems necessary to more clearly distinguish between Fe–Ni /Fe–C and Ni–Ni/Ni–Fe bonds. To achieve this, it would be beneficial to include WT-EXAFS data in addition to the FT-EXAFS results, as it may provide better resolution and allow for more precise identification of the coordination environments.

Response: We appreciate the reviewer for raising this valuable suggestion. The WT-EXAFS data have been analyzed. The Fe K-edge XAS spectrum of the precursor FeC-FeNi/Ni/CNTs exhibits a significant shift to higher energy compared to the Fe foil, indicating Fe in a higher oxidation state (Nature Communications 12, 5589 (2021). Nature Communications 15, 1720 (2024)). Importantly, the Fe K-edge of the regenerated catalyst (Cycle 5, Cycle 15 and Cycle 30 with a feedstock ratio of LDPE to PVC of 9:1) shifts to a lower energy, approaching that of Fe foil. This suggests a reduction of Fe in the precursor to a state close to Fe foil (**Fig. 29a**) (Nano Research 13, 328-334 (2020)). This is because that in-situ reconstruction occurs under the action of the Cl^- released by PVC during the microwave catalytic decomposition of the LDPE-PVC mixture process. This process leads to the formation of FeNi alloy compounds (Nature Communications 15, 7475 (2024)), facilitating the change of the precursor to a highly efficient FeNi/Ni/CNTs catalyst. Extended X-ray absorption fine structure (EXAFS) analysis reveals dominant Fe-Ni coordination at 2.2 Å in FeNi/Ni/CNTs versus Fe-C/Ni bonding at around 2.1 Å in the precursor FeC-FeNi/Ni/CNTs. Enhanced Ni-Fe scattering singles at ~4.0 Å and ~4.7 Å in FeNi/Ni/CNTs further validate structural evolution (**Fig. 29b**).

The coordination numbers of Fe-Ni in FeC-FeNi/Ni/CNTs and FeNi/Ni/CNTs also support the above findings. In FeC-FeNi/Ni/CNTs, the Fe-Ni coordination number is 6.3. In the regenerated FeNi/Ni/CNTs, it increases to 8.5 (Cycle 5), 8.3 (Cycle 15) and 8.7 (Cycle 30), respectively (**Fig. 29c**). Ni pre-edge of the X-ray absorption near-edge structure (XANE) region shows metallic nature in both FeC-FeNi/Ni/CNTs and FeNi/Ni/CNTs, with energies slightly below Ni foil. This indicates there is small electron transfer between Ni and Fe (**Fig. 29d**) (Nature Communications 15, 7475 (2024). Advanced Materials 32, 2005433 (2020)). The Ni-Fe coordination number also increases from 1.3 in FeC-FeNi/Ni/CNTs to 1.7 (Cycle 5), 1.6 (Cycle 15) and 1.5 (Cycle 30) in FeNi/Ni/CNTs. The Ni coordination number has minimal change due to the higher Ni content compared to Fe. Furthermore, a peak at approximately 2.2 Å corresponds to Ni-Ni/Fe coordination (**Fig. 29e**).

Wavelet transform (WT) was employed to further analyze Fe and Ni K-edge EXAFS oscillations. The WT contour plot of Fe foil shows a single intensity maximum at ~8.1 $Å^{-1}$ (**Fig. 29f**), corresponding to Fe-Fe coordination. For the FeC-FeNi/Ni/CNTs precursor, the WT maximum appears at 7.8 $Å^{-1}$. In contrast, the WT maxima of regenerated FeNi/Ni/CNTs (Cycles 5, 15, and 30) shift to ~8.2 $Å^{-1}$, consistent with Ni-Fe coordination. The above results indicate that iron does not form good coordination with nickel in the precursor but in the regenerated catalyst. For Ni K-edge EXAFS oscillations, the WT maxima of Ni foil, FeC-FeNi/Ni/CNTs precursor, and regenerated FeNi/Ni/CNTs (Cycles

5, 15, and 30) all appear at ~8.3 Å^{-1}, attributed to Ni-Ni or Ni-Ni/Fe coordination. This is due to the higher Ni content relative to Fe and the metallic nature of Ni. Collectively, the WT results are consistent with the aforementioned results (**Fig. 29a-e and Table R3**).

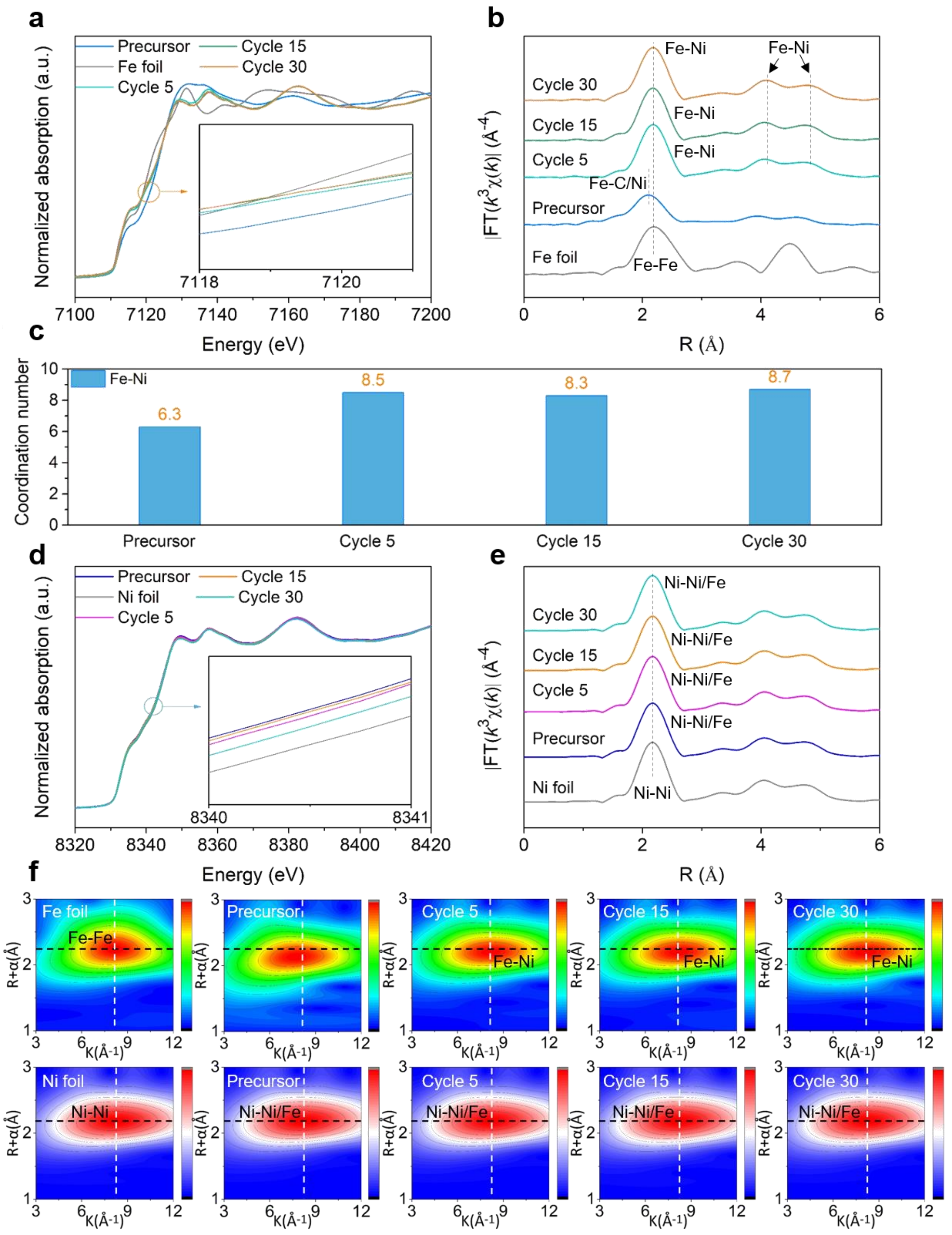

Fig. R29. XAS analysis. (a) Fe K-edge XANES spectra of FeC-FeNi/Ni/CNTs (precursor) and FeNi/Ni/CNTs (regenerated) catalysts as well as references. (b) Fourier transforms (FT)

of Fe EXAFS spectra of FeC-FeNi/Ni/CNTs and FeNi/Ni/CNTs catalysts and references. (c) Changes of coordination number in FeC-FeNi/Ni/CNTs and FeNi/Ni/CNTs catalysts. (d) Ni K-edge XANES spectra of FeC-FeNi/Ni/CNTs and FeNi/Ni/CNTs catalysts as well as references. (e) Fourier transforms (FT) of EXAFS spectra (Ni) of FeC-FeNi/Ni/CNTs and FeNi/Ni/CNTs catalysts and references. (f) WT-EXAFS of Fe and Ni.

Table R3. Fitting results of the precursor and regenerated catalyst.

**Names**	**Shell**	**CN**	**R (Å)**	**σ ^2 (10^{-2} $Å^2$)**	**ΔE_0 (eV)**	**r-factor**
Precursor (Fe)	Fe-C	3.1	2.01	1.5(±2.3)	-4.8(±2)	0.013
	Fe-Ni	6.3	2.51	0.76(±0.17)		
Precursor (Ni)	Ni-Fe	1.3	2.41	0.037(±0.23)	6.9(±0.86)	0.0013
	Ni-Ni	6.5	2.5	0.4(±0.33)		
Cycle 5 (Fe)	Fe-Ni	8.5	2.49	0.6(±0.037)	7.8(±0.44)	0.002
Cycle 5 (Ni)	Ni-Fe	1.7	2.42	0.099(±0.24)	6.89(±0.85)	0.0013
	Ni-Ni	6.3	2.51	0.43(±0.35)		
Cycle 15 (Fe)	Fe-Ni	8.25	2.49	0.6(±0.04)	6.98(±0.57)	0.003
Cycle 15 (Ni)	Ni-Fe	1.6	2.42	0.082(±0.29)	6.0(±0.045)	0.0017
	Ni-Ni	6.4	2.51	0.4(±0.4)		
Cycle 30 (Fe)	Fe-Ni	8.7	2.49	0.65(±0.045)	8.1(±0.54)	0.003
Cycle 30 (Ni)	Ni-Fe	1.5	2.42	0.093(±0.29)	5.96(±0.95)	0.001
	Ni-Ni	6.5	2.51	0.4(±0.37)		

CN: coordination number; R: distance between absorber and backscatter atoms; σ^2: Debye-Waller factor to account for both thermal and structural disorders; ΔE_0: Inner potential correction; r factor (%) indicates the goodness of the fit. Fitting conditions: k around 3.0-12.0. R around 1.0-3.0. Rbkg=1. kw=1/2/3

Fig. R29 and Table R3 have been labelled as Fig. 3 and Supplementary Table 8 in the manuscript and supplementary material.

11. In Supplementary Fig. 23, the XRD data presented to demonstrate the formation of FeCl3 is not fully convincing for the main FeC–FeNi/Ni@CNT sample, as the broad CNT-related XRD peak spanning 15–35° obscures the $FeCl_3$ peaks. Therefore, it is difficult to conclude that $FeCl_3$ formation is dominant compared to $NiCl_2$. This raises concerns about the adequacy of the presented analysis in supporting the claimed catalyst regeneration mechanism. Moreover, the core claim of this study—that Cl^- generated from PVC decomposition facilitates catalyst regeneration—appears to be insufficiently substantiated. While catalyst characterization before and after reaction is provided, there is a lack of real-time analysis linking Cl^- formation with structural changes in the catalyst. This gap limits the mechanistic understanding of the regeneration process.

Response: We honestly thank the reviewer for proposing this extremely key point and fully agree that it is the most convincing to observe the real time change of the catalyst under microwave irradiation, while it is impossible to do this due to the limit of the current technology available as no analytic tools can maintain sensitive and reliable measurement under microwave irradiation.

1) To verify whether PVC decomposition can promote the formation of iron chloride and nickel chloride, the following new indirect observation was conducted: 5 g of FeC-FeNi/Ni/CNTs and 5 g of PVC were mixed uniformly and reacted under 600 W microwave power for 10 minutes. Subsequently, 30 mL of deionized water was added and stirred at 25 °C for 2 hours. Afterwards, the above mixture was filtered, and the filtrate was dried completely in an oven at 100 °C, ultimately obtaining yellow crystals (**Fig. R30a**). XRD results of the yellow crystals indicate that they are composed of FeClx and $NiCl_2$, indicating that PVC decomposition can promote the formation of FeClx and $NiCl_2$ (**Fig. R30b**).

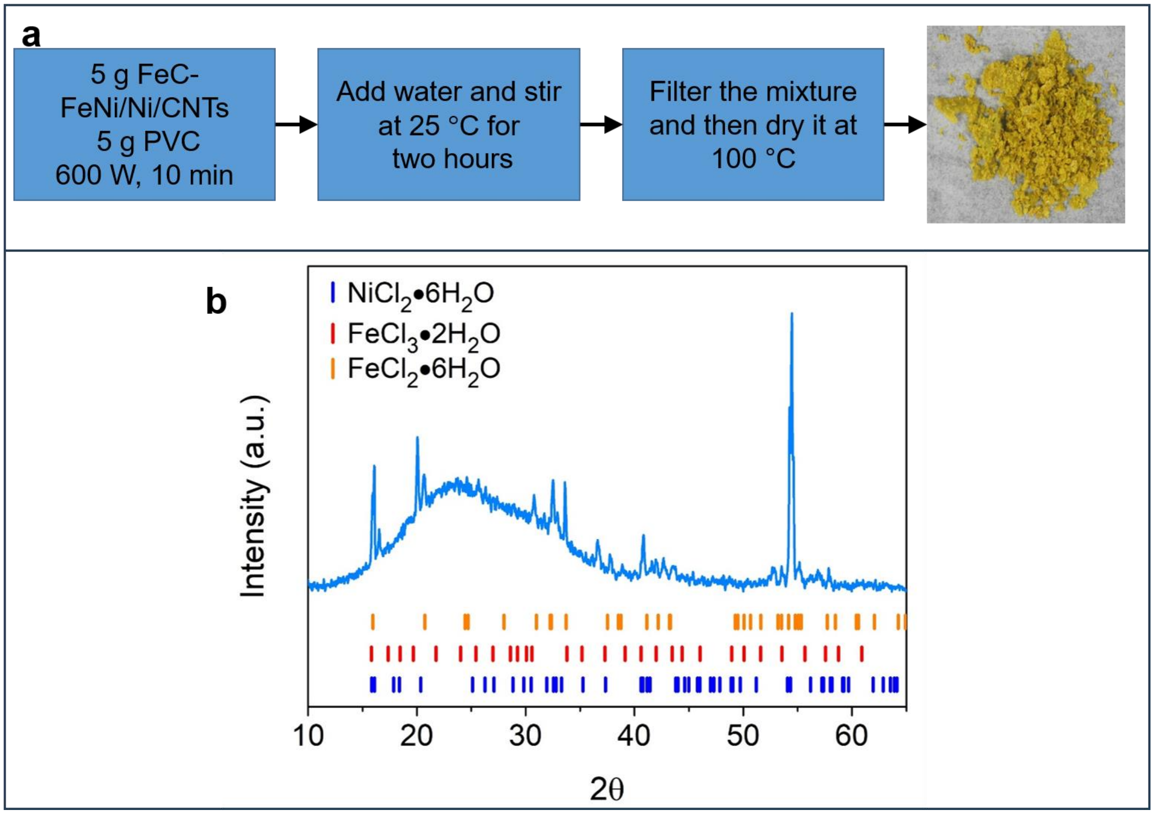

Fig. R30. (a) Schematic diagram of FeC-FeNi/Ni/CNTs and PVC reaction and post-treatment. (b) XRD results of the yellow crystals.

2) Furthermore, the reconstruction of FeNi alloy in the PVC-assisted FeC-FeNi/Ni/CNTs catalyst follows the procedure: hydrogen generated during plastic decomposition reduces the catalyst to produce water molecules, which combine with hydrogen chloride from PVC decomposition to form hydrochloric acid molecules. These hydrochloric acid molecules thereby effectively convert Ni and Fe into nickel chloride and iron chloride. Subsequently, the formed nickel chloride and iron chloride are reduced by hydrogen, thereby facilitating the formation of FeNi alloy.

This discussion is supported by the following new studies (**Figs. 31-35**). **Fig. R31** presents the H_2-TPR-MS results of the FeC-FeNi/Ni/CNTs catalyst precursor, and the mass spectrometry results show that water molecules are generated under the reduction of hydrogen, providing evidence for hydrogen acting as a reducing medium in the reaction.

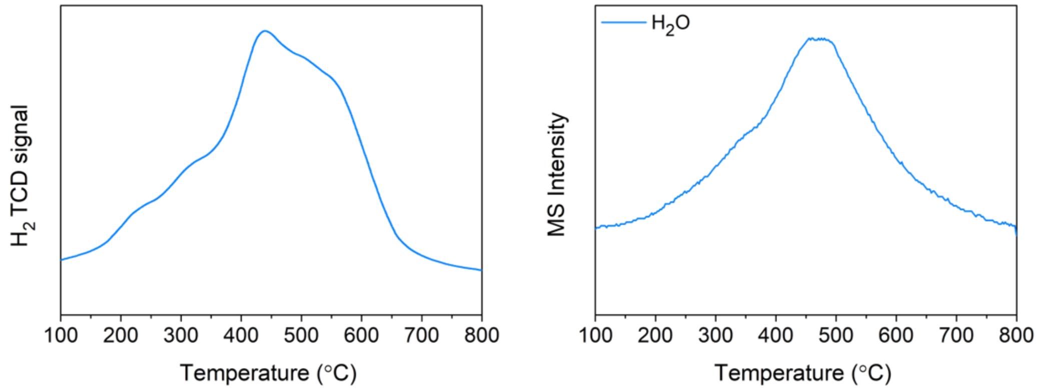

Fig. R31. The H_2-TPR-MS results of the FeC-FeNi/Ni/CNTs catalyst precursor.

3) To confirm that $NiCl_2$ and FeClx can form FeNi alloy under hydrogen, 0.3 g of yellow crystals and 0.1 g of commercial multi-walled carbon nanotubes were mixed uniformly and heated under 600 W microwave power and 10% H_2 atmosphere (flow rate 300 ml/min) for 10 minutes. It is found that FeNi alloy is formed (**Fig. R32**). Meanwhile, H_2-TPR-MS during the treatment of the obtained yellow crystals reveals the production of HCl, which is consistent with the aforementioned process of $NiCl_2$ and FeClx removing chlorine atoms under hydrogen (**Fig. R33**). These results support the above discussion regarding the PVC-assisted reconstruction of the catalyst to form FeNi alloy.

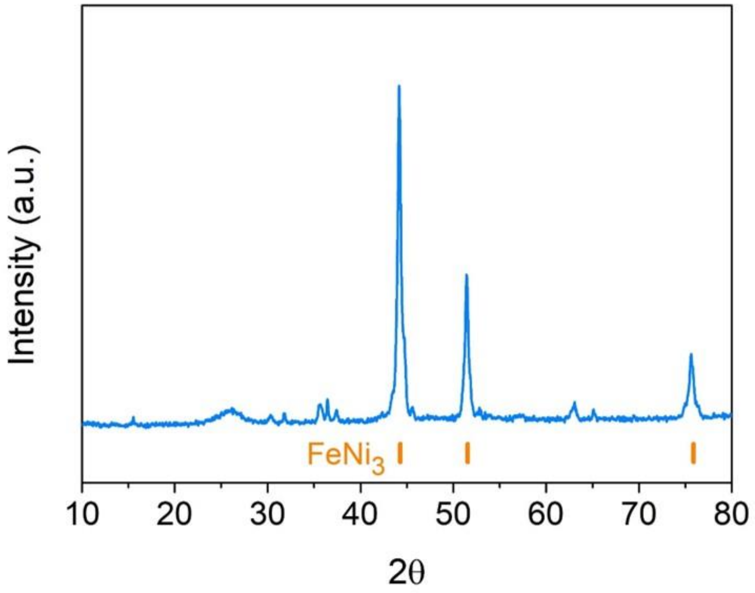

Fig. R32. XRD results of the yellow crystals after being treated with H_2.

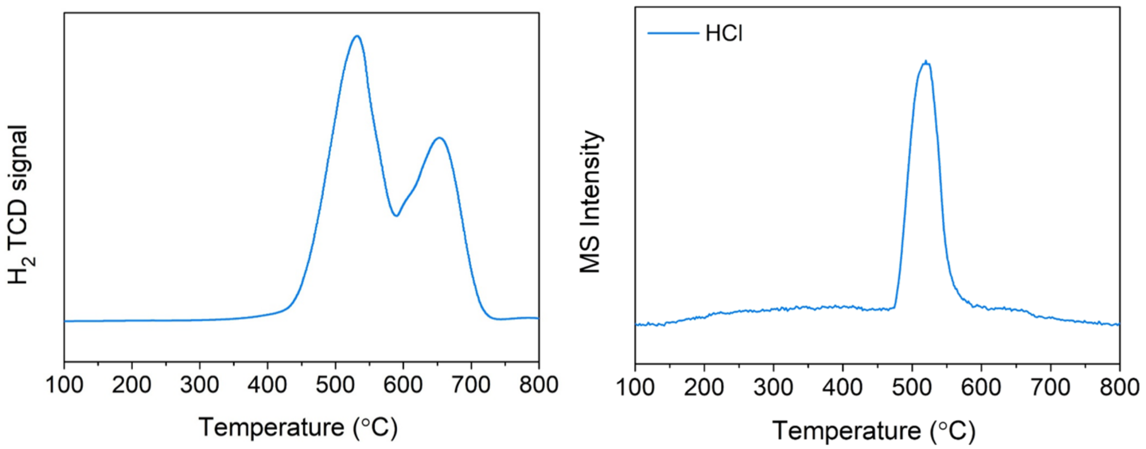

Fig. R33. The H_2-TPR-MS results of the yellow crystals.

4) Ex-situ time-dependent optical images and XRD patterns of PVC decomposition catalyzed by FeC-FeNi/Ni/CNTs further reveal details of the FeNi alloy formation process. In this process, Cl generated from PVC decomposition induces the conversion of Fe and Ni into metal chlorides. Subsequently, under the action of hydrogen produced from PVC decomposition, these metal chlorides are reduced and converted into FeNi alloys, thereby completing catalyst reconstruction. This process is manifested in **Fig. R34** as the gradual exposure of tiny white crystals, which subsequently disappear over time. Simultaneously, the corresponding XRD shows diffraction peaks of the FeNi alloy (**Fig. R35**).

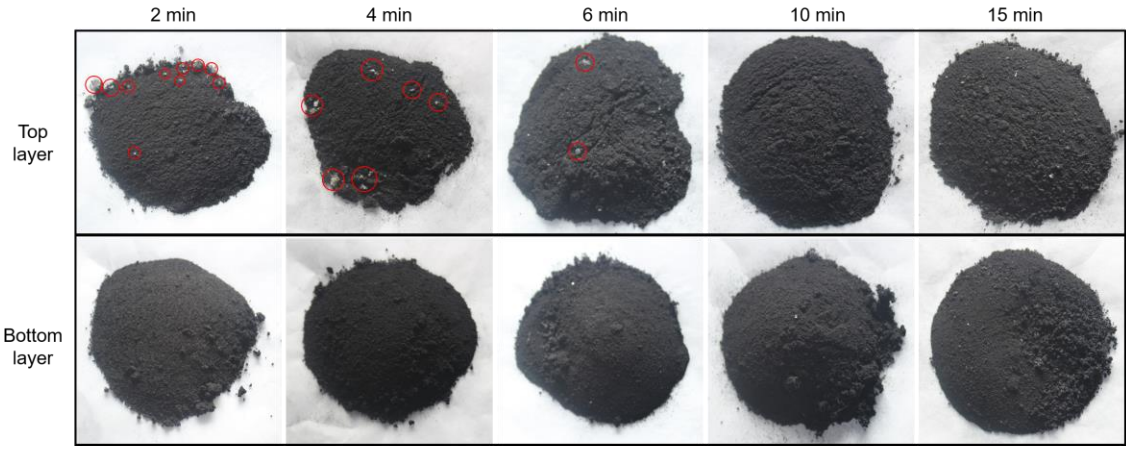

Fig. R34. Time-dependent optical images of PVC decomposition catalyzed by FeC-FeNi/Ni/CNTs. The circled white crystals are metal chlorides, while the black substance is the catalyst.

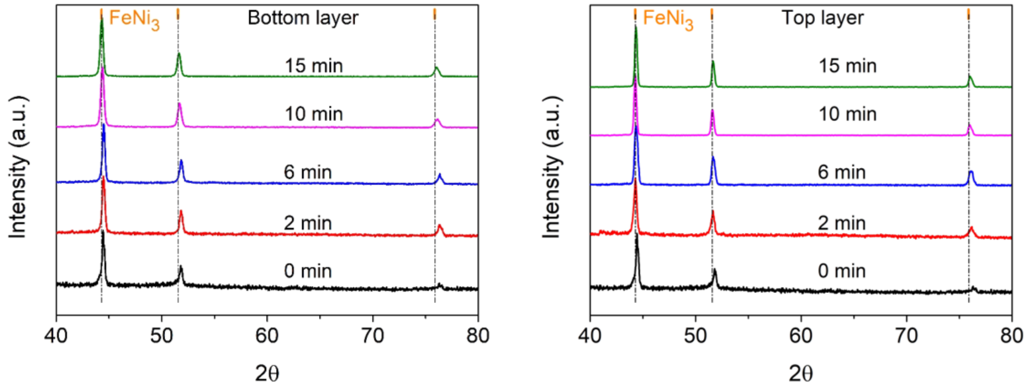

Fig. R35. Time-dependent XRD patterns of PVC decomposition catalyzed by FeC-FeNi/Ni/CNTs.

Figs. R30-35 have been labelled as Supplementary Figs. 28-31 in the supplementary material.

Reviewer #3: The presented work is interesting; however, the current form of this article requires that the following observation be addressed for publication.

1. The novelty of this work in terms of new mechanistic insight and comprehensive understanding of polymer degradation processes has not been included.

Response: We sincerely appreciate the reviewer's constructive comment and the reaction mechanism and polymer degradation process have been extensively investigated and presented below. Firstly, a detailed mechanism for the in situ active catalyst was proposed, and the FeC-FeNi/Ni/CNTs catalyst precursor was reconstituted into a highly active and stable FeNi/Ni/CNTs catalyst via PVC decomposition process. The construction of FeNi alloy in the PVC-assisted FeC-FeNi/Ni/CNTs catalyst transformation originates from hydrogen reduction during plastic decomposition. Hydrogen generated during plastic decomposition reduces the catalyst to produce water molecules, which combine with hydrogen chloride from PVC decomposition to form hydrochloric acid molecules. These hydrochloric acid molecules thereby effectively convert Ni and Fe into nickel chloride and iron chloride. Subsequently, chlorine is removed from the formed nickel chloride and iron chloride as hydrogen chloride under the action of hydrogen, thereby facilitating the formation of FeNi alloy.

This discussion is step by step evidenced by the following new studies (**Figs. 36-40**). **Fig. R36** presents the H_2-TPR-MS results of the FeC-FeNi/Ni/CNTs catalyst precursor, and the

mass spectrometry results show that water molecules are generated with the hydrogen reduction process, providing evidence for hydrogen acting as a reducing medium in the reaction.

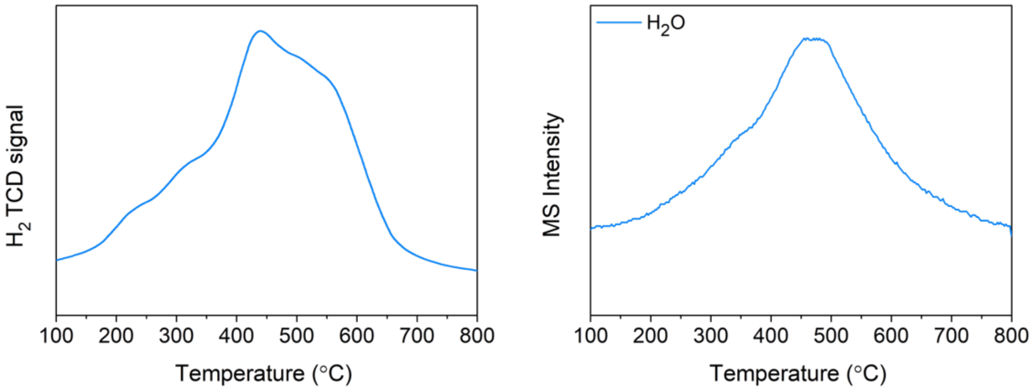

Fig. R36. The H_2-TPR-MS results of the FeC-FeNi/Ni/CNTs catalyst precursor.

To verify whether PVC decomposition can promote the formation of iron chloride and nickel chloride, the following new indirect observation was conducted: 5 g of FeC-FeNi/Ni/CNTs and 5 g of PVC were mixed uniformly and reacted under 600 W microwave power for 10 minutes. Subsequently, 30 mL of deionized water was added and stirred at 25 °C for 2 hours. Afterwards, the above mixture was filtered, and the filtrate was dried completely in an oven at 100 °C, ultimately obtaining yellow crystals (**Fig. R37a**). XRD results of the yellow crystals indicate that they are composed of FeClx and $NiCl_2$, indicating that PVC decomposition can promote the formation of FeClx and $NiCl_2$ (**Fig. R37b**).

To confirm that $NiCl_2$ and FeClx can form FeNi alloy under hydrogen, 0.3 g of yellow crystals and 0.1 g of commercial multi-walled carbon nanotubes were mixed uniformly and heated under 600 W microwave power and 10% H_2 atmosphere (flow rate 300 ml/min) for 10 minutes. It is found that FeNi alloy is formed (**Fig. R37c**). Meanwhile, H_2-TPR-MS during the treatment of the obtained yellow crystals reveals the production of HCl, which is consistent with the aforementioned process of $NiCl_2$ and FeClx removing chlorine atoms under hydrogen (**Fig. R37d**). These results support the above discussion regarding the PVC-assisted reconstruction of the catalyst to form FeNi alloy.

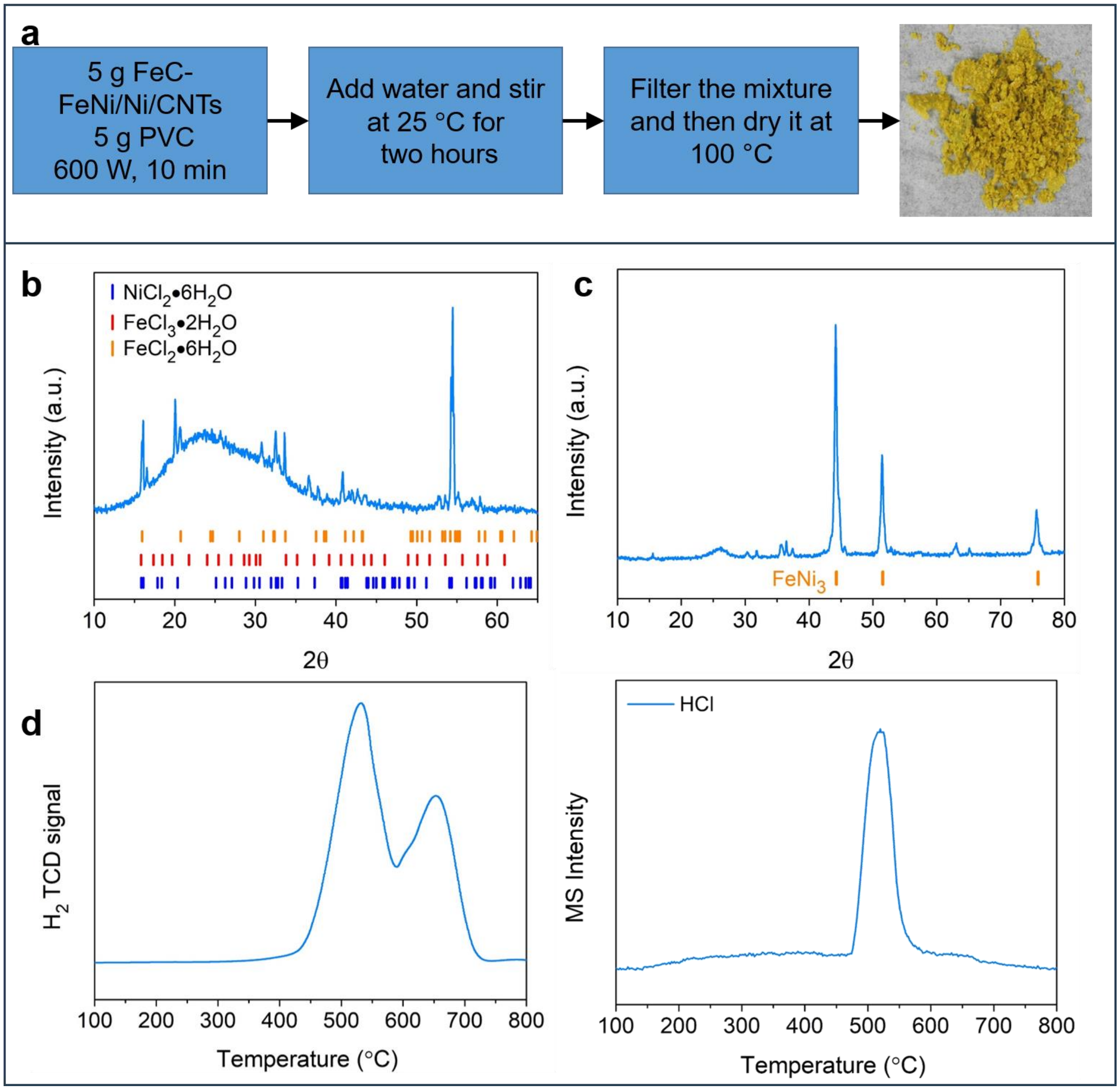

Fig. R37. (a) Schematic diagram of FeC-FeNi/Ni/CNTs and PVC reaction and post-treatment. (b) XRD results of the yellow crystals. (c) XRD results of the yellow crystals after being treated with H_2. (d) The H_2-TPR-MS results of the yellow crystals.

Ex-situ time-dependent optical images and XRD patterns of PVC decomposition catalyzed by FeC-FeNi/Ni/CNTs further reveal details of the FeNi alloy formation process. In this process, Cl generated from PVC decomposition induces the conversion of Fe and Ni into metal chlorides. Subsequently, under the action of hydrogen produced from PVC decomposition, these metal chlorides are reduced and converted into FeNi alloys, thereby completing catalyst reconstruction. This process is manifested in **Fig. R38** as the gradual exposure of tiny white crystals, which subsequently disappear over time. Simultaneously, the corresponding XRD shows diffraction peaks of the FeNi alloy (**Fig. R39**).

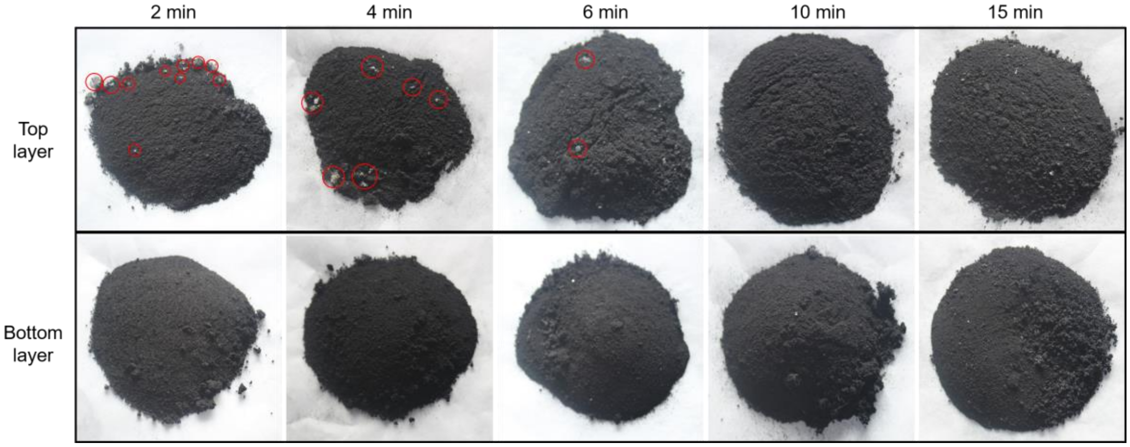

Fig. R38. Time-dependent optical images of PVC decomposition catalyzed by FeC-FeNi/Ni/CNTs. The circled white crystals are metal chlorides, while the black substance is the catalyst.

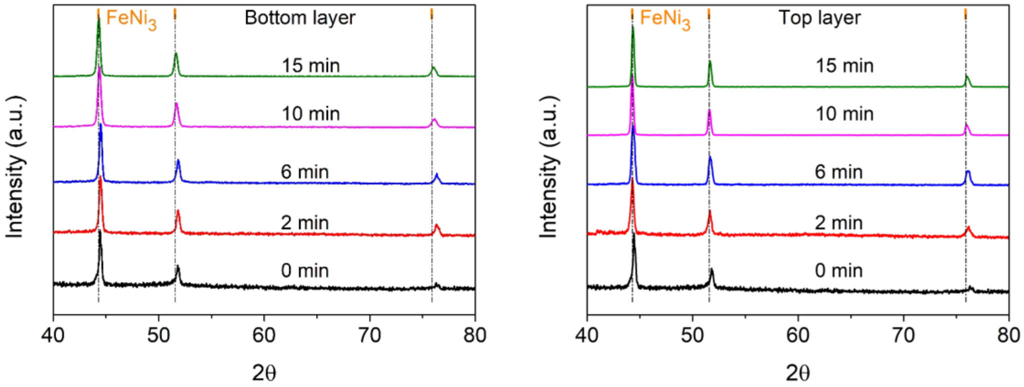

Fig. R39. Time-dependent XRD patterns of PVC decomposition catalyzed by FeC-FeNi/Ni/CNTs.

Second, in the tandem catalytic production of H_2 and CNTs from polymers, polymer degradation involves two steps: a bottom FeNi/Ni/CNTs catalyst layer first decomposes plastics into hydrogen and carbon, with accompanying C-C bond cleavage generating small-molecule hydrocarbons that escape with hydrogen (**Fig. R40**). These small-molecule hydrocarbons are then further dehydrogenated by the top catalyst layer, thereby enhancing hydrogen purity, hydrogen efficiency, and carbon efficiency. This is supported by **Fig. R41**, in the tandem design, the catalytic system exhibits consistent performance: over 95 wt% of the plastic mixture is converted into gas and solid carbon in each cycle, with only small amounts of organic by-products generated. H_2 efficiency between from 85.7% and 98.2% with a purity of 89.4-97 vol%, while carbon efficiency ranges from 83.3% to 99.9%. Corresponding yields of H_2 and CNTs reach 930 mmol $g^{-1}_{catalyst}$ and 10608 mg $g^{-1}_{catalyst}$,

respectively (**Fig. R41**). In contrast, in the presence of the bottom layer, hydrogen efficiency is 67-92% (purity: 87-96 vol%) and carbon efficiency is 63-89%. The respective H_2 and carbon yields are 812.9 mmol $g^{-1}_{catalyst}$ and 8743 mg $g^{-1}_{catalyst}$, which are significantly lower than those of the tandem design (**Fig. R42**). Air atmosphere thermogravimetric analysis reveals increased carbon content in both top and bottom catalyst layers with cycling, suggesting the top catalyst facilitates dehydrogenation of hydrocarbons generated by the bottom catalyst, improving the catalytic efficiency (**Fig. R43**). In addition, thermogravimetric mass spectrometry (TG-MS) results further support the above discussion: relative to the tandem catalytic mode, the standalone catalyst-plastic mixture mode produces less hydrogen and more hydrocarbons in the 10-90 m/z range (**Fig. R44**). This further indicates that the tandem mode can further catalyze the hydrocarbons generated during the reaction in the bottom layer, thereby enhancing hydrogen and carbon efficiency.

Moreover, during polymer decomposition, hydrogen in C_XH_Y species is continuously catalyzed by Ni (which exhibits high C-H activation activity) in FeNi/Ni/CNTs to form H_2, while Fe (with higher carbon solubility in FeNi/Ni/CNTs) binds to C atoms, promoting carbon diffusion and crystallization into carbon nanotubes (**Fig. R45**) (J. Am. Chem. Soc., 147, 38141-38157 (2025). Energy 262, 125415 (2023). Applied Catalysis B: Environmental 208, 44-59 (2017). Journal of Catalysis 222, 250-254 (2004)). Hydrogen chloride formed during the reaction maintains the catalyst's long-term stability by inducing FeNi alloy formation (**Figs. R36-40**), while fluoride sustains stability by shearing carbon nanotubes to re-expose active sites (**Fig. R46**). In addition, XAS results indicate that there is an enhanced electron cloud density of Fe and Ni, along with electron transfer from Fe to Ni (**Fig. R47**). Combined with the DFT calculation results, FeNi synergistically reduced the activation energy of the reaction, promoting plastic and small-molecule hydrocarbon adsorption, C-H activation, and overall catalytic activity. This contributes to enhanced hydrogen production from waste plastics, carbon nanotube formation, and improved catalytic stability (**Fig. R41**) (J. Am. Chem. Soc., 147, 38141-38157 (2025). Angew. Chem. Int. Ed., 61, e202202518 (2022)).

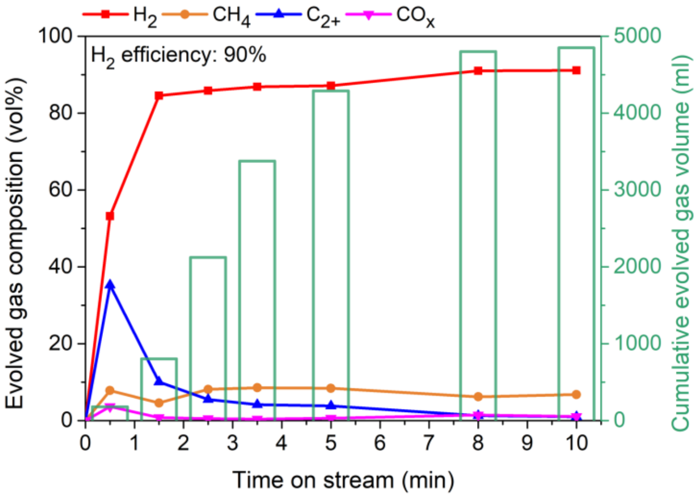

Fig. R40. A time-on-stream analysis of the product distribution.

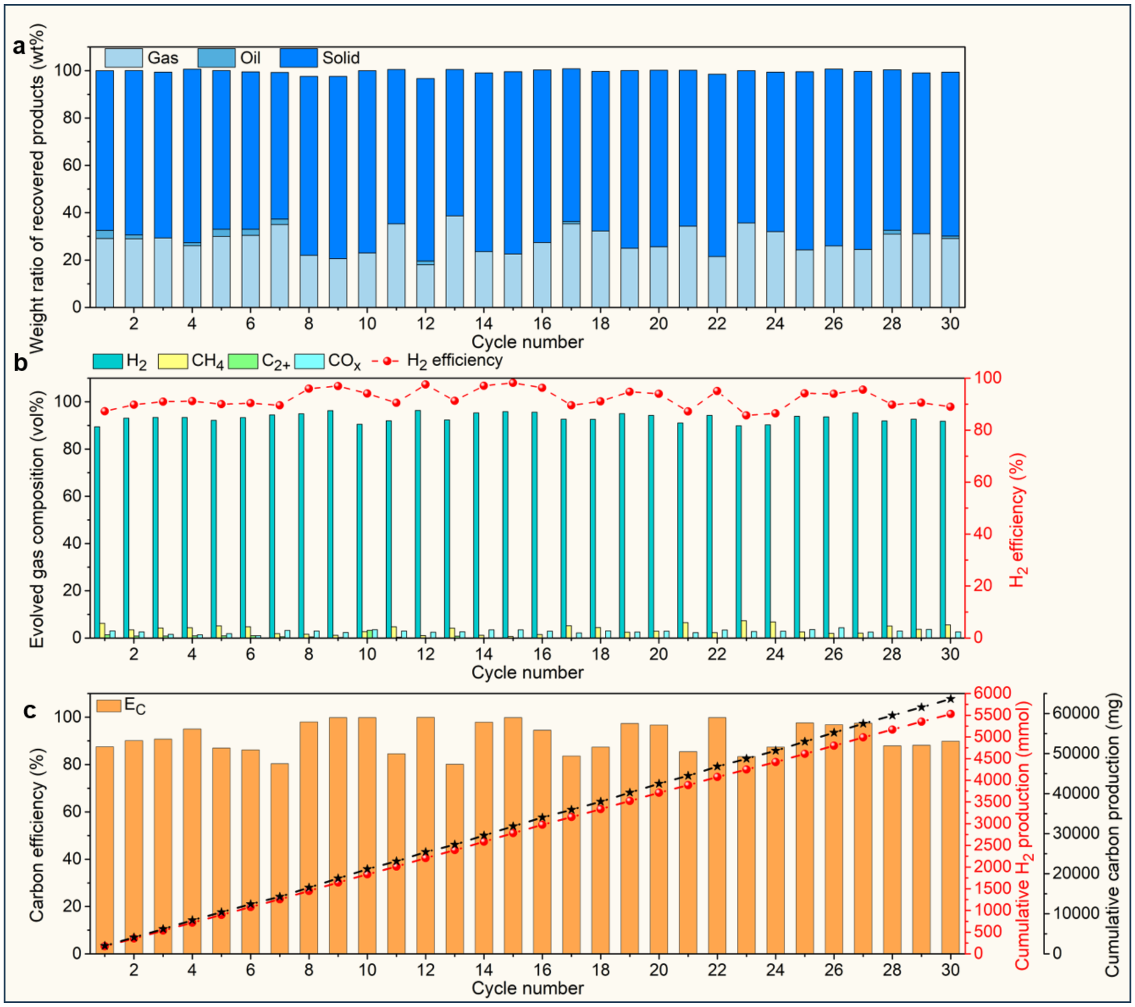

Fig. R41. Successive cycles of microwave catalytic decomposition of plastic over FeNi/Ni/CNTs catalyst. (a) The weight ratio of recovered gas, oil and solid. (b) Evolved gas composition (vol%) and H_2 efficiency (%). (c) Carbon efficiency (%) (E_C), cumulative H_2 production (mmol) and cumulative carbon production (mg). Reaction conditions: 3 g plastic and 3 g FeNi/Ni/CNTs catalyst in the bottom layer; additional 3 g FeNi/Ni/CNTs catalyst in the top layer. Plastic: LDPE and PVC mixture (the mass ratio of LDPE to PVC is 9:1).

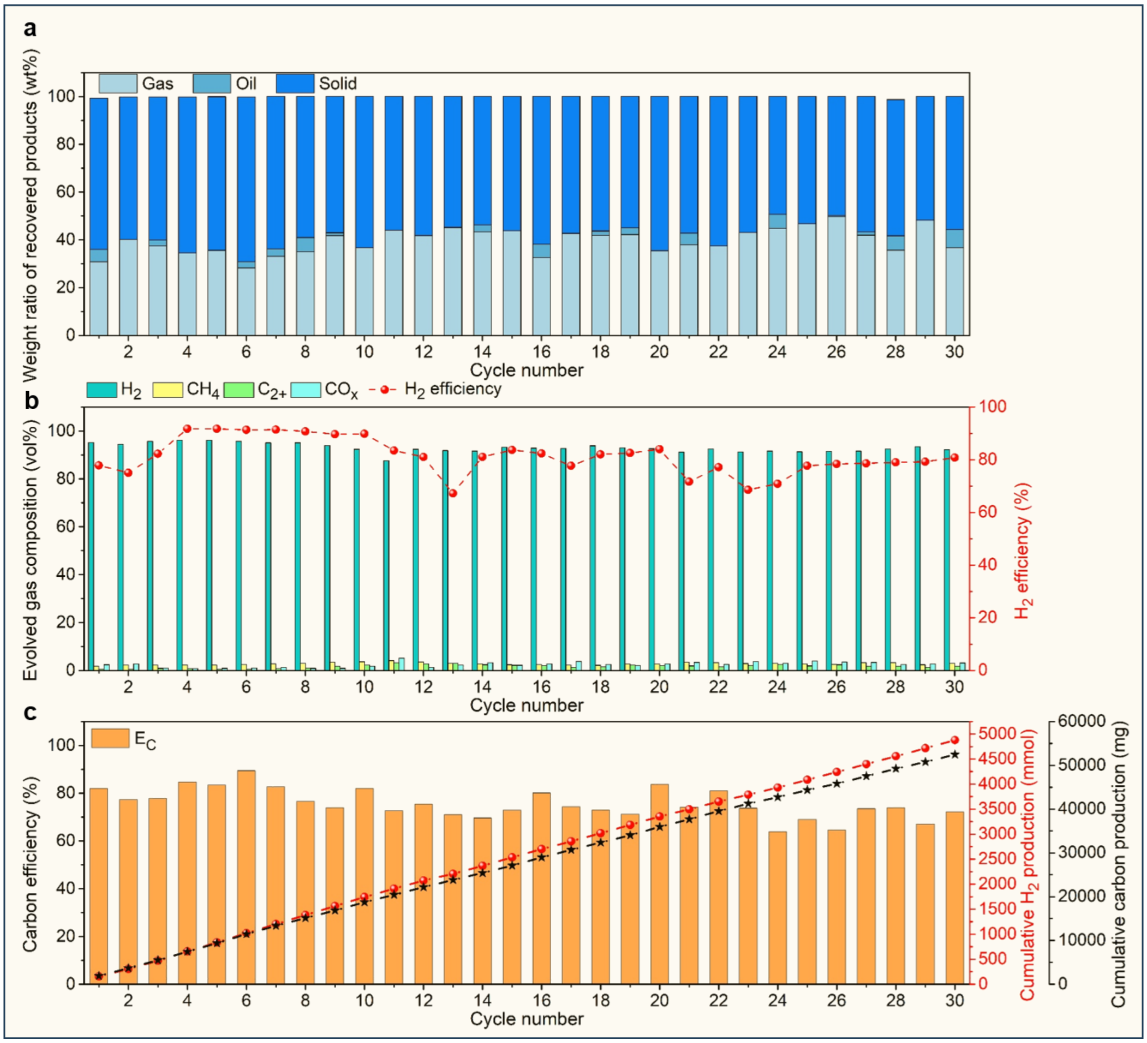

Fig. R42. Successive cycles of microwave catalytic decomposition of plastic over FeNi/Ni/CNTs catalyst. (a) The weight ratio of recovered gas, oil and solid. (b) Evolved gas composition (vol%) and H_2 efficiency (%). (c) Carbon efficiency (%) (E_C), cumulative H_2 production (mmol) and cumulative carbon production (mg). Reaction conditions: A mixture of 3 g plastic and 6 g FeNi/Ni/CNTs catalyst. Plastic: LDPE and PVC mixture (the mass ratio of LDPE to PVC is 9:1).

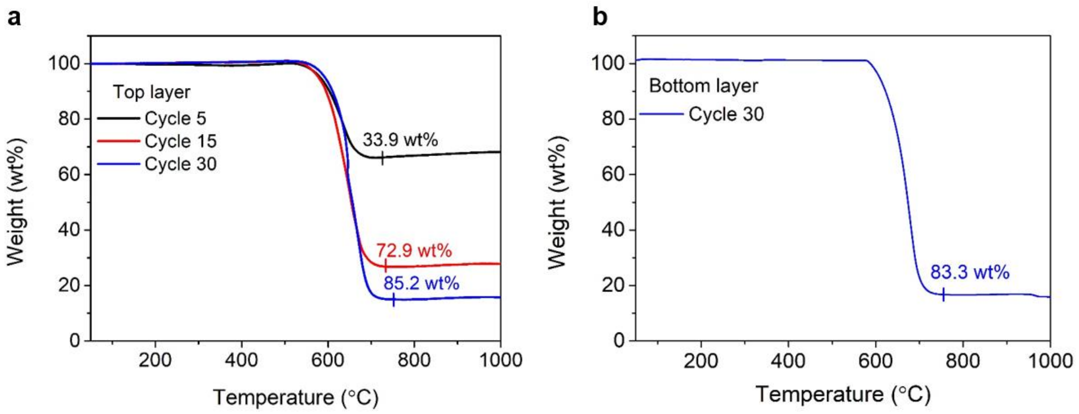

Fig. R43. Thermogravimetric analysis of the top and bottom catalyst after use.

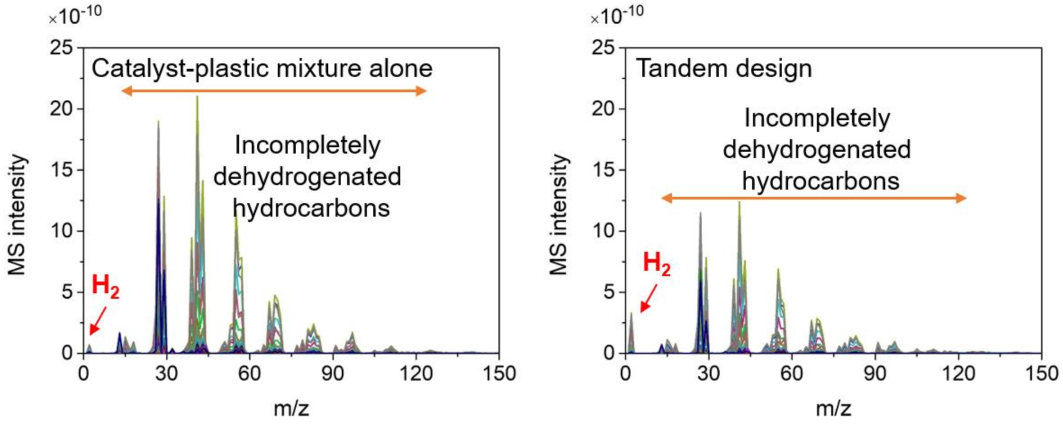

Fig. R44. TG-MS analysis of the differences in the product distribution of only catalyst-plastic mixture reaction modes and tandem catalytic modes.

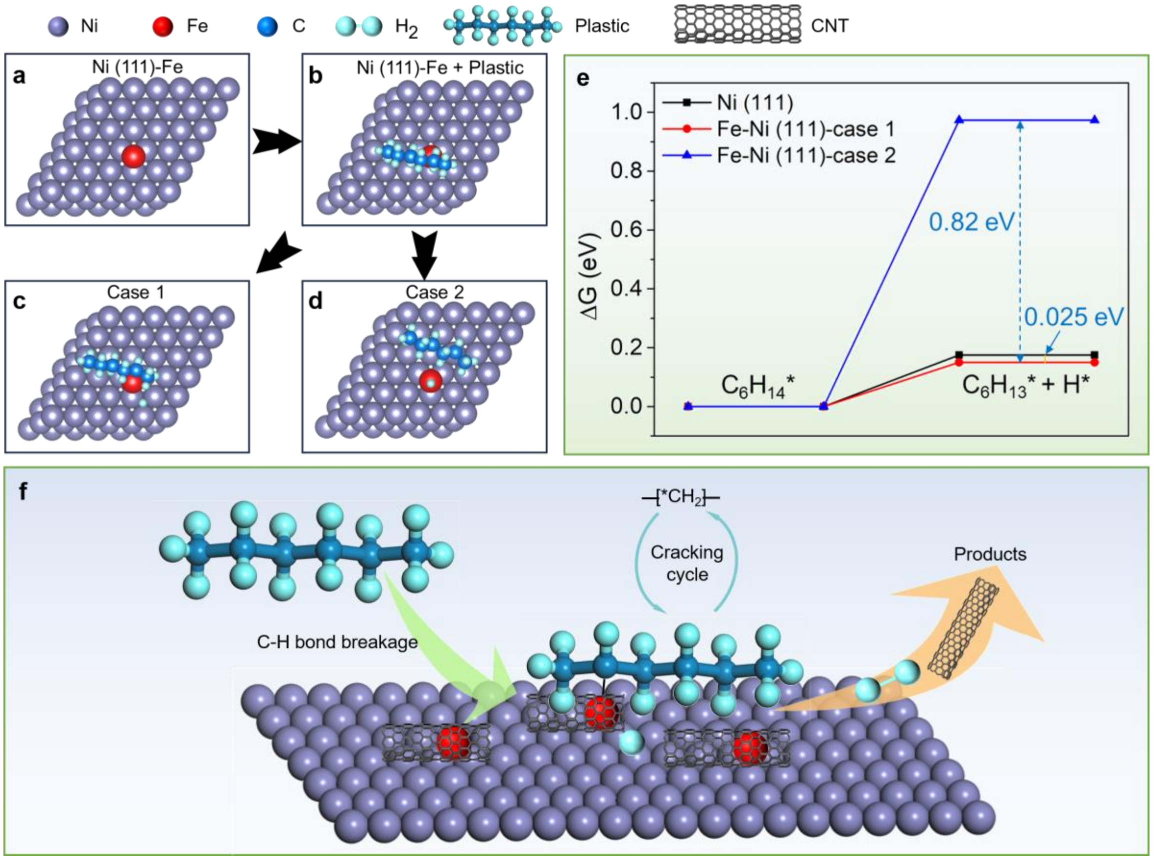

Fig. R45. Proposed mechanism. The model of (a) Ni-Fe, (b) Ni-Fe + plastic, (c) case1 and (d) case 2. (e) The calculated free-energy changes for C-H bond breaking reaction of C_6H_{14} on Ni (111), Fe-Ni (111)-case 1 and Fe-Ni (111)-case 2. (f) An illustration on microwave catalytic upcycling of plastic over FeNi/Ni/CNTs catalyst.

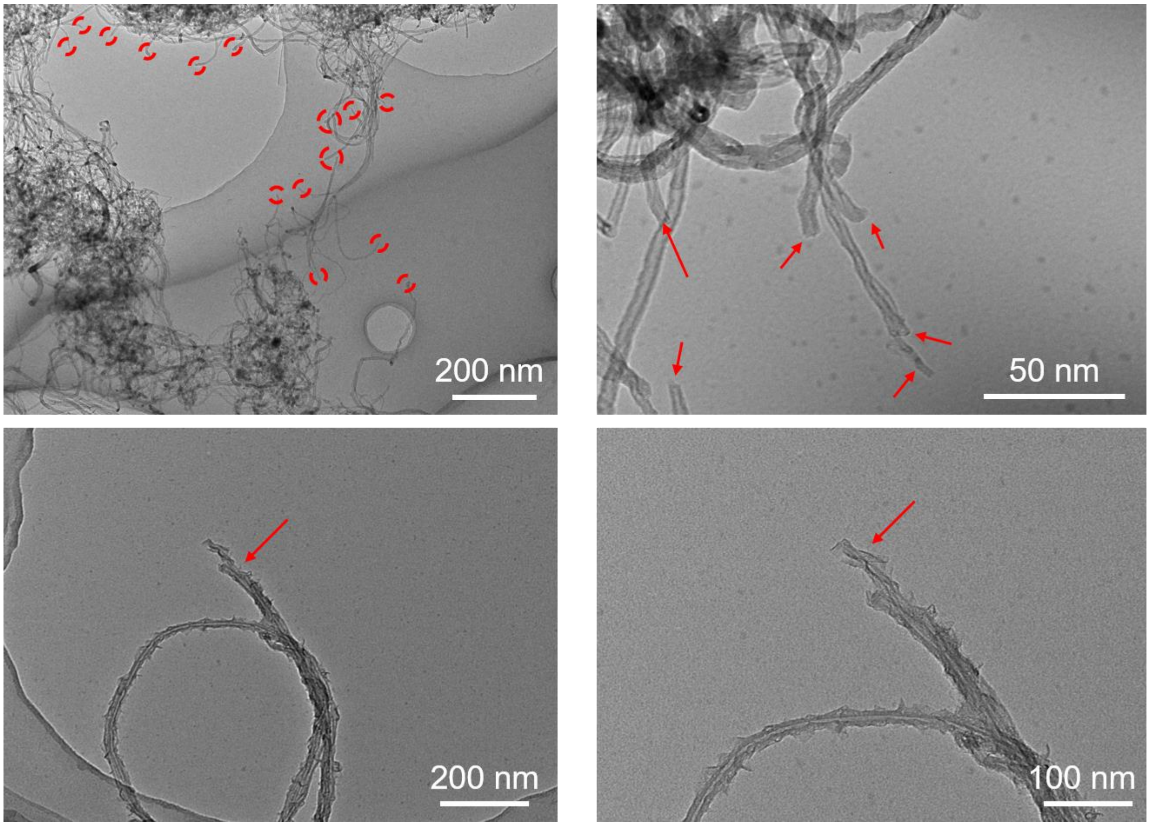

Fig. R46. The TEM images of the CNTs after the reaction with PTFE.

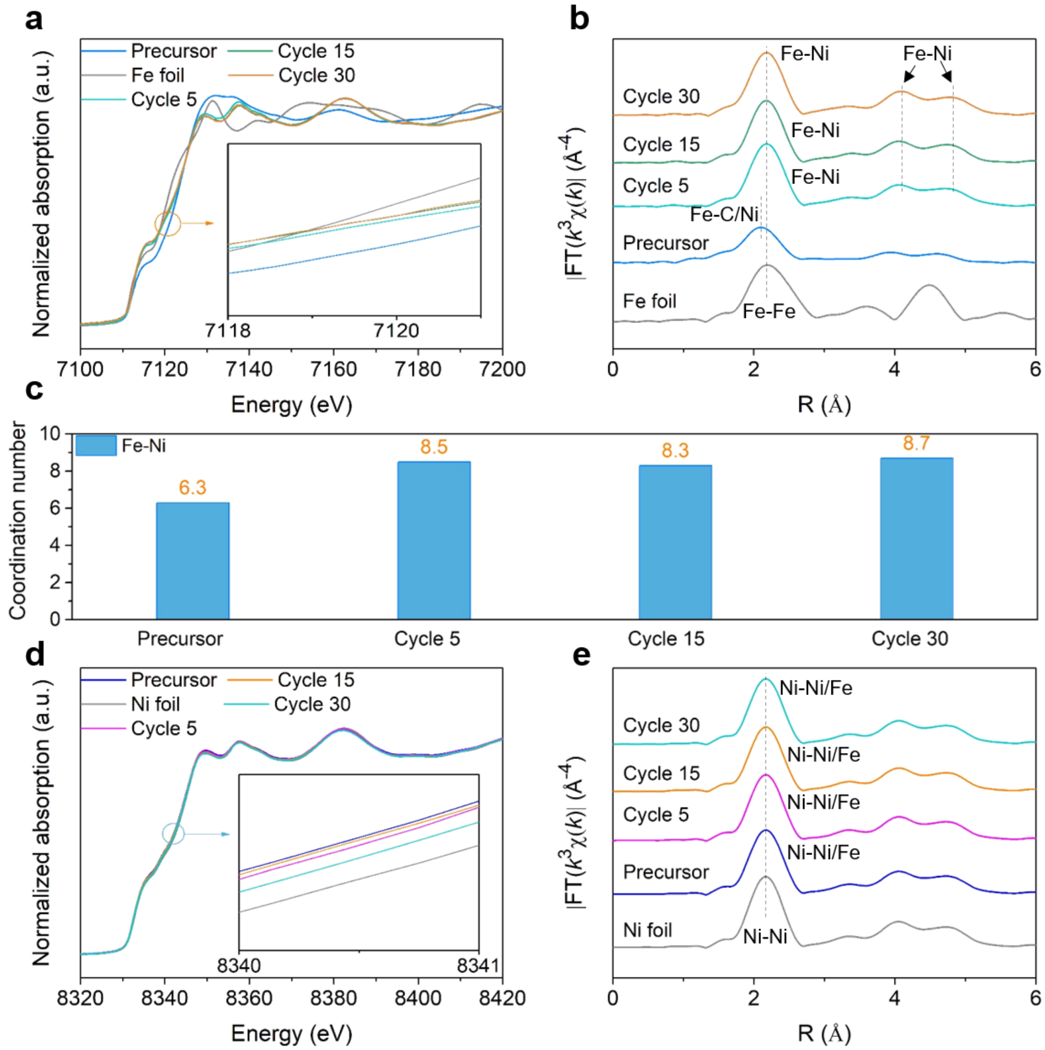

Fig. R47. XAS analysis. (a) Fe K-edge XANES spectra of FeC-FeNi/Ni/CNTs (precursor) and FeNi/Ni/CNTs catalysts as well as references. (b) Fourier transforms (FT) of Fe EXAFS spectra of FeC-FeNi/Ni/CNTs and FeNi/Ni/CNTs catalysts and references. (c) Changes of coordination number in FeC-FeNi/Ni/CNTs and FeNi/Ni/CNTs catalysts. (d) Ni K-edge XANES spectra of FeC-FeNi/Ni/CNTs and FeNi/Ni/CNTs catalysts as well as references. (e) Fourier transforms (FT) of EXAFS spectra (Ni) of FeC-FeNi/Ni/CNTs and FeNi/Ni/CNTs catalysts and references.

Fig. R36, Fig. R37, Fig. R38, Fig. R39, Fig. R40, Fig. R41, Fig. R42, Fig. R43, Fig. R44, Fig. R45, Fig. R46 and Fig. R47 have been labelled as Supplementary Fig. 28, Supplementary Fig. 29, Supplementary Fig. 30, Supplementary Fig. 31, Supplementary Fig. 2b, Fig. 1, Supplementary Fig. 8, Supplementary Fig. 9, Supplementary Fig. 10, Fig. 5, Supplementary Fig. 38, and Fig. 3 in the manuscript and supplementary material.

2. The abstract seems very superficial; it does not highlight innovative results that were obtained in summary. Although the title seems generic, the catalyst played a key role in this conversion process, so why wasn't it included?

Response: We thank the reviewer for raising this point. The original title was shortened as we were worried about the limit of word in the title.

To include all key information, the new title is "**100% upcycling of chlorinated/fluorinated plastic waste mixtures to nanotubes over FeNi/Ni/CNTs by microwave irradiation**"

The rather summary abstract was revised as

The catalytic upcycling of plastic waste mixtures into H_2 and carbon nanotubes (CNTs) represents a transformative strategy for advancing plastic waste management, carbon neutrality and the circular economy. In this work, an in-situ constructed high-performance FeNi/Ni/CNTs catalyst combined with prompt microwave catalysis enables efficient conversion of LDPE-PVC, LDPE-HDPE-PP-PS-PVC-PTFE and particluary landfilled waste mixture into valuable H_2 and CNTs, resulting into near-quantitative atom economy (i.e. H_2 and carbon efficiencies are nearly 100%) together with H_2 and CNTs yields reaching >930 mmol $g^{-1}_{catalyst}$ and >10600 mg $g^{-1}_{catalyst}$, respectively. Furthermore, this novel microwave catalysis strategy presents an unprecedented stability (>35 cycles) and energy efficiency (80% reduction vs. traditional thermal catalysis), alongside a high turnover number of 107.6 mol_{carbon} mol^{-1}_{NiFe}. Notably, Cl or F species released during microwave catalytic reactions facilitate the regeneration of deactivated catalysts. Fundamentally, the Ni species contribute to C-H breaking in the plastic decomposition and Fe as a cascade catalyst is beneficial for carbon tube formation during the microwave catalysis. These findings can provide a scalable and cost-effective platform for value-added utilization of chlorinated or fluorinated plastic waste mixtures, aligning with global decarbonization and zero-waste imperatives.

3. The structure of introduction is very poor (there is no introduction), says existing histories of plastic degradation lacking an in-depth analysis that explains the innovative aspects of the present work.

Response: We sincerely thank the reviewer for raising the valuable comments. The introduction has been revised. Please see the revised introduction below:

Plastics, predominantly derived from fossil resources, serve as an indispensable material in modern society. Yet over 70% of post-consumer plastic waste is either landfilled or discarded, driving environmental pollution and critical resources underutilization (Science

368, 1055-1055 (2020). Nature 619, 317-322 (2023). Science 368, 1184-1185 (2020)). From a sustainability perspective, plastic waste represents an untapped resource reservoir for synthesizing value-added chemicals (Angewandte Chemie International Edition 59, 15402-15423 (2020)). Among various plastic waste recycling methods, chemical recycling has emerged as a promising strategy due to its flexibility, sustainability and potential for circularity. In recent years, several innovative catalytic upcycling approaches have been proposed. These can selectively convert plastic waste into high-value products, such as lubricants, fuels, H_2, and advanced nano-carbon materials (Nature Reviews Chemistry 6, 635-652 (2022). Chem 7, 1487-1533 (2021). Science 381, 607-608 (2023). Nature Catalysis 6, 161-173 (2023). Science 379, 807-811 (2023). Nature Communications 16, 1726 (2025). Applied Catalysis B: Environmental 318, 121828 (2022).), offering a promising strategy for the upcycling of plastic waste.

Carbon nanotubes (CNTs) composed of hexagonally arranged carbon atoms forming cylindrical architectures are distinctive carbon-based nanostructures. They possess high electrical conductivity, exceptional tensile strength, and low density, supporting broad applications in power storage, aerospace engineering, electronics, semiconductors, flexible/wearable devices, and environmental technologies (Science 339, 535-539 (2013). Chem. Soc. Rev. 44, 250-290 (2015).). The global CNT market currently stands at approximately $12.5 billion, projected to exceed $35.8 billion by 2034 with a 12.4% compound annual growth rate (https://www.gminsights.com/industry-analysis/carbon-nanotubes-market). Against this market backdrop, sustainable CNT synthesis has emerged as a critical industrial priority.

Chemical vapor deposition (CVD) dominates current CNT production due to its operational simplicity and technical maturity. This process synthesizes CNTs by catalytically decomposing volatile precursors such as methane, ethylene, acetylene, benzene, xylene and toluene, which are produced from fossil fuels (Advanced Functional Materials 32, 2108541 (2022).). However, in the context of sustainable carbon materials development, substituting fossil-derived feedstocks and addressing energy-intensive requirements in CVD are critical for future low carbon CNT production.

Waste plastics, characterized by low carbon-to-hydrogen ratios (0-1:2 for polymers like PE, PP, PS, PVC and PTFE), represent a significantly underutilized carbon resource, with annual global waste exceeding 370 million tons (Chem. Soc. Rev. 44, 250-290 (2015). https://plasticseurope.org/es/knowledge-hub/plastics-the-fast-facts-2024/). This underscores their substantial potential as sustainable carbon feedstocks for CNT production. Furthermore, H_2 can be produced as another valuable chemical during plastic conversion.

As an alternative to CVD, catalytic decomposition enables one-step conversion of plastic waste at moderate temperatures (Nature Catalysis 3, 902-912 (2020). Chemical Engineering Journal 483, 149270 (2024).). Therefore, the catalytic synthesis of H_2 and

CNT from plastic wastes offers a promising alternative. This dual-output approach supports sustainable CNT production by valorizing plastic waste and reducing reliance on petrochemical precursors. However, to convert chlorine or fluorine -containing plastic is very challenging, in particular the durability of the catalyst.

Herein, a prompt strategy with a tandem catalyst structure was developed for upcycling chlorinated/fluorinated plastic waste mixtures into H_2 and CNTs using an in-situ constructed FeNi/Ni/CNTs catalyst under microwave promotion. The FeC-FeNi/Ni/CNTs were used as a precursor and synthesized via a solvent-free, rapid and energy-efficient microwave method. During upcycling, HCl released from PVC regenerates the catalyst by reconstructing the active FeNi/Ni/CNTs catalyst, enabling both catalytic lifespan (>35 cycles) and the high activity. Concurrently, fluoride species from PTFE decomposition cleave C-C bonds and expose fresh catalytic sites, amplifying efficiency. Ultimately, this well-designed process achieves H_2 yields of >930 mmol $g^{-1}_{catalyst}$ (purity of 87-98 vol%) and CNTs yields of >10600 mg $g^{-1}_{catalyst}$, ca. 100% H_2 and carbon efficiency after upcycling of complex plastic mixtures (LDPE-PVC or LDPE-HDPE-PP-PS-PVC-PTFE), while reducing energy consumption by 80% versus conventional thermal catalysis. The dual role of chlorinated/fluorinated plastic mixtures, as both feedstocks and in situ regenerants, enables long-term operation without supplementing additional fresh catalysts. This innovative strategy offers a promising and practical pathway for the treatment and valorization of chlorinated/fluorinated plastic waste mixtures.

4. The materials and methodology are presented in supplementary; it should be in the main article so that they can help researchers to reproduce.

Response: We appreciate the valuable suggestion provided by the reviewer. The materials and methodology have been moved to the main manuscript.

5. What is 100% conversion equivalent to, what is defined as yield in terms of a specific compound?

Response: We appreciate the reviewer for raising this important point. The 100% conversion equals to complete conversion of plastic wastes, so the sum of gas, oil, and carbon masses matches the initial plastic input mass, indicating a mass balance of ~100%. H_2 or CNT production yields are calculated by normalizing the total H_2 or CNT amounts generated following 30 consecutive cycles of plastic waste decomposition to the FeNi/Ni/CNTs catalyst mass. Please see the calculation formula below (Manuscript **Page 16**):

$$\text{Conversion}=\frac{\text{Total mass of gas, oil and carbon (g)}}{\text{Mass of plastic (g)}}\times 100\ (\%)$$

$$H_2\ \text{yield}=\frac{\sum_1^n \text{Efficiency}_{H_2\,n}\times \text{Theoretical molar content of } H_2 \text{ in plastic}_n}{\text{Mass of catalyst}}\ (\text{mmol g}^{-1}_{\text{catalyst}})$$

$$\text{CNT yield}=\frac{\sum_1^n \text{Efficiency}_{C\,n}\times \text{Theoretical mass content of C in plastic}_n}{\text{Mass of catalyst}}\times \text{CNT purity}\ (\text{mg g}^{-1}_{\text{catalyst}})$$

Herein, CNT purity refers to the purity of CNTs in the carbon material from the final cycle, as determined by thermogravimetric analysis. The theoretical molar content of H_2 in plastics was calculated by multiplying the respective masses of the plastic mixture by the sum of their corresponding hydrogen contents, then dividing by hydrogen's molecular weight. The theoretical carbon content was determined by multiplying the respective masses of the plastic mixture by the sum of their corresponding carbon contents, then dividing by carbon's atomic weight (Nature Catalysis 3, 902-912 (2020). Applied Catalysis B: Environment and Energy 378, 125616 (2025)).

6. The purification of FeNi-integrated carbon nanotubes is not discussed.

Response: We sincerely appreciate the reviewer's feedback. To further purify the resulting CNTs, they were pretreated at different temperatures (450 °C, 500 °C, 550 °C and 600 °C) in an air atmosphere (0.5 h) to expose the embedded metal catalysts and facilitate the subsequent acid dissolution (25 °C, 2 h) process. **Fig. R48** shows that no significant mass loss is observed when the CNTs are processed at 450 °C or 500 °C, which is insufficient for exposing the metal species. When the temperature is increased to 550 °C, a mass loss of 2.5 wt% is observed, indicating the metal species are likely to be successfully exposed. However, the mass loss rises sharply to 37 wt% at 600 °C, suggesting excessive oxidation that may damage the CNT structure. Meanwhile, although 600 °C also oxidizes metal species to metal oxides, it is difficult to remove them by acid treatment, which may be due to the sintering of metal species caused by high processing temperatures. Therefore, a treatment temperature of 550 °C is determined to be optimal as it balances metal exposure and preserves the CNT structure, as illustrated in **Figs. R49-56**.

Carbon purity from LDPE, LDPE-PVC, LDPE-HDPE-PP-PS-PVC-PTFE, and landfill waste plastic increases from 94.5 wt%, 83.3 wt%, 87/94 wt%, and 88.7 wt% to 97.5 wt%, 92.2 wt%, 93/96.8 wt%, and 92.6 wt%, respectively, following air treatment at 550 °C and subsequent acid leaching. Further qualitative and quantitative analysis of CNT purity (combustion temperature >530 °C) via TGA reveals a purity of LDPE, LDPE-PVC, LDPE-

HDPE-PP-PS-PVC-PTFE, and landfill waste plastic-derived CNTs is 97 wt%, 91 wt%, 92.7/94.5 wt%, and 91.2 wt%, respectively (**Fig. 54** and **Figs. R57-69**).

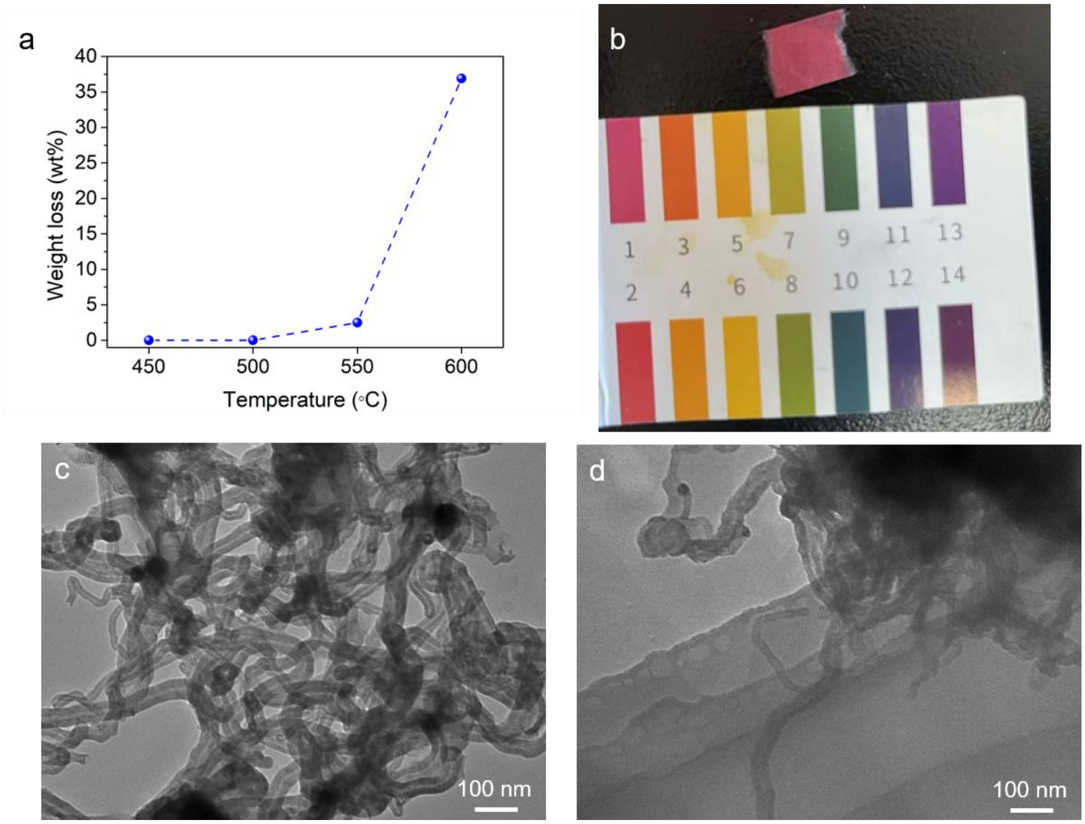

Fig. R48. (a) CNTs' weight loss (Cycle 30) after being processed in an air atmosphere at different temperatures for 0.5 h (450 °C, 500 °C, 550 °C and 600 °C). (b) pH of the aqueous solution (~2.5 mol/L) after Cl and F compounds were absorbed during the reaction. (c) TEM images of the prepared CNTs treated in an air atmosphere at 550 °C for 0.5 h and then acid treated at 50 °C for 2 h. (d) TEM images of the prepared CNTs treated in an air atmosphere at 600 °C and then acid treated at 50 °C for 2 h. CNTs production conditions: using FeNi/Ni/CNTs as the catalyst. Feedstock: LDPE and PVC mixture with a mass ratio of 9:1. 3 g plastic mixture and 3 g FeNi/Ni/CNTs catalyst in the bottom layer; 3 g FeNi/Ni/CNTs catalyst in the top layer.

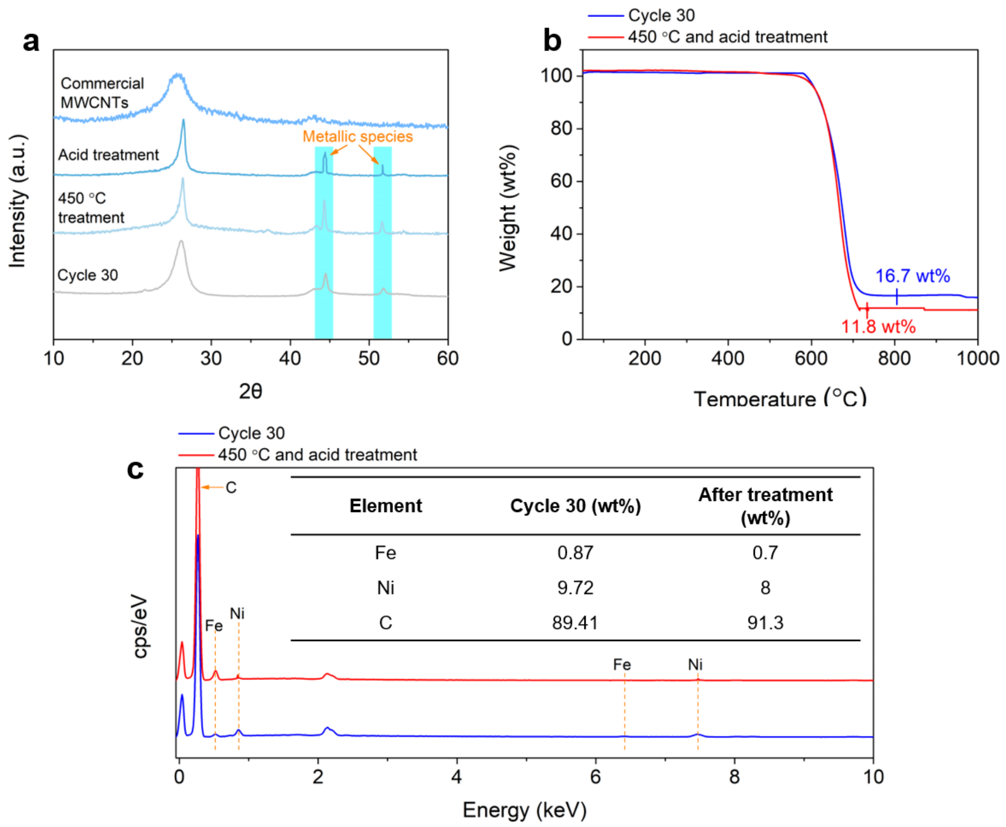

Fig. 49. (**a**) XRD patterns of the prepared CNTs (Cycle 30) before and after treatment, with the XRD pattern of industrial-grade multi-walled carbon nanotubes as a reference. (**b**) The metal content of the CNTs before and after treatment is approximately 16.7 wt% and 11.8 wt%, respectively. (**c**) EDX spectra and elemental composition of the prepared CNTs before and after treatment. The produced CNTs were treated in an air atmosphere at 450 °C for 0.5 h and then acid treated (~2.5 mol/L) at 50 °C for 2 h. CNTs production conditions: using FeNi/Ni/CNTs as the catalyst. Feedstock: LDPE and PVC mixture with a mass ratio of 9:1. 3 g plastic mixture and 3 g FeNi/Ni/CNTs catalyst in the bottom layer; 3 g FeNi/Ni/CNTs catalyst in the top layer.

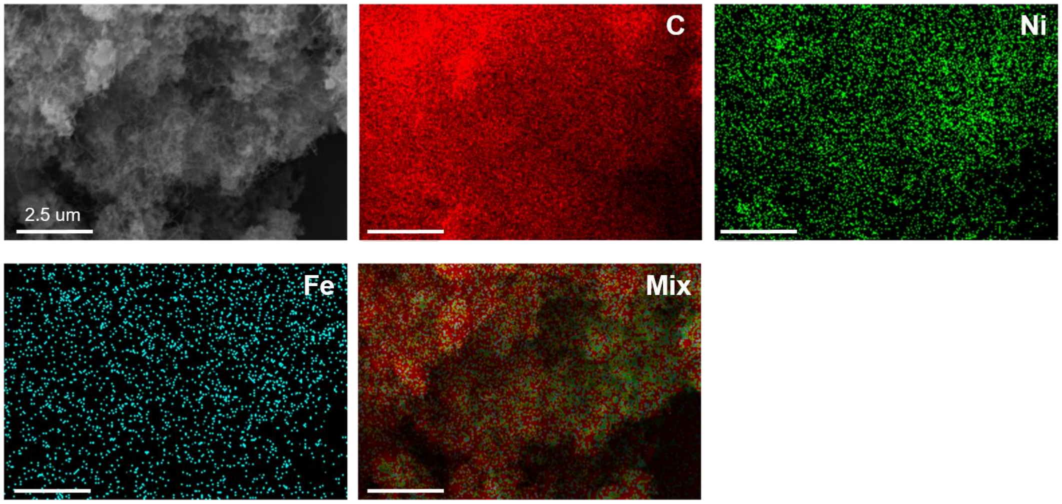

Fig. 50. EDX elemental mapping of the obtained CNTs after 30 successive cycles. CNTs production conditions: using FeNi/Ni/CNTs as the catalyst. Feedstock: LDPE and PVC mixture with a mass ratio of 9:1. 3 g plastic mixture and 3 g FeNi/Ni/CNTs catalyst in the bottom layer; 3 g FeNi/Ni/CNTs catalyst in the top layer.

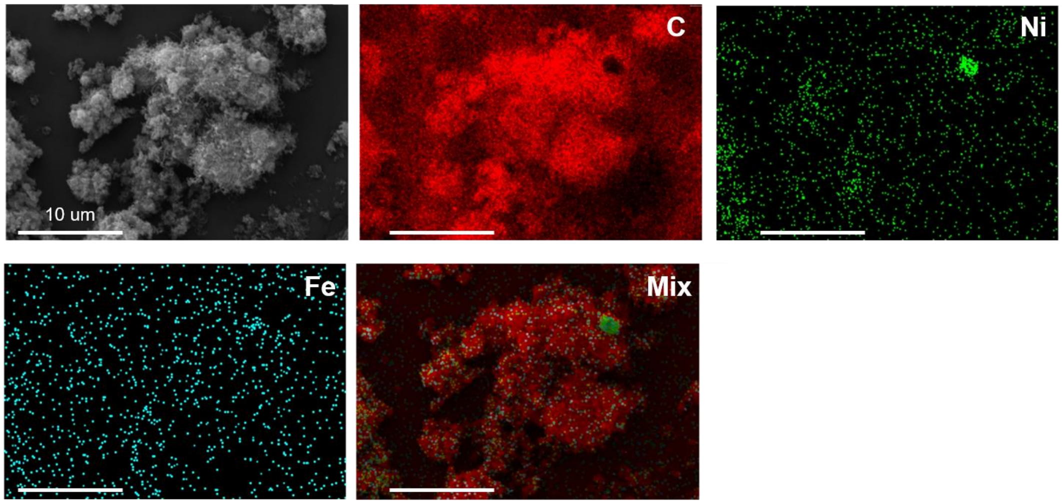

Fig. 51. EDX element mapping of the CNTs obtained after 30 successive cycles. The produced CNTs were treated in an air atmosphere at 450 °C for 0.5 h and then acid treated (~2.5 mol/L) at 50 °C for 2 h. CNTs production conditions: using FeNi/Ni/CNTs as the catalyst. Feedstock: LDPE and PVC mixture with a mass ratio of 9:1. 3 g plastic mixture and 3 g FeNi/Ni/CNTs catalyst in the bottom layer; 3 g FeNi/Ni/CNTs catalyst in the top layer.

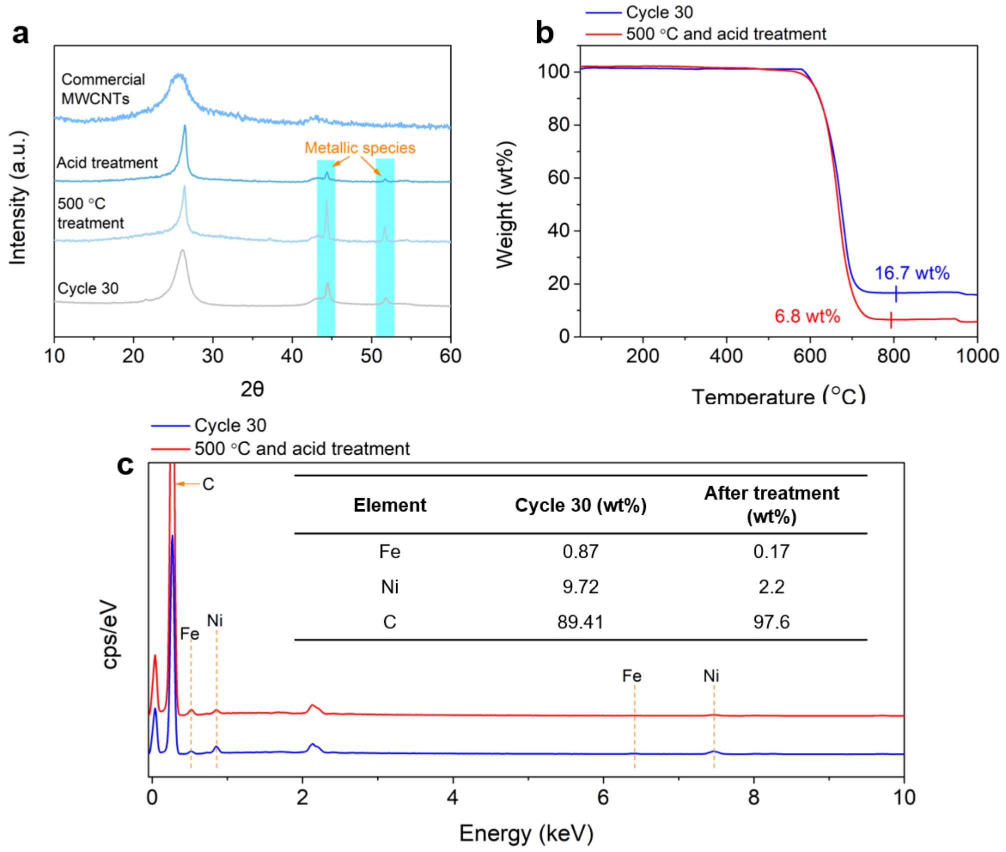

Element	Cycle 30 (wt%)	After treatment (wt%)
Fe	0.87	0.17
Ni	9.72	2.2
C	89.41	97.6

Fig. 52. (**a**) XRD patterns of the prepared CNTs (Cycle 30) before and after treatment, with the XRD pattern of industrial-grade multi-walled carbon nanotubes as a reference. (**b**) The metal content of the CNTs before and after treatment is approximately 16.7 wt% and 6.8 wt%, respectively. (**c**) EDX spectra and elemental composition of the prepared CNTs before and after treatment. The produced CNTs were treated in an air atmosphere at 500 °C for 0.5 h and then acid treated (~2.5 mol/L) at 50 °C for 2 h. CNTs production conditions: using FeNi/Ni/CNTs as the catalyst. Feedstock: LDPE and PVC mixture with a mass ratio of 9:1. 3 g plastic mixture and 3 g FeNi/Ni/CNTs catalyst in the bottom layer; 3 g FeNi/Ni/CNTs catalyst in the top layer.

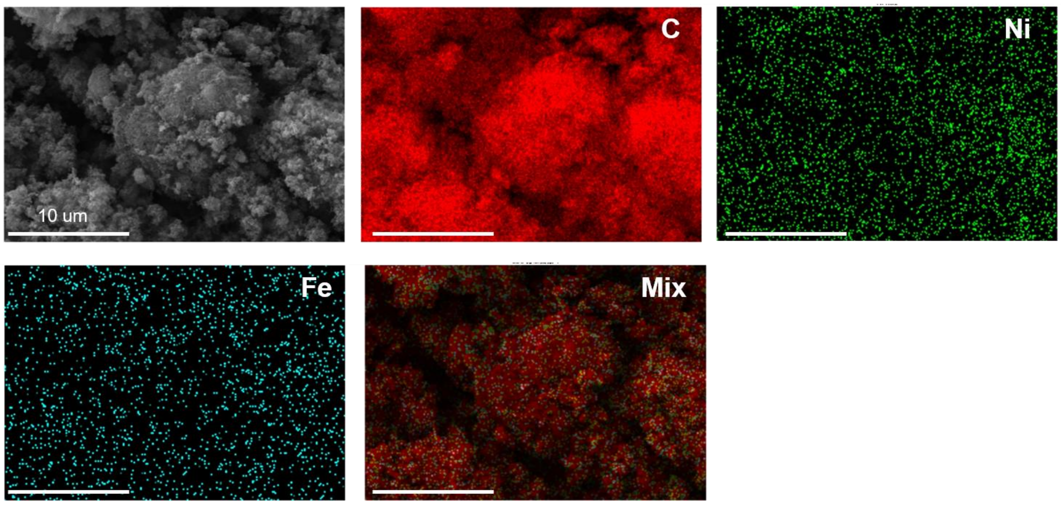

Fig. 53. EDX element mapping of the CNTs obtained after 30 successive cycles. The produced CNTs were treated in an air atmosphere at 500 °C for 0.5 h and then acid treated (~2.5 mol/L) at 50 °C for 2 h. CNTs production conditions: using FeNi/Ni/CNTs as the catalyst. Feedstock: LDPE and PVC mixture with a mass ratio of 9:1. 3 g plastic mixture and 3 g FeNi/Ni/CNTs catalyst in the bottom layer; 3 g FeNi/Ni/CNTs catalyst in the top layer.

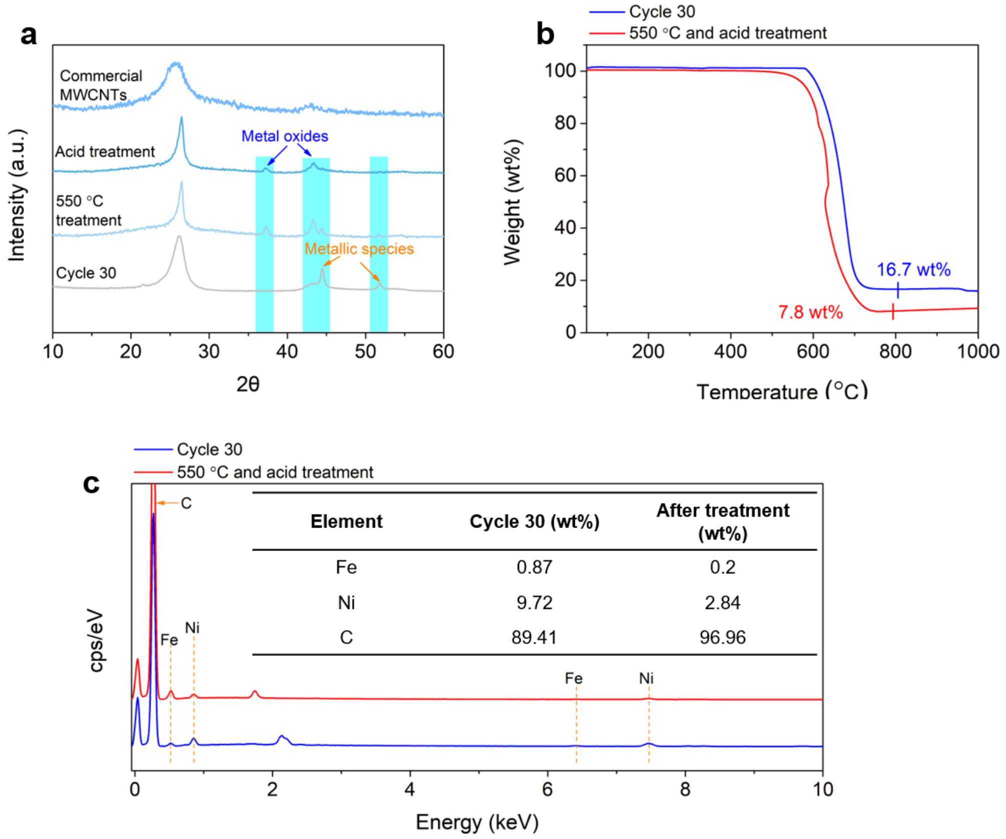

Fig. 54. (**a**) XRD patterns of the prepared CNTs (Cycle 30) before and after treatment, with the XRD pattern of industrial-grade multi-walled carbon nanotubes as a reference. (**b**) The metal content of the CNTs before and after treatment is approximately 16.7 wt% and 7.8 wt%, respectively. (**c**) EDX spectra and elemental composition of the prepared CNTs before and after treatment. The produced CNTs were treated in an air atmosphere at 550 °C for 0.5 h and then acid treated (~2.5 mol/L) at 50 °C for 2 h. CNTs production conditions: using FeNi/Ni/CNTs as the catalyst. Feedstock: LDPE and PVC mixture with a mass ratio of 9:1. 3 g plastic mixture and 3 g FeNi/Ni/CNTs catalyst in the bottom layer; 3 g FeNi/Ni/CNTs catalyst in the top layer.

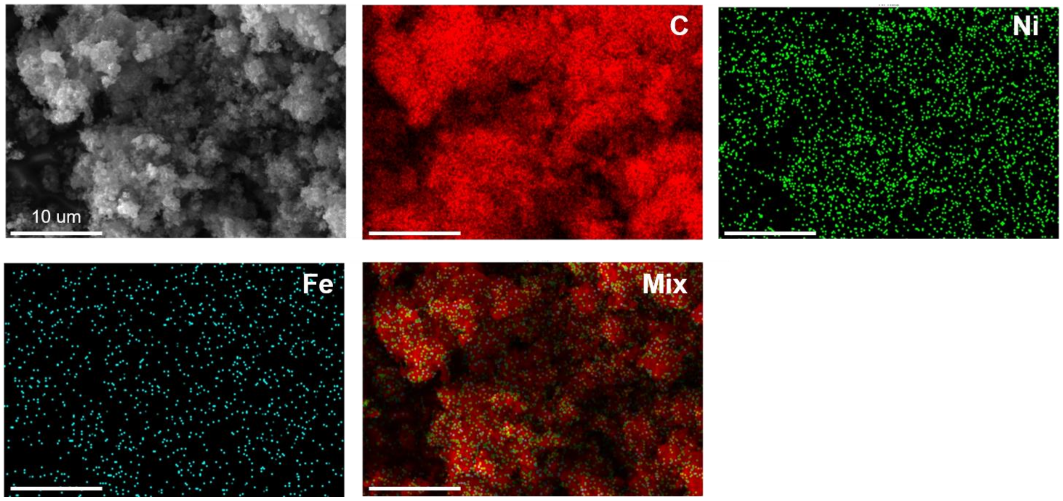

Fig. 55. EDX element mapping of the CNTs obtained after 30 successive cycles. The produced CNTs were treated in an air atmosphere at 550 °C for 0.5 h and then acid treated (~2.5 mol/L) at 50 °C for 2 h. CNTs production conditions: using FeNi/Ni/CNTs as the catalyst. Feedstock: LDPE and PVC mixture with a mass ratio of 9:1. 3 g plastic mixture and 3 g FeNi/Ni/CNTs catalyst in the bottom layer; 3 g FeNi/Ni/CNTs catalyst in the top layer.

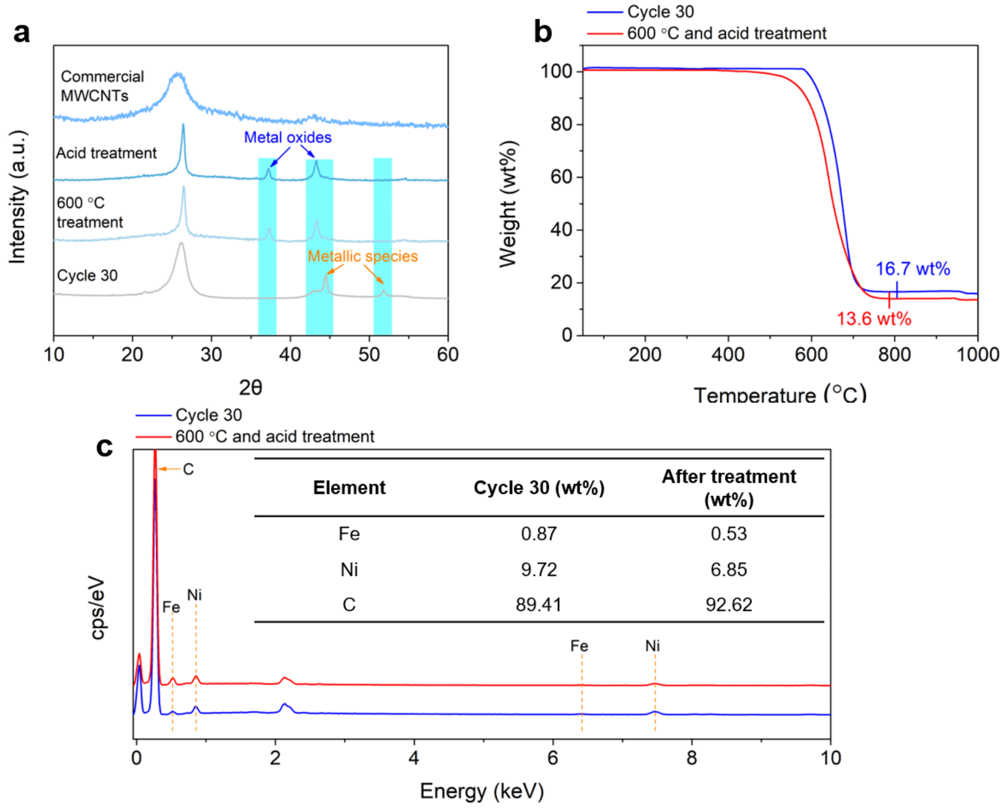

Fig. 56. (**a**) XRD patterns of the prepared CNTs (Cycle 30) before and after treatment, with the XRD pattern of industrial-grade multi-walled carbon nanotubes as a reference. (**b**) The metal content of the CNTs before and after treatment is approximately 16.7 wt% and 13.6 wt%, respectively. (**c**) EDX spectra and elemental composition of the prepared CNTs before and after treatment. The produced CNTs were treated in an air atmosphere at 600 °C for 0.5 h and then acid treated (~2.5 mol/L) at 50 °C for 2 h. CNTs production conditions: using FeNi/Ni/CNTs as the catalyst. Feedstock: LDPE and PVC mixture with a mass ratio of 9:1. 3 g plastic mixture and 3 g FeNi/Ni/CNTs catalyst in the bottom layer; 3 g FeNi/Ni/CNTs catalyst in the top layer.

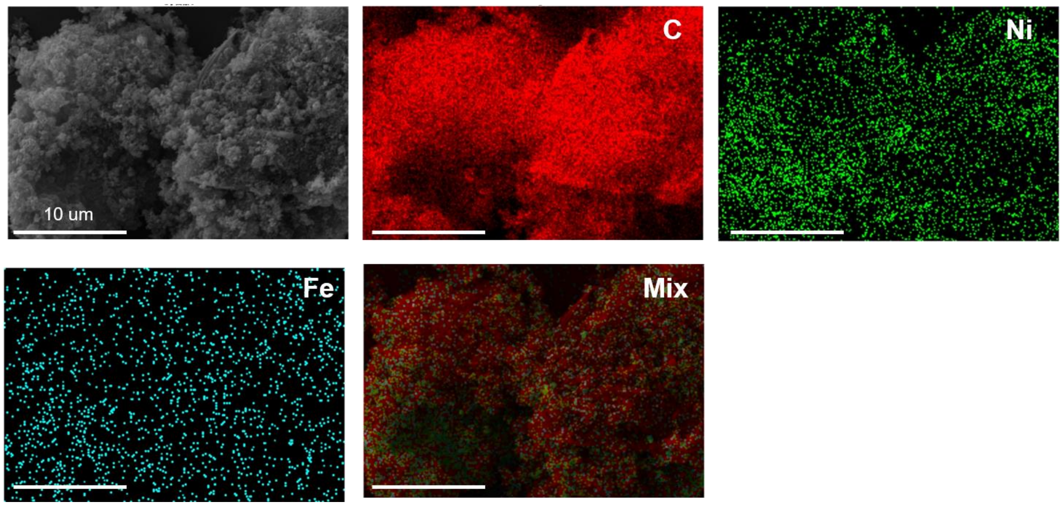

Fig. 57. EDX element mapping of the CNTs obtained after 30 successive cycles. The produced CNTs were treated in an air atmosphere at 600 °C for 0.5 h and then acid treated (~2.5 mol/L) at 50 °C for 2 h. CNTs production conditions: using FeNi/Ni/CNTs as the catalyst. Feedstock: LDPE and PVC mixture with a mass ratio of 9:1. 3 g plastic mixture and 3 g FeNi/Ni/CNTs catalyst in the bottom layer; 3 g FeNi/Ni/CNTs catalyst in the top layer.

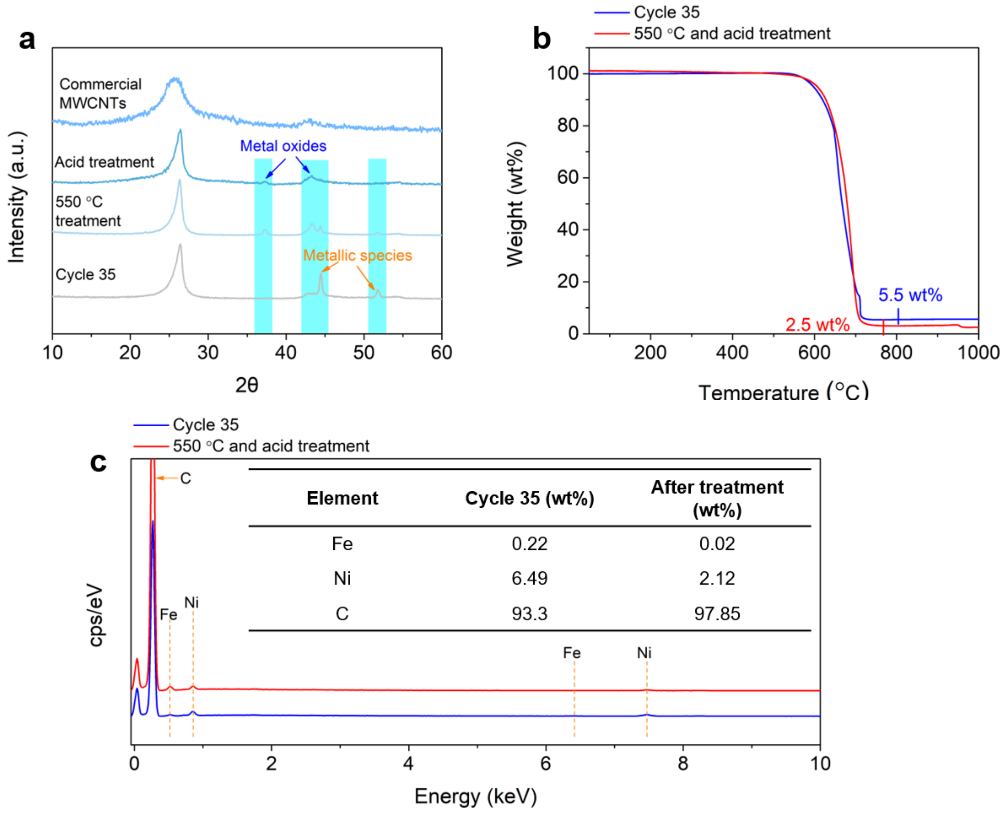

Fig. 58. (**a**) XRD patterns of the prepared CNTs (Cycle 35) before and after treatment, with the XRD pattern of industrial-grade multi-walled carbon nanotubes as a reference. (**b**) The metal content of the CNTs before and after treatment is approximately 5.5 wt% and 2.5 wt%, respectively. (**c**) EDX spectra and elemental composition of the prepared CNTs before and after treatment. The produced CNTs were treated in an air atmosphere at 550 °C for 0.5 h and then acid treated (~2.5 mol/L) at 50 °C for 2 h. CNTs production conditions: using FeNi/Ni/CNTs as the catalyst. Feedstock: LDPE (Cycle 1~Cycle 25, Cycle 31~Cycle 35), PVC (Cycle 26~Cycle 30). 3 g plastic and 3 g FeNi/Ni/CNTs catalyst in the bottom layer; 3 g FeNi/Ni/CNTs catalyst in the top layer.

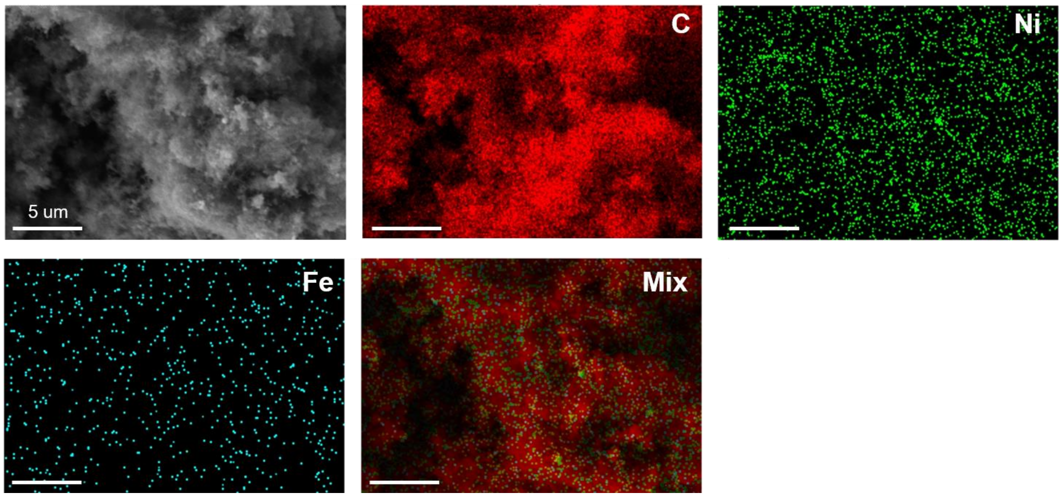

Fig. 59. EDX elemental mapping of the obtained CNTs after 35 successive cycles. CNTs production conditions: using FeNi/Ni/CNTs as the catalyst. Feedstock: LDPE (Cycle 1~Cycle 25, Cycle 31~Cycle 35), PVC (Cycle 26~Cycle 30). 3 g plastic and 3 g FeNi/Ni/CNTs catalyst in the bottom layer; 3 g FeNi/Ni/CNTs catalyst in the top layer.

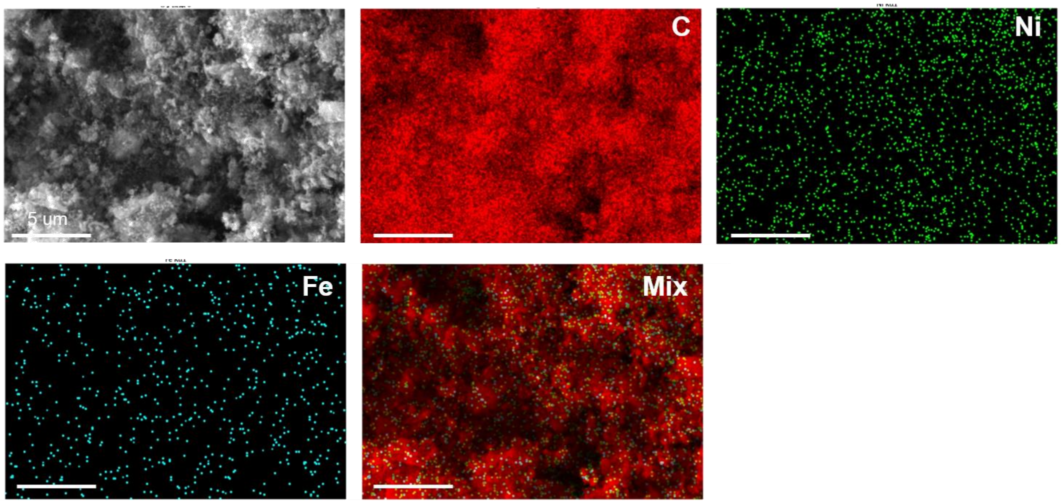

Fig. 60. EDX element mapping of the CNTs obtained after 35 successive cycles. The produced CNTs were treated in an air atmosphere at 550 °C for 0.5 h and then acid treated (~2.5 mol/L) at 50 °C for 2 h. CNTs production conditions: using FeNi/Ni/CNTs as the catalyst. Feedstock: LDPE (Cycle 1~Cycle 25, Cycle 31~Cycle 35), PVC (Cycle 26~Cycle 30). 3 g plastic and 3 g FeNi/Ni/CNTs catalyst in the bottom layer; 3 g FeNi/Ni/CNTs catalyst in the top layer.

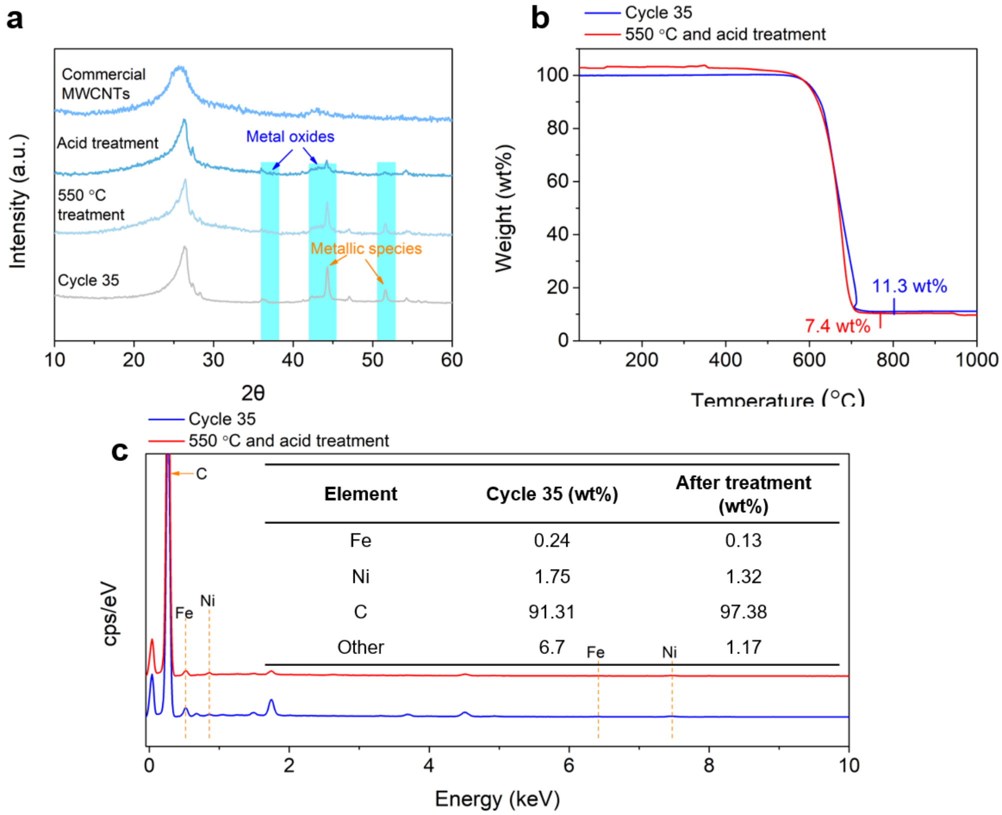

Fig. 61. (**a**) XRD patterns of the prepared CNTs (Cycle 35) before and after treatment, with the XRD pattern of industrial-grade multi-walled carbon nanotubes as a reference. (**b**) The metal content of the CNTs before and after treatment is approximately 11.3 wt% and 9.7 wt%, respectively. (**c**) EDX spectra and elemental composition of the prepared CNTs before and after treatment. The produced CNTs were treated in an air atmosphere at 550 °C for 0.5 h and then acid treated (~2.5 mol/L) at 50 °C for 2 h. CNTs production conditions: using FeNi/Ni/CNTs as the catalyst. Feedstock: PVC and landfilled plastic waste mixtures with a mass ratio of 1:9 (cycle 1 - cycle 30), PTFE and landfilled plastic waste mixtures with a mass ratio of 1:9 (cycle 31 - cycle 35). 3 g plastic mixtures and 3 g FeNi/Ni/CNTs catalyst in the bottom layer; 3 g FeNi/Ni/CNTs catalyst in the top layer.

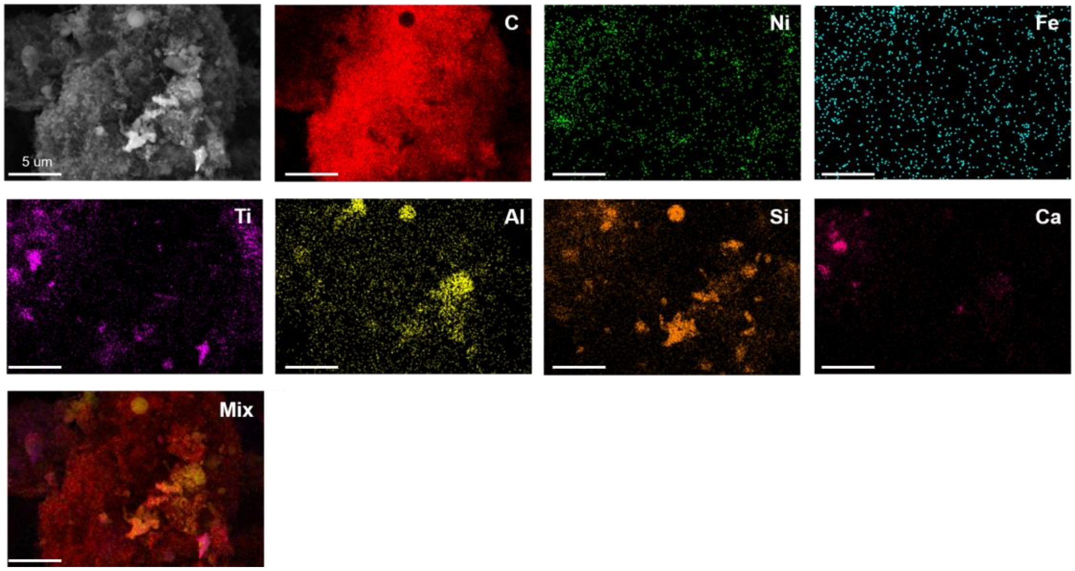

Fig. 62. EDX elemental mapping of the obtained CNTs after 35 successive cycles. CNTs production conditions: using FeNi/Ni/CNTs as the catalyst. Feedstock: PVC and landfilled plastic waste mixtures with a mass ratio of 1:9 (cycle 1 - cycle 30), PTFE and landfilled plastic waste mixtures with a mass ratio of 1:9 (cycle 31 - cycle 35). 3 g plastic mixtures and 3 g FeNi/Ni/CNTs catalyst in the bottom layer; 3 g FeNi/Ni/CNTs catalyst in the top layer.

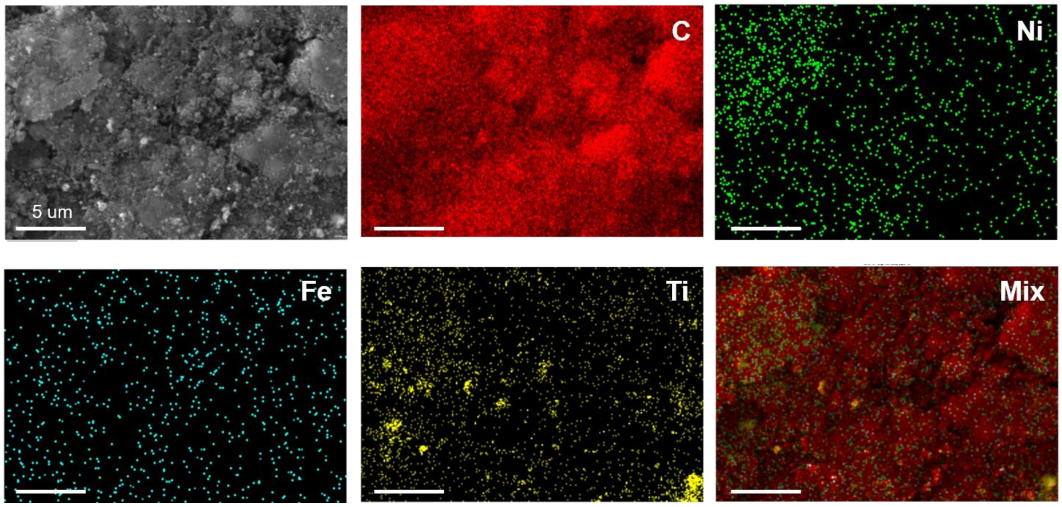

Fig. 63. EDX element mapping of the CNTs obtained after 35 successive cycles. The produced CNTs were treated in an air atmosphere at 550 °C for 0.5 h and then acid treated (~2.5 mol/L) at 50 °C for 2 h. CNTs production conditions: using FeNi/Ni/CNTs as the catalyst. Feedstock: PVC and landfilled plastic waste mixtures with a mass ratio of 1:9 (cycle 1 - cycle 30), PTFE and landfilled plastic waste mixtures with a mass ratio of 1:9 (cycle 31 - cycle 35). 3 g plastic mixtures and 3 g FeNi/Ni/CNTs catalyst in the bottom layer; 3 g FeNi/Ni/CNTs catalyst in the top layer.

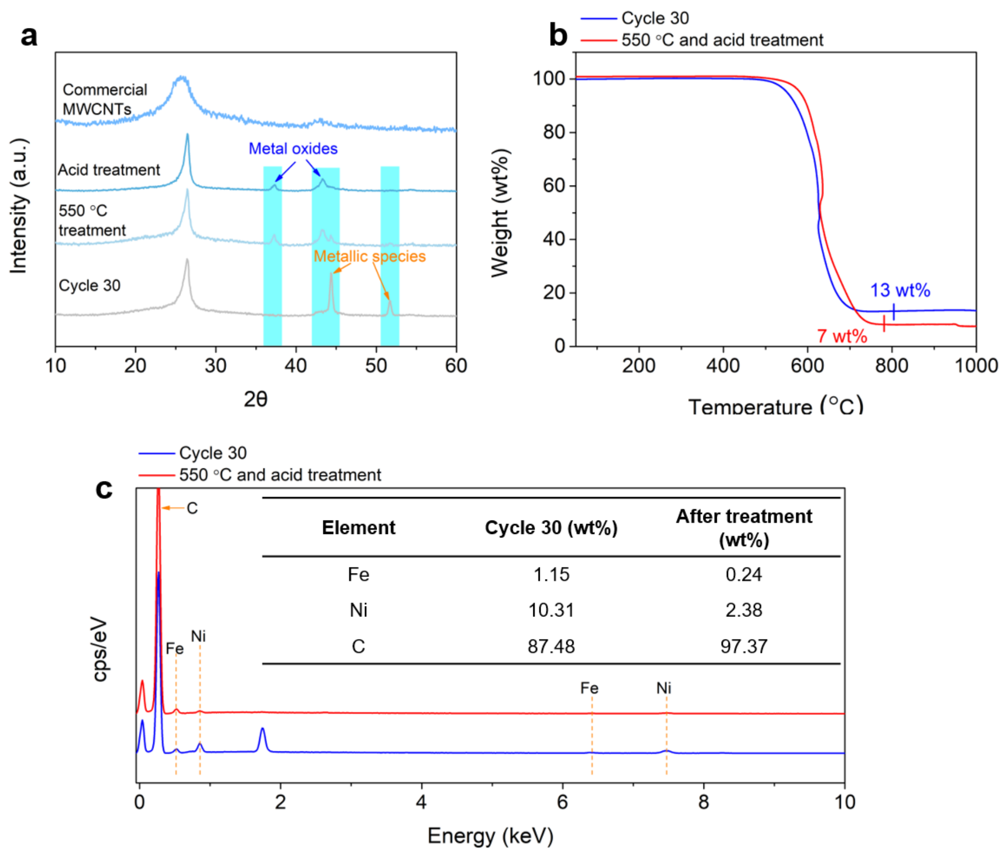

Fig. 64. (**a**) XRD patterns of the prepared CNTs (Cycle 30) before and after treatment, with the XRD pattern of industrial-grade multi-walled carbon nanotubes as a reference. (**b**) The metal content of the CNTs before and after treatment is approximately 13 wt% and 8 wt%, respectively. (**c**) EDX spectra and elemental composition of the prepared CNTs before and after treatment. The produced CNTs were treated in an air atmosphere at 550 °C for 0.5 h and then acid treated (~2.5 mol/L) at 50 °C for 2 h. CNTs production conditions: using FeNi/Ni/CNTs as the catalyst. Feedstock: plastic mixtures of LDPE, HDPE, PP, PS, PVC and PTFE with mass ratios of 15:7:2:5:7:5 (cycle 1 - cycle 5), 40:30:40:20:5:1 (cycle 6 - cycle 10), 2:6:4:6:4:1 (cycle 11 - cycle 15), 10:10:3:10:5:5 (cycle 16 - cycle 20), 5:10:3:15:10:3 (cycle 21 - cycle 25) and 5:2:15:5:5:1 (cycle 26 - cycle 30). 3 g LDPE, HDPE, PP, PS, PVC and PTFE plastic mixtures and 3 g FeNi/Ni/CNTs catalyst in the bottom layer; 3 g FeNi/Ni/CNTs catalyst in the top layer.

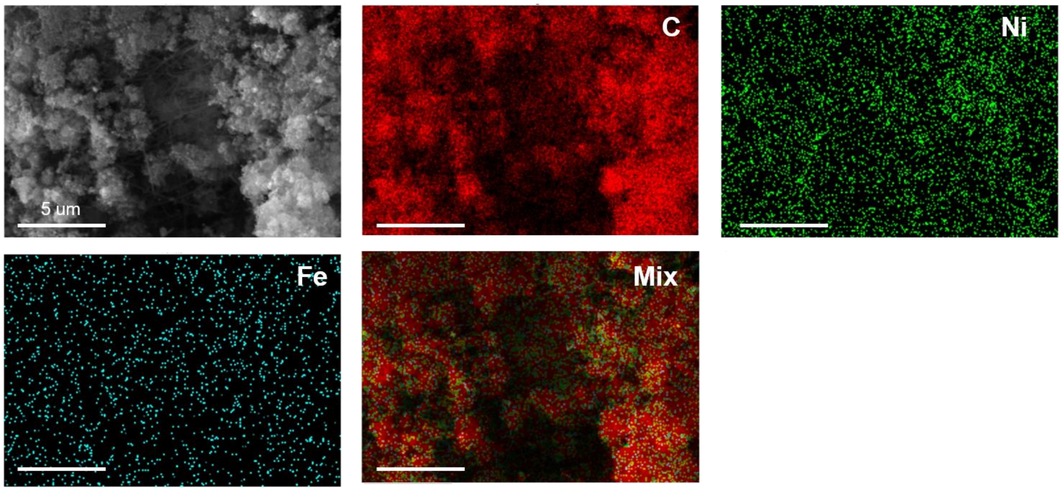

Fig. 65. EDX elemental mapping of the obtained CNTs after 30 successive cycles. CNTs production conditions: using FeNi/Ni/CNTs as the catalyst. Feedstock: plastic mixtures of LDPE, HDPE, PP, PS, PVC and PTFE with mass ratios of 15:7:2:5:7:5 (cycle 1 - cycle 5), 40:30:40:20:5:1 (cycle 6 - cycle 10), 2:6:4:6:4:1 (cycle 11 - cycle 15), 10:10:3:10:5:5 (cycle 16 - cycle 20), 5:10:3:15:10:3 (cycle 21 - cycle 25) and 5:2:15:5:5:1 (cycle 26 - cycle 30). 3 g LDPE, HDPE, PP, PS, PVC and PTFE plastic mixtures and 3 g FeNi/Ni/CNTs catalyst in the bottom layer; 3 g FeNi/Ni/CNTs catalyst in the top layer.

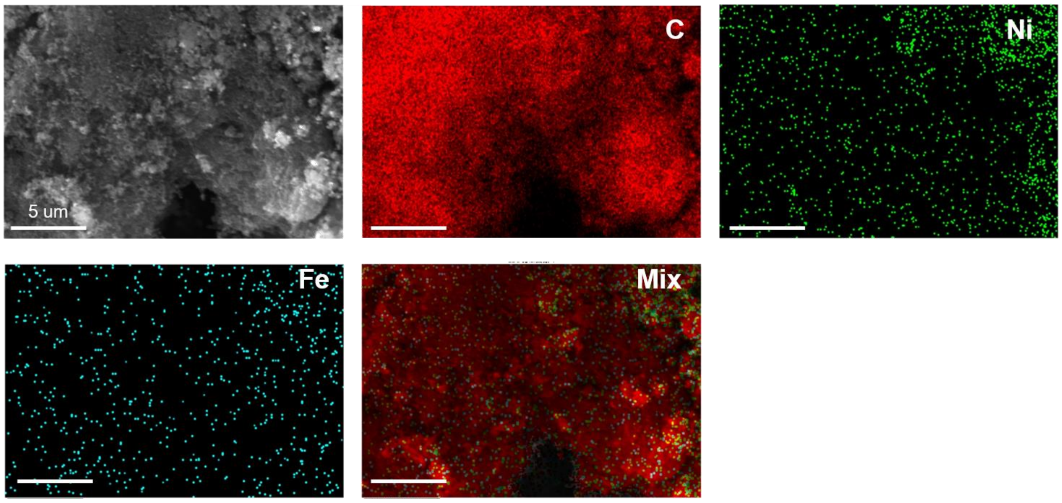

Fig. 66. EDX elemental mapping of the obtained CNTs after 30 successive cycles. The produced CNTs were treated in an air atmosphere at 550 °C for 0.5 h and then acid treated (~2.5 mol/L) at 50 °C for 2 h. CNTs production conditions: using FeNi/Ni/CNTs as the catalyst. Feedstock: plastic mixtures of LDPE, HDPE, PP, PS, PVC and PTFE with mass ratios of 15:7:2:5:7:5 (cycle 1 - cycle 5), 40:30:40:20:5:1 (cycle 6 - cycle 10), 2:6:4:6:4:1 (cycle 11 - cycle 15), 10:10:3:10:5:5 (cycle 16 - cycle 20), 5:10:3:15:10:3 (cycle 21 - cycle 25) and 5:2:15:5:5:1 (cycle 26 - cycle 30). 3 g LDPE, HDPE, PP, PS, PVC and PTFE

plastic mixtures and 3 g FeNi/Ni/CNTs catalyst in the bottom layer; 3 g FeNi/Ni/CNTs catalyst in the top layer.

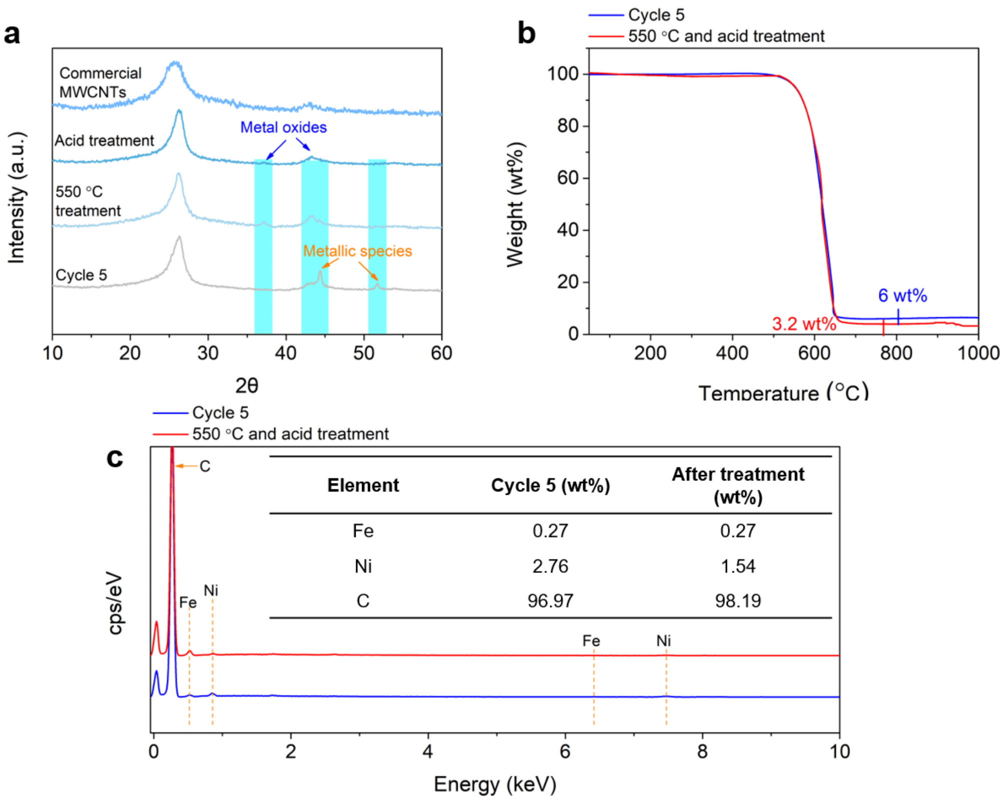

Fig. 67. (**a**) XRD patterns of the prepared CNTs (Cycle 5) before and after treatment, with the XRD pattern of industrial-grade multi-walled carbon nanotubes as a reference. (**b**) The metal content of the CNTs before and after treatment is approximately 6 wt% and 3.2 wt%, respectively. (**c**) EDX spectra and elemental composition of the prepared CNTs before and after treatment. The produced CNTs were treated in an air atmosphere at 550 °C for 0.5 h and then acid treated (~2.5 mol/L) at 50 °C for 2 h. CNTs production conditions: using FeNi/Ni/CNTs as the catalyst. Feedstock: LDPE, HDPE, PP, PS, PVC and PTFE plastic mixtures (875:615:965:330:510:12). 30 g LDPE, HDPE, PP, PS, PVC and PTFE plastic mixtures and 1.5 g FeNi/Ni/CNTs catalyst in the bottom layer; 1.5 g FeNi/Ni/CNTs catalyst in the top layer.

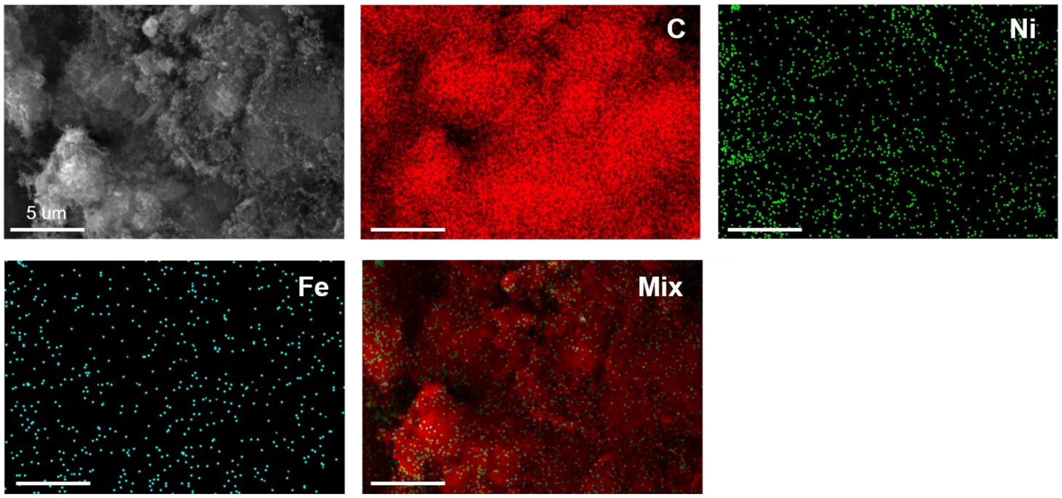

Fig. 68. EDX elemental mapping of the obtained CNTs after 5 successive cycles. CNTs production conditions: using FeNi/Ni/CNTs as the catalyst. Feedstock: LDPE, HDPE, PP, PS, PVC and PTFE plastic mixtures (875:615:965:330:510:12). 30 g LDPE, HDPE, PP, PS, PVC and PTFE plastic mixtures and 1.5 g FeNi/Ni/CNTs catalyst in the bottom layer; 1.5 g FeNi/Ni/CNTs catalyst in the top layer.

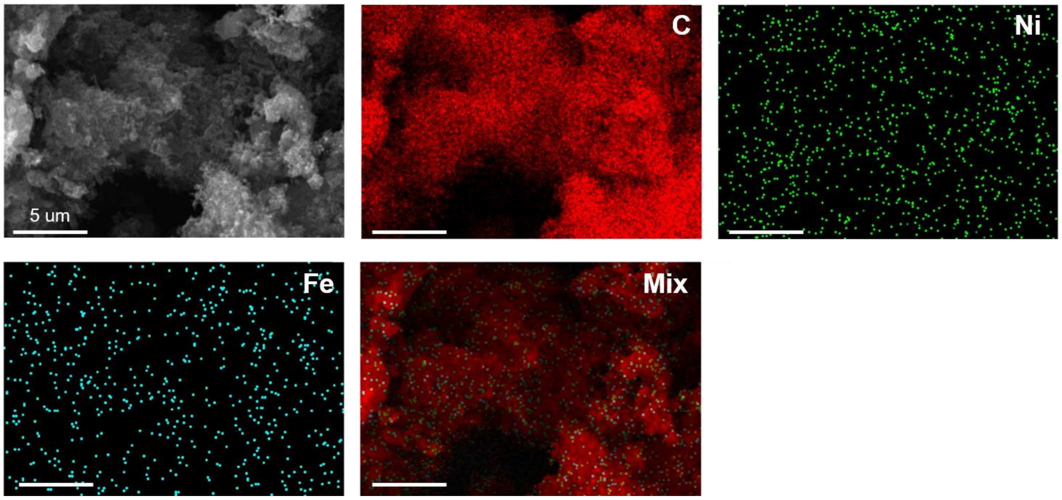

Fig. 69. EDX element mapping of the CNTs obtained 5 successive cycles. The produced CNTs were treated in an air atmosphere at 550 °C for 0.5 h and then acid treated (~2.5 mol/L) at 50 °C for 2 h. CNTs production conditions: using FeNi/Ni/CNTs as the catalyst. Feedstock: LDPE, HDPE, PP, PS, PVC and PTFE plastic mixtures (875:615:965:330:510:12). 30 g LDPE, HDPE, PP, PS, PVC and PTFE plastic mixtures and 1.5 g FeNi/Ni/CNTs catalyst in the bottom layer; 1.5 g FeNi/Ni/CNTs catalyst in the top layer.

Figs. R48-69 have been labelled as Supplementary Fig. 41 and Supplementary Figs. 43-63 in the supplementary material.

7. In general, the present work does not provide a deep insight into polymer degradation phenomena based on the basic science of polymers, as every polymer will degrade under elevated temperatures, high energy exposure, other radiation sources, or similar conditions. The current format of this article does not have the characteristics of a scientific article, but rather that of a technical report.

Response: We sincerely appreciate the reviewer's constructive feedback. The novelty and significance of this work are outlined below.

This research has developed an efficient in-situ constructed FeNi/Ni/CNTs catalyst for the catalytic decomposition of plastic waste mixture. Notably, the FeNi/Ni/CNTs catalyst enables rapid, efficient upcycling of chlorinated/fluorinated plastic waste mixtures into H_2 and CNTs under atmospheric pressure, alongside extended catalytic stability. Moreover, this approach significantly reduces energy consumption for plastic upcycling while achieving nearly 100% C and H atom utilization efficiency.

For catalytic performance: This approach achieves highly efficient, rapid upcycling of diverse real-world plastic waste mixtures into valuable H_2 and CNTs within minutes, thanks to the specially designed microwave catalytic system and high-performance FeNi/Ni/CNTs catalyst. In this in situ formed FeNi alloy, enhanced electron cloud density of Fe, Ni and electron transfer from Fe to Ni reduces the energy barrier of the reaction and improves catalytic activity. Notably, it attains nearly 100% atom economy, converting nearly all H and C atoms in plastics into H_2 and CNTs. Additionally, the catalyst exhibits an exceptionally high turnover number (107.6 $mol_{carbon}\ mol^{-1}_{NiFe}$), paired with a long catalytic lifetime of over 35 consecutive cycles, seven times longer than previous work (Nature Catalysis 3, 902–912 (2020)).

For feedstock adaptability: This approach shows strong tolerance to plastic waste. Even when applied to real-world chlorinated/fluorinated plastic waste mixtures (LDPE/HDPE/PP/PS/PVC/PTFE and landfilled plastics), it still achieves nearly 100% conversion of H and C atoms into H_2 and CNTs. Importantly, Cl^- and F^- gases released from PVC and PTFE during the process can be absorbed by the aqueous phase to form acid solutions. This feature allows CNT purification without commercial acids, reducing metal content in the resulting carbonaceous material and yielding purified CNTs. Ultimately, this well-designed microwave catalysis process efficiently upcycles real-world chlorinated/fluorinated plastic mixtures, eliminating over 90% of toxic Cl/F emissions while offering substantial commercial potential and environmental benefits. No similar

work has been reported as the majority of the related studies only focus on limited plastic waste sources.

For energy efficiency and viability: Importantly, this microwave catalysis consumes only ~1/5 the energy of traditional thermal catalysis under identical experimental conditions, thus representing an energy-efficient approach to plastic upcycling. Compared with other benchmark studies, this strategy demonstrates enhanced commercial competitiveness and robust market viability: the product's economic value is 2-28 times higher (achieved value: \$9568/ton$_{plastic}$ vs. \$1000-5200/ton$_{plastic}$), reaction time is reduced by 25-138 times (0.16 h vs. 4-22 h), and no exogenous hydrogen co-reactant is required (1 bar N_2 vs. 1-30 bar H_2) (Nature Chemical Engineering 1, 376-384 (2024); Nature Sustainability 6, 1258-1267 (2023)).

Fundamental understanding of both in-situ catalyst synthesis and plastic decomposition. The catalyst was in-situ formed via PVC-assisted chlorination and reduction by hydrogen gas generated from plastic decomposition, as proved by XRD, XAS, TEM, and H_2-TPR-MS. Different from the conventional heating mode, it is impossible to in-situ monitor plastic decomposition under microwave irradiation due to the limitations of the current technology available as no analytic tools can maintain sensitive and reliable measurement under microwave irradiation. So the plastic decomposition mechanism has extensively investigated indirectly by XAS, TG-MS, GC, SEM, TGA, XRD, TEM, comprehensive experimental studies, and DFT calculations. It demonstrates that Ni species contribute to C-H breaking in the plastic decomposition, and Fe is beneficial for carbon tube formation and growth during the microwave catalysis, which helps to elucidate the reaction mechanism.

Overall, this work demonstrates a new strategy to address key challenges in upcycling complex plastic mixtures, including harsh reaction conditions, catalyst instability, a narrow range of treatable plastics, and high energy consumption in recycling, together with the very likely reaction mechanism. We hope these 25 new case studies fully address the reviewer concerns and remarkably improve the scientific rigor of this work.

We extend our sincere gratitude to the three reviewers again for their invaluable comments. In response to this constructive feedback, we have conducted comprehensive studies and engaged in thorough discussions to address their concerns. We think that these efforts would significantly enhance the quality and scientific rigor of our work. All modifications are highlighted with a yellow background in both the main text and supplementary information.

Reviewer#1: most previous raised problems have been addressed properly, except for the calculating of energy efficiency. The explanation is not convincible. The absorbed energy should be calculated from the aspect of sample instead of microwave, i.e. how much energy was used to heat up the sample and maintain the reaction, including latent heat, sensible heat and reaction heat. So maybe author should reconsider the energy calculation formula.

Response: We appreciate the reviewer for pointing out this issue. We tried our best to calculate the energy efficiency while accurately measuring microwave energy utilization efficiency including latent heat, inductive heat, and reaction heat remains challenging due to the change of the microwave absorber during the reaction process, which has been proved below. We thus designed a process to simulate the energy absorbed by the sample, which involves heating the sample in a microwave field for a fixed duration, followed by rapid transfer to room-temperature water. By measuring the temperature rise of the water, the energy absorbed by the sample was calculated using the formula $Ea=m\cdot c_p\cdot \Delta T$ *(m, cp, and ΔT are the mass of water, the heat capacity of water, and the temperature difference before and after heating water, respectively)*. This value and reaction heat were subsequently compared with the total microwave input energy ($Ei=P\cdot t$, *P and t are microwave power and heating time, respectively*) to assess microwave energy utilization efficiency. Note: This procedure involves inherent risks.

Given LDPE's simple molecular structure and the relative ease of calculating its reaction enthalpy, catalytic decomposition experiments were conducted using a 99:1 mass ratio of LDPE-PVC plastic mixtures as feedstock. For Cycle 1, 3 g of the plastic mixture was combined with 3 g of FeNi/Ni/C catalyst and loaded into the bottom reactor layer, with an additional 3 g of FeNi/Ni/C catalyst placed in the upper layer; following 5 minutes of microwave irradiation at 600 W, the reactor was immediately immersed in room-temperature water, and once thermal equilibrium was reached, the water temperature was measured to calculate the temperature change relative to the initial value. For Cycle 15, 3 g of the plastic mixture was mixed with the used bottom layer catalyst from previous cycles and loaded into the bottom reactor chamber, with upper layer catalyst retained in the upper layer, and the same irradiation and temperature measurement protocol as Cycle 1 was followed. Experimental results indicate that the energy absorbed by the water is 24,660 J and 86,320 J for Cycle 1 and Cycle 15, respectively, while the total microwave input energy for each experiment is 180,000 J.

For the reaction heat of LDPE plastic decomposition, it is calculated by Benson's group additivity method (Journal of Physical and Chemical Reference Data, 1996, 25: 1411-1481. Nature Chemical Engineering 2, 650–661 (2025).).

$$(CH_2)_n \longrightarrow nH_2 + nC$$

$$\Delta_r H = n\Delta_r H_{H_2} + n\Delta_r H_C - n\Delta_r H_{CH_2}$$

$$n = \frac{m_{PE}}{M_{CH_2}}$$

Wherein, $\Delta_r H$, $n\Delta_r H_{H_2}$, $n\Delta_r H_C$, and $n\Delta_r H_{CH_2}$ correspond to the reaction enthalpy, enthalpy of hydrogen formation, enthalpy of carbon formation, and enthalpy of polyethylene chain segment formation, respectively; m_{PE} and M_{CH_2} denote the mass of polyethylene plastic and the molar mass of the polyethylene repeating unit, respectively.

The heat of reaction for plastic decomposition calculated via the aforementioned process is 4395 J (**Table R1**). Accordingly, the microwave energy absorbed by the samples should be 29055 J (24660 J + 4395 J, Cycle 1) and 90715 J (86320 J + 4395 J, Cycle 15), respectively, as the energy consumed for plastic decomposition is transferred from the catalyst materials (Nature Catalysis 3, 902-912 (2020)). Based on this, the microwave energy utilization efficiencies are calculated to be 16.1% (100% × 29055 / 180000, Cycle 1) and 50.4% (100% × 90715 / 180000, Cycle 15), respectively. The enhanced microwave energy utilization efficiency after cycle 15 compared to that after cycle 1 is mainly attributed to the large quantity of CNTs generated during catalytic plastic decomposition, which is an excellent microwave-absorbing material, thus improving microwave absorption efficiency. On the other hand, the failure to achieve nearly full microwave energy utilization is due to the large volume of the reaction chamber and the insufficient dosage of microwave-absorbing materials, which results in incomplete absorption of microwave energy.

Table R1 Enthalpy values of different substances

	$\Delta H^{o}_{f\,298}$ (kJ/mol)
H_2	0
C	0
CH_2	-20.72

The above measurement and discussion were added to pages 13-14 in the Supplementary information.

Reviewer #2: General Comments: While the authors have addressed our previous comments to some extent, several points remain insufficiently resolved. Accordingly, substantial revisions are required before the manuscript can be considered for publication in Nature Communications.

1. Direct evidence demonstrates that the metal oxide surface is re-exposed due to the role of fluorine is required. The current explanation, which attributes this effect solely to CNT shearing, remains speculative and insufficiently supported. Given that a major strength of this work lies in the effective recycling of chlorinated and fluorinated plastics, a more rigorous and explicit discussion of this mechanism is necessary. In addition, when PTFE is used as the feedstock, the authors should clearly assign and explain each XRD peak observed for the FeC–FeNi/Ni/CNT catalyst, including what each peak represents.

Response: We thank the reviewer for raising this very insightful comment. XRD analysis of the catalyst recovered after catalytic decomposition of PTFE over the FeC-FeNi/Ni/C precursor reveals a primary composition of Ni and NiF_2, as shown in **Fig. R1a**. Peaks at 2θ values of 44.5°, 51.8°, and 76.3° are assigned to metallic Ni, while those at 2θ = 27.1°, 34.8°, 38.6°, 40°, 43.4°, 53°, 55.8°, 59.9°, 63.1°, 66.9°, 67.5°, 70.9°, and 73.6° correspond to NiF_2. However, no iron fluoride compounds are detected in post-reaction catalysts. When Fe_3C was employed as a catalyst for PTFE decomposition at 600 W for 10 minutes, XRD analysis confirmed the catalyst retained its Fe_3C structure, with no iron fluoride formation observed (**Fig. R1b**). Therefore, fluorine in PTFE primarily reacts with nickel.

To further investigate whether PTFE can induce the transformation of FeC-FeNi/Ni/C catalyst precursors into FeNi/Ni/C, analogous to PVC's effect, five consecutive catalytic cycles were performed using FeC-FeNi/Ni/C precursors with an LDPE-PTFE mixture (9:1) as feedstock. Post-reaction XRD analysis suggests PTFE appears to promote FeNi alloy formation similar to PVC (**Fig. R1c**). Taken together, these results indicate fluorine facilitates FeNi alloy formation via the following pathway: during reaction, fluorine from PTFE combines with nickel to form NiF_2, which is then reduced by hydrogen generated from LDPE decomposition, combining with iron to form FeNi alloys. Concurrently, H_2-TPR-MS analysis of the material obtained post-PTFE reaction detects HF formation, which aligns with the aforementioned defluorination process of NiF_2 under a hydrogen atmosphere (**Fig. R1d)**. Complemented by TEM observations (**Fig. R2**), fluorine species may enhance catalyst long-term stability through two mechanisms: reconstructing FeNi active sites and shearing CNTs to expose additional catalytic sites.

To verify the functionality of PVC, which could be similar to that of PTFE, Ni/C and Fe_3C catalysts were synthesized via microwave-assisted methods, then mixed with PVC at a 1:1 mass ratio (5 g each) and reacted under 600 W microwave irradiation for 10 minutes. Next, 30 mL of deionized water was added, and the mixture was stirred at 25 °C for 2 hours. The resulting suspension was filtered, and the filtrate was dried completely in a 100 °C oven, yielding green nickel chloride crystals and yellow ferric chloride crystals, respectively (**Fig. R3a-b, insert**). Subsequent XRD characterization confirms the identity of the green crystals as nickel chloride and yellow crystals as ferric chloride, verifying the formation of these chloride compounds (**Figs. R4-5**). Subsequently, the formed nickel chloride and iron chloride are reduced by hydrogen, thereby facilitating the formation of FeNi alloy (**Fig. 6**). It is also true that catalyst precursors can be formed again during the reaction, which is then changed to metal chlorides/metal fluorides (**Fig. 7**). Following a similar reduction step, the FeNi alloy can be regenerated.

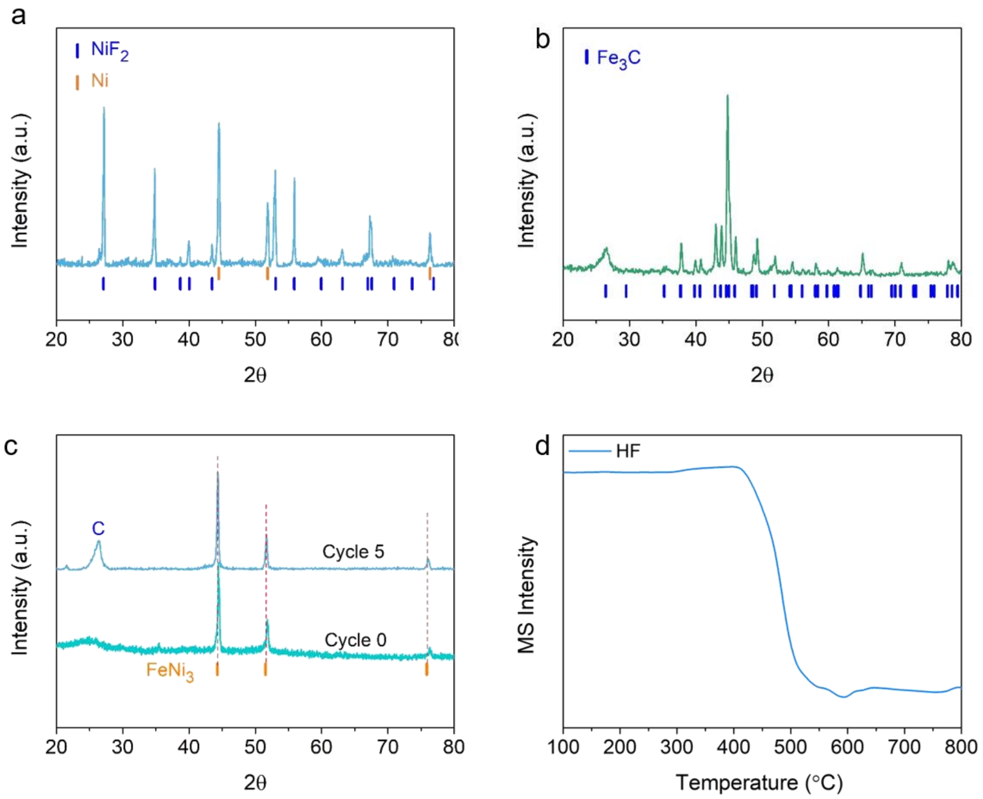

Fig. R1. (a) XRD pattern of the sample obtained after catalytic decomposition of PTFE over the FeC-FeNi/Ni/C precursor. (b) XRD pattern of the sample obtained after catalytic decomposition of PTFE over the Fe_3C. (c) XRD patterns of the sample obtained after different cycles of catalytic decomposition of PTFE over the FeC-FeNi/Ni/C precursor. (d) H_2-TPR-MS characterization of the sample obtained after catalytic decomposition of PTFE over the FeC-FeNi/Ni/C precursor.

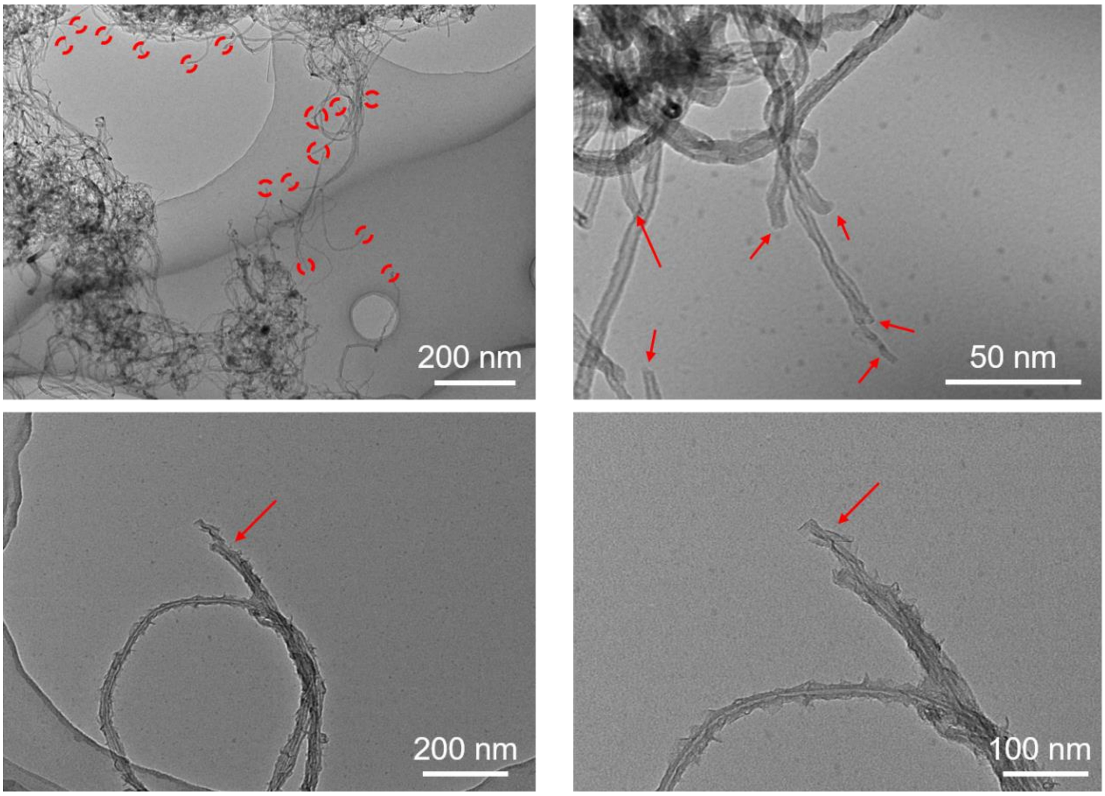

Fig. R2. TEM images of the CNTs after reaction with PTFE show that the CNTs have been cut off.

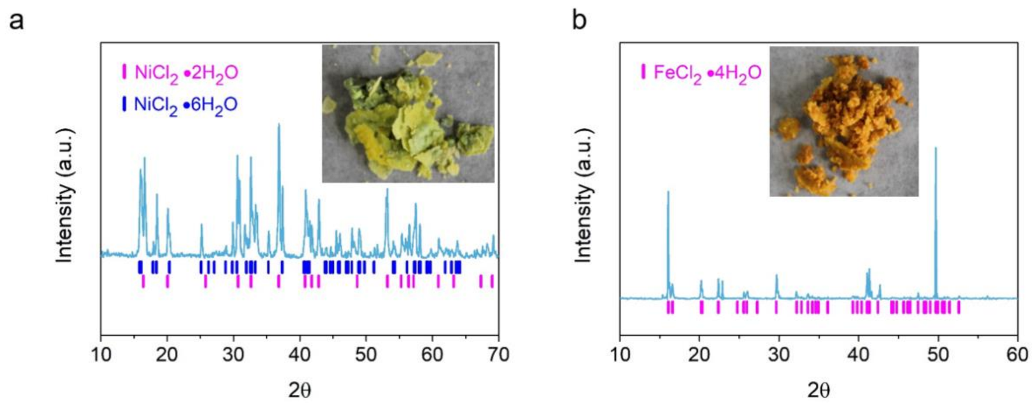

Fig. R3. Optical images and corresponding XRD patterns of the green (a) and yellow (b) crystals.

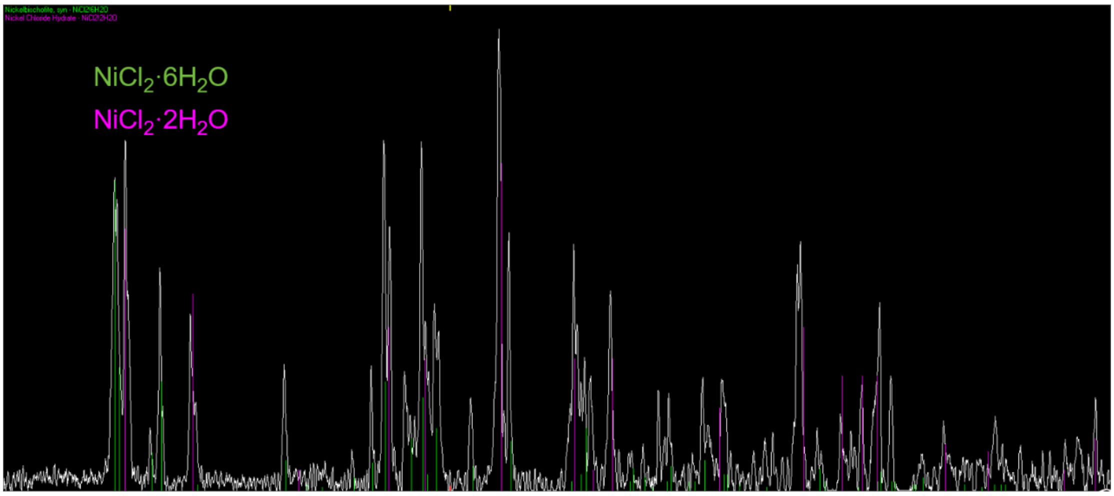

Fig. R4. XRD fitting results for the green crystals are consistent with $NiCl_2·6H_2O$ and $NiCl_2·2H_2O$.

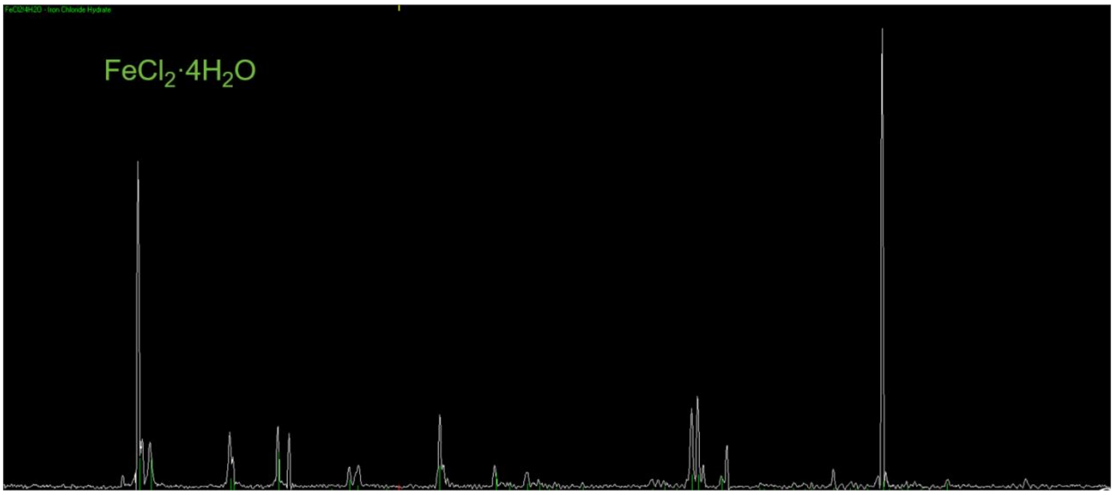

Fig. R5. XRD fitting results for the yellow crystals are consistent with $FeCl_2·4H_2O$.

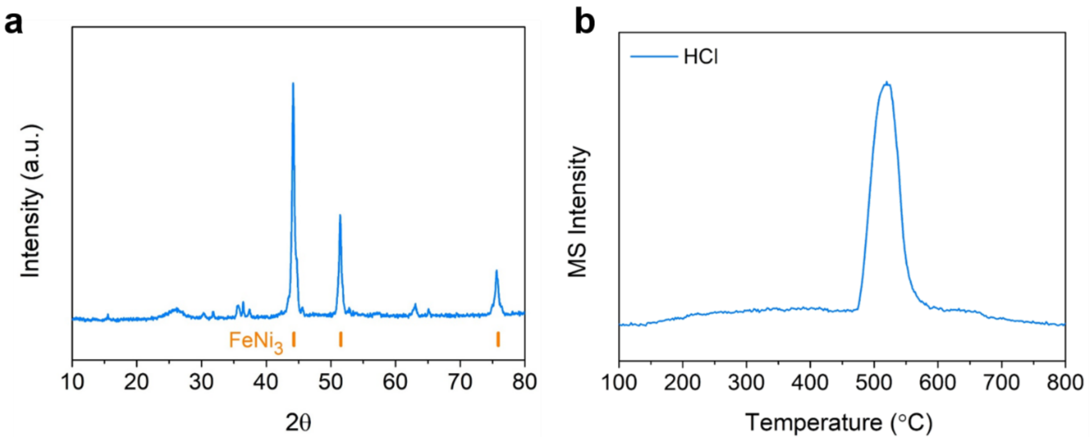

Fig. R6. (a) XRD results of the crystals after being treated with H_2. (b) The H_2-TPR-MS results of the crystals.

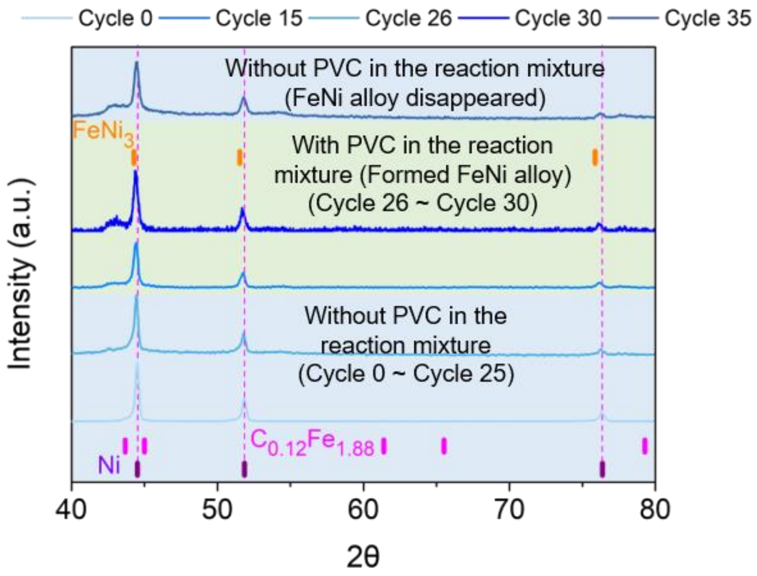

Fig. R7. XRD analysis of the catalyst structure in the reaction mixture with and without PVC.

Fig. R1, Fig. R2, Fig. R3, Fig.R6 and Fig. R7 have been marked as Supplementary Fig. 37, Supplementary Fig. 38, Supplementary Fig. 29, Supplementary Fig. 29 and Supplementary Fig. 32 in the supplementary material.

2. The magnified XANES region (8340–8341 eV) shown in the inset does not correspond to the edge onset or the edge energy (E_0) of the Ni K-edge but rather lies outside the edge onset region (Fig. R17b). As a result, the relative spectral positions in this range cannot be reliably used to discuss oxidation state differences. In particular, the fact that the Ni foil does not appear at the lowest energy may lead to confusion. In addition, the authors attribute the observed behavior to electron transfer from Fe to Ni induced by the formation of NiFe species. However, based on the XANES results, both Fe and Ni appear to progressively approach a more metallic state. Such a trend does not support the presence of directional electron transfer between Fe and Ni. If electron transfer were occurring, one would expect Fe to become more electron-deficient while Ni becomes more electron-rich, a trend that is not observed in the present analysis. Therefore, the observed changes in the electronic states are more likely attributable to factors other than intermetallic electron transfer between Fe and Ni. This point requires clearer clarification and more rigorous discussion.

Response: We appreciate the reviewer for raising this important point. In the previous manuscript, we employed X-ray absorption near-edge structure (XANES) spectroscopy to investigate the oxidation state of nickel in both the catalyst precursor and regenerated catalyst, focusing on the 8340-8341 eV energy region. In this revised version, we have changed E_0 in accordance with the reviewer's suggestion as the accurate edge position for analysis. The zoomed region in the XANES spectrum has been aligned with the onset of the K-edge, as illustrated in the revised **Fig. R8**. Additionally, we conducted an in-depth analysis and literature review regarding electron transfer between Fe and Ni. XANES characterization reveals that both Fe and Ni species in the regenerated catalyst exhibit metallic character. However, detailed analysis of the Fe K-edge energy (E_0) shows a slight positive shift from 7112.8 eV (Fe foil reference) to 7113.1 eV in the regenerated catalyst, suggesting minor electron loss and a slightly higher oxidation state relative to metallic iron.

Similarly, analysis of the Ni K-edge energy reveals a minor negative shift from 8332.6 eV (Ni foil reference) to 8332.4 eV in the regenerated catalyst, implying a small gain in electrons and a slightly lower oxidation state compared with metallic nickel. These observations support electron transfer from Fe to Ni species, which aligns with the Fe-to-Ni electron transfer behavior reported by Shi et al. for FeNi alloy catalysts (Energy Environ. Sci., 2025, 18, 10205-10215).

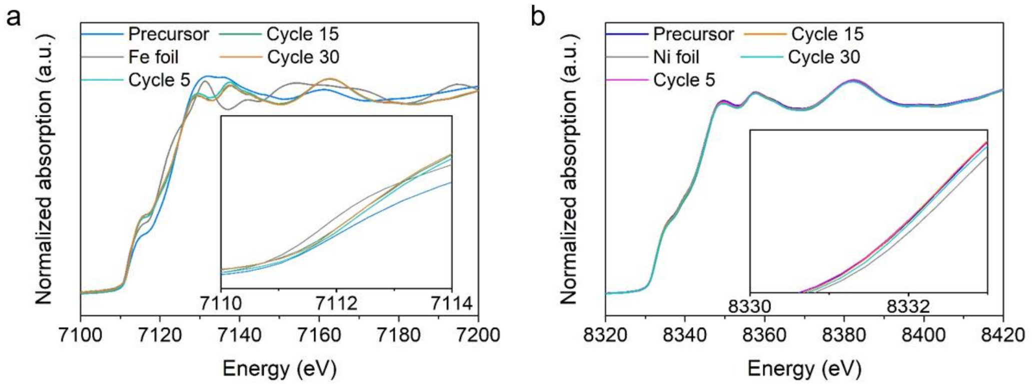

Fig. R8. XAS analysis. (a) Fe K-edge XANES spectra of FeC-FeNi/Ni/C (precursor) and FeNi/Ni/C (regenerated) catalysts as well as references. (b) Ni K-edge XANES spectra of FeC-FeNi/Ni/C and FeNi/Ni/C catalysts as well as references.

3. The EXAFS fitting results exhibit large uncertainties in the Debye–Waller factors (σ^2). For example, the reported value ($\sigma^2 = 0.093 \times 10^{-2}$ Å^2) has an uncertainty ($\pm 0.29 \times 10^{-2}$ Å^2) that significantly exceeds the fitted value itself, indicating that this parameter is not statistically well constrained and cannot be considered quantitatively meaningful. The authors are therefore requested to report the uncertainties for all other fitted parameters as well, to properly assess the robustness and reliability of the EXAFS analysis.

Response: We sincerely thank the reviewer for the critical comments and apologize for the suboptimal fitting of certain XAS data in the previous version. We have now re-fit the data with greater precision to ensure all results fall within acceptable bounds, as summarized in **Table R2**.

Table R2. Fitting results of the precursor and regenerated catalyst.

**Names**	**Shell**	**CN**	**R (Å)**	**σ ^2 (10^{-2} Å^2)**	**ΔE$_0$ (eV)**	**r-factor**
Precursor (Fe)	Fe-C<br>Fe-Ni	3.1(±0)<br>6.7(±0.01)	2.0(±0)<br>2.48(±0)	0.006(set)	-3(±0)	0.015
Precursor (Ni)	Ni-Fe<br>Ni-Ni	6.5(±0.4)<br>8.7(±0.3)	2.49(±0.004)<br>2.5(±0.002)	0.39(±0.057)<br>0.6(±0.03)	7.3(±0.7)	0.006
Cycle 5 (Fe)	Fe-Ni	8.5(±0.3)	2.49(±0.002)	0.6(±0.037)	7.8(±0.44)	0.002
Cycle 5 (Ni)	Ni-Fe<br>Ni-Ni	6.8(±0.4)<br>9.1(±0.3)	2.5(±0.004)<br>2.49(±0.002)	0.4(±0.05)<br>0.63(±0.03)	6.1(±0.4)	0.0018
Cycle 15 (Fe)	Fe-Ni	8.2(±0.4)	2.49(±0.003)	0.6(±0.04)	6.98(±0.57)	0.003
Cycle 15	Ni-Fe	6.6(±0.4)	2.5(±0.004)	0.4(±0.056)	6.8(±0.7)	0.006

(Ni)	Ni-Ni	8.9(±0.3)	2.51(±0.003)	0.6(±0.03)		
Cycle 30 (Fe)	Fe-Ni	8.7(±0.4)	2.49(±0.003)	0.65(±0.045)	8.1(±0.54)	0.003
Cycle 30 (Ni)	Ni-Fe	6.8±0.4	2.5(±0.004)	0.4(±0.055)	6.8(±0.7)	0.006
	Ni-Ni	9.0±0.3	2.51(±0.002)	0.63(±0.03)		

CN: coordination number; R: distance between absorber and backscatter atoms; σ^2: Debye-Waller factor to account for both thermal and structural disorders; ΔE_0: Inner potential correction; r factor (%) indicates the goodness of the fit. Fitting conditions: k around 3.0-12.0. R around 1.0-3.0. Rbkg=1. kw=1/2/3

Table R2 has been labelled as Supplementary Table 8 in the supplementary material.

4. In the TGA data shown in Fig. R21, no distinct weight loss is observed for cycles 15 and 30. Based on the described reaction setup, the catalyst and feedstock are mixed during the reaction, and the conversion is evaluated by analyzing the compositional changes of the resulting mixture. This implies that unreacted plastic is recovered together with the catalyst after the reaction. Consequently, the post-reaction plastic mixture should contain catalyst components, including CNTs, for which a measurable weight loss would normally be expected during TGA analysis. The absence of any observable weight loss under these conditions is therefore unexpected and requires clarification.
Response: We appreciate the reviewer for raising this point and apologize for the unclear description of the experimental conditions. No weight loss is observed in the TGA as it was conducted under a nitrogen atmosphere. Weight loss typically occurs when materials contain thermally labile, easily decomposable components, for example, when analyzing LDPE-PVC mixtures via TGA. The feedstock consists of CNTs and catalysts, with no readily decomposable plastics present, so CNTs are stable under nitrogen (**Fig. R9**). We further carried out the analysis under an oxygen atmosphere, which clearly results in weight loss, **Fig. R10**. These figures were added to Supplementary Fig. 7b and Supplementary Fig. 43 in the supplementary materials.

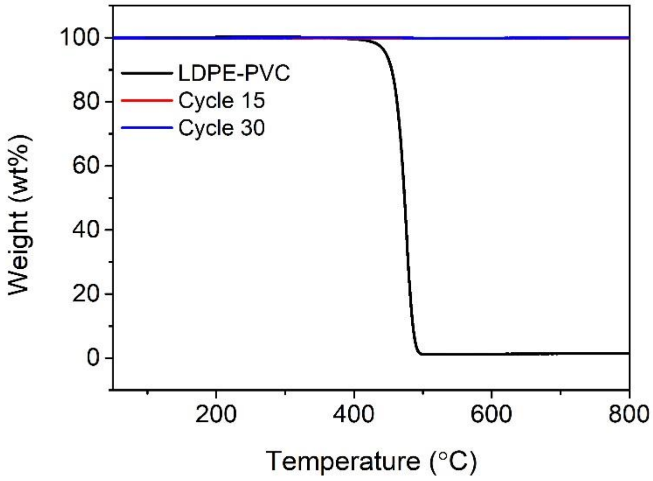

Fig. R9. Thermogravimetric analysis results of the LDPE-PVC plastic mixture and the used catalyst after 15 and 30 catalytic cycles under a nitrogen atmosphere.

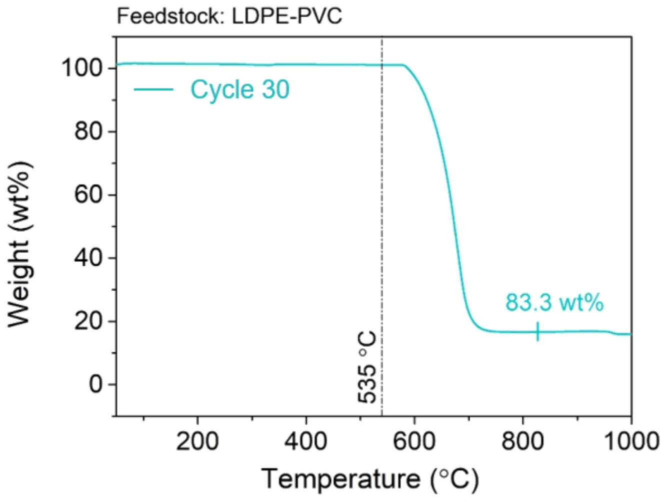

Fig. R10. Thermogravimetric analysis of the spent catalyst after 30 successive catalytic cycles under an oxygen atmosphere.

5. In Fig. R30, the formation of metal chlorides is supported by color changes and XRD analysis. However, additional evidence would be beneficial to more convincingly demonstrate the formation of metal chlorides. In particular, emphasizing the yellow color alone primarily suggests the formation of Fe-based chlorides, and does not provide sufficient evidence for the presence of $NiCl_2$, which is typically associated with a greenish color. Therefore, color observation should be regarded only as a supplementary indicator for chloride formation. Moreover, the XRD patterns appear to contain multiple overlapping peaks, making it difficult to unambiguously identify the formation of individual chloride species. In this regard, XAFS analysis of the yellow crystalline phase could provide more definitive evidence for the formation of $FeCl_2$, $FeCl_3$, and $NiCl_2$, and is suggested to strengthen the conclusions.

Response: We honestly thank the reviewer for proposing this point. As the XAS characterization instrument is in high demand, rapid result acquisition is challenging. Therefore, to further clarify the formation of ferric chloride and nickel chloride compounds, Ni/C and Fe_3C catalysts were synthesized via microwave-assisted methods, then mixed with PVC at a 1:1 mass ratio (5 g each) and reacted under 600 W microwave irradiation for 10 minutes. Next, 30 mL of deionized water was added, and the mixture was stirred at 25 °C for 2 hours. The resulting suspension was filtered, and the filtrate was dried completely in a 100 °C oven, yielding green nickel chloride crystals and yellow ferric chloride crystals, respectively (**Fig. R11a-b, insert**). Subsequent XRD characterization confirms the identity of the green crystals as nickel chloride and yellow crystals as ferric chloride, verifying the formation of these chloride compounds (**Figs. R12-13**).

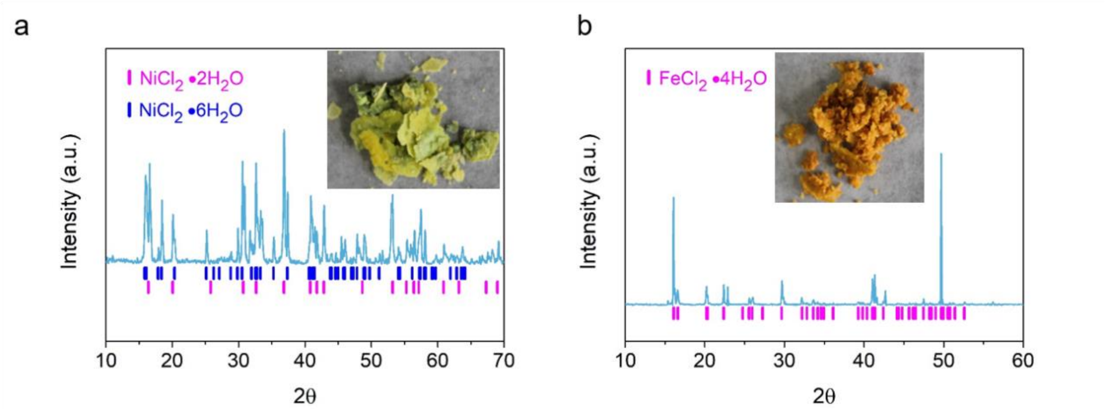

Fig. R11. Optical images and corresponding XRD patterns of the green (a) and yellow (b) crystals.

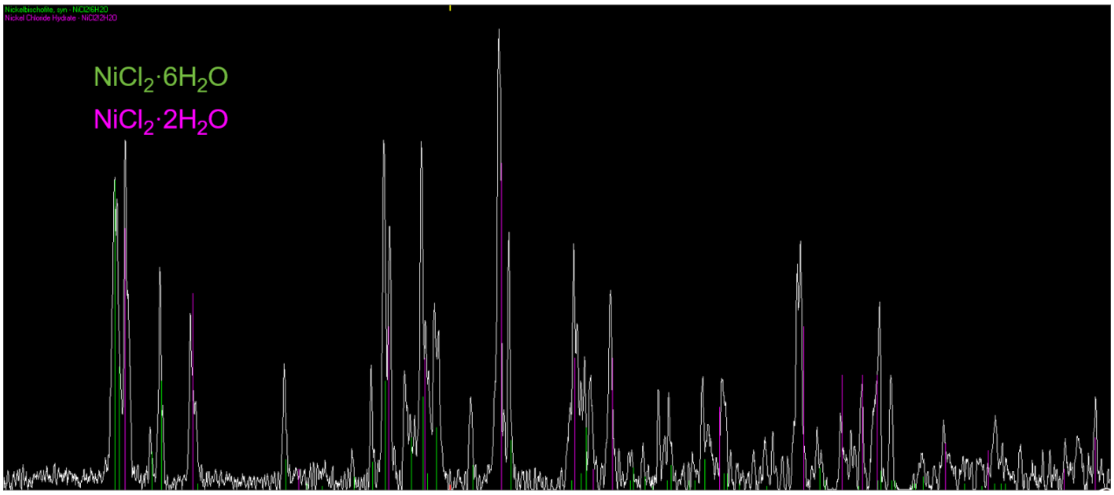

Fig. 12. XRD fitting results for the green crystals are consistent with $NiCl_2{\cdot}6H_2O$ and $NiCl_2{\cdot}2H_2O$.

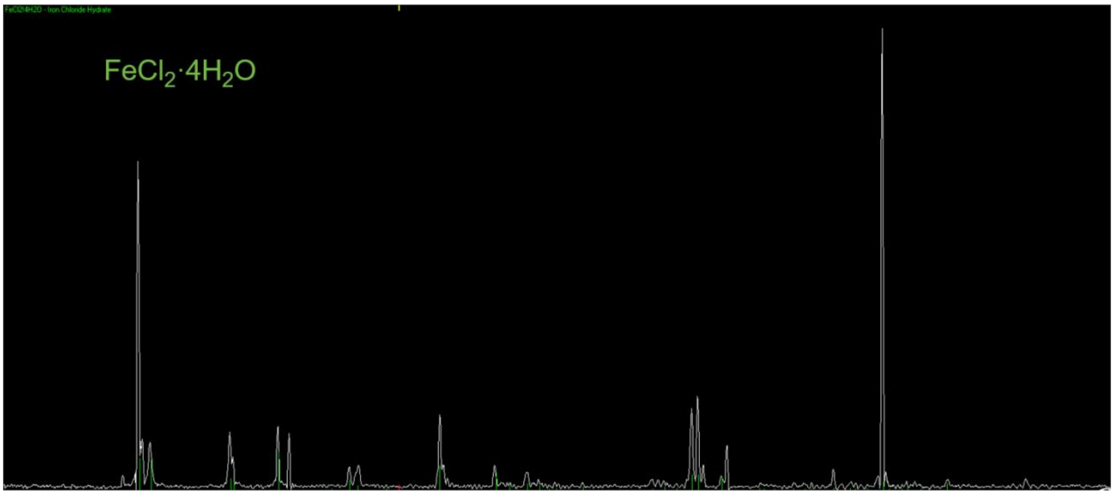

Fig. R13. XRD fitting results for the yellow crystals are consistent with $FeCl_2{\cdot}4H_2O$.

Fig. R11 has been marked as Supplementary Fig. 29 in the supplementary material.

Reviewer #3: After reviewing the revised manuscript, I recommend the following correction before publication: Indicate clearly where the Introduction section starts and place the results and discussion of this work under the Results and Discussion section. Additionally, summarize the information in each section so that readers can grasp the key points of the work more quickly.

Response: We appreciate the reviewer for raising this insightful comment. The start of the introduction section has been clearly indicated; all study findings and analytical discussion are consolidated in the dedicated "Results and Discussion" section; each section incorporates a concise summary (**page 5, page 6, page 8, page 10, page 11, page 12, and page 13**). Please kindly see below:

Based on the above results, the optimal reaction parameters are identified as follows: tandem catalysis configuration, $Ni_{9.5}Fe/C$ as the catalyst precursor, 600 W microwave power, a 1:1 mass ratio of plastic to catalyst (3 g each) in the bottom layer, and 3 g of catalyst in the top layer (**Page 5**).

Results presented above suggest that PVC can effectively enhance the cyclic lifespan and catalytic stability of FeNi/Ni/C catalysts, while microwave catalysis exhibits lower energy requirements and more favorable catalytic performance compared to traditional thermal catalysis (**Page 6**).

Collectively, the carbonaceous product derived from FeNi/Ni/C-catalyzed plastic decomposition consists of multi-walled carbon nanotubes, which exhibit promising application prospects for lithium battery energy storage and electromagnetic wave absorption (**Page 8**).

Therefore, chlorine derived from PVC plastics reacts with Fe and Ni species in FeC-FeNi/Ni/C catalyst precursors to form ferric chloride and nickel chloride. These compounds are subsequently reduced to FeNi alloys by hydrogen generated during plastic decomposition, yielding stable FeNi/Ni/C catalysts that notably enhance catalytic stability and activity (**Page 10**).

Overall, FeNi/Ni/C catalysts demonstrate robust catalytic stability and facilitate the efficient conversion of multi-component plastic mixtures, including LDPE, HDPE, PP, PS, PVC, and PTFE, and landfilled plastic blends to hydrogen and carbon nanotubes (**Page 11**).

In brief, underlying mechanistic studies suggest that nickel facilitates C-H bond cleavage during plastic decomposition, whereas iron promotes carbon nanotube nucleation and growth; these complementary effects enable the efficient conversion of plastic to hydrogen and carbon nanotubes via synergistic catalytic interactions (**Page 12**).

Thus, this approach has substantial economic and social benefits, which supports its wider application (**Page 13**).
